# The emergence of putative epistatic mutations and iSNVs in SARS-CoV-2 XBB.1.16 variants linked with alteration in immunogenic determinants

**DOI:** 10.3389/fimmu.2026.1846973

**Published:** 2026-08-20

**Authors:** Mukesh Kumar Jogi, Sristy Shikha, Pushpendra Singh, Devesh Sharma, Shreyansh Shreyansh, Prem Mishra, Aiswariya Priyadarsini, Aahamya Priyadarsini, Deokrishna Kumar Choudhary, Meenu Jain, Ritu Sagar, Anuj Kumar, Robin Marwal, Md Kausar Neyaz, Dheeraj Dube Prakashchand, Rakesh Gupta, Shalini Singh, Pramod Kumar

**Affiliations:** 1 Division of Molecular Biology, Indian Council of Medical Research (ICMR)-National Institute of Cancer Prevention and Research, Noida, India; 2 Division of Environmental Biotechnology, Genetics and Molecular Biology (EBGMB), Indian Council of Medical Research (ICMR) - National Institute for Research in Environmental Health, Bhopal, India; 3 Department of Biotechnology, Central University of Haryana, Mahendergarh, India; 4 Department of Biochemistry, Govt Institute of Medical Sciences, Greater Noida, India; 5 Department of Biotechnology, Anugrah Narayan (A.N.) College, Patna, India; 6 Department of Biological Sciences, Indian Institute of Science Education and Research (IISER), Berhampur, India; 7 School of Mechanical and Materials Engineering, Indian Institute of Technology (IIT) Mandi, Mandi, Himachal Pradesh, India; 8 Viral Research and Diagnostic Laboratory (VRDL), Gajra Raja Medical College, Gwalior, Madhya Pradesh, India; 9 Faculty of Medical Research, Academy of Scientific and Innovative Research (AcSIR), Ghaziabad, India

**Keywords:** epistasis, genomics, immune evasion, iSNVs, SARS-CoV2

## Abstract

The SARS-CoV-2 XBB variants have been proposed to evolve towards immune evasion against vaccination or natural infection, which may contribute to higher transmissibility. The XBB.1.16 independently emerged due to accumulation of two important substitutions, E180V and T478R in the spike protein. Its pseudoviral infectivity and evasion of humoral immunity were similar to XBB.1 and XBB.1.5. In March 2023, XBB.1.16 had outcompeted other dominant XBB variants in India, which indicate a potential growth advantage. Here, intra-host single nucleotide variations (iSNV) and mutations were screened in SARS-CoV-2 genomes in closely related individuals at two time points: at symptoms onset, and during recovery. The prominence of putative epistatic iSNVs (E180V, G184V, G252V, D253G, and P521S/T) in XBB.1.16 variants were detected during the recovery phase. E180V exhibits mutational constellations with the G252V and P521T in a subset of samples, and this pattern was also detected in contemporary SARS-CoV-2 genomes. Higher order protein structural predictions suggested that the putative epistatic interactions among E180V, G184V, and G252V, D253G may be associated with S protein folding and structural stability. This study involving genomics and computational analyses highlights the potential role of these putative epistatic interactions in immune evasion, which may have contributed to dominance of XBB variants.

## Introduction

SARS-CoV-2 has diversified constantly since its emergence in late 2019, generating successive variants of concern that differ in transmissibility, antigenic profile, and capacity to evade host immunity (1, 2). The rapid global implementation of vaccines based on the spike glycoprotein substantially reduced severe coronavirus disease 2019 (COVID-19), but simultaneously imposed strong selective pressure on circulating viruses (1, 3). Most of the resulting phenotypic diversification is attributable to amino-acid substitutions in the spike protein, particularly within the receptor-binding domain (RBD) and N-terminal domain (NTD), which jointly determine angiotensin-converting enzyme 2 (ACE2) engagement and recognition by neutralizing antibodies (2). Large-scale genomic surveillance has shown that antigenically drifted or recombined spike lineages are repeatedly selected in populations with widespread infection- and vaccine-induced immunity (4, 5).

The Omicron lineage represents a major inflection point in this evolutionary trajectory, characterized by an unusually high burden of spike mutations and extensive remodeling of key antigenic determinants (6). Omicron sublineages maintain high-affinity ACE2 binding yet exhibit marked reductions in serum neutralization and diminished recognition by many therapeutic monoclonal antibodies, reflecting alterations in both B-cell and T-cell epitopes within the RBD and NTD (7, 8). Within the BA.2 background, recombination between BJ.1 (BA.2.10.1.1) and BA.2.75 (BM.1.1.1) generated the XBB recombinant, which disseminated rapidly and was associated with substantial escape from vaccine-elicited humoral immunity (9). Subsequent derivatives, including XBB.1 and XBB.1.5, accumulated additional substitutions across the RBD and NTD that further enhanced immune evasion while preserving efficient viral entry and transmission (10).

XBB.1.16 emerged as a prominent descendant, harboring characteristic substitutions E180V in the NTD and T478R in the RBD on an XBB.1-like background (10). By March 2023, XBB.1.16 had displaced contemporaneous variants in India and exhibited a higher effective reproductive number than XBB.1 and XBB.1.5, despite broadly comparable pseudoviral infectivity and resistance to neutralizing antibodies *in vitro* (10). These observations suggest that factors beyond bulk humoral escape-such as fine-scale modulation of spike structural stability, alterations in epitope architecture, or effects on T-cell recognition-may contribute to the fitness advantage of XBB.1.16 and related sublineages, but the underlying mechanisms remain incompletely defined (5, 8).

At the structural level, both RBD and NTD mutations in Omicron and XBB variants appear to be constrained by epistatic interactions that balance immune escape with preservation of spike conformational integrity (2, 5). Defined constellations of substitutions can cooperatively remodel antibody epitopes while maintaining the folding and dynamics required for receptor binding and membrane fusion, and several NTD alterations have been linked to loss of binding by strain-restricted neutralizing antibodies and altered sensitivity to broadly reactive or enhancing NTD-directed antibodies (11, 12). It is yet unclear how the particular combination of NTD and RBD mutations in XBB.1.16, including E180V and T478R, reshapes this epistatic landscape and influences humoral and cellular recognition.

In the present study, genomic analysis was conducted in SARS-CoV-2 XBB.1.16 and closely related XBB-lineage viruses circulating in India. The spike mutation patterns including intra-host single-nucleotide variants (iSNV) were analyzed followed by computing how these changes influenced the structural stability and antigenic architecture of the NTD and RBD. The consequences of these mutational constellations for B-cell and T-cell epitope presentation were also predicted, with particular emphasis on mechanisms of immune evasion that may confer a viral structural fitness advantage over co-circulating variants.

## Methods

### Sample collection, RNA extraction, and RT-qPCR

This was a retrospective study using archived, de-identified secondary specimens collected during routine diagnostics. The informed consent wasn’t required, i.e. that the Institutional Ethics Committee (ICMR-NICPR, Noida) determined consent was not applicable for secondary, fully anonymized samples. Nasopharyngeal and oropharyngeal swab (NPS/OPS) specimens that tested positive for SARS-CoV-2 via PCR were selected for this study. Samples were collected from four closely related individuals at two time points: symptom onset and recovery. An additional 30 RT-PCR-positive specimens, collected from the Government Institute of Medical Sciences (GIMS), Noida, were also included with vaccination history, reinfection confirmation, severity, and outcome status (**Supplementary Excel Sheet 1**). All samples were transported in a cold chain to ICMR-NICPR, Noida, and then thermally inactivated at 56 °C for 30 min. Total RNA was extracted, and viral loads were quantified by reverse transcription quantitative polymerase chain reaction (RT-qPCR), as described previously (13, 14). The longitudinal paired analysis (onset & recovery) was conducted on four individuals who were sampled sequentially over time. In addition, the study includes a cross-sectional dataset comprising 30 supplementary specimens, which were utilized for mutational landscape characterization and potential interaction analyses.

### cDNA library preparation and quality control

Purified RNA was quantified using the Qubit hsRNA Kit (Life Technologies, USA). Complementary DNA (cDNA) was synthesized from each isolate using SuperScript™ VILOTM Master Mix, as described previously (15). Libraries were then prepared using the Ion AmpliSeq™ Library Kit Plus with primer pools 1 and 2. The amplicons were partially digested with FuPa reagent, followed by ligation of specific barcode adaptors (IonCode™ adaptors 0401-0496). Barcoded libraries were purified using AMPure XP beads (16).

### Enrichment of purified library and whole genome sequencing

A Qubit dsDNA High Sensitivity Kit was used for quantification of the amplified library and the reading was done using Qubit 4.0 Fluorometer (Thermo Fisher Scientific, Waltham, MA, USA). The libraries were subsequently diluted to obtain a concentration of 100 pM and pooled accordingly before being processed in the Ion Chef System. This system facilitated the generation of template-positive ion sphere particles (ISPs) containing clonally amplified DNA, using the Ion S5 Chef solutions and reagents. The template-positive ISPs were enriched using the Ion Chef System and automatically loaded onto an Ion 540™ Chip. Subsequently, the loaded chip was kept on the Ion GeneStudio S5 NGS platform for sequencing.

### Plugins used in sequencing

Sequencing reads were aligned to the SARS-CoV-2 reference genome (NC_045512.2) using Torrent Suite v5.18.1. Variant detection and annotation were performed with the Coverage Analysis (v1.3.0.2), Variant Caller (v5.16.0.0; default “Generic-S5/S5XL (540)-Germ Line-Low Stringency” settings), and COVID19AnnotateSnpEff (v1.3.0.2) plugins, the latter tailored for SARS-CoV-2 variant effect prediction (15).

### Retrieval and alignment of sequences

FASTA sequences were assembled from ion torrent suites IRMA algorithms (17), and aligned using MUSCLE Tool of MEGA11 (18).

### Single-nucleotide variant and intra-host iSNV analysis and data visualization

Further analyses focused on single-nucleotide variant (SNV) identification and curation. Aligned sequences were scanned for mutations in MEGA 11, and candidate variants were validated against Torrent Variant Caller (TVC) output, BAM/BAI files inspected in Integrative Genomics Viewer (IGV v2.16.2), and the SARS-CoV-2 reference genome NC_045512.2 (19), (20). Detected variants were cross-checked with the Variant Caller (v5.16.0.0) VCF files, and discrepancies were resolved by manually correcting positions where mutations were clearly supported by the raw read data but absent from the VCF. The resulting high-confidence variant set was incorporated into curated consensus FASTA sequences, which were subsequently submitted to the NCBI and GISAID repositories.

iSNVs were identified by extracting single-nucleotide polymorphisms (SNPs) into a binary mutation matrix (0/1), compiled as a CSV file, representing the presence or absence of mutations relative to the sample set. iSNVs were defined as nucleotide positions at which the minor allele was present at a frequency of 5%, requiring ≥100× total coverage with ≥10 uniquely mapped reads per position, and strand-bias filtering requiring the minor allele be supported by ≥2% of reads on each strand independently. All iSNV calls were further validated by cross-referencing with the Torrent Variant Caller (TVC) VCF output and manual inspection of BAM files in IGV v2.16.2. Ion Torrent sequencing achieved a mean depth of >1,500x (minimum 30–50x per amplicon) with >98% genome coverage, required for reliable consensus and variant calling. Amplicon primer sequences were trimmed prior to alignment, and amplicons showing anomalous/discordant coverage relative to neighboring regions were excluded from iSNV analysis at overlapping positions. iSNVs were characterized using BAM files alongside assembled genomes to track their emergence (>60%) in sequentially infected individuals at symptom onset and their subsequent distribution in genomes during recovery. iSNVs mapping to immunogenic or immunodominant regions were further examined for potential functional and immune-related implications (21).

For downstream visualization, the curated mutation matrix was used to generate clustered heatmaps in RStudio, employing packages such as RColorBrewer (22), viridisLite (23), pheatmap (24), readr (R Core Team (25), 26), and the hclust function to depict genetic variation patterns among samples (27).

### Epitope mapping

Epitope mapping for the spike protein was performed on selected samples representing distinct phylogenetic groups. CD4^+^ T cell epitope prediction was carried out using NetMHCIIpan 4.3 (https://services.healthtech.dtu.dk/services/NetMHCIIpan-4.3/) (28) with default settings and the following HLA class II alleles, selected based on published data (15): DRB1_0101, DRB1_0405, DRB1_0701, DRB1_0802, DRB1_1201, DRB1_1302, HLA-DPA10103-DPB110101, HLA-DPA10201-DPB110201, HLA-DPA10301-DPB110301, HLA-DQA10101-DQB10201, HLA-DQA10301-DQB10301, HLA-DQA10401-DQB10401, HLA-DQA10501-DQB10501, HLA-DQA10102-DQB10202, and HLA-DQA10402-DQB10402. Predicted epitopes were ranked by %Rank_EL, and peptides with %Rank_EL ≤ 1 were retained as high-confidence binders.

CD8^+^ T cell epitope prediction for the same spike protein sequences was performed using NetMHCpan 4.1 (https://services.healthtech.dtu.dk/services/NetMHCpan-4.1/) with default parameters and the following HLA class I alleles, chosen according to literature (15): HLA-A*02:01, HLA-A*02:03, HLA-A*02:05, HLA-A*02:06, HLA-A*24:02, HLA-A*11:01, HLA-B*07:02, HLA-B*08:01, HLA-B*13:02, HLA-B*15:01, HLA-B*18:01, HLA-B*35:01, HLA-B*40:06, HLA-B*46:01, HLA-B*51:01, HLA-B*52:01, HLA-C*01:02, HLA-C*04:01, HLA-C*06:02, and HLA-C*07:01. CD8^+^ epitopes were filtered using a stringent threshold of %Rank_EL ≤ 0.5 to define strong binders.

Linear B cell epitope prediction was conducted using ABCpred (https://webs.iiitd.edu.in/raghava/abcpred/) with a threshold score of 0.51 and an 18-mer window length. Predicted B cell epitopes above the threshold were further screened to identify regions harboring putative epistatic iSNVs (E180V, G184V, G252V, D253G, and P521S/T) (29).

### Structural assessment of residue exposure and antibody-spike interfaces

#### Solvent-accessible surface area analysis

Residue exposure was quantified by computing solvent-accessible surface area (SASA) with Getarea using default settings (1.4 Å probe and standard atomic radii). Per-residue SASA values were interpreted in line with established solvent accessibility frameworks, converting absolute SASA into relative solvent accessibility and classifying residues with low relative accessibility (approximately <20%) as buried and those above this threshold as exposed. SASA of key spike residues (H146, E180, D253) was calculated using the Getarea server with default parameters. Values were compared between wild-type and mutant models to assess changes in residue exposure at epitope sites.

#### Interface analysis (ePISA)

Antibody-spike interfaces were delineated and quantified using PDBePISA, which computes buried surface area as the loss of SASA upon complex formation using its standard Lee-Richards-type surface algorithm. Antibody-spike interfaces were characterized with ePISA, which reports buried surface area and interfacial contacts. Wild-type and mutant complexes were compared to determine mutation-induced changes in interface burial and contact patterns.

#### Structural visualization using UCSF ChimeraX

Point mutations (SNVs) were modelled in UCSF ChimeraX with the Rotamers tool, which samples side-chain conformations from a backbone-dependent rotamer library derived from high-resolution protein structures. For each substitution, rotamers were ranked by their library probability and steric compatibility, and high-probability, low-clash conformations were selected for downstream SASA and ePISA re-analysis to assess how the mutation altered residue exposure and antibody-spike interface geometry. Spike receptor-binding domain (RBD) complexes with class 1–4 antibodies (C105 Fab, LY-CoV555, S309 Fab, C118 Fab respectively) and N-terminal domain (NTD) complexes with 4A8, 8D2, and TXG-0078 (PDB IDs 7C2L, 7DZX, 8SWH) were examined in UCSF ChimeraX to map mutated residues within epitopes and assess their spatial relationship to antibody contact regions.

To directly validate these predictions and quantify the local conformational consequences of each iSNV at atomic resolution, 100 ns all-atom molecular dynamics simulations of the wild-type NTD and four single-mutant systems were performed independently and in combination (**Supplementary Table 1**). Step by step simulation details have been mentioned in supplementary method section.

### Statistical analysis

All statistical analyses were performed using R software (version 4.3.2; R). Continuous variables were summarized as mean ± standard deviation (SD) or median with interquartile range (IQR), as appropriate, whereas categorical variables were presented as frequencies and percentages. Comparisons of viral load, represented by RT-PCR cycle threshold (Ct) values, among different vaccination status and age groups were performed using one-way analysis of variance (ANOVA). Pearson’s correlation coefficients were calculated to evaluate pairwise associations between spike mutation allele frequencies, and the resulting correlation matrix was visualized as a heatmap. All graphical visualizations were generated using the ggplot2, corrplot, dplyr, tidyr, and reshape2 packages in R. All statistical tests were two-tailed, and a *p* value <0.05 was considered statistically significant.

## Results

The clinical association analysis of the subject cohort (n= 34, excluding the 4 subjects whose sample was collected during recovery phase as shown in **Table 1**) shows that Ct values did not significantly vary across any of the clinical or demographic variables tested. For the age group comparison (≤30, 30–45, and >45 years), mean CT values were 21.71, 22.96, and 20.68, respectively, showing no statistical difference (p = 0.252). Similarly, comparing symptomatic vs. asymptomatic cohorts also not statistically significant (p = 0.143). Finally, analyzing vaccine doses (Unvaccinated vs. 2 Doses vs. 3 Doses) showed highly uniform mean CT values respectively, with no significant differences observed (p = 0.881). These findings indicate that age, symptom status, and vaccination dose level did not significantly influence viral load dynamics in this study population (**Supplementary Figures 1A–C**). This may be due small cohort size.

**Table 1:** Longitudinal profiling of amino acid (AA) substitutions and allelic frequencies within the SARS-CoV-2 spike protein across initiation (I) and recovery phases (R). Mutation frequency is expressed as percentage in the dataset.

POS	REF	ALT	AA	COD POS	Patient-1		Patient-2		Patient-3		Patient-4	
					NICPR445 (I)	NICPR433 (R)	NICPR434 (I)	NICPR451 (R)	NICPR448 (I)	NICPR454 (R)	NICPR442 (I)	NICPR436 (R)
22101	A	T	E180V	2	0	0	0	0	0	0	0	71
22113	G	T	G184V	2	0	0	0	0	0	0	86	0
22132	G	T	R190S	3	28	0	0	50	0	50	0	0
22281	C	T	T240I	2	0	0	0	0	30	0	0	0
22317	G	T	G252V	0	0	0	0	0	0	0	11	99
22320	A	G	D253G	2	11	0	0	81	68	0	11	0
22342	T	C	A260A	3	0	0	0	0	0	0	0	0
22775	G	A	D405N	1	100	100	100	100	100	67	100	100
22786	A	C	R408S	3	86	83	83	86	75	67	0	88
23123	C	T	P521S	1	0	0	3	94	99	0	100	0
23202	C	T	T546I	2	0	0	0	0	0	0	0	0
23335	T	C	S591F	3	16	11	24	18	18	0	75	73
23952	T	G	F797C	2	11	25	21	8	0	0	13	22
23957	G	T	G798C	1	11	16	14	2	6	0	0	13
24125	T	G	F855V	1	13	11	11	18	10	0	29	12
24151	T	C	P863P	3	0	0	0	0	0	20	0	0
24200	G	A	G880S	1	0	0	0	0	0	43	0	0
24357	G	C	G932A	2	28	1	1	7	6	33	0	0
24398	G	A	G946R	1	0	0	20	0	0	0	0	14
24645	A	G	K1028R	2	0	0	0	0	0	38	0	0
24940	T	A	C1126Y	3	0	0	0	21	9	13	0	0

POS: Genomic position relative to the SARS-CoV-2 reference genome (NC_045512.2).

REF: Reference allele present in the NC_045512.2 genome.

ALT: Alternate allele identified through NGS.

AA: Resulting amino acid substitution in the Spike (S) protein.

COD POS: Position of the nucleotide within the specific codon (1, 2, or 3).

Initiation (I): Viral genome captured at the time of symptom onset.

Recovery (R): Viral genome captured during the patient’s recovery phase.

Numerical Values: Represent the percentage frequency (depth of coverage) of the alternate allele within the intra-host viral population.

Further a subset of samples showing putative epistatic changes, the association of viral load (Ct) between the XBB.1.16-like and XBB.2.3-like variants (p=0.678) or among different age groups (p=0.187) did not differ significantly this may be due to very small cohort size. In contrast, vaccination status showed a significant association with Ct values (2,9): 5.69, p=0.0253). Individuals who had received a booster dose exhibited significantly higher Ct values (lower viral load) compared with unvaccinated individuals, while double-dose recipients displayed intermediate Ct values (**Supplementary Figures 1D–F**). However, these observations are limited to a relatively small sample size, warranting validation in larger cohorts.

The longitudinal genomic analysis of the SARS-CoV-2 spike protein across the study cohort indicated a highly dynamic intra-host evolutionary trajectory, characterized by a transition from early XBB lineages toward more advanced sublineages. A stable Omicron BA.2-derived backbone was confirmed across all patients by the near-fixation of core substitutions, specifically D405N (POS 22775) and R408S (POS 22786), which were maintained at high frequencies ranging from 67% to 100% throughout the infection. Beyond this conserved framework, the data captured significant intra-host divergence marked by the emergence of signatures defining the XBB.1.16 and XBB.2.3 sub-variants. Notably, Patient-4 exhibited a distinct evolutionary shift toward the XBB.1.16 lineage during the recovery phase, evidenced by the emergence of E180V (POS 22101), which rose from undetectable levels at symptom onset to 71% frequency, alongside the fixation of G252V (POS 22317) at 99%.

The transition toward advanced variants was further highlighted by rapid selective sweeps and the fluctuation of lineage-specific markers within individual hosts. In Patient-3, the XBB.2.3 marker P521S (POS 23123) underwent a dramatic shift from a 3% minority variant at symptom onset to 94% frequency during recovery, suggesting a possible substantial fitness advantage under potential host-specific selection pressure. Conversely, some substitutions appeared transient or underwent intra-host reversion; for instance, G184V (POS 22113) was identified at 86% during the initial infection in Patient 4 but was absent in the recovery genome. Similarly, the D253G (POS 22320) substitution demonstrated high variability, appearing at 81% in Patient-2 during recovery and 68% in Patient 3 at symptom onset, while reverting from 11% to 0% in Patient 4.

**Figure 1:**
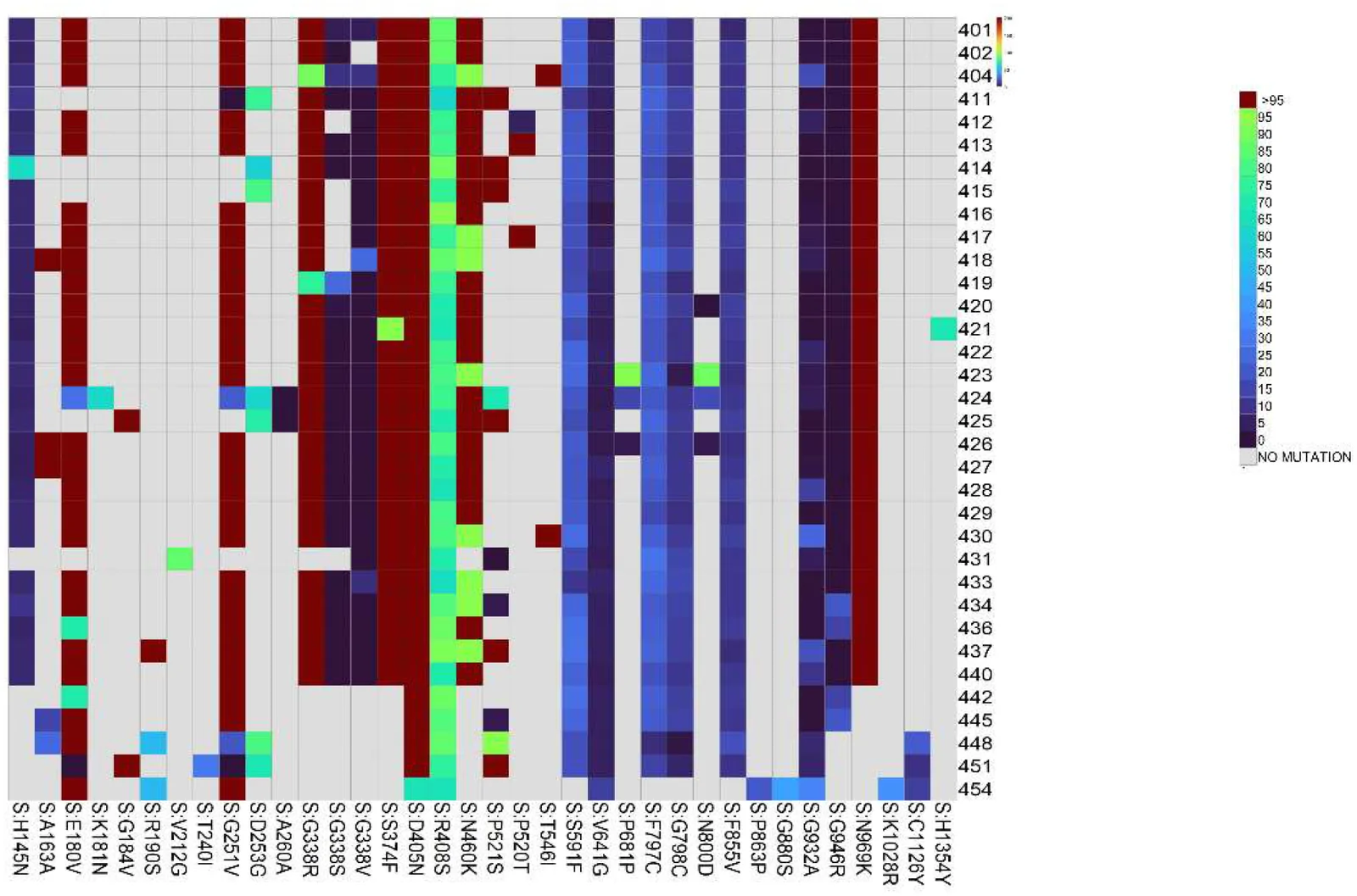
A heatmap representing the percentage iSNVs in the SARS-CoV-2 genomes of the structural protein region.

These coordinated allelic fluctuations were consistent with an underlying putative epistatic change relating to structural stability, and this may play a role in predicted immune evasion. The results indicate that the emergence of XBB.2.3 markers (P521S and D253G) generally occurred in the absence of XBB.1.16 signatures (E180V and G252V), and vice versa. The heatmap (**Figure 1**) also provides robust evidence of putative epistatic phenomena, favored in a mutually exclusive manner. Structural modelling supports this finding, suggesting that simultaneous mutations such as E180V and G184V may disrupt alpha-helix stability, necessitating a “balancing” effect through coordinated substitutions. While dominant markers dictated the primary evolutionary path, several low-frequency iSNVs including F797C, G798C, and F855V, persisted at frequencies between 8% to 29%, representing a diverse quasispecies reservoir that facilitates the ongoing adaptation of the virus within the host environment (30).

### Intra-host single nucleotide variations

The longitudinal genomic analysis of the SARS-CoV-2 structural protein region has shown a highly dynamic intra-host mutational landscape, characterized by the emergence of distinct iSNVs and putative epistatic interactions between the onset of symptoms and the recovery phase. Analysis of the iSNV distribution (**Figure 1**) demonstrated a significant genomic concentration within the S gene, which accounted for 88% of total identified variations, while the N and M genes represented only 10% and 2% of the mutational burden, respectively. Within the spike protein, 22% of iSNVs were localized to the receptor-binding domain (RBD), highlighting this region as a critical hotspot for host-driven selection. Notably, the D253G genomic position exhibited the highest frequency of iSNVs across the cohort, reflecting intense selection pressure from the host immune response on this specific immunogenic site.

The heatmap (**Figure 1**) also displays a suggestive pattern of epistatic relationship, where specific nucleotide substitutions appeared to influence the presence or absence of others in a coordinated manner. A prominent reciprocal relationship was observed among subvariant markers: the presence of the XBB.2.3-associated substitutions P521S and D253G appeared to inhibit the emergence of XBB.1.16-defining markers E180V and G252V, and vice versa. This pattern suggests a potentially linked evolutionary trajectory where the virus may prioritize specific mutational combinations, which might balance predicted immune evasion with structural integrity. Furthermore, specific clusters of shared substitutions including P521S, D253G, G184V, and T478K were identified in select genomes, reinforcing the coordinated nature of these intra-host genetic alterations (**Table 2**).

**Table 2:** Distribution of nucleotide variants (%) at specified genomic positions within two distinct read types demonstrating epistatic phenomena associated with variant mutations.

POS	REF	ALT	AA	NICPR 448	NICPR 425	NICPR 442	NICPR 411	NICPR 414	NICPR 415	NICPR 451	NICPR 424	NICPR 413	NICPR417	NICPR445	NICPR436
22101	A	T	E180V	1	0	0	0	0	0	100	27	100	100	65	71
22113	G	T	G184V	98	99	86	0	0	0	0	0	0	0	6	0
22317	G	T	G252V	1	0	11	0	0	0	19	21	99	100	89	99
22320	A	G	D253G	68	72	11	77	58	80	81	61	0	0	11	0
23123	C	A	P521T	0	0	0	0	0	0	0	0	100	99	0	0
23123	C	T	P521S	99	100	100	99	99	99	94	69	0	0	0	0

Color coding indicates distinct mutational constellations identified from the allelic-frequency profiles. Blue-shaded samples represent the G184V/P521S-associated mutational pattern; pink-shaded samples represent the P521S/D253G-associated pattern; cyan-shaded samples represent a mixed/intermediate pattern in which XBB.2.3- and XBB.1.16-associated substitutions co-occur; orange-shaded samples represent the E180V/G252V/P521T-associated pattern. The light-blue shading denotes samples showing predominantly XBB.1.16-associated substitutions with residual/sub-consensus variants. Colors are used solely to facilitate visualization of sample-wise mutational groupings and do not represent mutation frequency, statistical significance, or a quantitative scale.

The temporal dynamics recorded between at symptom onset and recovery phases suggest that these putative epistatic iSNVs facilitate rapid viral adaptation. Dominant mutations such as E180V and P521S were primarily detected or significantly enriched during the recovery phase, implying a potential selective sweep within the host environment. These findings support the hypothesis that intra-host evolution may be influenced by immune-mediated pressure, potentially selecting for variants that conserve predicted structural stability of the S protein while simultaneously altering immunogenic determinants which could provide a fitness advantage over dominant circulating strains.

At several positions, a specific consensus nucleotide and an iSNV were detected at the same genomic site within the same sample (sample 433, spike position G338S, G338V G338R) suggesting the coexistence of multiple viral subpopulations. This pattern reflect either simultaneous circulation of two variant lineages at the time of sampling or immune-driven selection acting on the viral quasispecies within the host.

The nucleotide substitution pattern demonstrated that certain substitutions influence the presence or absence of others, indicating putative linkage between these SNPs and providing evidence of putative epistatic relationships. Additionally, a specific consensus nucleotide and an iSNV were simultaneously observed at the same genomic position within the same sample, suggesting the co-existence of multiple viral subpopulations. This may reflect either the concurrent circulation of distinct variant lineages at the time of sampling or the impact of immune-driven selection on the intra-host viral quasispecies.

### Epistatic mutation networks shaping XBB and XBB.1.16 spike evolution

The comparative analysis of nucleotide variants across the cohort (**Table 2**) indicated a non-random distribution of substitutions, defining two distinct and mutually exclusive evolutionary trajectories within the XBB lineage. These pathways suggest the putative epistatic constraints where specific genetic backgrounds influence the presence or absence of subsequent substitutions. A prominent evolutionary axis was identified where the lineage-defining markers for XBB.2.3, specifically P521S and D253G, are genetically linked with the G184V substitution. In genomes such as NICPR448 and NICPR425, P521S and D253G approached near-fixation (approximately 99-100% and 68-72%, respectively) in concert with G184V (98-99%), while the XBB.1.16-associated substitutions E180V and G252V were completely absent, implying a strong negative epistatic relationship between these sets of mutations. Patient 4 (NICPR442) showed an early manifestation of this trajectory at initial infection, with fixed P521S and high-frequency G184V (86%), whereas E180V and G252V remained at baseline.

A secondary and contrasting evolutionary pathway was observed, primarily characterizing the transition toward the XBB.1.16 sub-lineage. In genomes NICPR413, NICPR417, and the recovery sample of Patient 4 (NICPR436), E180V and G252V reached near-fixation (typically >70% and >99%, respectively), accompanied by complete loss of P521S and D253G, consistent with a reciprocal, negatively epistatic relationship or mutational trade-off between XBB.2.3- and XBB.1.16-defining markers. In NICPR413 and NICPR417, E180V and G252V co-occurred with P521T (99-100%) instead of P521S, suggesting branching within the XBB.1.16 genetic landscape.

Across the dataset, P521S and D253G were consistently present without G252V and E180V in viral genomes NICPR411, NICPR414, NICPR442, NICPR425, NICPR424, NICPR415, and NICPR451, whereas the remaining genomes displayed the opposite pattern, harboring G252V, E180V, and T478R while lacking P521S and D253G. Additional epistatic patterns were evident: in NICPR413 and NICPR417, E180V co-segregated with both G252V and P521T, while in NICPR451 and NICPR424, E180V co-occurred with sub-consensus G252V (20% of reads) and high-frequency D253G (81% and 61%, respectively) in the presence of P521S but absence of P521T. Finally, G184V was consistently associated with D253G and P521S in genomes NICPR425, NICPR442, and NICPR448. Together, these observations indicate mutational constellation in spike are shaped by putative epistatic interactions that structure the genetic landscape of the XBB sublineages.

Correlation analysis of allelic frequencies demonstrated coordinated evolution among several spike substitutions. Strong positive correlations were observed between E180V and G252V (r = 0.82), D253G and P521S (r = 0.83), and G252V and P521T (r = 0.65), referring that these mutations frequently co-occur. In contrast, strong inverse relationships were identified between G252V and P521S (r = −0.99), G252V and D253G (r = −0.86), E180V and P521S (r = −0.77), and D253G and P521T (r = −0.57), supporting the existence of alternative lineage-defining haplotypes rather than random mutation accumulation (**Supplementary Figure 1H**).

### Mutational landscape of the SARS-CoV-2 spike protein reveals immune-driven hotspots in NTD and RBD of NICPR genomes

The spike gene in these genomes reads like an evolutionary landscape, with the N-terminal domain (NTD) and receptor-binding domain (RBD) carrying most of the mutations from immune pressure. In the NTD, a deletion spanning residues L242-A243 in NICPR411 reshapes a region known to be a key antigenic “supersite,” a pattern that mirrors deletions in other variants that blunt neutralizing antibody binding. This is coupled with RBD substitutions such as T385I and T478Q, changes in a domain that directly contacts ACE2 and is repeatedly remodeled in variants to fine-tune the balance between receptor binding and immune escape.

Across the broader RBD segment (positions 331-524), the genomes show a mosaic of polarity-shifting substitutions that subtly re-engineer the spike surface. In 8% of sequences, polar-to-non-polar changes disrupt hydrogen bonds and can destabilize local structure; 5% retain polarity but alter charge and side-chain volume, adjusting how the RBD engages antibodies or neighboring residues. Non-polar-to-non-polar swaps appear more conservative, but non-polar-to-polar transitions, such as P521T in NICPR417 and NICPR413, locally reduce hydrophobicity and may increase solvent exposure at the edge of the RBD.

Position 478 stands out as a true hotspot, with T478K, T478R, T478Q, and T478N all represented, echoing the importance of this site in many globally circulating variants. NICPR411 carries T478Q and NICPR415 carries T478N, both polar-to-polar exchanges that tweak hydrogen-bonding and surface electrostatics without introducing a charge, in contrast to T478K/R changes that are known to boost positive surface charge and can enhance ACE2 binding and immune evasion. Other substitutions such as T259A, T326I, and A344V layer additional structural adjustments by altering local packing and flexibility around the RBD.

**Figure 2:**
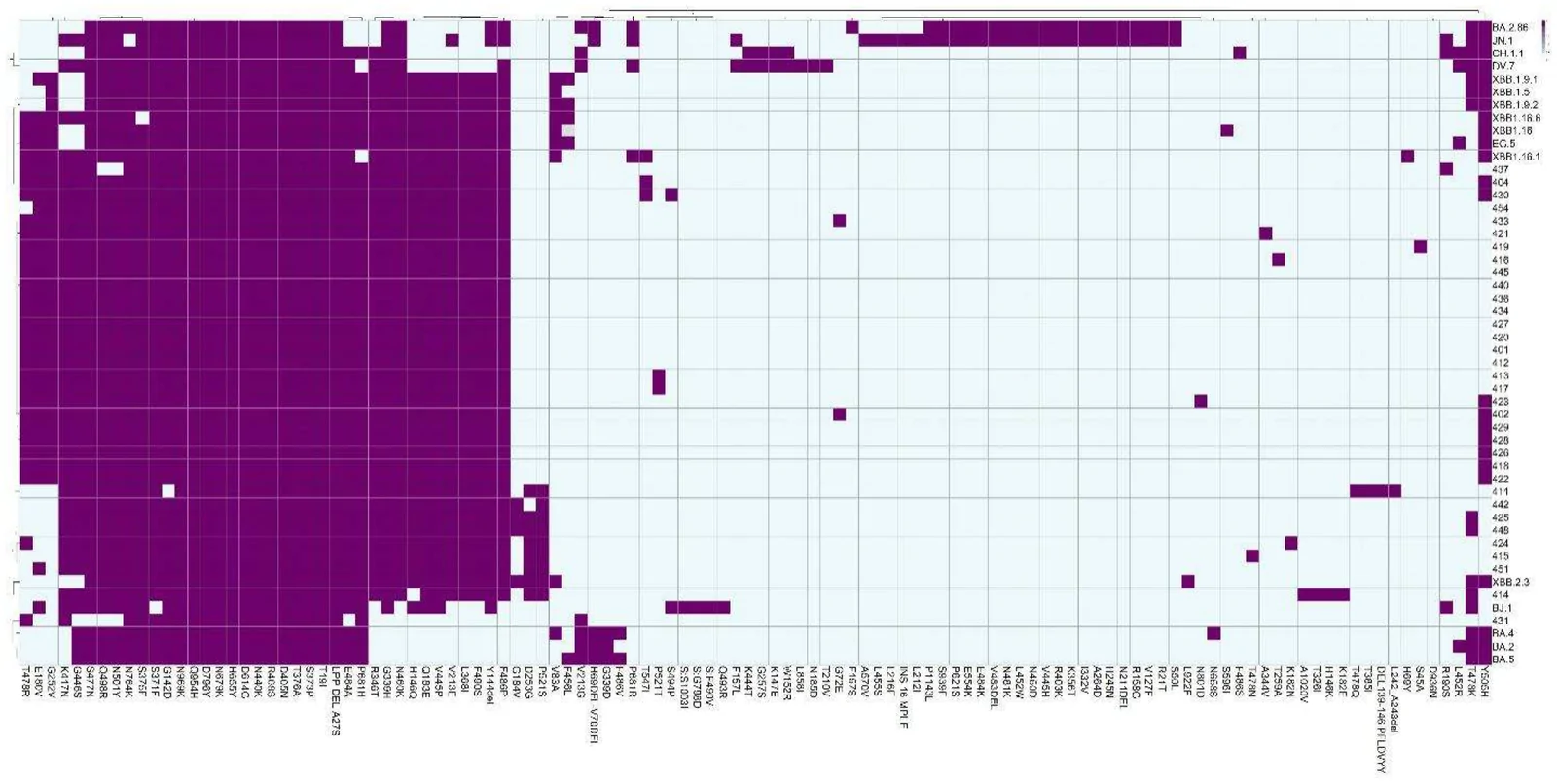
A heatmap illustrating the mutations identified in the spike region of the SARS-CoV-2 genomes.

The clustered heatmap in **Figure 2** turns this mutational landscape into a visual “signature” for each NICPR genome, revealing recurring patterns and rare, lineage-defining events. K182N, T259A, A344V, T478N, and N801D appear in multiple genomes, suggesting shared evolutionary routes, while G72E in NICPR402 and NICPR433 points to convergent changes in the NTD. R190S in NICPR437 links directly to the CH.1.1 lineage, tying the local dataset into global variant dynamics, and unique findings such as T574I (NICPR404) and S494P (NICPR430) add further layers to the functional plasticity of spike.

Together, the text, the allele-frequency table, and the heatmap portray a spike protein under continuous refinement, where combinations of deletions, polarity shifts, and hotspot substitutions at positions like 242-243, 478, and 521 collectively shape viral fitness, receptor engagement, and susceptibility to neutralizing antibodies.

### Heatmap of non-spike structural proteins (N, M, and E) showing distinct mutational patterns

**Figure 3:**
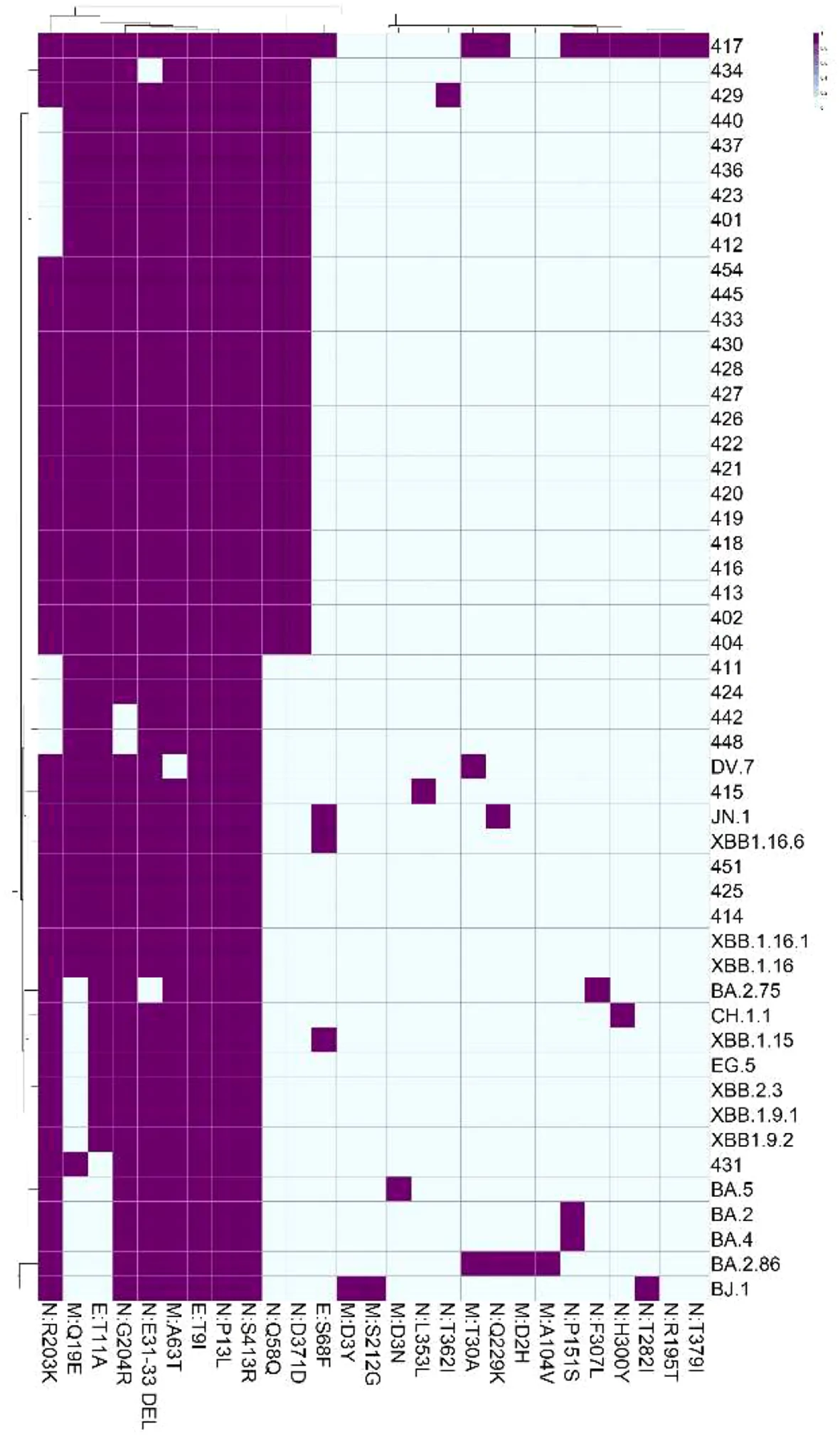
A clustered heatmap illustrating the mutations identified in the other structural proteins (Nucleocapsid, Envelope and Membrane protein) of the SARS-CoV-2 genomes.

To further dissect the genomic architecture, a heatmap of the non-spike structural proteins (N, M, and E) was plotted to show the accumulation of distinct mutational patterns across the cohort (**Figure 3**).

#### Nucleocapsid protein: lineage markers, unique changes, and synonymous variants

In the N protein, multiple substitutions act as lineage-informative markers and sample-specific signatures. N:F307L and N:H300Y map to CH.1.1- and XBB.1.15-like reference lineages, respectively, while N:P151 is shared with both BA.2 and BA.4, suggesting overlap with these Omicron sublineages. Within the cohort, NICPR417 is particularly enriched in distinctive changes: N:R195T and N:T379I occur exclusively in this sample, and it also shares N:Q229K with BA.2.86 and N:T282I with BJ.1, positioning NICPR417 at the intersection of several Omicron descendant backgrounds. Another sample, NICPR429, is defined by a unique N:T362I substitution, which does not appear in the references and therefore marks an additional independent branch within the N-protein mutational space.

Synonymous (silent) variants further refine the N-protein landscape without altering the amino acid sequence. NICPR415 carries a synonymous N:L353L change, while N:Q58Q and N:D371D are present in approximately 73% of the genomes, forming strong but functionally conservative signals in the heatmap. These synonymous markers, together with the non-synonymous sample-specific substitutions, document ongoing mutational activity in N even where protein structure and function are largely preserved.

A set of N-protein substitutions is highly conserved across both study genomes and reference lineages, reflecting well-known Omicron-era structural signatures. N:P13L, N:R203K, N:G204R, and N:S413R are widely recognized mutations associated with enhanced viral assembly and infectivity, and they are correspondingly frequent in the cohort. The N:E31-33del deletion, which removes the ERS motif in the N-terminal domain, appears in all samples and references except NICPR434 and BA.2.86, providing a nearly universal marker that ties the cohort to Omicron-associated N-protein architecture. N:G204R is detected in all references and almost all samples except NICPR442 and NICPR448, while N:R203K is present in 88% of genomes and references, consistent with their established role as a recurrent mutational pair that modulates N-protein stability and viral fitness.

#### Membrane protein: reference-only versus cohort-specific signatures

In the M protein, the heatmap clearly separates mutations that are characteristic of global reference lineages from those that define the study cohort. Several substitutions present only in reference sequences and absent from all cohort genomes underscore distinct evolutionary paths. BJ.1 carries M:D3Y and M:S212G, BA.5 carries M:D3N, and BA.2.86 carries M:D3H and M:A104V; none of these mutations are found in the study genomes, indicating that these specific M-protein profiles are not represented among the sampled viruses. The systematic variation at M position 3-D→Y in BJ.1, D→N in BA.5, and D→H in BA.2.86-creates a lineage-stratifying axis in the reference set that is absent from the cohort, reinforcing the genetic distinction between local samples and these global variants.

In contrast, the study genomes share a strong cohort-specific M-protein signal: M:Q19E is present in all collected genomes but absent from every reference sequence. In the heatmap, this appears as a solid block across all cohort rows with no corresponding signal in references, strongly suggesting that M:Q19E is a novel, cohort-defining membrane mutation. Because Q19E resides in the N-terminal luminal region of M, its fixation in the cohort may influence virion assembly or interactions with other structural proteins, although functional validation would be required. In addition, M:A63T is universally present across both samples and references, reflecting its widespread selection in Omicron-related lineages and reinforcing the shared structural backbone between the cohort and circulating variants.

#### Envelope protein: conserved backbone with cohort-enriched changes

The E protein shows a combination of globally conserved and cohort-enriched substitutions. E:T9I is universally detected across study genomes and references, consistent with its high prevalence among Omicron variants and its proposed impact on ion-channel activity and virion assembly. In contrast, E:T11A shows a more restricted distribution: it is present in almost all study samples but absent from several major reference lineages, including BA.2, BA.4, BA.5, BA.2.86, and BJ.1, as well as in sample NICPR431. This pattern implied that E:T11A is enriched within the cohort and not yet fixed across global lineages, suggesting a relatively recent or regionally constrained adaptation in the envelope protein.

### Epistatic iSNVs reshape overlapping B-cell, CD4+, and CD8+ T-cell epitopes in the SARS-CoV-2 spike protein

The impact of epistatic intra-host single nucleotide variations (iSNVs) on the immunogenic landscape of the SARS-CoV-2 spike protein was evaluated by mapping B-cell, CD4+ T-cell (HTL), and CD8+ T-cell (CTL) epitopes encompassing the key sites E180V, G184V, G252V, D253G, and P521S/T.

### CD4+ T-cell (HTL) epitopes

Within the CD4+ T-cell compartment (**Supplementary Table 3**), the wild-type S167-181E epitope was conserved in the reference sequence and several genomes (NICPR425, 411, 415, 424) and showed strong binding to multiple MHC class II alleles, including HLA-DPA10103-DPB11031 and HLA-DPA10301-DPB11031. The corresponding mutant S167-181V variant, carrying E180V and detected in NICPR413, 417, and 436, retained putative binding to these MHC-II alleles, referred that this substitution does not abolish class II recognition. The S171–185 epitope, incorporating V and G at positions 180 and 184 in samples NICPR413, 417, and 436, was predicted to bind HLA-DRB1*04:05, further supporting preserved HTL recognition in this region.

A second HTL region, S182-196 (core: FKNLREFVF), displayed substantial sequence diversity. The wild-type peptide (S182-196KQGNFKNLREFVFKN) was present in the reference genome, whereas multiple variants-S182-196KEGNFKNLREFVFKN, S182-196EEGNFKNLREFVFKN, S182-196NEGNFKNLREFVFKN, S182-196KEVNFKNLREFVFKN, and S182-196EEVNFKNLREFVFKN were identified among clinical isolates, yet all maintained binding to HLA-DRB1*12:01.* In the distal spike region, the S512-526P epitope was detected in both the reference and NICPR436 and bound to HLA-DRB101:01, suggesting that P521S/T-adjacent HTL epitopes remain MHC-II competent despite local variation.

Comparative NetMHCIIpan analysis of wild-type and mutant epitopes indicated that the identified spike substitutions exerted heterogeneous effects on HLA class II presentation. Contrary to complete epitope loss, several mutations improved predicted HLA binding affinity and antigen presentation (**Supplementary Table 12**). For example, the E180V-containing epitope VSQPFLMDLVGKEGN exhibited a marked improvement in binding to HLA-DRB1*04:05, with Rank_EL decreasing from 3.82 to 0.91 and predicted affinity improving from 475.3 nM to 51.3 nM. Similar trends were observed for G184V, G252V, and D253G containing epitopes (**Table 3**). Furthermore, the P521T-associated peptide YRVVVLSFELLHATA converted from a predicted non-binder to a weak binder. Collectively, these findings referred that the identified mutations predominantly remodel T-cell epitope presentation or forming neoantigen rather than abolishing HLA recognition.

**Table 3:** Comparative HLA class II binding analysis of wild-type and mutant SARS-CoV-2 spike epitopes harboring epistatic mutations.

Mutation	WT rank_EL	Mutant rank_EL	WT aaffinity (nM)	Mutant affinity (nM)
E180V (VSQPFLMDLEGKEGN → VSQPFLMDLVGKEGN)	3.82	0.91	475.3	51.3
G184V (multiple overlapping peptides)	4.2–11.0	1.2–4.8	158–4111	36–677
G252V	2.54	1.24	36.9	21.1
D253G	2.54	1.84	36.9	19.2

### CD8+ T-cell (CTL) epitopes

Epistatic iSNVs similarly reconfigured the CTL epitope landscape (**Supplementary Table 4**). The E180V substitution mapped to the 10-mer epitope S175–184 in NICPR425, which showed strong binding to multiple HLA-A alleles. G184V was embedded in both the 10-mer S175-184 (NICPR425) and the 11mer S182-192 (NICPR448), again with robust class I binding, and a combined E180V+G184V double-mutant S175–184 epitope was also observed in NICPR425, demonstrating tolerance of these coordinated changes within the same CTL peptide.

G252V and D253G were localized to two overlapping CTL epitopes: the 10-mer S244-252 (NICPR413, 417, 436) and the 9-mer S250-258 (NICPR413, 414, 436), suggesting that this NTD/RBD boundary region is repeatedly targeted by CD8+ T cells while undergoing coordinated amino-acid substitutions. P521T/S had the broadest CTL footprint, occurring in seven distinct 9- and 10-mer epitopes; for example, in S515–523 both the wild-type P and mutant T variants bound HLA-B*40:06. These patterns show that epistatic iSNVs at 180, 184, 252-253, and 521 modulate, but generally do not eliminate, CTL epitope presentation, instead shifting the repertoire of presented peptides.

### B-cell epitopes

B-cell epitope predictions (**Supplementary Table 5**) highlighted extensive integration of epistatic iSNVs into putative antibody-recognition sites. E180V (nucleotide POS 22101) was present in seven predicted B-cell epitopes, while G184V (POS 22113) appeared in nine, suggesting broad remodeling of N-terminal domain antigenic surfaces. Likewise, G252V and D253G (POS 22317 and 22320) were incorporated into seven B-cell epitopes, and P521T/S (POS 23123) mapped to nine distinct epitopes. This repeated embedding of iSNVs within linear B-cell epitopes implies a potential for altered antibody binding and putative humoral immune escape in these regions.

Across all three epitope classes, the patterns converge to suggest that putative epistatic iSNVs may systematically remodel, rather than uniformly abolish, immune recognition of the spike protein. E180V and G184V repeatedly occur within overlapping CD4+ HTL, CD8+ CTL, and B-cell epitopes in the N-terminal region, where they often preserve MHC binding while altering peptide sequences and predicted antibody contact sites. Similarly, G252V/D253G and P521S/T extend these coordinated changes into central and S2-proximal regions, where they are embedded in multiple CTL and B-cell epitopes, suggesting that the same nucleotide substitutions can concurrently reshape cellular and humoral targets. Together, this recurrent overlap between iSNV-harboring residues and immunodominant epitopes supports a model in which intra-host evolution fine-tunes epitope structure to balance immune escape with preservation of spike functionality and MHC presentation.

### Structure analysis: three-dimensional mapping of spike S1 SNPs and iSNVs within RBD and NTD antigenic domains

To delineate how distinct classes of sequence variation influence spike protein stability, all single nucleotide variants (SNVs) and intra-host single nucleotide variants (iSNVs) detected in the S1 subunit were mapped onto the three-dimensional spike monomer, with particular emphasis on the RBD and NTD antigenic domains.

**Figure 4:**
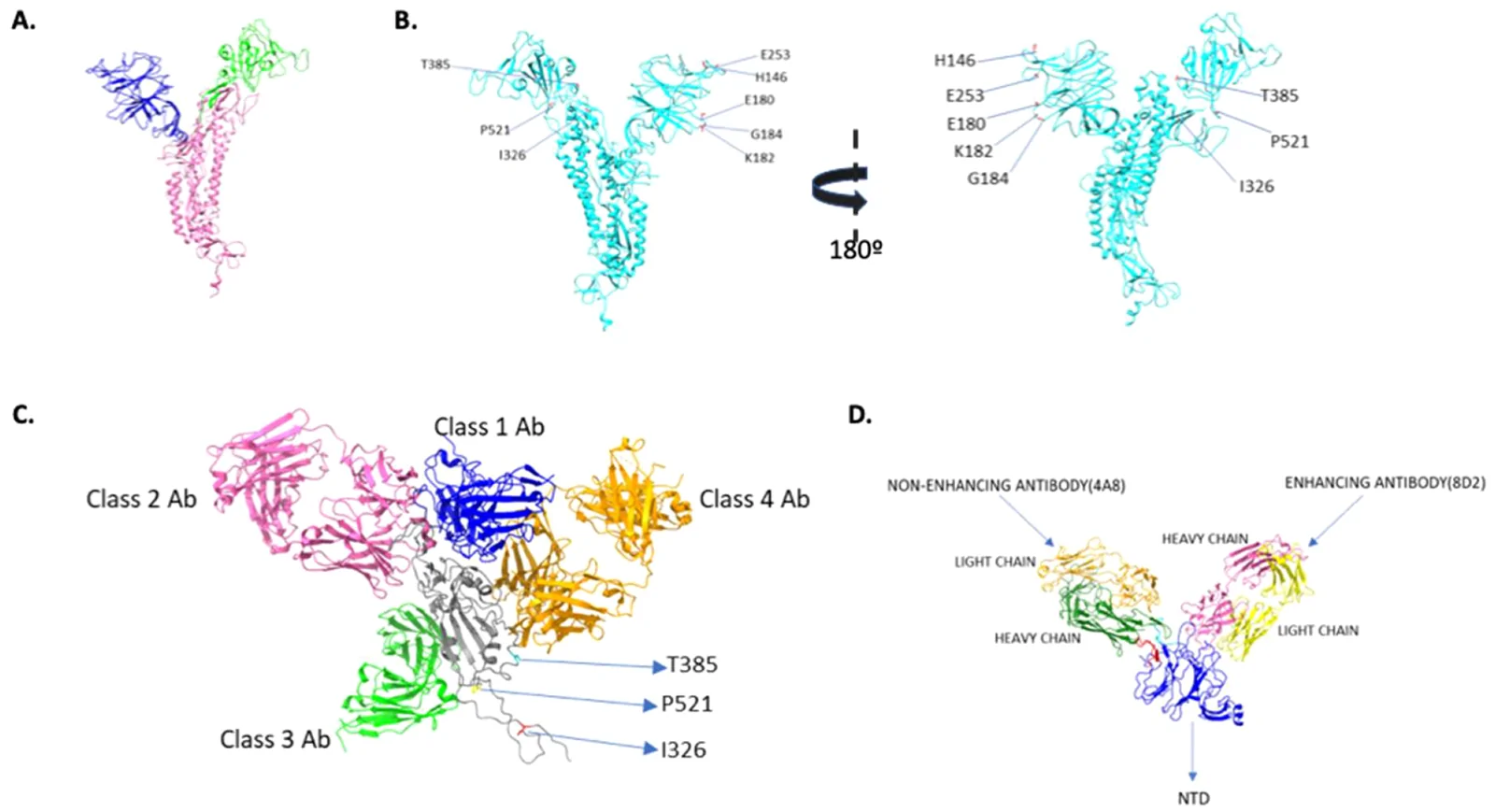
**(A)** A Spike monomer with S1 (NTD, residues 14-305, blue; RBD, residues 319-541, green) and S2 (residues 686-1273, pink) subunits. **(B)** 180°-rotated views of the monomer illustrating the spatial distribution of residues analyzed in this study. **(C)** RBD (grey) showing footprints of four antibody classes (Class 1, blue; Class 2, pink; Class 3, green; Class 4, orange) and highlighted residues I326 (red), T385 (cyan), and P521 (yellow) at the RBD base. **(D)** NTD (blue) bound by the non-enhancing antibody 4A8 (heavy chain, forest green; light chain, orange) targeting the N3 (red) and N5 (cyan) loops, and by the enhancing antibody 8D2 (heavy chain, pink; light chain, yellow).

**Figure 5:**
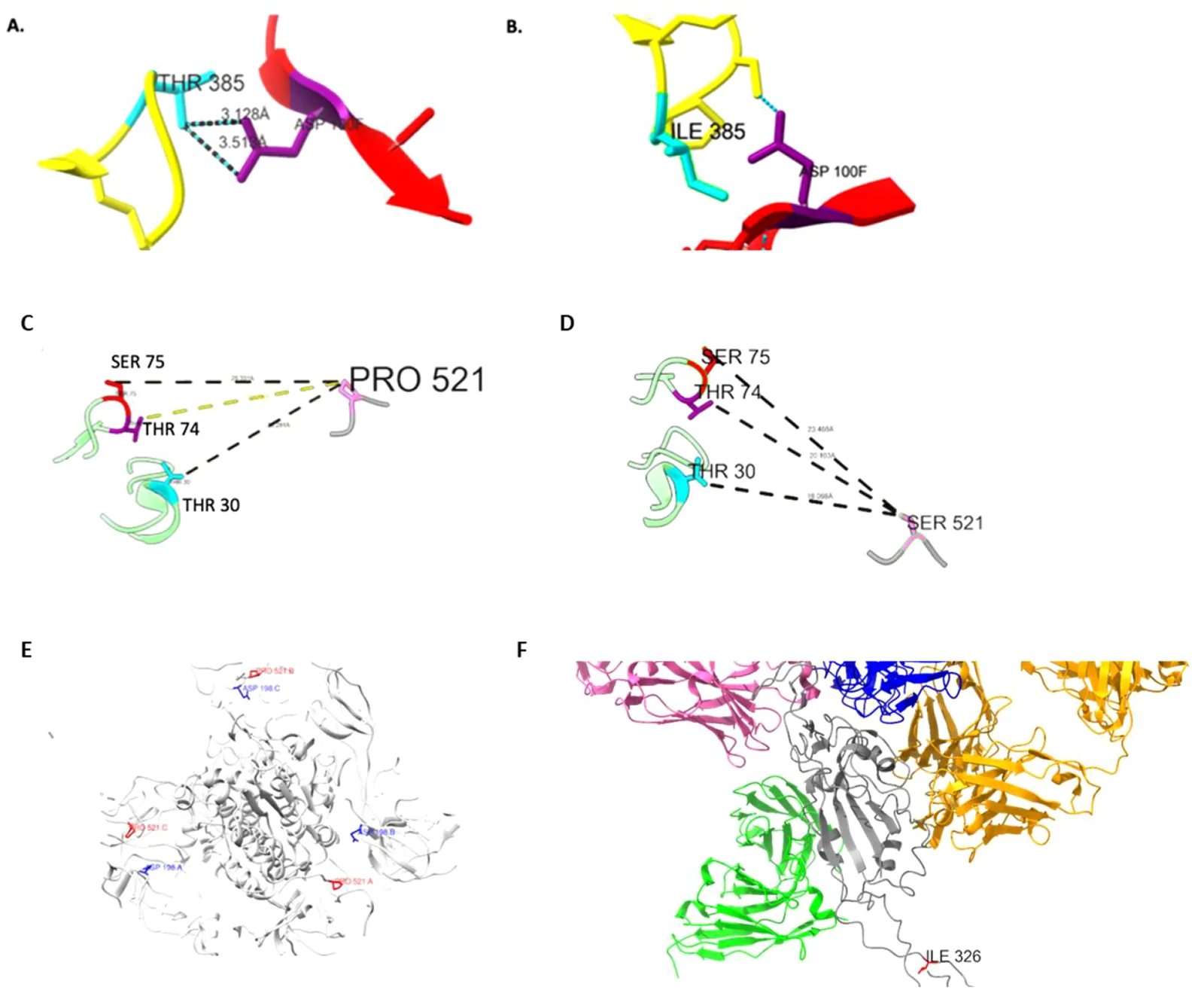
Structural impact of RBD-base mutations on local interactions and antibody interfaces. **(A)** Wild-type T385 (cyan) forms hydrogen bonds (dotted lines) with Asp100F (purple). **(B)** The T385I substitution abolishes this hydrogen-bond interaction. **(C)** Baseline spatial arrangement and distances between P521 (pink) and neighboring residues T30 (cyan), T74 (purple), and S75 (red) **(D)** Altered inter-residue distances after introduction of P521S. **(E)** Inter-protomer contact between P521 (red) on chain A and D198 (blue) on chain B forming a ring-like assembly. **(F)** Positioning of I326 (red) at the RBD base (grey) relative to surrounding antibody class footprints.

Fixed SNVs (consensus-level substitutions) were predominantly localized to structurally constrained regions at the RBD base and within the NTD antigenic supersite (**Figure 4**). In the RBD, substitutions at I326, T385, and P521 cluster at the basal scaffold that supports ACE2 engagement and binding of Class 3/4 neutralizing antibodies. *In silico* structural analyses suggest that T385I perturbs local hydrogen-bonding networks and side-chain packing at this base region, inducing modest local destabilization while maintaining the global RBD fold and ACE2-interacting surface, consistent with antigenic remodeling under structural constraint (**Figure 5**). In the NTD, fixed substitutions at H146, K182, D253 and adjacent residues map to the flexible N3 and N5 loop elements that define the major NTD supersite targeted by antibodies such as 4A8 and TXG-0078. These loop-localized SNVs are predicted to alter loop conformation and surface electrostatics without compromising the underlying β-sandwich core, thereby modulating epitope topology while preserving domain integrity.

By contrast, iSNVs frequently occur in combinations and exhibit features of stability-mediated epistasis. In the NTD, the epistatic pair E180V-G184V lies within a short helical/loop segment adjacent to the N3 region. Computational stability predictions suggest that single substitutions in this segment (for example, E180V alone) induce a moderate decrease in local stability, whereas the E180V+G184V double substitution markedly disrupts alpha-helical propensity and increases backbone flexibility, approaching the threshold for local unfolding (**Table 4**). This pattern is compatible with folding-mediated epistasis, in which structurally tolerated single iSNVs become deleterious when combined, thereby restricting the set of viable mutational trajectories. A similar principle is observed at the NTD-RBD junction, where G252V and D253G occupy a hinge-like region that couples NTD motion to RBD positioning: G252V is predicted to rigidify this hinge, whereas D253G increases local flexibility; their co-occurrence likely fine-tunes inter-domain dynamics without globally destabilizing the trimer. At the lower RBD, P521S/T affects a peripheral loop in which replacement of proline with serine or threonine relaxes a local kink and modestly increases flexibility, again consistent with “edge” mutations that reshape epitope geometry at limited structural cost.

In aggregate, this SNV/iSNV-based categorization may refer that spike evolution in S1 proceeds via a layered strategy: fixed SNVs establish relatively stable antigenic configurations at the RBD base and NTD supersite under stringent structural constraints, while iSNVs explore additional sequence space around these sites. Only those iSNV combinations that maintain acceptable local stability and preserve the global fold are retained, whereas structurally destabilizing constellations are pruned from the population.

### Impact of RBD base SNPs on antibody accessibility and local structural interactions

To extend the structural analysis from the NTD antigenic supersite to the RBD base, representative SNPs arising in the XBB.1.16-like background were examined at positions that integrate antibody binding and local stability. T385I, P521S/T, and I326T were selected because they cluster at the RBD base and thus provide complementary readouts on Class 3/Class 4 antibody epitopes and intradomain packing.

In the C118-RBD complex (Class 4 epitope; PDB 7RKS), T385 forms a hydrogen bond with heavy-chain residue D100, so T385I directly probes an XBB.1.16-associated alteration within a conserved cryptic epitope. The attached structural view illustrates the spatial relationship of P521 (mutated to S521) with Class 3 antibody residues T30, T74, and S75: although these residues are predicted to be solvent-exposed and proximal, distance mapping no direct contacts between S521 and the antibody, implying that the P521S substitution minimally perturbs the Class 3 interface and is more likely to affect inter-protomer packing in the trimer. I326T, located at the RBD base but outside defined antibody footprints, is predicted to interact with N540 within the RBD, serving as an internal comparator for how XBB.1.16-linked substitutions tune intradomain packing and local structural integrity without directly reshaping mapped epitope surfaces.

Solvent-accessibility analysis further supports the functional distinction between the three RBD residues. I326 shows a near-zero SASA (1.18 Å²; 0.3%), predicting that it is buried within the RBD core and more likely to influence internal packing than direct antibody binding, in line with its proposed role in contacting N540 (**Table 5**). T385, by contrast, is highly solvent-exposed (108.01 Å²; 85.2%), consistent with its direct engagement in the Class 4 cryptic epitope and hydrogen bonding to the C118 heavy chain. P521 is also fully exposed (100% relative SASA), placing it on the RBD surface within the Class 3 footprint; however, the neighboring Class 3 antibody residues T30 and S75, although solvent-accessible (22.4% and 82.1% respectively), do not form direct contacts with P521/S521, reinforcing the conclusion from the structural analysis that the P521S substitution has minimal impact on the antibody interface and instead is more likely to modulate interprotomer interactions.

**Table 4:** Predicted effects of XBB-associated spike substitutions on conformational stability (ΔΔG, DynaMut).

Mutation	Wild type	Mutant	ΔΔG DynaMut kcal/mol
E180V	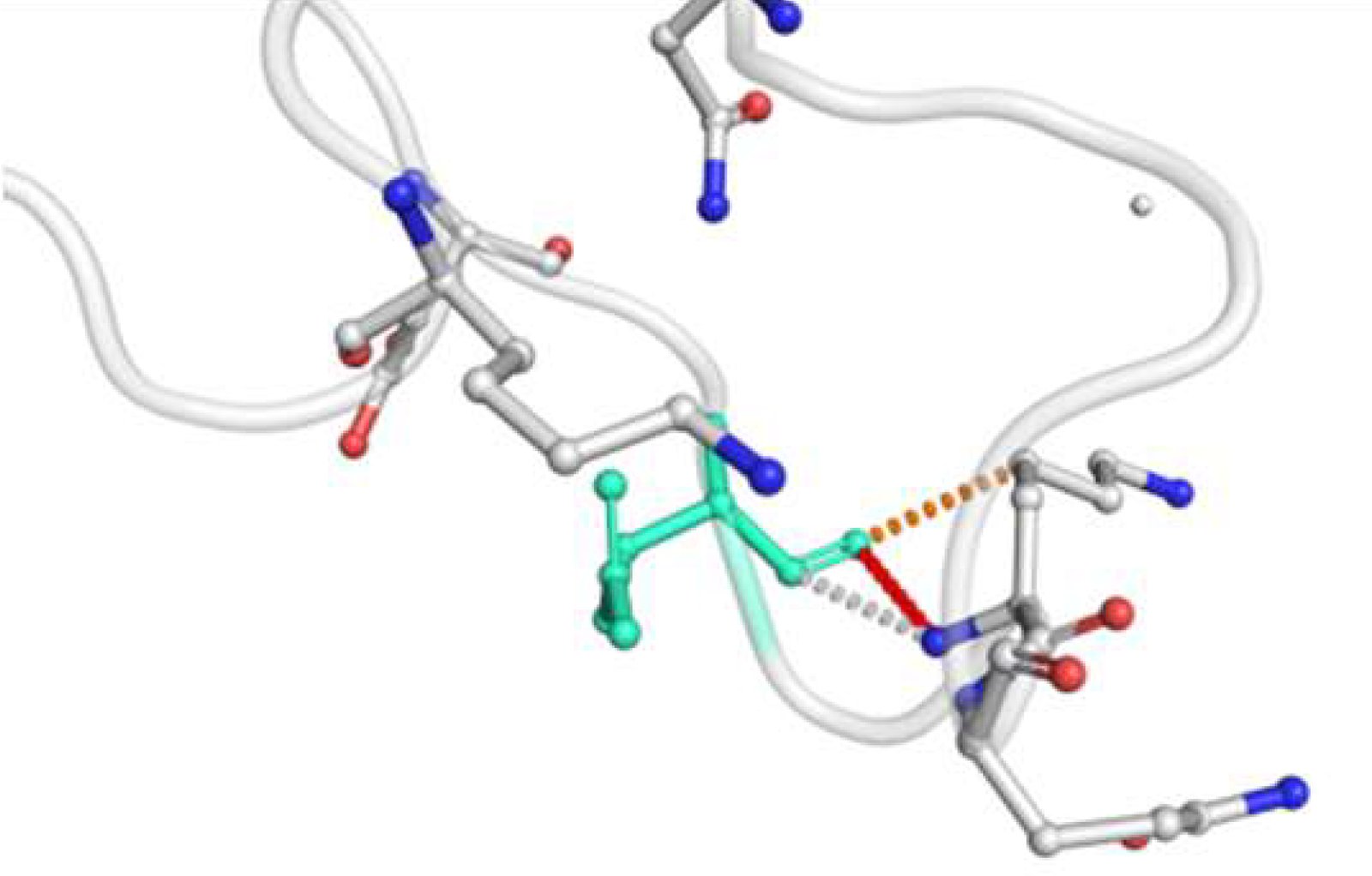 E180	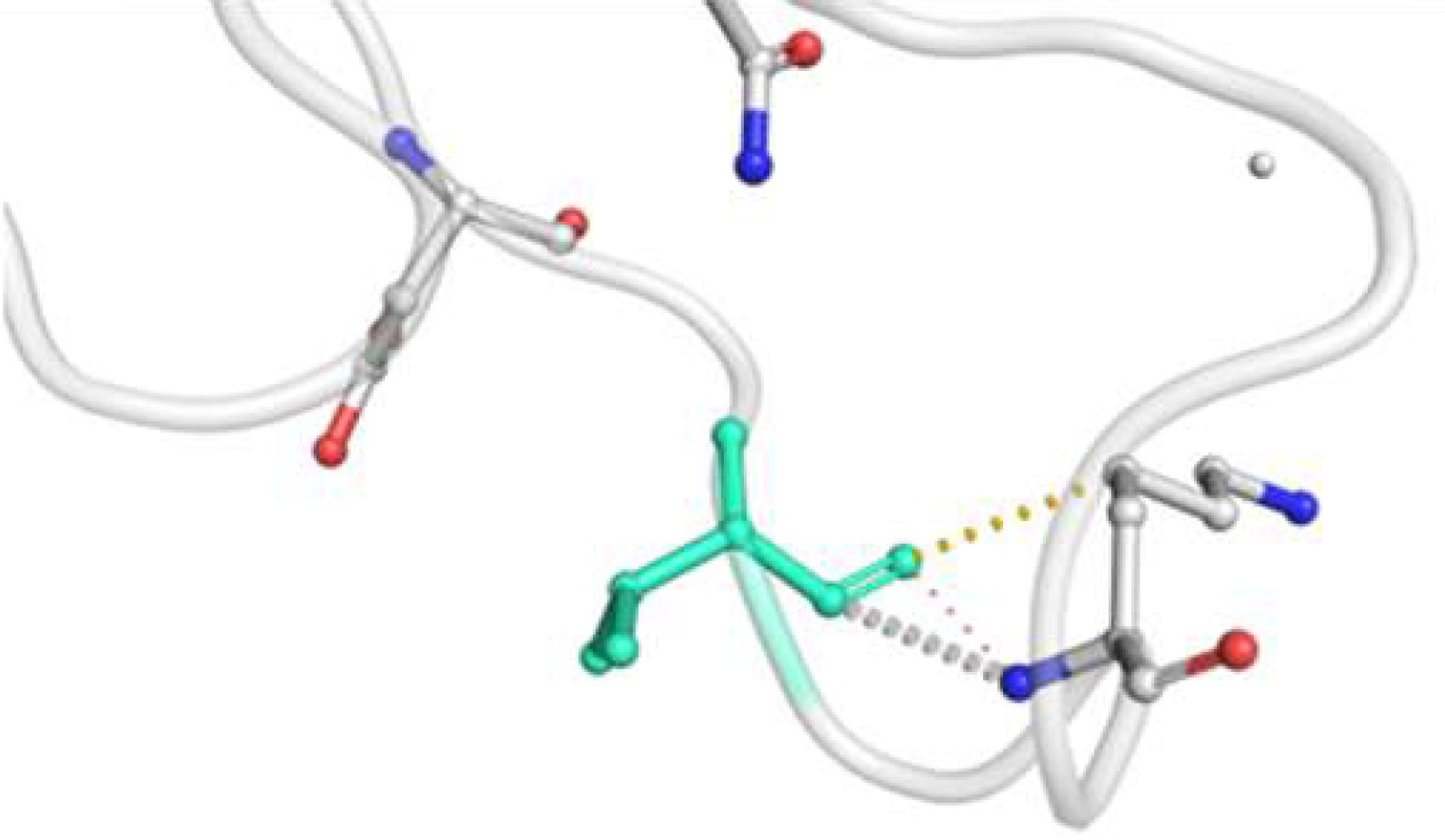 V180	-0.624
G184V	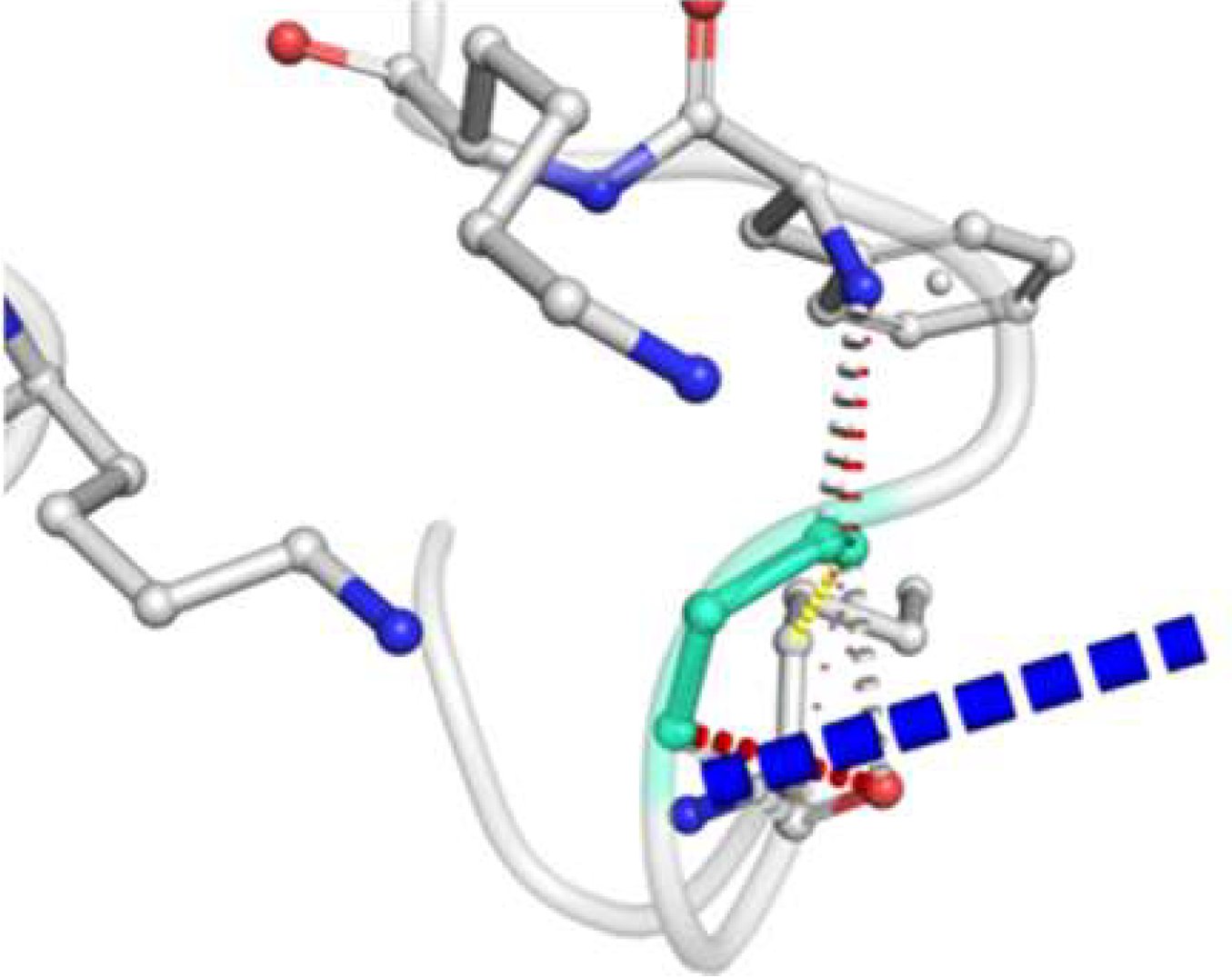 G184	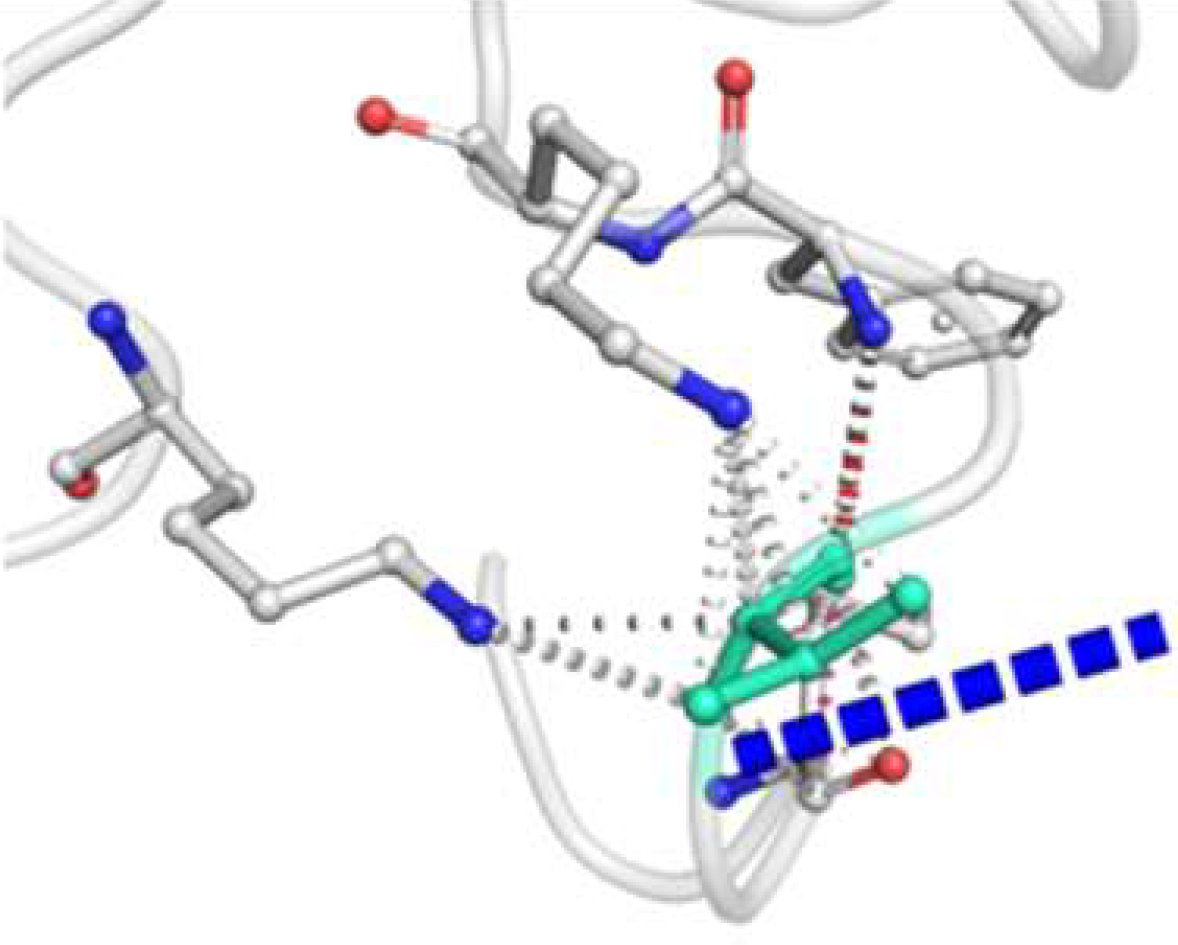 V184	-0.992
G252V	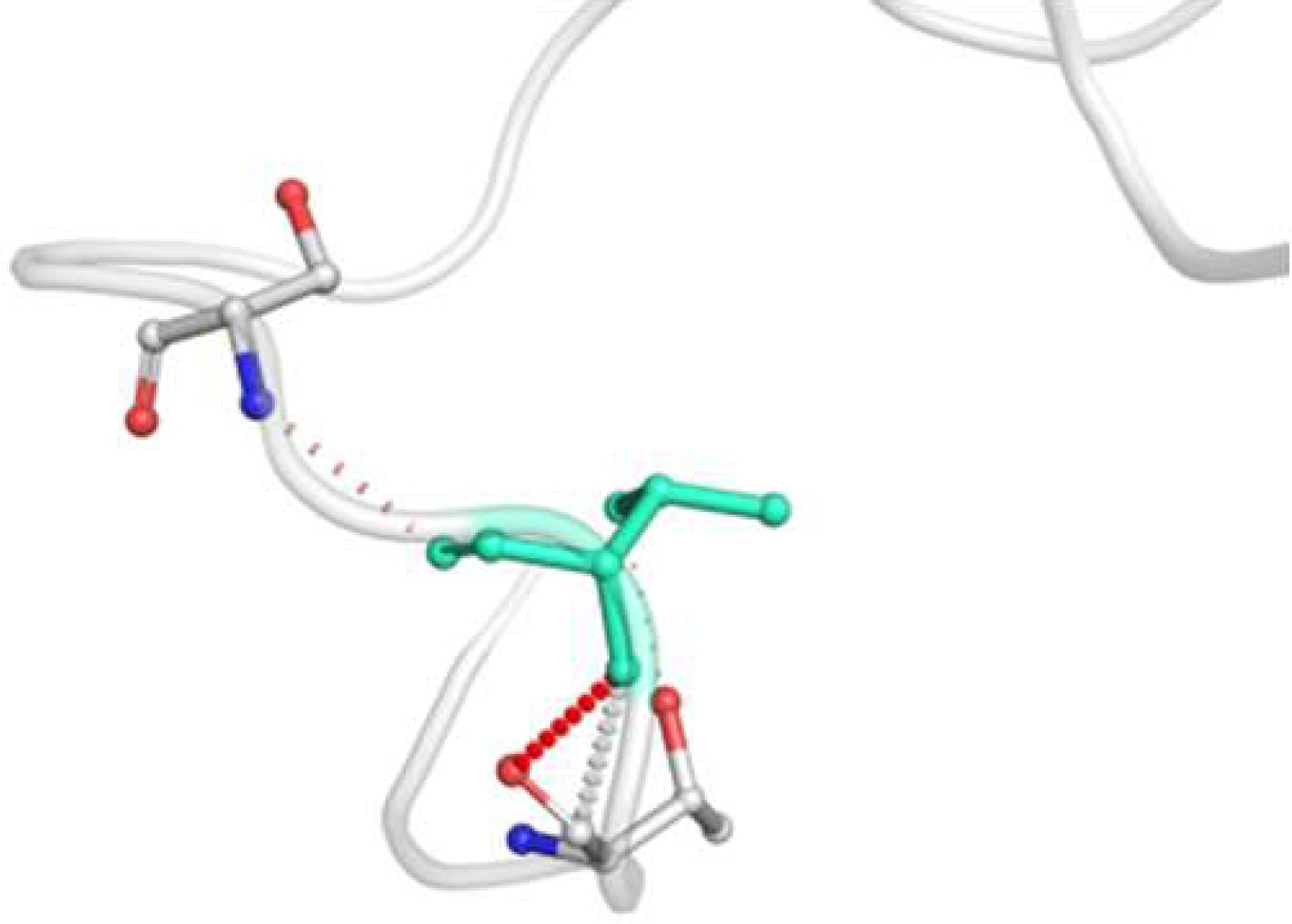 G252	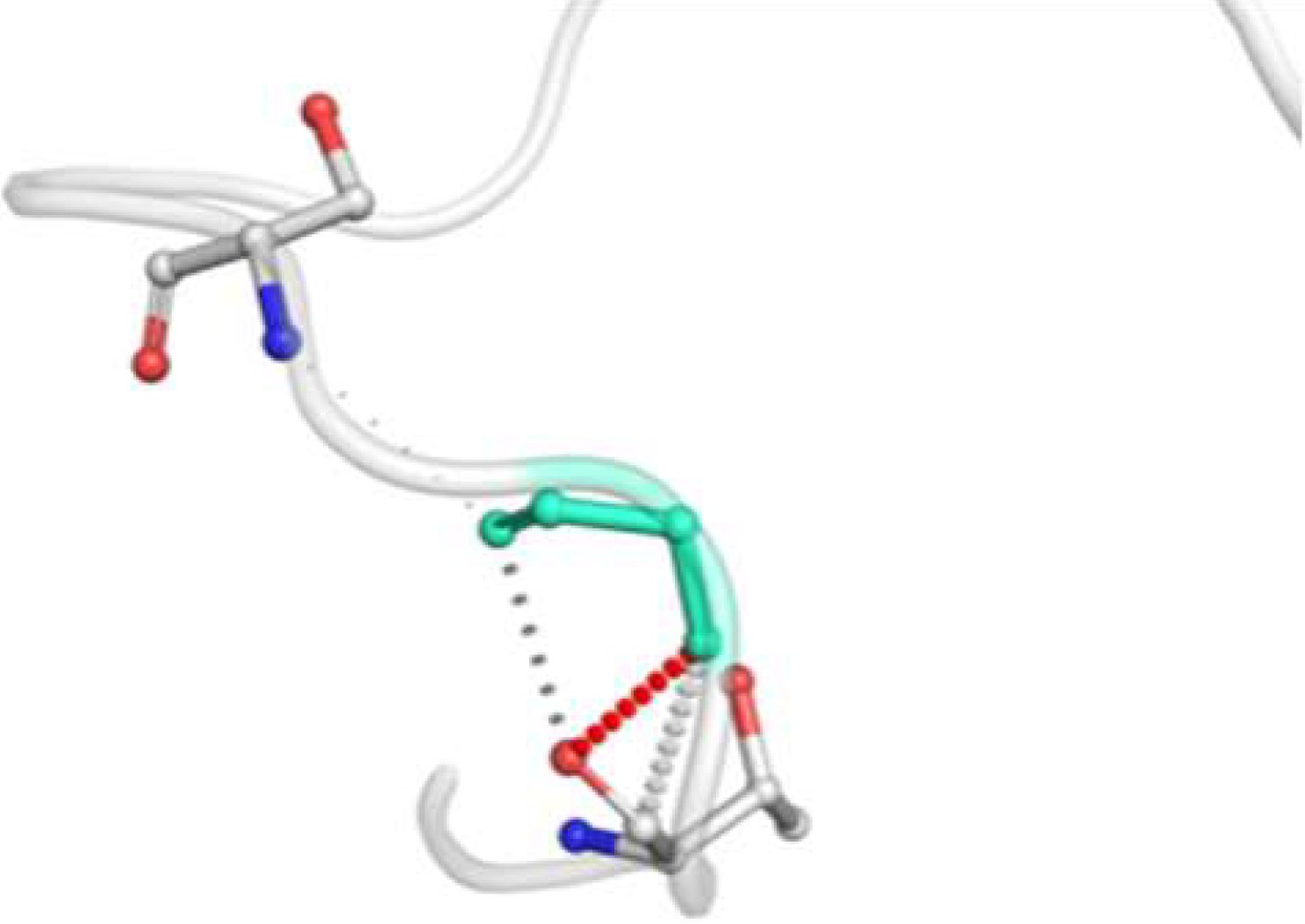 V252	0.284
D253G	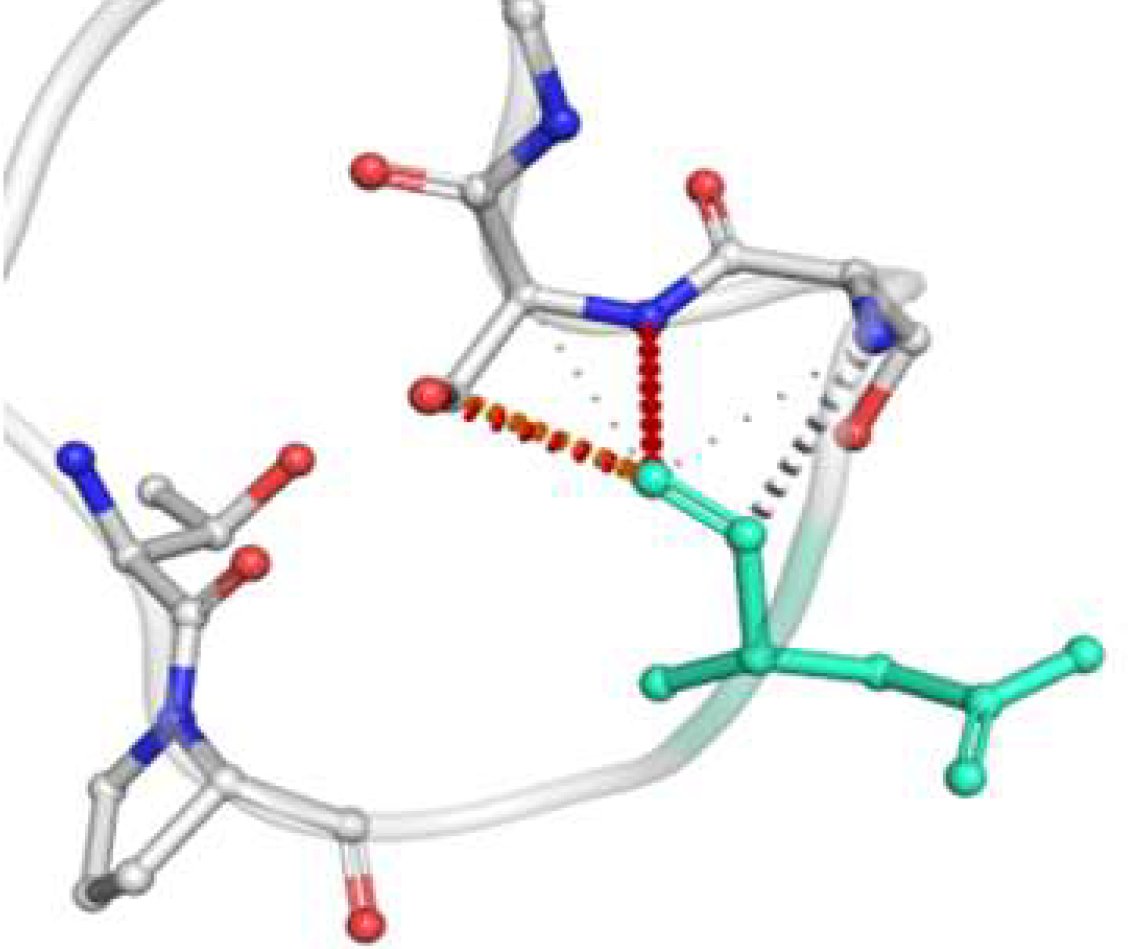 D253	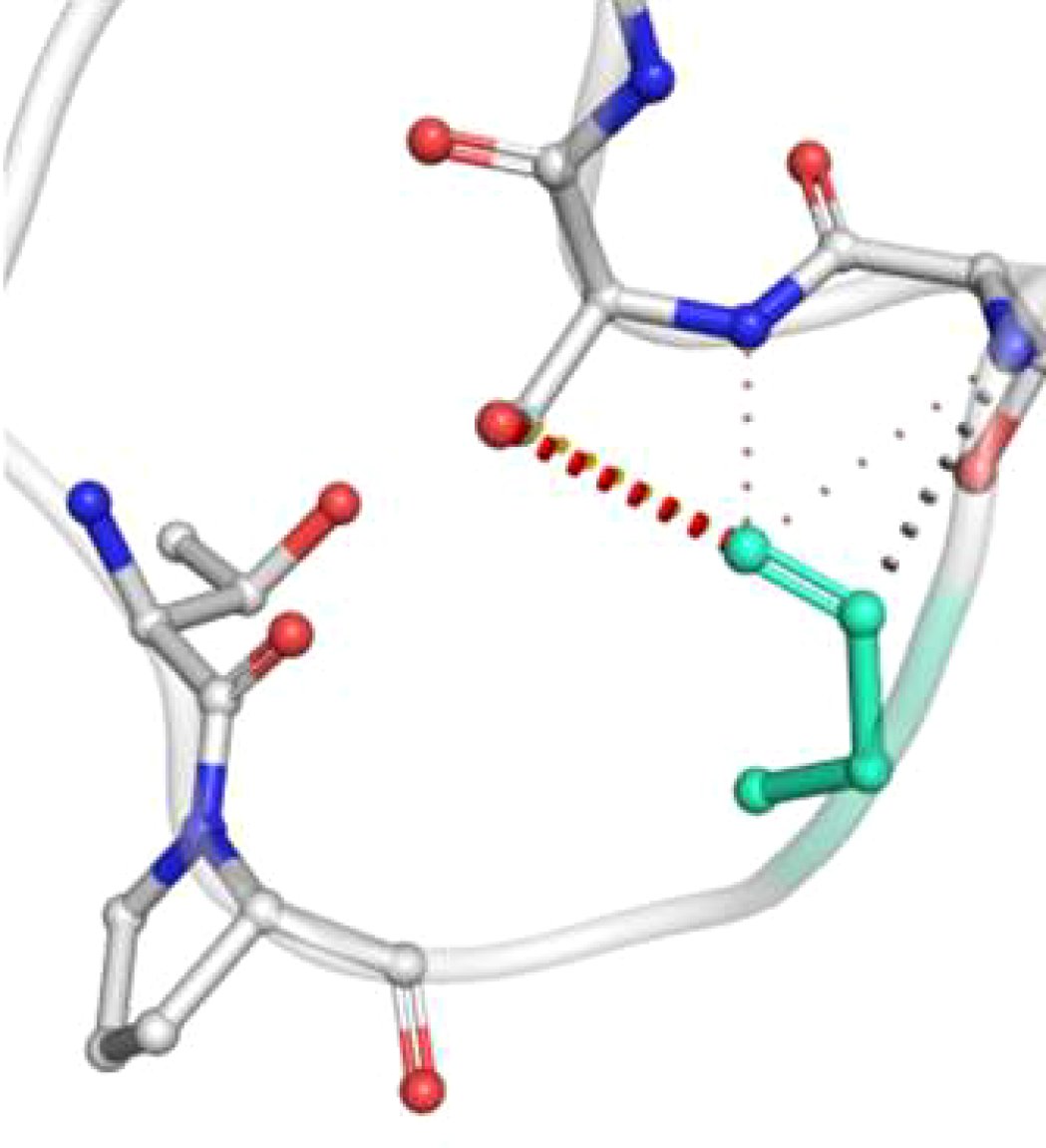 G253	-0.580
P521S	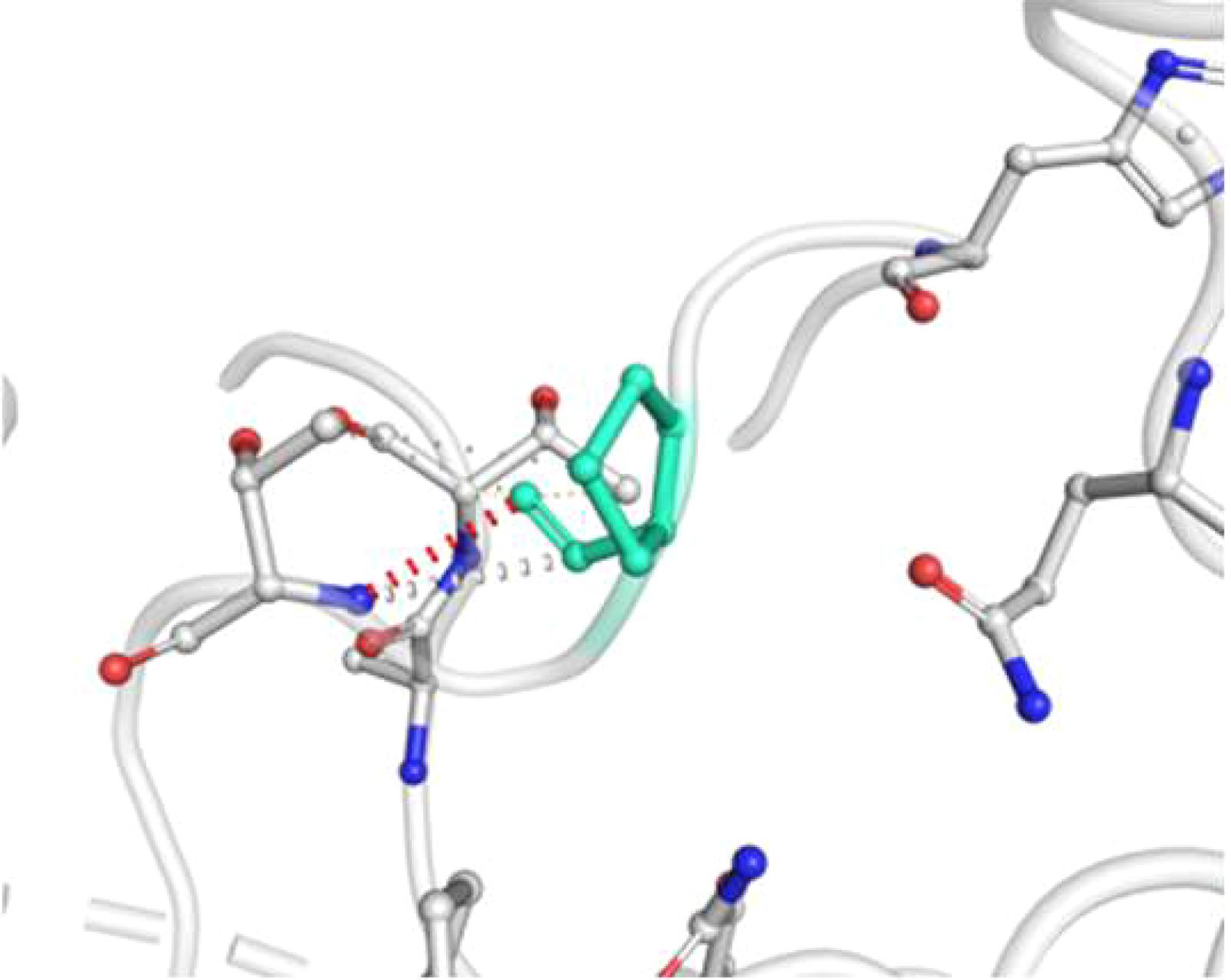 P521	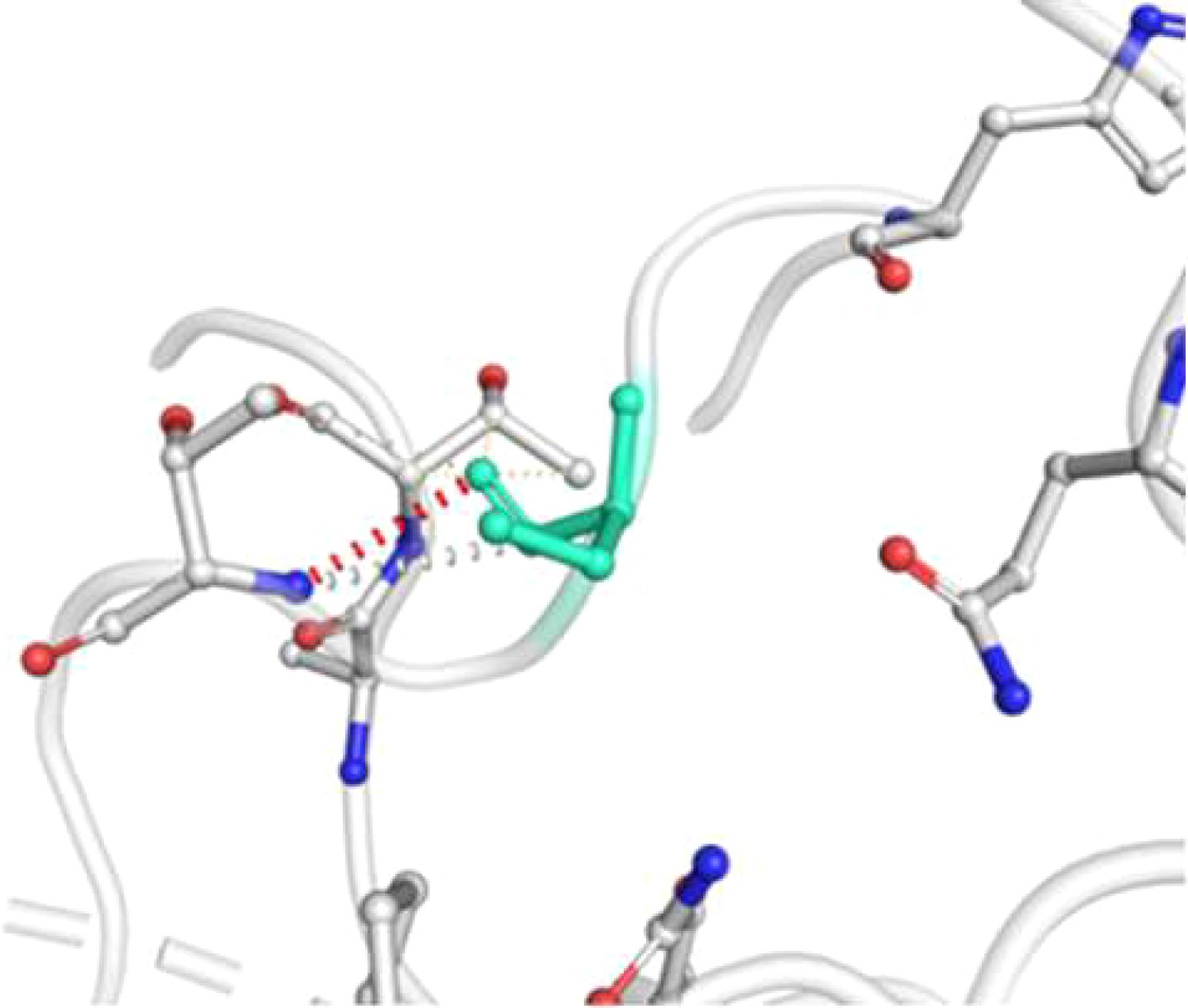 S521	0.313

### Mutation analysis of NTD residues

#### NTD supersite remodeling: differential effects of XBB-linked mutations on neutralizing (4A8/TXG-0078) and enhancing (8D2) antibody interfaces

**Figure 6:**
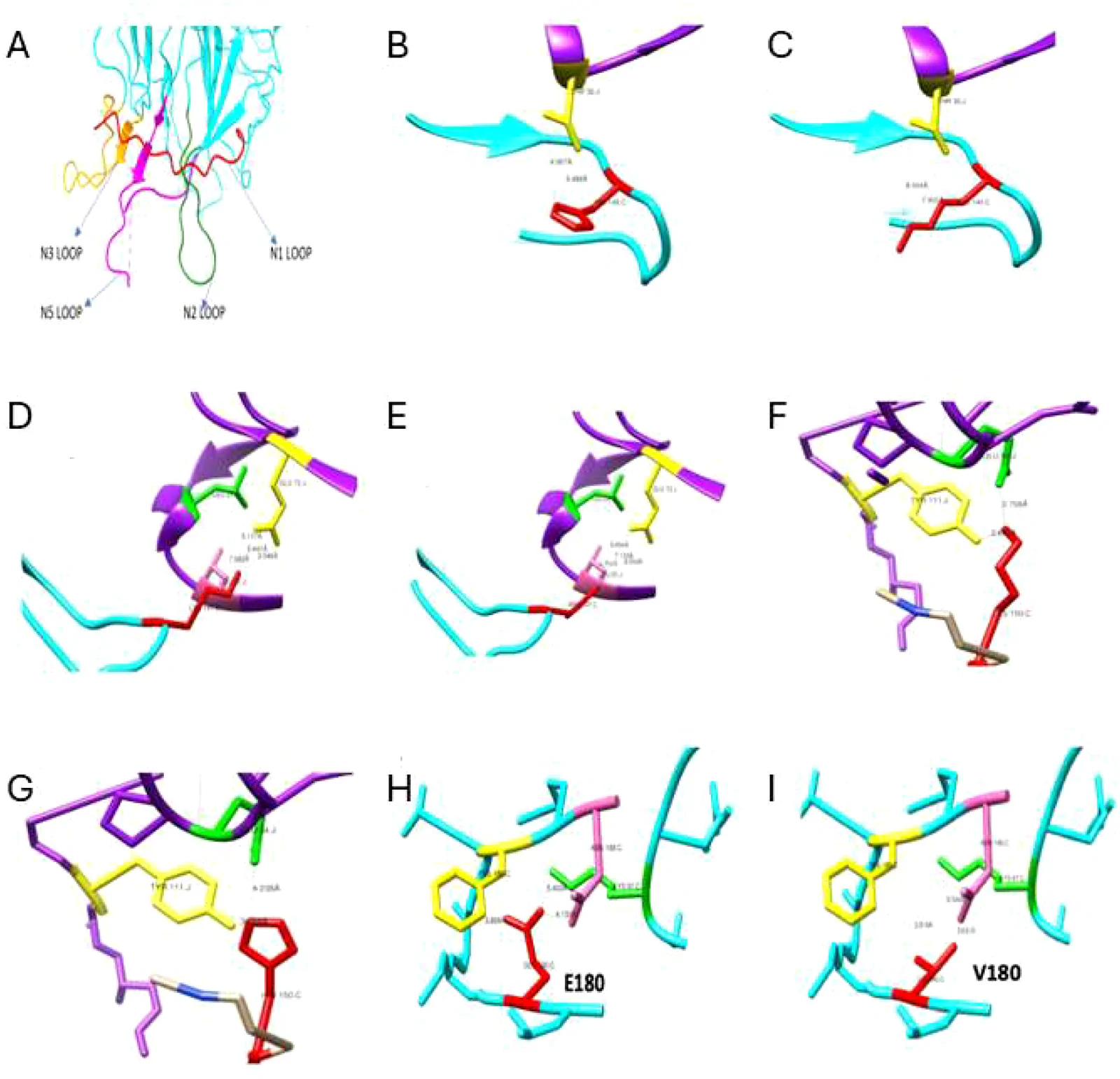
**(A)** Overview of the NTD (cyan) visualized using UCSF ChimeraX, highlighting key functional loop regions: N1 (red), N2 (forest green), N3 (orange), and N5 (magenta). Impact on interaction of spike’s residue mutation with antibody 4A8. **(B)** Residue H146 on the NTD (Chain C) forms a 2.627 Å hydrogen bond (H- bond) with T30 on the 4A8 heavy chain (Chain J). **(C)** Structural impact H146K mutation at the NTD-4A8 interface. **(D)** K147 (red) forms four H-bonds: L29 (green; 3.405 Å), L32 (pink; 2.749 Å), and two bonds with D72 (yellow; 3.442 Å and 3.346 Å). **(E)** Disruption of K147H mutation on the local H-bonding network. **(F)** K150 forms H bonds E54 (2.757 Å) and Y111 (2.881 Å). **(G)** Weakening of interaction due to K150H mutation **(H)** E180 (red) exhibits a distance of 4.132 Å to the adjacent NTD residue N188. **(I)** Alteration in local hydrophilic and hydrophobic intradomain interactions due to E180V mutation.

NTD interaction occurs through four loops (**Figure 6**). 4A8, 8D2, and TXG-0078 were selected as reference antibodies because they collectively represent neutralizing, enhancing, and broadly neutralizing NTD-directed responses at the same antigenic supersite, providing a functional framework for interpreting XBB-associated NTD mutations.

Using 4A8 as the primary structural probe (PDB 7C2L), the impact of NTD substitutions on a well-defined neutralizing supersite was quantified. It interacts with heavy chain and light chain mostly with the N3 loop (141-156) and N5 loop (246-260) (**Figure 6**). H146K and K150H increased the hydrogen-bond distances to heavy-chain residues T30 and E54/Y111, respectively, while K147R abolished electrostatic and hydrophobic contacts with E72, L32, and L29, suggesting a net weakening and redistribution of 4A8 interactions across the N3/N5 loop interface. In contrast, R246K introduced a potentially stronger ionic interaction with E31, suggesting a compensatory strengthening at the N5 loop. Residues E180, K182, and G184 were located outside the 4A8 footprint and exhibited low solvent exposure; SASA and intradomain contact analysis predicted that E180V primarily perturbs an internal NTD contact with N188, consistent with a structural-stability rather than direct epitope effect. Interface residues were defined by SASA reduction upon complex formation, and changes in buried surface area and contact networks between wild-type and mutant complexes supported a mutation-driven modulation, rather than complete loss, of the 4A8-NTD neutralizing interface.

In the 8D2-NTD complex (PDB 7DZX), the XBB-linked residues E180 and G184 are positioned adjacent to the 8D2 footprint, but their roles differ from those observed for 4A8. G184 is solvent-exposed at the periphery of the enhancing epitope, whereas E180 and H146 are largely buried and make minimal direct contact with 8D2, implying that these positions primarily support local NTD architecture rather than constituting core enhancing contacts, in contrast to their stronger contribution to the 4A8/TXG-0078 neutralizing supersite.

For TXG-0078, which binds the same NTD supersite as 4A8 but with broader tolerance, the effects are mixed. H146 is both an intradomain stabilizer (via N149) and an interface residue; H146K reconfigures this region by forming a new contact with S151, representing a gain of an alternative stabilizing interaction rather than a simple loss. In contrast, D253 is a genuine interface hot spot: it forms a hydrogen bond with Q1 of the TXG-0078 heavy chain and shows substantial accessible and buried surface area. The D253G mutation abolishes this hydrogen bond and is predicted to weaken TXG-0078 binding, representing a clear loss of interaction at this supersite. E180, despite being solvent-accessible, lies outside the TXG-0078 contact zone; its mutation is therefore not expected to significantly alter TXG-0078 binding, consistent with its limited role in 4A8 and 8D2 interfaces.

Overall, these data suggest that XBB-associated NTD mutations preferentially erode specific neutralizing contacts (4A8, TXG-0078) while leaving enhancing (8D2) interactions largely intact and, in some cases (R246K, H146K), introducing alternative stabilizing or compensatory contacts, consistent with immune escape under preserved structural fitness.

### Structure-function analysis of mutations on spike protein

**Figure 7:**
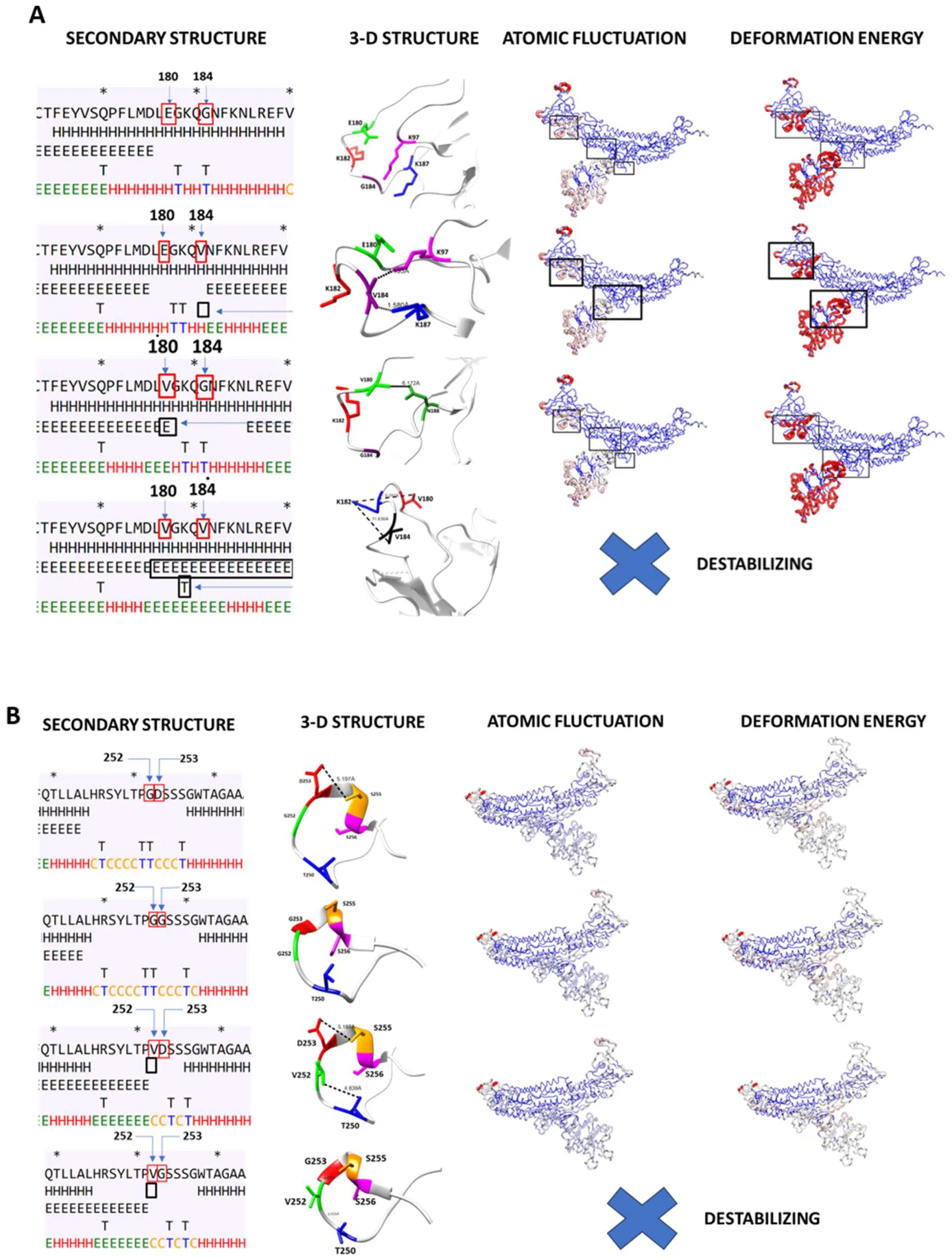
Structural effects of putative epistatic mutations in the SARS-CoV-2 spike N-terminal domain. **(A)** E180V/G184V and **(B)** G252V/D253G mutational regions. Secondary structures were predicted using CFSSP, and corresponding 3-D models were generated in UCSF ChimeraX. Residue-level atomic fluctuations and deformation energies were calculated with DynaMut and visualized as tubes, where color and thickness indicate residue flexibility (blue, low; white, intermediate; red, high). Black boxes highlight regions showing the greatest structural changes. Both mutation combinations exhibit increased local flexibility and are predicted to exert destabilizing effects on the NTD structure.

The molecular dynamics analysis suggested that XBB-linked substitutions at positions E180V and G184V as well as G252V & D253G induce localized adjustments in NTD loop architecture without disrupting the global fold. Across variants, secondary-structure predictions show only minor conversions between turn and coil elements around these residues 180 &184, and G252V & D253G while the overall β-sheet/helix pattern of the NTD is preserved, consistent with maintenance of the structural scaffold required for NTD-directed antibody binding. Atomic fluctuation and deformation-energy profiles showed a spatially confined redistribution of flexibility and strain within the supersite region, consistent with fine-tuning rather than destabilization of this epitope and providing a structural basis for the partial remodeling of the 4A8/TXG-0078 neutralizing interface while maintaining the enhancing epitope for 8D2. Simultaneous E180V/G184V substitution was predicted to fully disrupt a local α-helical element yet yielded a negative ΔΔG in DynaMut, indicating a net stabilizing effect on the folded state (**Figure 7A**), whereas single mutations G252V and D253G were predicted to increase and decrease stability, respectively, in line with their distinct local contacts (G252-T250 and D253-P521). Combined G252V/D253G mutation is therefore expected to balance these opposing effects, producing local but not global destabilization (**Figure 7B**), and P521S in the S2 region was likewise predicted to further stabilize spike structure, suggesting that XBB-associated substitutions collectively modulate local NTD dynamics and overall spike stability in a manner compatible with preserved structural fitness and altered antibody recognition.

**Table 5:** Solvent accessibility of RBD base residues (I326, T385, P521) and class 3 antibody contact residue.

Spike Domain	Residue	SASA value		Buried/exposed
		ePISA(Å²)	Getarea(%)	
RBD	I326	1.18	0.3%	Buried
	T385	108.01	85.2%	Exposed
	P521	**-**	100.0%	Exposed
NTD	H146	70.76	28.4%	**-**
	K147	155.87	3.3%	Buried
	K150	154.56	42.8%	**-**
	R246	84.69	6.2%	Buried
	E180	32.66	23.1%	**-**
	K182	100.87	59.4%	Exposed
	G184	13.02	17.7%	Buried
	H146	**-**	0.0%	Buried
	E180	**-**	0.0%	Buried
	G184	**-**	71.1%	Exposed
	H146	52.32	21.7%	**-**
	D253	147.39	54.9%	Exposed
	E180	128.02	73.0%	Exposed

### Trajectory analysis and local RMSD computation

**Figure 8:**
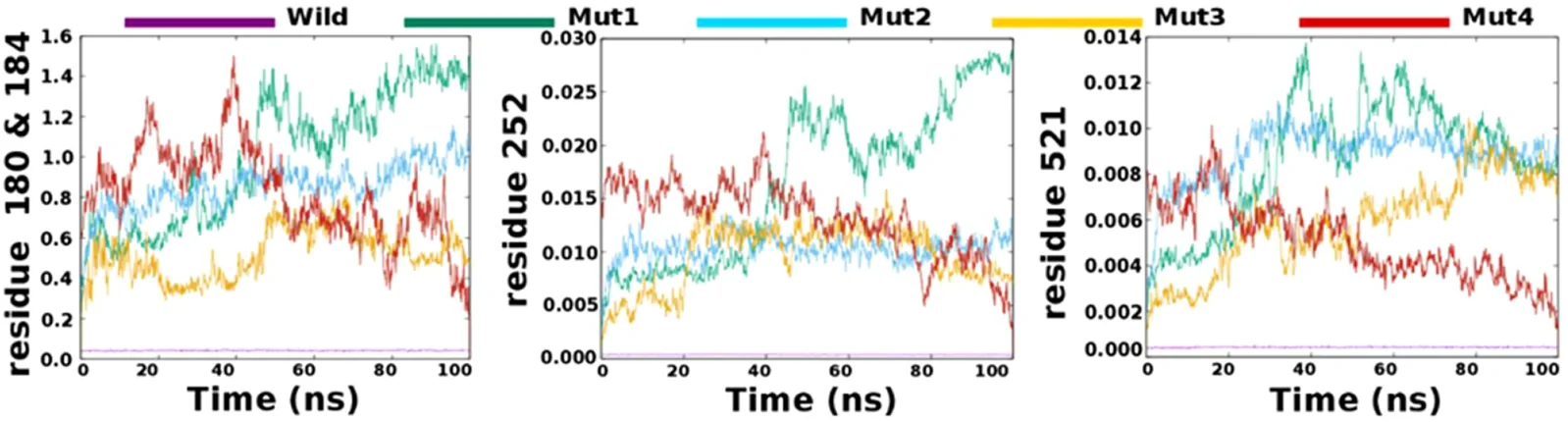
Local backbone RMSD trajectories for wild-type and mutant proteins.

Backbone RMSD (root mean square deviation) was computed over 10-residue windows centered on iSNV sites (residues 176–186 for E180V/G184V, 247–256 for G252V, 516–525 for P521S) at 100 ps intervals across 100 ns trajectories, following PBC correction (gmx trjconv), centering, and Cα least-squares fitting to energy-minimized structures (GROMACS 2023.1). The four regional residue windows were: a combined residue-window of 176–186 for E180V and G184V, residue window of 247–256 for G252V, and residue-window of 516–525 for P521S (**Figure 8**). RMSD was computed at 100 ps intervals over the full 100 ns trajectory. Wild-type maintains stable RMSD profiles while mutants show elevated fluctuations, this may be due to local structural instability induced by mutations. Thus, this elevated RMSD fluctuations signify mutation-induced conformational perturbations, disrupting local stability and potentially impairing NTD function in antibody binding or viral processes consistent with reduced structural rigidity in disease-related variants. This local-window analysis isolates these effects from global dynamics, aligning with biophysical frameworks for assessing iSNV impacts.

## Discussion

The longitudinal genomic analysis of spike sequences in this cohort suggests a dynamic intra-host evolutionary process in which early XBB backgrounds progressively acquire hallmark mutations of more derived sublineages while retaining a stable Omicron BA.2 core. Near-fixation of conserved substitutions such as D405N and R408S is consistent with structural studies of BA.2 spikes showing that these changes lie outside the ACE2 contact surface and preserve the overall Omicron scaffold while peripheral mutations tune receptor binding and antigenicity (6, 31). Superimposed on this stable backbone, we observe rapid shifts in sub-lineage-defining markers, including XBB.1.16 and XBB.2.3 associated substitutions, within individual hosts. The expansion of P521S from a minor intra-host variant to near-fixation in Patient 3 is consistent with strong positive selection for specific spike constellations, similar to the selective enrichment of stabilizing and receptor enhancing mutations reported in XBB.1.5 and XBB.1.16 (10, 32). These patterns support the view that the XBB fitness landscape is epistatically structured, with only certain combinations of mutations simultaneously compatible with structural integrity and immune escape (2, 5).

A striking feature of our data is the apparent mutual exclusivity between XBB.2.3like (P521S, D253G, G184V) and XBB.1.16 like (E180V, G252V, P521T) constellations, suggesting negative epistasis or strong functional trade-offs between these marker sets. Deep mutational scanning and structural modelling of Omicron and XBB spikes have similarly shown that, although individual substitutions can be tolerated or even beneficial, particular combinations destabilize local secondary structure or disrupt intramolecular interaction networks, thereby constraining accessible evolutionary pathways (6, 33). Our stability predictions are concordant with this framework: E180V alone induces only modest local destabilization, whereas its combination with G184V substantially reduces α-helical propensity and approaches thresholds associated with local unfolding. Such folding E180V alone induces only modest local mediated epistasis where a structurally acceptable single mutation becomes deleterious in combination has been documented in RNA viruses generally and specifically at the SARS-CoV-2 RBD interface, where certain double mutants show pronounced losses in expression or ACE2 binding despite individually neutral effects (34, 35). This folding-mediated epistatic mechanism likely plays a similar role in sculpting the accessible mutational space of the XBB spike.

Structural mapping further predicted that fixed SNVs tend to define stable antigenic configurations at the RBD base and NTD supersite, while intra-host iSNVs cluster around these regions and probe adjacent sequence and conformational space. In the NTD, E180V and G184V lie near the N3 loop, a key component of the antigenic supersite where Omicron-lineage mutations and insertions have been shown to markedly alter neutralizing epitope geometry while preserving the global NTD fold (11, 36, 37). At the NTD-RBD junction, G252V and D253G appear to exert opposing effects on local flexibility, analogous to hinge-proximal substitutions in Omicron spikes that shift interdomain dynamics and RBD up/down equilibria without destabilizing the trimeric assembly (38, 39). Position 478 exemplifies a recurrent functional hotspot: substitutions such as T478K/R/Q/N modulate surface charge and H-bonding at the RBD-ACE2 interface and may be linked to predicted enhanced ACE2 affinity and putative immuneescape in Omicron sublineages (40, 41).

These structurally constrained mutation patterns translate into targeted remodeling of the spike immunogenic landscape. Comprehensive epitope analyses of Omicron and XBB spikes have shown preferential accumulation of changes within NTD and RBD antigenic supersites that diminish binding by key classes of neutralizing antibodies while maintaining receptor engagement and fusogenic function (6, 42). In agreement, we find that E180V, G184V, G252V, D253G, and P521S/T overlap or lie adjacent to predicted B-cell, CD4^+^, and CD8^+^ T-cell epitopes and intersect known footprints of NTD-directed antibodies such as 4A8 and TXG-0078, as well as class 3 RBD-directed antibodies (11, 12). Structural analysis suggests that D253G perturbs a key H-bonding hotspot in the TXG-0078 interface, consistent with reduced neutralization observed for analogous NTD loop alterations in Omicron variants (12), whereas P521S/T at the RBD base produces minimal direct disruption of antibody contacts and instead likely modulates interprotomer packing and epitope presentation (32).

The integrated analysis of B cell and T cell epitope analysis suggests a dual immunological consequence of the epistatic mutations enhanced HLA class II presentation with reduced B-cell epitope accessibility. This pattern suggests antigenic remodeling rather than complete immune escape, because the substitutions appear to preserve or improve CD4+ T-cell recognition while weakening antibody-targeted interactions. Such uncoupling of B-cell and T-cell effects is consistent with reports showing that SARS-CoV-2 variant mutations can alter peptide-HLA-II presentation through register shifts or TCR-facing changes, while broad CD4+ T-cell immunity remains relatively resilient. In parallel, studies of SARS-CoV-2 evolution have shown that spike mutations can reduce antibody recognition while maintaining functional constraints on T-cell epitopes, supporting selective immune adaptation under humoral pressure (43, 44).

Overall, these computational findings suggest a potential immune escape strategy in which the virus may remodel B-cell epitopes but retain or enhance predicted CD4+ T-cell presentation. Such a mechanism would allow partial evasion of neutralizing antibody pressure while preserving helper T-cell immunogenicity, which may help maintain viral fitness and antigenic flexibility.

Taken together, these observations support a model in which intra-host evolution within XBB lineages may not be driven by random accumulation of changes, but by epistatically constrained combinations of mutations that maintain the Omicron predicted structural scaffold while progressively optimizing immune evasion. This pattern mirrors broader evolutionary trends reported for XBB.1.5, XBB.1.16, and other advanced Omicron derivatives, in which spike evolution proceeds along narrow, structurally compatible trajectories that balance stability, receptor binding, and antibody escape (5, 6, 8).

While formal selection-pressure analyses, such as HyPhy-based dN/dS estimation, could provide additional insights into the evolutionary significance of the observed mutations, such approaches are most informative when applied to larger, phylogenetically resolved datasets. Future studies incorporating broader genomic datasets may further elucidate the selective forces shaping these variants. In the present study, evolutionary inferences are based on longitudinal intra-host mutational dynamics, co-occurrence patterns, and structural analyses, which are aligned with the primary objective of characterizing the structural and antigenic consequences of SARS-CoV-2 mutations. Finally, while the present study establishes robust computational and structural evidence for altered immunogenic determinants, the functional consequences of these iSNVs on antibody neutralization and T-cell responses will be experimentally validated through dedicated *in vitro* studies currently underway, which will provide direct biological confirmation of the immune evasion potential predicted here.

## Conclusion

This study shows that intra-host evolution of SARS-CoV-2 XBB lineages in vaccinated individuals is dominated by mutations in the spike gene, particularly within the RBD of XBB.1.16 viruses from reinfection and breakthrough cases in the Delhi-NCR region. These mutations arise in highly constrained, non-random constellations: BA.2-derived structural scaffolds are preserved while XBB.1.16-like and XBB.2.3 like marker sets emerge stepwise and remain largely mutually exclusive, suggesting epistatic constraints that may couple local folding stability and receptor competence to immune escape potential. Recurrent substitutions at positions 180, 184, 252-253, 478, and 521 may behave as epistatic hubs, potentially reshaping predicted B-cell and T-cell epitopes at the NTD supersite and RBD base while maintaining modeled ACE2 engagement and trimer integrity, thereby supporting possible immune evasion without compromising predicted structural fitness. Collectively, our computational findings suggest that the emergence and accumulation of spike iSNVs under potential vaccine-induced immune pressure may promote the selection of putatively compatible mutational combinations that could confer a fitness advantage over contemporaneous variants, underscoring the need for longitudinal, structure-informed genomic surveillance to anticipate the emergence of immune-evasive XBB derivatives.

The findings of this study are supported by multiple computational and structural analyses, including solvent-accessible surface area (SASA) assessment, protein–protein interface analysis (ePISA), protein stability predictions (ΔΔG), molecular dynamics simulations (RMSD), and epitope-binding predictions. While these approaches provide valuable insights into the potential structural and antigenic consequences of the identified mutations, direct experimental validation through neutralization assays, binding affinity measurements, and T-cell functional studies are the limitations of the study. Future studies integrating such experimental approaches would further strengthen the biological interpretation of the computational predictions reported here.

## Data Availability

The original contributions presented in the study are publicly available. This data can be found here: https://www.ncbi.nlm.nih.gov/nuccore, accession ID OR915533 to OR915559, OR922269 & OR922270.
