# Supplementary material for: The emergence of putative epistatic mutations and iSNVs in SARS-CoV-2 XBB.1.16 variants linked with alteration in immunogenic determinants
Source: Front Immunol. 2026 Aug 20;17:1846973. doi: 10.3389/fimmu.2026.1846973 (PMC13538099; doi:10.3389/fimmu.2026.1846973)
Supplement: Supplementary file 1 [file Supplementaryfile1.docx]

**SUPPLEMENTARY INFORMATION**

**The emergence of putative epistatic iSNVs in SARS-CoV-2 XBB.1.16 variants linked with alteration in immunogenic determinants**

Mukesh K Jogi¹^,2^#, Sristy Shikha¹#, Pushpendra Singh^3^#, Devesh Sharma^4^, Shreyansh^5^, Prem Kumar Mishra^5^, Aiswariya Priyadarsini^6^, Aahamya Priyadarsini^6^, Deokrishna Kumar Choudhary^7^, Meenu Jain^8^, Ritu Sagar¹, Anuj Kumar¹^,9^, Robin Marwal^4^, Md Kausar Neyaz^4^, Dheeraj Dubey Prakashchand^7^, Rakesh Gupta^4^, Shalini Singh¹^,9^, Pramod Kumar¹^,9^*

**Affiliations**

1: Division of Molecular Biology, ICMR-National Institute of Cancer Prevention and Research, Noida, India

2: ICMR - National Institute for Research in Environmental Health, Bhopal, India

3: Department of Biotechnology, Central University of Haryana, India

4: Govt Institute of Medical Sciences, Greater Noida, India

5: A.N. College, Patna, India

6: Department of Biological Sciences, IISER, Berhampur, India

7: School of Mechanical and Materials Engineering, Indian Institute of Technology (IIT) Mandi, Mandi, Himachal Pradesh 175005, India

8: VRDL, Gajra Raja Medical College, Gwalior-474009, Madhya Pradesh, India

9: Faculty of Medical Research, Academy of Scientific & Innovative Research (AcSIR), Ghaziabad, UP, India

#: equal contribution

*: Correspondence

Division of Molecular Biology, ICMR-National Institute of Cancer Prevention and Research (NICPR), NOIDA (India)

**Email:** [pramod.kumar@icmr.gov.in](mailto:pramod.kumar@icmr.gov.in), [pramodjnu1@gmail.com](mailto:pramodjnu1@gmail.com)

**Structure retrieval mutation introduction and side-chain completion**

After retrieving PDB structure, the antibody chains, crystallographic water molecules, heteroatoms, and buffer molecules were removed, retaining only the NTD polypeptide chain to isolate the native backbone geometry of the region of interest. Point mutations were introduced into the cleaned wild-type coordinate file using VMD (Visual Molecular Dynamics, latest version; Humphrey et al., 1996). The initial protein structure obtained from the RCSB Protein Data Bank consisted of backbone atoms (N, Cα, C) with residue identities specified but lacking explicit side-chain coordinates. Point mutations (E180V, G184V, G252V, and P521S) were introduced by modifying the residue identities directly in the coordinate file. Subsequently, complete atomic structures were reconstructed using the PDBFixer module from OpenMM, which was employed to identify and add missing heavy atoms, including full side chains, based on standard residue templates. Missing residues were not rebuilt, and hydrogen atoms were intentionally omitted at this stage to allow consistent protonation during subsequent simulation setup. The resulting five structures, each comprising a complete heavy-atom model with reconstructed side chains, were subsequently used as starting coordinates for topology generation, hydrogen addition, and molecular dynamics simulation setup in GROMACS following energy minimization and equilibration.

**SI Table 1 |** *Amino acid composition at all four iSNV positions (180, 184, 252, 521) across the wild-type reference and each of the four multi-site mutant simulation systems. Green cells indicate substituted (mutated) residues relative to the wild-type sequence; light grey cells indicate positions retained as wild type within that mutant structure. The wild-type residues at each position are: Glu (E) at 180, Gly (G) at 184, Gly (G) at 252, and Pro (P) at 521. Mutant 1 carries two substitutions (G252V, P521S) while retaining Glu at position 180. Mutant 2 and Mutant 3 each carry three substitutions (E180V, G252V, P521S) while retaining Gly at position 184. Mutant 4 carries substitutions at all four positions simultaneously (E180V, G184V, G252V, P521S). PDB: 7DZX; mutations introduced using VMD Mutator plugin; side chains completed with PDBFixer openMM*

| **Simulation System** | **Label** | **Position 180** | **Position 184** | **Position 252** | **Position 521** |
| --- | --- | --- | --- | --- | --- |
| ***Amino acid substitution*** |  | ***WT: Glu (E)*** | ***WT: Gly (G)*** | ***WT: Gly (G)*** | ***WT: Pro (P)*** |
| **Wild Type (7DZX)** | **WT** | Glu (E) | Gly (G) | Gly (G) | Pro (P) |
| **Mutant 1** | Mut1 | Glu (E) -- WT | Gly (G) -- WT | **Val (V) -- MUT** | **Ser (S) -- MUT** |
| **Mutant 2** | Mut2 | Glu (E) -- WT | **Val (V) -- MUT** | **Val (V) -- MUT** | **Ser (S) -- MUT** |
| **Mutant 3** | Mut3 | **Val (V) -- MUT** | Gly (G) -- WT | **Val (V) -- MUT** | **Ser (S) -- MUT** |
| **Mutant 4** | Mut4 | **Val (V) -- MUT** | **Val (V) -- MUT** | **Val (V) -- MUT** | **Ser (S) -- MUT** |

**Molecular dynamics simulation setup**

All molecular dynamics simulations were performed using GROMACS 2023.1 (Abraham et al., 2015; Van Der Spoel et al., 2005). Each PDB file was processed through the GROMACS pdb2gmx utility to assign topology, partial charges, and bonded parameters using the CHARMM36 all-atom force field (Best et al., 2012; Huang et al., 2017). Protonation states were assigned at neutral pH. The TIP3P explicit water model, the internally consistent solvent parameterization for CHARMM36, was specified at this step. Each protein system was placed at the geometric centre of a cubic simulation box with a minimum distance of 1.2 nm between any protein heavy atom and the nearest box face, ensuring that the protein and its periodic images do not interact across boundary replicas. The solvated box was filled with TIP3P water molecules using gmx solvate, and the system charge was neutralized by replacing water molecules with Na+ and Cl- ions using gmx genion; NaCl was supplemented to a final concentration of 0.15 M to approximate physiological ionic strength. Periodic boundary conditions were applied in all three Cartesian dimensions throughout all simulation phases.

**Energy minimization and equilibration**

Each solvated and ionised system was energy-minimised using the steepest descent algorithm until the maximum force on any atom in the system converged below 1000 kJ mol-1 nm-1, following the convergence criterion used in the GROMACS lysozyme tutorial. Successful minimisation was confirmed by monitoring the smooth decay of the total potential energy to a stable minimum. Equilibration was then conducted in two sequential stages, again following the standard protocol. In the first stage, a 100 ps NVT (constant particle number, volume, and temperature) simulation was run with harmonic positional restraints applied to all protein backbone heavy atoms (force constant 1000 kJ mol-1 nm-2) to allow the solvent and ions to relax and redistribute around the protein while preventing large-scale protein motion. Temperature was maintained at 300 K using the V-rescale velocity-rescaling thermostat (Bussi et al., 2007) with a coupling time constant of 0.1 ps. In the second stage, a 100 ps NPT (constant particle number, pressure, and temperature) equilibration was performed with the same backbone restraints, replacing the fixed-volume constraint with pressure regulation at 1 bar using the Parrinello-Rahman barostat (Parrinello and Rahman, 1981) with a coupling time constant of 2.0 ps and an isothermal compressibility of 4.5 x 10-5 bar-1. Equilibration was considered complete when temperature, pressure, and system density had converged to stable values consistent with 300 K and 1 bar.

**Production molecular dynamics and trajectory collection**

Following equilibration, all positional restraints were removed and each of the five systems was advanced to an unrestrained production MD simulation of 100 ns duration. The integration timestep was 2 fs throughout. Covalent bonds involving hydrogen atoms were constrained using the LINCS algorithm (Hess et al., 1997), and the rigid geometry of TIP3P water molecules was maintained with the SETTLE algorithm (Miyamoto and Kollman, 1992). Long-range electrostatic interactions were computed using Particle Mesh Ewald (Darden et al., 1993) with a real-space cutoff of 1.2 nm and a Fourier grid spacing of 0.16 nm. Van der Waals interactions were truncated at 1.2 nm with long-range dispersion correction applied to both energy and pressure. Temperature and pressure coupling were maintained using the identical V-rescale thermostat and Parrinello-Rahman barostat settings as in the NPT equilibration stage. Coordinates and energies were written to the trajectory every 10 ps, generating 10,000 frames per 100 ns trajectory for each system. All five simulations were run independently and identically on the Jambvanta cluster to ensure rigorous cross-system comparability. Key simulation parameters are summarised in Table 1.

**SI Table 2 | Molecular dynamics simulation parameters applied uniformly across all five systems (WT, Mut1-Mut4), following the GROMACS lysozyme-in-water tutorial protocol (Lemkul, 2019). EM: energy minimization.**

| **Parameter** | **Equilibration** | **Production MD** |
| --- | --- | --- |
| Force field | CHARMM36 | CHARMM36 |
| Water model | TIP3P | TIP3P |
| Box type | Cubic, 1.2 nm clearance | Cubic, 1.2 nm clearance |
| Salt concentration | 0.15 M NaCl | 0.15 M NaCl |
| Integrator | Steepest descent (EM only) | md (leap-frog) |
| Integration timestep | 2 fs | 2 fs |
| Duration | 100 ps NVT + 100 ps NPT | 100 ns |
| Thermostat | V-rescale, tau = 0.1 ps, 300 K | V-rescale, tau = 0.1 ps, 300 K |
| Barostat | Parrinello-Rahman, tau = 2.0 ps, 1 bar | Parrinello-Rahman, tau = 2.0 ps, 1 bar |
| Electrostatics | PME, cutoff 1.2 nm | PME, cutoff 1.2 nm |
| vdW cutoff | 1.2 nm | 1.2 nm |
| Bond constraints | LINCS (H-bonds); SETTLE (H2O) | LINCS (H-bonds); SETTLE (H2O) |
| Backbone restraints | 1000 kJ mol-1 nm-2 | None |
| Energy minimisation criterion | Fmax < 1000 kJ mol-1 nm-1 | -- |
| Trajectory output interval | -- | Every 10 ps |

**Results**

**SI Figure 1:** Clinical meta data association analysis of all samples (A) Ct versus Age group viral load, (B) Ct Versus vaccine status (C) Ct with symptoms status Clinical meta data association analysis of samples having putative epistatic changes (D) Ct values between XBB.1.16-like and XBB.2.3-like variants (E) Association of age group and viral load. (F) Association of vaccination status Ct value. (G) Lineage-specific mutation analysis. (H) Corelation analysis of associations among spike mutations, indicating coordinated mutation co-occurrence and mutual exclusivity during variant evolution.

**SI Figure 2:** I326(in red) and its interaction with four classes of antibody surrounding RBD (in grey).

**SI Figure 3:** Mutating E180 to V180 and its interaction are studied using UCSF Chimera. Distance between E180.C-N188.C significantly decreased from 4.132Å to 3.631Å, affecting hydrophilic and hydrophobic interactions.

***SI Figure 4. A.****NTD – 8D2 Complex (PDB ID- 7DZX) NTD Region- Cyan,8D2 Heavy Chain- Purple 8D2 Light Chain- Yellow* ***B.*** *Residues Mapped: E180(in red), K182(in green), G184(in blue). PDB ID-7DZX was used to prepare the structure.*

**SI Figure 5*:*** *Interaction between NTD and TXG-0078 fab antibody is studied.* ***A****. PDB ID 8SWH was used to prepare the figure. The heavy and light chains of TXG-0078 are coloured pink and blue respectively. The NTDs of the Trimeric S protein are coloured Cyan.* ***B.*** *Mutation of H146K of NTD and its interaction with other residues are studied using ChimeraX.****(A)****H146 forms an H-bond with N149 of NTD providing structural stability.* ***C.*** *On mutation K146 interacts better with S151 of NTD.*

**SI Figure 6:** D253 in forest green forming H-bond with Q1(in pink) on heavy chain of TXG-0078 fab fragment of antibody (3.006Å).

**SI Figure 7:** E180  is mapped on NTD-TXG-0078 complex suggesting E180 appears to be at a distance from antibody binding region, resulting in limited interaction with residues involved in binding complex.

**Supplementary Table 3:** Denoting the accession number and country of origin of the selected references with their pango lineages.

| **S.No** | **ACCESSION NO** | **VARIANTS/LINEAGE** | **ORIGIN** |
| --- | --- | --- | --- |
| 1 | OR582756.1 | XBB.1.5 | USA |
| 2 | OR568636.1 | CH.1.1 | USA |
| 3 | OR578449.1 | XBB1.9.1 | USA |
| 4 | OR578450.1 | XBB1.9.2 | USA |
| 5 | OR578505.1 | XBB.2.3 | USA |
| 6 | OR569223.1 | BA.2.86 | USA |
| 7 | OR584358.1 | BA.2 | USA |
| 8 | OY570844.1 | BA.4 | UK |
| 9 | OR584355.1 | BA.5 | USA |
| 10 | OR582798.1 | EG.5 | USA |
| 11 | OQ859938.1 | BJ.1 | IND |
| 12 | OR357653.1 | BA.2.75 | IND |

**Supplementary Table 4: The sequences with their GISAID accession no**

| **S.No.** | **Sequence ID** | **GISAID Accession ID** |
| --- | --- | --- |
| 1 | hCoV-19/India/Delhi-NCR/NICPR401/23 | EPI_ISL_18598578 |
| 2 | hCoV-19/India/Delhi-NCR/NICPR402/23 | EPI_ISL_18598579 |
| 3 | hCoV-19/India/Delhi-NCR/NICPR404/23 | EPI_ISL_18598580 |
| 4 | hCoV-19/India/Delhi-NCR/NICPR411/23 | EPI_ISL_18598581 |
| 5 | hCoV-19/India/Delhi-NCR/NICPR412/23 | EPI_ISL_18598582 |
| 6 | hCoV-19/India/Delhi-NCR/NICPR413/23 | EPI_ISL_18598583 |
| 7 | hCoV-19/India/Delhi-NCR/NICPR414/23 | EPI_ISL_18598584 |
| 8 | hCoV-19/India/Delhi-NCR/NICPR415/23 | EPI_ISL_18598585 |
| 9 | hCoV-19/India/Delhi-NCR/NICPR417/23 | EPI_ISL_18598586 |
| 10 | hCoV-19/India/Delhi-NCR/NICPR418/23 | EPI_ISL_18598587 |
| 11 | hCoV-19/India/Delhi-NCR/NICPR419/23 | EPI_ISL_18598588 |
| 12 | hCoV-19/India/Delhi-NCR/NICPR420/23 | EPI_ISL_18598589 |
| 13 | hCoV-19/India/Delhi-NCR/NICPR421/23 | EPI_ISL_18598590 |
| 14 | hCoV-19/India/Delhi-NCR/NICPR422/23 | EPI_ISL_18598591 |
| 15 | hCoV-19/India/Delhi-NCR/NICPR423/23 | EPI_ISL_18598592 |
| 16 | hCoV-19/India/Delhi-NCR/NICPR424/23 | EPI_ISL_18598593 |
| 17 | hCoV-19/India/Delhi-NCR/NICPR425/23 | EPI_ISL_18598594 |
| 18 | hCoV-19/India/Delhi-NCR/NICPR426/23 | EPI_ISL_18598595 |
| 19 | hCoV-19/India/Delhi-NCR/NICPR427/23 | EPI_ISL_18598596 |
| 20 | hCoV-19/India/Delhi-NCR/NICPR428/23 | EPI_ISL_18598597 |
| 21 | hCoV-19/India/Delhi-NCR/NICPR429/23 | EPI_ISL_18598598 |
| 22 | hCoV-19/India/Delhi-NCR/NICPR430/23 | EPI_ISL_18598599 |
| 23 | hCoV-19/India/Delhi-NCR/NICPR431/23 | EPI_ISL_18598600 |
| 24 | hCoV-19/India/Delhi-NCR/NICPR434/23 | EPI_ISL_18598601 |
| 25 | hCoV-19/India/Delhi-NCR/NICPR436/23 | EPI_ISL_18598602 |
| 26 | hCoV-19/India/Delhi-NCR/NICPR437/23 | EPI_ISL_18598603 |
| 27 | hCoV-19/India/Delhi-NCR/NICPR440/23 | EPI_ISL_18598604 |
| 28 | hCoV-19/India/Delhi-NCR/NICPR416/23 | EPI_ISL_18604379 |
| 29 | hCoV-19/India/Delhi-NCR/NICPR433/23 | EPI_ISL_18604380 |

**Supplementary Table 5:** The sequences with their NCBI accession no and gene-bank submission ID

| **S.No.** | **GenBank submission** | **Sequence ID** | **Accession ID** |
| --- | --- | --- | --- |
| 1 | SUB14028510 | hCoV-19/India/Delhi-NCR/NICPR401/23 | OR915533 |
| 2 | SUB14028510 | hCoV-19/India/Delhi-NCR/NICPR411/23 | OR915534 |
| 3 | SUB14028510 | hCoV-19/India/Delhi-NCR/NICPR412/23 | OR915535 |
| 4 | SUB14028510 | hCoV-19/India/Delhi-NCR/NICPR413/23 | OR915536 |
| 5 | SUB14028510 | hCoV-19/India/Delhi-NCR/NICPR414/23 | OR915537 |
| 6 | SUB14028510 | hCoV-19/India/Delhi-NCR/NICPR415/23 | OR915538 |
| 7 | SUB14028510 | hCoV-19/India/Delhi-NCR/NICPR417/23 | OR915539 |
| 8 | SUB14028510 | hCoV-19/India/Delhi-NCR/NICPR418/23 | OR915540 |
| 9 | SUB14028510 | hCoV-19/India/Delhi-NCR/NICPR419/23 | OR915541 |
| 10 | SUB14028510 | hCoV-19/India/Delhi-NCR/NICPR402/23 | OR915542 |
| 11 | SUB14028510 | hCoV-19/India/Delhi-NCR/NICPR420/23 | OR915543 |
| 12 | SUB14028510 | hCoV-19/India/Delhi-NCR/NICPR421/23 | OR915544 |
| 13 | SUB14028510 | hCoV-19/India/Delhi-NCR/NICPR422/23 | OR915545 |
| 14 | SUB14028510 | hCoV-19/India/Delhi-NCR/NICPR423/23 | OR915546 |
| 15 | SUB14028510 | hCoV-19/India/Delhi-NCR/NICPR424/23 | OR915547 |
| 16 | SUB14028510 | hCoV-19/India/Delhi-NCR/NICPR425/23 | OR915548 |
| 17 | SUB14028510 | hCoV-19/India/Delhi-NCR/NICPR427/23 | OR915549 |
| 18 | SUB14028510 | hCoV-19/India/Delhi-NCR/NICPR426/23 | OR915550 |
| 19 | SUB14028510 | hCoV-19/India/Delhi-NCR/NICPR428/23 | OR915551 |
| 20 | SUB14028510 | hCoV-19/India/Delhi-NCR/NICPR429/23 | OR915552 |
| 21 | SUB14028510 | hCoV-19/India/Delhi-NCR/NICPR430/23 | OR915553 |
| 22 | SUB14028510 | hCoV-19/India/Delhi-NCR/NICPR431/23 | OR915554 |
| 23 | SUB14028510 | hCoV-19/India/Delhi-NCR/NICPR434/23 | OR915555 |
| 24 | SUB14028510 | hCoV-19/India/Delhi-NCR/NICPR436/23 | OR915556 |
| 25 | SUB14028510 | hCoV-19/India/Delhi-NCR/NICPR437/23 | OR915557 |
| 26 | SUB14028510 | hCoV-19/India/Delhi-NCR/NICPR404/23 | OR915558 |
| 27 | SUB14028510 | hCoV-19/India/Delhi-NCR/NICPR440/23 | OR915559 |
| 28 | SUB14028483 | hCoV-19/India/DL-NCR_NICPR416/2023 | OR922269 |
| 29 | SUB14028483 | hCoV-19/India/DL-NCR_NICPR433/2023 | OR922270 |

**Supplementary Table 6:** The reference sequences with their NCBI accession no and country of origin

| S.NO | NCBI ACCESSION | PANGOLIN | COUNTRY OF ORIGIN |
| --- | --- | --- | --- |
| 1 | BS007749.1 | JN.1 | JAP |
| 2 | OR568636.1 | CH.1.1 | USA |
| 3 | OR569223.1 | BA.2.86 | USA |
| 4 | OR578449.1 | XBB1.9.1 | USA |
| 5 | OR578450.1 | XBB1.9.2 | USA |
| 6 | OR578505.1 | XBB.2.3 | USA |
| 7 | OR579035.1 | XBB1.16 | USA |
| 8 | OR582756.1 | XBB.1.5 | USA |
| 9 | OR582798.1 | EG.5 | USA |
| 10 | OR584355.1 | BA.5 | USA |
| 11 | OR584358.1 | BA.2 | USA |
| 12 | OR584409.1 | BA.4 | USA |
| 13 | OY254839.1 | GER | GER |
| 14 | OY570844.1 | UK | WALES |
| 15 | OY726350.1 | UK | UK |
| 16 | PP092326.1 | XBB.1.16.6 | USA |
| 17 | OQ859938.1 | BJ.1\IND | BJ.1\IND |

**Supplementary Table 7: Epitope mapping of the patient’s sample sequences for CD8+ T-cell**

| CODE | S_POS | POS | EPITOPE | CORE | MHC | SARS_CoV_2_REF | S_N411 | S_N413 | S_N414 | S_N415 | S_N417 | S_N424 | S_N425 | S_N436 | S_N442 | S_N445 | S_N448 | S_N451 | S_N454 |
| --- | --- | --- | --- | --- | --- | --- | --- | --- | --- | --- | --- | --- | --- | --- | --- | --- | --- | --- | --- |
| S39-53 | 39 | 39-53 | PDKVFRSSVLHSTQD | FRSSVLHST | HLA-DPA10103-DPB11011 | 1 | 1 | 1 | 1 | 1 | 1 | 1 | 1 | 1 | 0 | - | 0 | 0 | 0 |
|  |  |  |  |  | HLA-DPA10301-DPB11031 |  |  |  |  |  |  |  |  |  |  |  |  |  |  |
|  |  |  |  |  | HLA-DQA10401-DQB10401 |  |  |  |  |  |  |  |  |  |  |  |  |  |  |
|  |  |  |  |  | HLA-DQA10402-DQB10402 |  |  |  |  |  |  |  |  |  |  |  |  |  |  |
| S75-89 | 75 | 75-89 | GTKRFDNPVLPFNDG | RFDNPVLPF | HLA-DPA10301-DPB11031 | 1 | 1 | 1 | 0 | 0 | 1 | 0 | 1 | 0 | 0 | - | 0 | 0 | 0 |
| S132-147 | 132 | 132-147 (DELETION AT 145) | EFQFCNDPFLDVY**K**K | FCNDPFLDV | HLA-DPA10103-DPB11011 | 0 | 0 | 0 | 1 | 0 | 0 | 0 | 0 | 0 | 0 | - | 0 | 0 | 0 |
|  |  |  | EFQFCNDPFLDVY**Q**K | FCNDPFLDV | HLA-DPA10103-DPB11011 | 0 | 0 | 1 | 0 | 1 | 1 | 1 | 1 | 1 | 1 | - | 1 | 0 | 0 |
| S136-151 | 136 | 136-151 (DELETION AT 145) | CNDPFLDVYKKNNKS | FLDVYKKNN | DRB1_0802 | 0 | 0 | 0 | 1 | 0 | 0 | 0 | 0 | 0 | 0 | - | 0 | 0 | 0 |
| S137-151 | 137 | 137-151 | NDPFLGVYYHKNNKS | FLGVYYHKN | HLA-DPA10103-DPB110101 | 1 | 0 | 0 | 0 | 0 | 0 | 0 | 0 | 0 | 0 | - | 0 | 0 | 0 |
|  |  |  |  |  | HLA-DPA10301-DPB110301 |  |  |  |  |  |  |  |  |  |  |  |  |  |  |
| S137-152 | 137 | 137-152 (DELETION AT 145) | NDPFLDVYKKNNKSW | LDVYKKNNK | DRB1_1302 | 0 | 0 | 0 | 1 | 0 | 0 | 0 | 0 | 0 | 0 | - | 0 | 0 | 0 |
| S167-181 | 167 | 167-181 | TFEYVSQPFLMDL**E**G | YVSQPFLMD | HLA-DPA10103-DPB11011 | 1 | 1 | 0 | 1 | 1 | 0 | 1 | 1 | 0 | 0 | - | 0 | 0 | 0 |
|  |  |  |  |  | HLA-DPA10201-DPB11021 |  |  |  |  |  |  |  |  |  |  |  |  |  |  |
|  |  |  |  |  | HLA-DPA10301-DPB11031 |  |  |  |  |  |  |  |  |  |  |  |  |  |  |
|  |  |  | TFEYVSQPFLMDL**V**G | YVSQPFLMD | HLA-DPA10103-DPB11011 | 0 | 0 | 1 | 0 | 0 | 1 | 0 | 0 | 1 | 0 | - | 0 | 0 | 0 |
|  |  |  |  |  | HLA-DPA10301-DPB11031 | 0 |  |  |  |  |  |  |  |  |  |  |  |  |  |
| S171-185 | 171 | 171-185 | VSQPFLMDLVGKEGN | FLMDLVGKE | DRB1_0405 | 0 | 0 | 1 | 0 | 0 | 1 | 0 | 0 | 1 | 0 | - | 0 | 0 | 0 |
| S182-196 | 182 | 182-196 | **EEG**NFKNLREFVFKN | FKNLREFVF | DRB1_1201 | 0 | 0 | 0 | 1 | 0 | 0 | 0 | 0 | 0 | 0 | - | 0 | 0 | 0 |
|  |  |  | **KEG**NFKNLREFVFKN | FKNLREFVF | DRB1_1201 | 0 | 1 | 1 | 0 | 1 | 1 | 0 | 0 | 1 | 1 | - | 0 | 1 | 0 |
|  |  |  | **KEV**NFKNLREFVFKN | FKNLREFVF | DRB1_1201 | 0 | 0 | 0 | 0 | 0 | 0 | 0 | 1 | 0 | 0 | - | 0 | 0 | 0 |
|  |  |  | **NEG**NFKNLREFVFKN | FKNLREFVF | DRB1_1201 | 0 | 0 | 0 | 0 | 0 | 0 | 1 | 0 | 0 | 0 | - | 0 | 0 | 0 |
|  |  |  | **EEV**NFKNLREFVFKN | FKNLREFVF | DRB1_1201 | 0 | 0 | 0 | 0 | 0 | 0 | 0 | 0 | 0 | 0 | - | 1 | 0 | 0 |
|  |  |  | **KQG**NFKNLREFVFKN | FKNLREFVF | DRB1_1201 | 1 | 0 | 0 | 0 | 0 | 0 | 0 | 0 | 0 | 0 | - | 0 | 0 | 0 |
| S194-208 | 194 | 194-208 | FKNIDGYFKIYSKHT | KSYIKFYGD | HLA-DPA10201-DPB11021 | 1 | 1 | 1 | 1 | 1 | 1 | 1 | 1 | 1 | 1 | - | 1 | 1 | 0 |
| S195-209 | 195 | 195-209 | KNIDGYFKIYSKHTP | KSYIKFYGD | HLA-DPA10201-DPB11021 | 1 | 1 | 1 | 1 | 1 | 1 | 1 | 1 | 1 | 1 | - | 1 | 1 | 0 |
|  |  |  |  |  | HLA-DPA10301-DPB11031 |  |  |  |  |  |  |  |  |  |  |  |  |  |  |
| S196-210 | 196 | 196-210 | NIDGYFKIYSKHTPI | KSYIKFYGD | HLA-DPA10201-DPB11021 | 1 | 1 | 1 | 1 | 1 | 1 | 1 | 1 | 1 | 1 | - | 1 | 1 | 0 |
| S197-211 | 197 | 197-211 | IDGYFKIYSKHTPIN | FKIYSKHTP | DRB1_1302 | 1 | 1 | 1 | 1 | 1 | 1 | 1 | 1 | 1 | 1 | - | 1 | 1 | 0 |
|  |  |  |  | KSYIKFYGD | HLA-DPA10201-DPB11021 | 1 | 1 | 1 | 1 | 1 | 1 | 1 | 1 | 1 | 1 | - | 1 | 1 | 0 |
| S207-221 | 207 | 207-221 | HTPINL**E**RDLPQGFS | INLERDLPQ | HLA-DQA10101-DQB10201 | 0 | 1 | 1 | 1 | 1 | 1 | 1 | 1 | 1 | 1 | - | 1 | 1 | 0 |
|  |  |  |  |  | HLA-DQA10102-DQB10202 |  |  |  |  |  |  |  |  |  |  |  |  |  |  |
|  |  |  | HTPINL**V**RDLPQGFS | INLVRDLPQ | HLA-DQA10101-DQB10201 | 1 | 0 | 0 | 0 | 0 | 0 | 0 | 0 | 0 | 0 | - | 0 | 0 | 0 |
|  |  |  |  |  | HLA-DQA10102-DQB10202 |  |  |  |  |  |  |  |  |  |  |  |  |  |  |
| S216-230 | 216 | 216-230 | LPQGFSALEPLVDLP | FSALEPLVD | DRB1_0405 | 1 | 1 | 1 | 1 | 1 | 1 | 1 | 1 | 1 | 1 | - | 1 | 1 | 0 |
| S229-243 | 229 | 229-243 | LPIGINITRFQTLLA | INITRFQTL | HLA-DPA10103-DPB11011 | 1 | 0 | 1 | 1 | 1 | 1 | 1 | 1 | 1 | 1 | - | 1 | 1 | 1 |
|  |  |  |  |  | HLA-DPA10301-DPB11031 |  |  |  |  |  |  |  |  |  |  |  |  |  |  |
| S230-246 | 230 | 230-246 (DELETION AT 243,244) | PIGINITRFQTLLHR | INITRFQTL | HLA-DPA10103-DPB11011 | 0 | 1 | 0 | 0 | 0 | 0 | 0 | 0 | 0 | 0 | - | 0 | 0 | 0 |
| S255-269 | 255 | 255-269 | SSGW**T**AGAAAYYVGY | TAGAAAYYV | HLA-DQA10301-DQB10301 | 1 | 1 | 1 | 1 | 1 | 1 | 1 | 1 | 1 | 1 | - | 1 | 1 | 0 |
|  |  |  | SSGW**A**AGAAAYYVGY | AAGAAAYYV | HLA-DQA10301-DQB10301 | 0 | 0 | 0 | 0 | 0 | 0 | 0 | 0 | 0 | 0 | - | 0 | 0 | 1 |
| S269-283 | 269 | 269-283 | YLQPRTFLLKYNENG | PRTFLLKYN | HLA-DQA10401-DQB10401 | 1 | 1 | 1 | 1 | 1 | 1 | 1 | 1 | 1 | 1 | - | 1 | 1 | 1 |
|  |  |  |  |  | HLA-DQA10402-DQB10402 |  |  |  |  |  |  |  |  |  |  |  |  |  |  |
| S272-286 | 272 | 272-286 | PRTFLLKYNENGTIT | FLLKYNENG | DRB1_0802 | 1 | 1 | 1 | 1 | 1 | 1 | 1 | 1 | 1 | 1 | - | 1 | 1 | 1 |
| S300-314 | 300 | 300-314 | KCTLKSFTVEKGIYQ | LKSFTVEKG | HLA-DQA10401-DQB10401 | 1 | 1 | 1 | 1 | 1 | 1 | 1 | 1 | 1 | 0 | - | 1 | 0 | 0 |
|  |  |  |  |  | HLA-DQA10402-DQB10402 |  |  |  |  |  |  |  |  |  |  |  |  |  |  |
| S308-322 | 308 | 308-322 | VEKGIYQTSNFRVQP | IYQTSNFRV | DRB1_0701 | 1 | 1 | 1 | 1 | 1 | 1 | 1 | 1 | 1 | 0 | - | 1 | 0 | 0 |
| S310-324 | 310 | 310-324 | KGIYQTSNFRVQP**I**E | YQTSNFRVQ | HLA-DPA10103-DPB11011 | 0 | 0 | 0 | 1 | 0 | 0 | 0 | 0 | 0 | 0 | - | 0 | 0 | 0 |
|  |  |  | KGIYQTSNFRVQP**T**E | YQTSNFRVQ | HLA-DPA10103-DPB11011 | 1 | 1 | 1 | 0 | 1 | 1 | 1 | 1 | 1 | 0 | - | 1 | 1 | 0 |
| S311-325 | 311 | 311-325 | GIYQTSNFRVQP**I**ES | TSNFRVQPI | HLA-DQA10401-DQB10401 | 0 | 0 | 0 | 1 | 0 | 0 | 0 | 0 | 0 | 0 | - | 0 | 0 | 0 |
|  |  |  |  |  | HLA-DQA10402-DQB10402 |  |  |  |  |  |  |  |  |  |  |  |  |  |  |
|  |  |  | GIYQTSNFRVQP**T**ES | TSNFRVQPT | HLA-DQA10401-DQB10401 | 1 | 1 | 1 | 0 | 1 | 1 | 1 | 1 | 1 | 0 | - | 1 | 1 | 0 |
|  |  |  |  |  | HLA-DQA10402-DQB10402 |  |  |  |  |  |  |  |  |  |  |  |  |  |  |
| S314-328 | 314 | 314-328 | QTSNFRVQP**I**ESIVR | FRVQPIESI | HLA-DQA10501-DQB10501 | 0 | 0 | 0 | 1 | 0 | 0 | 0 | 0 | 0 | 0 | - | 0 | 0 | 0 |
|  |  |  | QTSNFRVQP**T**ESIVR | FRVQPTESI | HLA-DQA10501-DQB10501 | 1 | 1 | 1 | 0 | 1 | 1 | 1 | 1 | 1 | 0 | - | 1 | 1 | 0 |
| S315-329 | 315 | 315-329 | TSNFRVQPTESIVRF | FRVQPTESI | DRB1_0101 | 1 | 1 | 1 | 0 | 1 | 1 | 1 | 1 | 1 | 0 | - | 1 | 1 | 0 |
| S338-352 | 338 | 338-352 | F**H**EVFNAT**T**FASVYA | FNATTFASV | HLA-DPA10103-DPB11011 | 0 | 1 | 1 | 1 | 1 | 1 | 1 | 1 | 1 | 0 | - | 1 | 1 | 0 |
|  |  |  |  |  | HLA-DPA10301-DPB11031 |  |  |  |  |  |  |  |  |  |  |  |  |  |  |
|  |  |  | F**G**EVFNAT**R**FASVYA | FNATRFASV | HLA-DPA10103-DPB110101 | 1 | 0 | 0 | 0 | 0 | 0 | 0 | 0 | 0 | 0 | - | 0 | 0 | 0 |
|  |  |  |  |  | HLA-DPA10201-DPB110201 |  |  |  |  |  |  |  |  |  |  |  |  |  |  |
|  |  |  |  |  | HLA-DPA10301-DPB110301 |  |  |  |  |  |  |  |  |  |  |  |  |  |  |
| S343-357 | 343 | 343-357 | NATRFASVYAWNRKR | RFASVYAWN | HLA-DPA10301-DPB110301 | 1 | 0 | 0 | 0 | 0 | 0 | 0 | 0 | 0 | 0 | - | 0 | 0 | 0 |
| S346-360 | 346 | 346-360 | **R**FASVYAWNRKRISN | VYAWNRKRI | DRB1_1302 | 1 | 0 | 0 | 0 | 0 | 0 | 0 | 0 | 0 | 0 | - | 0 | 0 | 0 |
|  |  |  | **T**FASVYAWNRKRISN | VYAWNRKRI | DRB1_1302 | 0 | 1 | 1 | 1 | 1 | 1 | 1 | 1 | 1 | 0 | - | 1 | 1 | 0 |
| S371-385 | 371 | 371-385 | SASFSTFKCYGVSPT | FSTFKCYGV | HLA-DQA10501-DQB10501 | 1 | 0 | 0 | 0 | 0 | 0 | 0 | 0 | 0 | 0 | - | 0 | 0 | 0 |
| S372-386 | 372 | 372-386 | APFFAFKCYGVSPIK | FAFKCYGVS | HLA-DPA10103-DPB11011 | 0 | 1 | 0 | 0 | 0 | 0 | 0 | 0 | 0 | 0 | - | 0 | 0 | 0 |
| S372-386 | 372 | 372-386 | APFFAFKCYGVSPTK | FAFKCYGVS | HLA-DPA10103-DPB11011 | 0 | 0 | 1 | 1 | 1 | 1 | 1 | 1 | 1 | 0 | - | 1 | 1 | 1 |
| S394-408 | 394 | 394-408 | NVYADSFVIRG**N**EV**S** | ADSFVIRGN | HLA-DQA10401-DQB10401 | 0 | 1 | 1 | 1 | 1 | 1 | 1 | 1 | 1 | 0 | - | 1 | 1 | 1 |
|  |  |  |  |  | HLA-DQA10402-DQB10402 |  |  |  |  |  |  |  |  |  |  |  |  |  |  |
|  |  |  | NVYADSFVIRG**D**EV**R** | ADSFVIRGD | HLA-DQA10401-DQB10401 | 1 | 0 | 0 | 0 | 0 | 0 | 0 | 0 | 0 | 0 | - | 0 | 0 | 0 |
|  |  |  |  |  | HLA-DQA10402-DQB10402 |  |  |  |  |  |  |  |  |  |  |  |  |  |  |
| S397-411 | 397 | 397-411 | ADSFVIRG**N**EV**S**QIA | FVIRGNEVS | DRB1_0802 | 0 | 1 | 1 | 1 | 1 | 1 | 1 | 1 | 1 | 0 | - | 1 | 1 | 1 |
|  |  |  | ADSFVIRG**D**EV**R**QIA | FVIRGDEVR | DRB1_1302 | 1 | 0 | 0 | 0 | 0 | 0 | 0 | 0 | 0 | 0 | - | 0 | 0 | 0 |
| S399-413 | 399 | 399-413 | SFVIRGDEVRQIAPG | IRGDEVRQI | HLA-DQA10102-DQB10202 | 1 | 0 | 0 | 0 | 0 | 0 | 0 | 0 | 0 | 0 | - | 0 | 0 | 0 |
|  |  |  |  |  | HLA-DQA10501-DQB10501 |  |  |  |  |  |  |  |  |  |  |  |  |  |  |
| S426-440 | 426 | 426-440 | PDDFTGCVIAWNSNK | FTGCVIAWN | HLA-DQA10102-DQB10202 | 0 | 1 | 1 | 1 | 1 | 1 | 1 | 1 | 1 | 0 | - | 1 | 1 | 1 |
| S429-444 | 429 | 429-444 | TGCVIAWNSN**K**LDSK | VIAWNSNKL | HLA-DQA10401-DQB10401 | 0 | 1 | 1 | 1 | 1 | 1 | 1 | 1 | 1 | 0 | - | 1 | 1 | 1 |
|  |  |  |  |  | HLA-DQA10402-DQB10402 |  |  |  |  |  |  |  |  |  |  |  |  |  |  |
|  |  |  |  |  | HLA-DQA10501-DQB10501 |  |  |  |  |  |  |  |  |  |  |  |  |  |  |
| S430-444 | 430 | 430-444 | TGCVIAWNSN**N**LDSK | VIAWNSNNL | HLA-DQA10401-DQB10401 | 1 | 0 | 0 | 0 | 0 | 0 | 0 | 0 | 0 | 0 | - | 0 | 0 | 0 |
|  |  |  |  |  | HLA-DQA10402-DQB10402 |  |  |  |  |  |  |  |  |  |  |  |  |  |  |
|  |  |  |  |  | HLA-DQA10501-DQB10501 |  |  |  |  |  |  |  |  |  |  |  |  |  |  |
| S446-460 | 446 | 446-460 | **S**GNYNYLYRLFR**K**S**K** | YNYLYRLFR | DRB1_1302 | 0 | 1 | 1 | 1 | 1 | 1 | 1 | 1 | 1 | 0 | - | 1 | 1 | 0 |
|  |  |  | **S**GNYNYLYRLFR**R**S**K** | YNYLYRLFR | DRB1_1302 | 0 | 0 | 0 | 0 | 0 | 0 | 0 | 0 | 0 | 0 | - | 0 | 0 | 1 |
|  |  |  | **G**GNYNYLYRLFR**K**S**N** | YNYLYRLFR | DRB1_1302 | 1 | 0 | 0 | 0 | 0 | 0 | 0 | 0 | 0 | 0 | - | 0 | 0 | 0 |
| S448-462 | 448 | 448-462 | NYNYLYRLFR**K**S**K**LK | YLYRLFRKS | HLA-DPA10103-DPB11011 | 0 | 1 | 1 | 1 | 1 | 1 | 1 | 1 | 1 | 0 | - | 1 | 1 | 0 |
|  |  |  | NYNYLYRLFR**R**S**K**LK | YLYRLFRRS | HLA-DPA10103-DPB110101 | 0 | 0 | 0 | 0 | 0 | 0 | 0 | 0 | 0 | 0 | - | 0 | 0 | 1 |
|  |  |  | NYNYLYRLFR**K**S**N**LK | YLYRLFRKS | HLA-DPA10103-DPB110101 | 1 | 0 | 0 | 0 | 0 | 0 | 0 | 0 | 0 | 0 | - | 0 | 0 | 0 |
| S449-463 | 449 | 449-463 | YNYLYRLFR**K**S**K**LKP | YRLFRKSKL | DRB1_1302 | 0 | 1 | 1 | 1 | 1 | 1 | 1 | 1 | 1 | 0 | - | 1 | 1 | 0 |
|  |  |  | YNYLYRLFR**R**S**K**LKP | YRLFRRSKL | DRB1_1302 | 0 | 0 | 0 | 0 | 0 | 0 | 0 | 0 | 0 | 0 | - | 0 | 0 | 1 |
|  |  |  | YNYLYRLFR**K**S**N**LKP | YRLFRKSNL | DRB1_1302 | 1 | 0 | 0 | 0 | 0 | 0 | 0 | 0 | 0 | 0 | - | 0 | 0 | 0 |
| S489-503 | 489 | 489-503 | YFPLQSYGFQPTNGV | LQSYGFQPT | HLA-DPA10103-DPB110101 | 1 | 0 | 0 | 0 | 0 | 0 | 0 | 0 | 0 | 0 | - | 0 | 0 | 0 |
| S493-507 | 493 | 493-507 | QSYGF**Q**PT**N**GVGYQP | FQPTNGVGY | DRB1_0701 | 1 | 0 | 0 | 0 | 0 | 0 | 0 | 0 | 0 | 0 | - | 0 | 0 | 0 |
|  |  |  | QSYGF**R**PT**Y**GVGYQP | FRPTYGVGY | DRB1_0701 | 0 | 1 | 1 | 1 | 1 | 1 | 1 | 1 | 1 | 0 | - | 0 | 0 | 0 |
| S512-526 | 512 | 512-526 | VLSFELLHAPATVCG | FELLHAPAT | DRB1_0101 | 1 | 0 | 0 | 0 | 0 | 0 | 0 | 0 | 1 | 0 | - | 0 | 0 | 0 |
| S540-554 | 540 | 540-554 | NFNFNGLTGTGVLTE | FNGLTGTGV | DRB1_0101 | 1 | 1 | 1 | 1 | 1 | 1 | 1 | 1 | 1 | 1 | - | 1 | 1 | 0 |
| S554-568 | 554 | 554-568 | ESNKKFLPFQQFGRD | KKFLPFQQF | HLA-DPA10201-DPB11021 | 1 | 1 | 1 | 1 | 1 | 1 | 1 | 1 | 1 | 1 | - | 1 | 1 | 0 |
|  |  |  |  |  | HLA-DPA10301-DPB11031 |  |  |  |  |  |  |  | 0 |  |  |  |  |  |  |
| S556-570 | 556 | 556-570 | NKKFLPFQQFGRDIA | FLPFQQFGR | DRB1_1302 | 1 | 1 | 1 | 1 | 1 | 1 | 1 | 1 | 1 | 1 | - | 1 | 1 | 0 |
| S566-580 | 566 | 566-580 | GRDIADTTDAVRDPQ | IADTTDAVR | HLA-DQA10102-DQB10202 | 0 | 0 | 0 | 0 | 0 | 0 | 0 | 0 | 0 | 1 | - | 1 | 1 | 1 |
|  |  |  |  |  | HLA-DQA10101-DQB10201 | 1 | 1 | 1 | 1 | 1 | 1 | 1 | 1 | 1 | 1 | - | 1 | 1 | 1 |
| S618-632 | 618 | 618-632 | TEVPVAIHADQLTPT | PVAIHADQL | HLA-DQA10102-DQB10202 | 1 | 1 | 1 | 1 | 1 | 1 | 1 | 1 | 1 | 1 | - | 1 | 1 | 0 |
|  |  |  |  |  | HLA-DQA10501-DQB10501 |  |  |  |  |  |  |  |  |  |  |  |  |  |  |
| S671-685 | 671 | 671-685 | CASYQTQTKSHRRAR | YQTQTKSHR | DRB1_1302 | 0 | 1 | 1 | 1 | 1 | 1 | 1 | 1 | 1 | 1 | - | 1 | 1 | 1 |
| S714-728 | 714 | 714-728 | IPTNFTISVTTEILP | FTISVTTEI | DRB1_0701 | 1 | 1 | 1 | 1 | 1 | 1 | 1 | 1 | 1 | 0 | - | 1 | 1 | 0 |
| S761-775 | 761 | 761-775 | TQLKRALTGIAVEQD | KRALTGIAV | HLA-DQA10101-DQB10201 | 0 | 1 | 1 | 1 | 1 | 1 | 1 | 1 | 1 | 0 | - | 1 | 1 | 0 |
|  |  |  |  |  | HLA-DQA10102-DQB10202 |  |  |  |  |  |  |  |  |  |  |  |  |  |  |
| S762-776 | 762 | 762-776 | QL**K**RALTGIAVEQDK | RALTGIAVE | HLA-DPA10201-DPB11021 | 0 | 1 | 1 | 1 | 1 | 1 | 1 | 1 | 1 | 0 | - | 1 | 1 | 0 |
|  |  |  |  |  | HLA-DPA10301-DPB11031 |  |  |  |  |  |  |  |  |  |  |  |  |  |  |
|  |  |  |  |  | HLA-DQA10102-DQB10202 |  |  |  |  |  |  |  |  |  |  |  |  |  |  |
|  |  |  | QL**N**RALTGIAVEQDK | RALTGIAVE | HLA-DPA10201-DPB110201 | 1 | 0 | 0 | 0 | 0 | 0 | 0 | 0 | 0 | 1 | - | 0 | 0 | 0 |
|  |  |  |  |  | HLA-DPA10301-DPB110301 |  |  |  |  |  |  |  |  |  |  |  |  |  |  |
|  |  |  |  |  | HLA-DQA10102-DQB10202 |  |  |  |  |  |  |  |  |  |  |  |  |  |  |
| S763-777 | 763 | 763-777 | LKRALTGIAVEQDKN | LTGIAVEQD | DRB1_0405 | 0 | 0 | 0 | 0 | 0 | 0 | 0 | 0 | 0 | 0 | - | 0 | 1 | 0 |
|  |  |  |  |  | HLA-DQA10101-DQB10201 |  |  |  |  |  |  |  |  |  |  |  |  |  |  |
|  |  |  |  |  | HLA-DQA10102-DQB10202 |  |  |  |  |  |  |  |  |  |  |  |  |  |  |
| S764-778 | 764 | 764-778 | **K**RALTGIAVEQDKNT | LTGIAVEQD | DRB1_0405 | 0 | 1 | 1 | 1 | 1 | 1 | 1 | 1 | 1 | 0 | - | 1 | 0 | 0 |
|  |  |  |  |  | HLA-DQA10101-DQB10201 |  |  |  |  |  |  |  |  |  |  |  |  |  |  |
|  |  |  |  |  | HLA-DQA10102-DQB10202 |  |  |  |  |  |  |  |  |  |  |  |  |  |  |
|  |  |  | **N**RALTGIAVEQDKNT | LTGIAVEQD | DRB1_0405 | 1 | 0 | 0 | 0 | 0 | 0 | 0 | 0 | 0 | 1 | - | 0 | 0 | 0 |
|  |  |  |  |  | HLA-DQA10101-DQB10201 |  |  |  |  |  |  |  |  |  |  |  |  |  |  |
|  |  |  |  |  | HLA-DQA10102-DQB10202 |  |  |  |  |  |  |  |  |  |  |  |  |  |  |
| S778-792 | 778 | 778-792 | TQEVFAQVKQIYKTP | VFAQVKQIY | DRB1_1201 | 1 | 1 | 1 | 1 | 1 | 1 | 1 | 1 | 1 | 1 | - | 1 | 0 | 0 |
|  |  |  |  |  | DRB1_1302 |  |  |  |  |  |  |  |  |  |  |  |  |  |  |
| S785-799 | 785 | 785-799 | VKQIYKTPPIKYFGG | IYKTPPIKY | DRB1_1201 | 0 | 1 | 1 | 1 | 1 | 1 | 1 | 1 | 1 | 0 | - | 1 | 0 | 0 |
| S790-804 | 790 | 790-804 | KTPPIKYFGGFNFSQ | IKYFGGFNF | DRB1_1201 | 0 | 1 | 1 | 1 | 1 | 1 | 1 | 1 | 1 | 0 | - | 1 | 0 | 0 |
| S797-811 | 797 | 797-811 | FGGFNFSQILPDPSK | FNFSQILPD | DRB1_0405 | 1 | 1 | 1 | 1 | 1 | 1 | 1 | 1 | 1 | 1 | - | 1 | 0 | 0 |
|  |  |  |  |  | HLA-DQA10102-DQB10202 |  |  |  |  |  |  |  |  |  |  |  |  |  |  |
| S802-816 | 802 | 802-816 | FSQILPDPSKPSKRS | ILPDPSKPS | HLA-DQA10501-DQB10501 | 1 | 1 | 1 | 1 | 1 | 1 | 1 | 1 | 1 | 1 | - | 1 | 0 | 0 |
| S811-825 | 811 | 811-825 | KPSKRSFIEDLLFNK | KRSFIEDLL | HLA-DQA10101-DQB10201 | 1 | 1 | 1 | 1 | 1 | 1 | 1 | 1 | 1 | 1 | - | 1 | 0 | 0 |
|  |  |  |  |  | HLA-DQA10102-DQB10202 |  |  |  |  |  |  |  |  |  |  |  |  |  |  |
| S812-826 | 812 | 812-826 | PSKRSFIEDLLFNKV | RSFIEDLLF | HLA-DQA10101-DQB10201 | 1 | 1 | 1 | 1 | 1 | 1 | 1 | 1 | 1 | 1 | - | 1 | 0 | 0 |
| S818-832 | 818 | 818-832 | IEDLLFNKVTLADAG | LTVKNFLLD | HLA-DPA10301-DPB11031 | 1 | 1 | 1 | 1 | 1 | 1 | 1 | 1 | 1 | 1 | - | 1 | 1 | 0 |
| S919-933 | 919 | 919-933 | NQKLIANQFNSAIGK | LIANQFNSA | HLA-DPA10103-DPB11011 | 1 | 1 | 1 | 1 | 1 | 1 | 1 | 1 | 1 | 1 | - | 1 | 1 | 1 |
|  |  |  |  |  | HLA-DPA10301-DPB11031 |  |  |  |  |  |  |  |  |  |  |  |  |  |  |
| S947-961 | 947 | 947-961 | KLQDVVN**H**NAQALNT | VVNHNAQAL | HLA-DQA10501-DQB10501 | 0 | 1 | 1 | 1 | 1 | 1 | 1 | 1 | 1 | 1 | - | 1 | 1 | 0 |
|  |  |  | KLQDVVN**Q**NAQALNT | VVNQNAQAL | HLA-DQA10501-DQB10501 | 1 | 0 | 0 | 0 | 0 | 0 | 0 | 0 | 0 | 0 | - | 0 | 0 | 0 |
| S953-972 | 953 | 953-972 (DELETION AT 963,964,965,966,967) | NENFKMWSTISKFGA | FKMWSTISK | DRB1_1302 | 0 | 0 | 0 | 0 | 0 | 0 | 0 | 0 | 0 | 0 | - | 0 | 0 | 1 |
| S953-967 | 953 | 953-967 | NQNAQALNTLVKQLS | KVLTNLAQA | HLA-DPA10201-DPB110201 | 1 | 0 | 0 | 0 | 0 | 0 | 0 | 0 | 0 | 0 | - | 0 | 0 | 0 |
| S978-992 | 978 | 978-992 | NDILSRLDKVEAEVQ | LSRLDKVEA | DRB1_0802 | 0 | 0 | 0 | 1 | 0 | 0 | 0 | 0 | 0 | 0 | - | 0 | 0 | 0 |
| S1009-1023 | 1009 | 1009-1023 | TQQLIRAAEIR**A**SAN | IRAAEIRAS | HLA-DQA10401-DQB10401 | 1 | 1 | 1 | 0 | 1 | 1 | 1 | 1 | 1 | 0 | - | 1 | 1 | 1 |
|  |  |  |  |  | HLA-DQA10402-DQB10402 |  |  |  |  |  |  |  |  |  |  |  |  |  |  |
|  |  |  | TQQLIRAAEIR**V**SAN | IRAAEIRVS | HLA-DQA10401-DQB10401 | 0 | 0 | 0 | 1 | 0 | 0 | 0 | 0 | 0 | 0 | - | 0 | 0 | 0 |
|  |  |  |  |  | HLA-DQA10402-DQB10402 |  |  |  |  |  |  |  |  |  |  |  |  |  |  |
| S1016-1030 | 1016 | 1016-1030 | AEIR**A**SANLAATKMS | RASANLAAT | HLA-DPA10301-DPB11031 | 1 | 1 | 1 | 0 | 1 | 1 | 1 | 1 | 1 | 1 | - | 1 | 1 | 1 |
|  |  |  | AEIR**V**SANLAATKMS | RVSANLAAT | HLA-DPA10301-DPB11031 | 0 | 0 | 0 | 1 | 0 | 0 | 0 | 0 | 0 | 0 | - | 0 | 0 | 0 |
| S1039-1053 | 1039 | 1039-1053 | RVDFCGKGYHLMSFP | FCGKGYHLM | HLA-DPA10103-DPB11011 | 1 | 1 | 1 | 1 | 1 | 1 | 1 | 1 | 1 | 1 | - | 1 | 1 | 1 |
| S1057-1071 | 1057 | 1057-1071 | PHGVVFLHVTYVPAQ | VVFLHVTYV | DRB1_1201 | 1 | 1 | 1 | 1 | 1 | 1 | 1 | 1 | 1 | 1 | - | 1 | 1 | 1 |
| S1059-1073 | 1059 | 1059-1073 | GVVFLHVTYVPAQEK | FLHVTYVPA | HLA-DPA10103-DPB11011 | 1 | 1 | 1 | 1 | 1 | 1 | 1 | 1 | 1 | 1 | - | 1 | 1 | 1 |
| S1097-1111 | 1097 | 1097-1111 | SNGTHWFVTQRNFYE | THWFVTQRN | HLA-DQA10401-DQB10401 | 1 | 1 | 1 | 1 | 1 | 1 | 1 | 1 | 1 | 1 | - | 1 | 1 | 1 |
|  |  |  |  |  | HLA-DQA10402-DQB10402 |  |  |  |  |  |  |  |  |  |  |  |  |  |  |
| S1099-1113 | 1099 | 1099-1113 | GTHWFVTQRNFYEPQ | FVTQRNFYE | DRB1_0405 | 1 | 1 | 1 | 1 | 1 | 1 | 1 | 1 | 1 | 1 | - | 1 | 1 | 1 |
| S1126-1140 | 1126 | 1126-1140 | CDVVIGIVNNTVYDP | IGIVNNTVY | DRB1_1201 | 1 | 1 | 1 | 1 | 1 | 1 | 1 | 1 | 1 | 1 | - | 1 | 1 | 1 |
| S1135-1149 | 1135 | 1135-1149 | NTVYDPLQPELDSFK | YDPLQPELD | DRB1_0405 | 1 | 1 | 1 | 1 | 1 | 1 | 1 | 1 | 1 | 1 | - | 1 | 0 | 1 |
| S1150-1164 | 1150 | 1150-1164 | EELDKYFKNHTSPDV | DKYFKNHTS | HLA-DQA10401-DQB10401 | 1 | 1 | 1 | 1 | 1 | 1 | 1 | 1 | 1 | 1 | - | 1 | 1 | 1 |
|  |  |  |  |  | HLA-DQA10402-DQB10402 |  |  |  |  |  |  |  |  |  |  |  |  |  |  |
| S1151-1165 | 1151 | 1151-1165 | ELDKYFKNHTSPDVD | YFKNHTSPD | DRB1_0405 | 1 | 1 | 1 | 1 | 1 | 1 | 1 | 1 | 1 | 1 | - | 1 | 1 | 1 |

**Supplementary Table 8:** Epitope mapping of the patient’s sample sequences for CD4+ T-cell for 9-mer peptide length.

| CODE | S_POS | POS | EPITOPE | CORE | MHC | SARS_CoV_2_REF | S_N411 | S_N413 | S_N414 | S_N415 | S_N417 | S_N424 | S_N425 | S_N436 | S_N442 | S_N445 | S_N448 | S_N451 | S_N454 |
| --- | --- | --- | --- | --- | --- | --- | --- | --- | --- | --- | --- | --- | --- | --- | --- | --- | --- | --- | --- |
| S8-16 | 8 | 8-16 | LPLVSSQCV | LPLVSSQCV | HLA-B*51:01 | 1 | 1 | 1 | 1 | 1 | 1 | 1 | 1 | 1 | 0 | - | 1 | 1 | 0 |
| S17-28 | 17 | 17-28 (DELETION AT 24,25,26) | NLITRTQSY | NLITRTQSY | HLA-B*15:01 | 0 | 1 | 1 | 1 | 1 | 1 | 1 | 1 | 1 | 0 | - | 1 | 1 | 0 |
|  |  |  |  |  | HLA-B*35:01 |  |  |  |  |  |  |  |  |  |  |  |  |  |  |
|  |  |  |  |  | HLA-B*46:01 |  |  |  |  |  |  |  |  |  |  |  |  |  |  |
| S20-28 | 20 | 20-28 | TRTQLPPAY | TRTQLPPAY | HLA-C*06:02 | 1 | 0 | 0 | 0 | 0 | 0 | 0 | 0 | 0 | 0 | - | 0 | 0 | 0 |
|  |  |  |  |  | HLA-C*07:01 |  |  |  |  |  |  |  |  |  |  |  |  |  |  |
| S21-32 | 21 | 21-32 (DELETION AT 24,25,25) | RTQSYTNSF | RTQSYTNSF | HLA-B*15:01 | 0 | 1 | 1 | 1 | 1 | 1 | 1 | 1 | 1 | 0 | - | 1 | 1 | 0 |
|  |  |  |  |  | HLA-B*46:01 |  |  |  |  |  |  |  |  |  |  |  |  |  |  |
| S23-25 | 23 | 23-25 (DELETION AT 24,25,26) | QSYTNSFTR | QSYTNSFTR | HLA-A*11:01 | 1 | 1 | 1 | 1 | 1 | 1 | 1 | 1 | 1 | 0 | - | 1 | 1 | 0 |
| S24-32 | 24 | 24-32 | LPPAYTNSF | LPPAYTNSF | HLA-B*07:02 | 1 | 0 | 0 | 0 | 0 | 0 | 0 | 0 | 0 | 0 | - | 0 | 0 | 0 |
|  |  |  |  |  | HLA-B*35:01 |  |  |  |  |  |  |  |  |  |  |  |  |  |  |
|  |  |  |  |  | HLA-B*51:01 |  |  |  |  |  |  |  |  |  |  |  |  |  |  |
|  |  |  |  |  | HLA-C*01:02 |  |  |  |  |  |  |  |  |  |  |  |  |  |  |
| S28-36 | 28 | 28-36 | YTNSFTRGV | YTNSFTRGV | HLA-A*02:05 | 1 | 1 | 1 | 1 | 1 | 1 | 1 | 1 | 1 | 0 | - | 1 | 1 | 0 |
|  |  |  |  |  | HLA-A*02:06 |  |  |  |  |  |  |  |  |  |  |  |  |  |  |
|  |  |  |  |  | HLA-C*06:02 |  |  |  |  |  |  |  |  |  |  |  |  |  |  |
| S30-38 | 30 | 30-38 | NSFTRGVYY | NSFTRGVYY | HLA-B*35:01 | 1 | 1 | 1 | 1 | 1 | 1 | 1 | 1 | 1 | 0 | - | 0 | 0 | 0 |
|  |  |  |  |  | HLA-B*46:01 |  |  |  |  |  |  |  |  |  |  |  |  |  |  |
|  |  |  |  |  | HLA-C*06:02 |  |  |  |  |  |  |  |  |  |  |  |  |  |  |
|  |  |  |  |  | HLA-C*07:01 |  |  |  |  |  |  |  |  |  |  |  |  |  |  |
| S35-43 | 35 | 35-43 | GVYYPDKVF | GVYYPDKVF | HLA-B*15:01 | 1 | 1 | 1 | 1 | 1 | 1 | 1 | 1 | 1 | 0 | - | 0 | 0 | 0 |
| S40-48 | 40 | 40-48 | DKVFRSSVL | DKVFRSSVL | HLA-B*08:01 | 1 | 1 | 1 | 1 | 1 | 1 | 1 | 1 | 1 | 0 | - | 0 | 0 | 0 |
| S41-49 | 41 | 41-49 | KVFRSSVLH | KVFRSSVLH | HLA-A*11:01 | 1 | 1 | 1 | 1 | 1 | 1 | 1 | 1 | 1 | 0 | - | 0 | 0 | 0 |
| S43-51 | 43 | 43-51 | FRSSVLHST | FRSSVLHST | HLA-C*06:02 | 1 | 1 | 1 | 1 | 1 | 1 | 1 | 1 | 1 | 0 | - | 0 | 0 | 0 |
| S47-55 | 47 | 47-55 | VLHSTQDLF | VLHSTQDLF | HLA-B*15:01 | 1 | 1 | 1 | 1 | 1 | 1 | 1 | 1 | 1 | 0 | - | 0 | 0 | 0 |
| S50-58 | 50 | 50-58 | STQDLFLPF | STQDLFLPF | HLA-B*46:01 | 1 | 1 | 1 | 1 | 1 | 1 | 1 | 1 | 1 | 0 | - | 0 | 0 | 0 |
| S51-59 | 51 | 51-59 | TQDLFLPFF | TQDLFLPFF | HLA-C*04:01 | 1 | 1 | 1 | 1 | 1 | 1 | 1 | 1 | 1 | 0 | - | 0 | 0 | 0 |
| S56-64 | 56 | 56-64 | LPFFSNVTW | LPFFSNVTW | HLA-B*35:01 | 1 | 1 | 1 | 1 | 1 | 1 | 1 | 1 | 1 | 0 | - | 0 | 0 | 0 |
|  |  |  |  |  | HLA-B*51:01 |  |  |  |  |  |  |  |  |  |  |  |  |  |  |
| S57-65 | 57 | 57-65 | PFFSNVTWF | PFFSNVTWF | HLA-A*24:02 | 1 | 1 | 1 | 1 | 1 | 1 | 1 | 1 | 1 | 0 | - | 0 | 0 | 0 |
| S60-68 | 60 | 60-68 | SNVTWFHAI | SNVTWFHAI | HLA-B*52:01 | 1 | 1 | 1 | 1 | 1 | 1 | 1 | 1 | 1 | 0 | - | 0 | 0 | 0 |
| S62-70 | 62 | 62-70 | VTWFHAIHV | VTWFHAIHV | HLA-A*02:06 | 1 | 1 | 1 | 1 | 1 | 1 | 1 | 1 | 1 | 0 | - | 0 | 0 | 0 |
| S78-86 | 78 | 78-86 | RFDNPVLPF | RFDNPVLPF | HLA-A*24:02 | 1 | 1 | 1 | 0 | 0 | 1 | 0 | 1 | 0 | 0 | - | 0 | 0 | 0 |
|  |  |  |  |  | HLA-C*04:01 |  |  |  |  |  |  |  |  |  |  |  |  |  |  |
| S84-92 | 84 | 84-92 | LPFNDGVYF | LPFNDGVYF | HLA-B*35:01 | 1 | 1 | 1 | 0 | 0 | 1 | 0 | 1 | 0 | 0 | - | 0 | 0 | 0 |
|  |  |  |  |  | HLA-B*51:01 |  |  |  |  |  |  |  |  |  |  |  |  |  |  |
| S89-97 | 89 | 89-97 | GVYFASTEK | GVYFASTEK | HLA-A*11:01 | 1 | 1 | 1 | 0 | 0 | 1 | 1 | 1 | 1 | 0 | - | 0 | 0 | 0 |
| S109-117 | 109 | 109-117 | TLDSKTQSL | TLDSKTQSL | HLA-A*02:01 | 1 | 1 | 1 | 1 | 1 | 1 | 1 | 1 | 1 | 1 | - | 1 | 1 | 0 |
|  |  |  |  |  | HLA-A*02:03 |  |  |  |  |  |  |  |  |  |  |  |  |  |  |
|  |  |  |  |  | HLA-A*02:05 |  |  |  |  |  |  |  |  |  |  |  |  |  |  |
|  |  |  |  |  | HLA-A*02:06 |  |  |  |  |  |  |  |  |  |  |  |  |  |  |
|  |  |  |  |  | HLA-B*08:01 |  |  |  |  |  |  |  |  |  |  |  |  |  |  |
|  |  |  |  |  | HLA-C*01:02 |  |  |  |  |  |  |  |  |  |  |  |  |  |  |
|  |  |  |  |  | HLA-C*04:01 |  |  |  |  |  |  |  |  |  |  |  |  |  |  |
| S111-119 | 111 | 111-119 | DSKTQSLLI | DSKTQSLLI | HLA-B*51:01 | 1 | 1 | 1 | 1 | 1 | 1 | 1 | 1 | 1 | 1 | - | 1 | 1 | 0 |
| S122-130 | 122 | 122-130 | NATNVVIKV | NATNVVIKV | HLA-B*51:01 | 1 | 1 | 1 | 1 | 1 | 1 | 1 | 1 | 1 | 1 | - | 1 | 1 | 0 |
|  |  |  |  |  | HLA-B*52:01 |  |  |  |  |  |  |  |  |  |  |  |  |  |  |
| S133-141 | 133 | 133-141 | FQFCNDPFL | FQFCNDPFL | HLA-A*02:05 | 1 | 0 | 1 | 1 | 1 | 1 | 1 | 1 | 1 | 1 | - | 1 | 1 | 0 |
|  |  |  |  |  | HLA-A*02:06 |  |  |  |  |  |  |  |  |  |  |  |  |  |  |
| S135-143 | 135 | 135-143 | FCNDPFLGV | FCNDPFLGV | HLA-A*02:06 | 1 | 0 | 0 | 0 | 0 | 0 | 0 | 0 | 0 | 0 | - | 0 | 0 | 0 |
| S142-150 | 142 | 142-150 | GVYYHKNNK | GVYYHKNNK | HLA-A*11:01 | 1 | 0 | 0 | 0 | 0 | 0 | 0 | 0 | 0 | 0 | - | 0 | 0 | 0 |
| S143-152 | 143 | 143-152 (DELETION AT 145) | VY**K**KNNKSW | VYKKNNKSW | HLA-A*24:02 | 0 | 0 | 0 | 1 | 0 | 0 | 0 | 0 | 0 | 0 | - | 0 | 0 | 0 |
|  |  |  | VY**Q**KNNKSW | VYQKNNKSW | HLA-A*24:02 | 0 | 0 | 1 | 0 | 1 | 1 | 1 | 1 | 1 | 1 | - | 1 | 0 | 0 |
| S144-152 | 144 | 144-152 | YYHKNNKSW | YYHKNNKSW | HLA-A*24:02 | 1 | 0 | 0 | 0 | 0 | 0 | 0 | 0 | 0 | 0 | - | 0 | 0 | 0 |
| S152-160 | 152 | 152-160 | WMESEFRVY | WMESEFRVY | HLA-B*15:01 | 1 | 1 | 1 | 1 | 1 | 1 | 1 | 1 | 1 | 1 | - | 1 | 0 | 0 |
|  |  |  |  |  | HLA-B*46:01 |  |  |  |  |  |  |  |  |  |  |  |  |  |  |
| S155-163 | 155 | 155-163 | SEFRVYSSA | SEFRVYSSA | HLA-B*18:01 | 1 | 1 | 1 | 1 | 1 | 1 | 1 | 1 | 1 | 1 | - | 1 | 0 | 0 |
|  |  |  |  |  | HLA-B*40:06 |  |  |  |  |  |  |  |  |  |  |  |  |  |  |
| S160-168 | 160 | 160-168 | YSSANNCTF | YSSANNCTF | HLA-B*46:01 | 1 | 1 | 1 | 1 | 1 | 1 | 1 | 1 | 1 | 0 | - | 0 | 0 | 0 |
| S162-170 | 162 | 162-170 | SANNCTFEY | SANNCTFEY | HLA-B*35:01 | 1 | 1 | 1 | 1 | 1 | 1 | 1 | 1 | 1 | 0 | - | 0 | 0 | 0 |
|  |  |  |  |  | HLA-B*46:01 |  |  |  |  |  |  |  |  |  |  |  |  |  |  |
| S167-175 | 167 | 167-175 | TFEYVSQPF | TFEYVSQPF | HLA-C*04:01 | 1 | 1 | 1 | 1 | 1 | 1 | 1 | 1 | 1 | 0 | - | 0 | 0 | 0 |
| S168-176 | 168 | 168-176 | FEYVSQPFL | FEYVSQPFL | HLA-B*18:01 | 1 | 1 | 1 | 1 | 1 | 1 | 1 | 1 | 1 | 0 | - | 0 | 0 | 0 |
|  |  |  |  |  | HLA-B*40:06 |  |  |  |  |  |  |  |  |  |  |  |  |  |  |
|  |  |  |  |  | HLA-B*52:01 |  |  |  |  |  |  |  |  |  |  |  |  |  |  |
| S169-177 | 169 | 169-177 | EYVSQPFLM | EYVSQPFLM | HLA-A*24:02 | 1 | 1 | 1 | 1 | 1 | 1 | 1 | 1 | 1 | 0 | - | 0 | 0 | 0 |
| S192-200 | 192 | 192-200 | FVFKNIDGY | FVFKNIDGY | HLA-B*15:01 | 1 | 1 | 1 | 1 | 1 | 1 | 1 | 1 | 1 | 1 | - | 1 | 1 | 0 |
|  |  |  |  |  | HLA-B*35:01 |  |  |  |  |  |  |  |  |  |  |  |  |  |  |
|  |  |  |  |  | HLA-B*46:01 |  |  |  |  |  |  |  |  |  |  |  |  |  |  |
| S193-201 | 193 | 193-201 | VFKNIDGYF | VFKNIDGYF | HLA-A*24:02 | 1 | 1 | 1 | 1 | 1 | 1 | 1 | 1 | 1 | 1 | - | 1 | 1 | 0 |
| S195-203 | 195 | 195-203 | KNIDGYFKI | KNIDGYFKI | HLA-B*13:02 | 1 | 1 | 1 | 1 | 1 | 1 | 1 | 1 | 1 | 1 | - | 1 | 1 | 0 |
|  |  |  |  |  | HLA-B*52:01 |  | 1 | 1 | 1 | 1 | 1 | 1 | 1 | 1 |  |  |  |  |  |
| S202-210 | 202 | 202-210 | KIYSKHTPI | KIYSKHTPI | HLA-A*02:03 | 1 | 1 | 1 | 1 | 1 | 1 | 1 | 1 | 1 | 1 | - | 1 | 1 | 0 |
|  |  |  |  |  | HLA-B*08:01 |  |  |  |  |  |  |  |  |  |  |  |  |  |  |
|  |  |  |  |  | HLA-B*13:02 |  |  |  |  |  |  |  |  |  |  |  |  |  |  |
| S204-212 | 204 | 204-212 | YSKHTPINL | YSKHTPINL | HLA-B*46:01 | 1 | 1 | 1 | 1 | 1 | 1 | 1 | 1 | 1 | 1 | - | 1 | 1 | 0 |
|  |  |  |  |  | HLA-C*01:02 |  |  |  |  |  |  |  |  |  |  |  |  |  |  |
|  |  |  |  |  | HLA-C*06:02 |  |  |  |  |  |  |  |  |  |  |  |  |  |  |
|  |  |  |  |  | HLA-C*07:01 |  |  |  |  |  |  |  |  |  |  |  |  |  |  |
| S205-213 | 205 | 205-213 | SKHTPINLV | SKHTPINLV | HLA-B*52:01 | 1 | 0 | 0 | 0 | 0 | 0 | 0 | 0 | 0 | 0 | - | 0 | 0 | 0 |
|  |  |  |  |  | HLA-C*06:02 |  |  |  |  |  |  |  |  |  |  |  |  |  |  |
| S208-216 | 208 | 208-216 | TPINLVRDL | TPINLVRDL | HLA-B*07:02 | 1 | 0 | 0 | 0 | 0 | 0 | 0 | 0 | 0 | 0 | - | 0 | 0 | 0 |
| S212-220 | 212 | 212-220 | L**E**RDLPQGF | LERDLPQGF | HLA-B*18:01 | 0 | 1 | 1 | 1 | 1 | 1 | 1 | 1 | 1 | 1 | - | 1 | 1 | 0 |
|  |  |  | L**V**RDLPQGF | LVRDLPQGF | HLA-B*15:01 | 1 | 0 | 0 | 0 | 0 | 0 | 0 | 0 | 0 | 0 | - | 0 | 0 | 0 |
|  |  |  |  |  | HLA-B*46:01 |  |  |  |  |  |  |  |  |  |  |  |  |  |  |
| S214-222 | 214 | 214-222 | RDLPQGFSA | RDLPQGFSA | HLA-B*40:06 | 1 | 1 | 1 | 1 | 1 | 1 | 1 | 1 | 1 | 1 | - | 1 | 1 | 0 |
| S215-223 | 215 | 215-223 | DLPQGFSAL | DLPQGFSAL | HLA-C*01:02 | 1 | 1 | 1 | 1 | 1 | 1 | 1 | 1 | 1 | 1 | - | 1 | 1 | 0 |
| S227-235 | 227 | 227-235 | VDLPIGINI | VDLPIGINI | HLA-B*52:01 | 1 | 1 | 1 | 1 | 1 | 1 | 1 | 1 | 1 | 1 | - | 1 | 1 | 1 |
| S233-241 | 233 | 233-241 | INITRFQTL | INITRFQTL | HLA-B*08:01 | 1 | 1 | 1 | 1 | 1 | 1 | 1 | 1 | 1 | 1 | - | 1 | 1 | 1 |
|  |  |  |  |  | HLA-B*52:01 |  |  |  |  |  |  |  |  |  |  |  |  |  |  |
| S236-244 | 236 | 236-244 | TRFQTLLAL | TRFQTLLAL | HLA-C*06:02 | 1 | 0 | 1 | 1 | 1 | 1 | 1 | 1 | 1 | 1 | - | 1 | 1 | 1 |
|  |  |  |  |  | HLA-C*07:01 |  |  |  |  |  |  |  |  |  |  |  |  |  |  |
| S238-248 | 238 | 238-248 (DELETION 243,244) | FQTLLHRSY | FQTLLHRSY | HLA-B*15:01 | 0 | 1 | 0 | 0 | 0 | 0 | 0 | 0 | 0 | 0 | - | 0 | 0 | 0 |
|  |  |  |  |  | HLA-B*18:01 |  |  |  |  |  |  |  |  |  |  |  |  |  |  |
|  |  |  |  |  | HLA-B*46:01 |  |  |  |  |  |  |  |  |  |  |  |  |  |  |
| S240-248 | 240 | 240-248 | TLLALHRSY | TLLALHRSY | HLA-B*15:01 | 1 | 0 | 1 | 1 | 1 | 1 | 1 | 1 | 1 | 1 | - | 1 | 1 | 1 |
|  |  |  |  |  | HLA-B*46:01 |  |  |  |  |  |  |  |  |  |  |  |  |  |  |
| S241-249 | 241 | 241-249 | LLALHRSYL | LLALHRSYL | HLA-A*02:03 | 1 | 0 | 1 | 1 | 1 | 1 | 1 | 1 | 1 | 1 | - | 1 | 1 | 1 |
|  |  |  |  |  | HLA-B*08:01 |  |  |  |  |  |  |  |  |  |  |  |  |  |  |
| S250-258 | 250 | 250-258 | TPVDSSSGW | TPVDSSSGW | HLA-B*35:01 | 0 | 0 | 1 | 0 | 0 | 1 | 0 | 0 | 1 | 0 | - | 0 | 0 | 1 |
| S267-275 | 267 | 267-275 | VGYLQPRTF | VGYLQPRTF | HLA-B*46:01 | 1 | 1 | 1 | 1 | 1 | 1 | 1 | 1 | 1 | 1 | - | 1 | 1 | 1 |
|  |  |  |  |  | HLA-B*52:01 |  |  |  |  |  |  |  |  |  |  |  |  |  |  |
|  |  |  |  |  | HLA-C*06:02 |  |  |  |  |  |  |  |  |  |  |  |  |  |  |
| S268-276 | 268 | 268-276 | GYLQPRTFL | GYLQPRTFL | HLA-A*24:02 | 1 | 1 | 1 | 1 | 1 | 1 | 1 | 1 | 1 | 1 | - | 1 | 1 | 1 |
| S269-277 | 269 | 269-277 | YLQPRTFLL | YLQPRTFLL | HLA-A*02:01 | 1 | 1 | 1 | 1 | 1 | 1 | 1 | 1 | 1 | 1 | - | 1 | 1 | 1 |
|  |  |  |  |  | HLA-A*02:03 |  |  |  |  |  |  |  |  |  |  |  |  |  |  |
|  |  |  |  |  | HLA-A*02:05 |  |  |  |  |  |  |  |  |  |  |  |  |  |  |
|  |  |  |  |  | HLA-A*02:06 |  |  |  |  |  |  |  |  |  |  |  |  |  |  |
|  |  |  |  |  | HLA-B*08:01 |  |  |  |  |  |  |  |  |  |  |  |  |  |  |
|  |  |  |  |  | HLA-B*13:02 |  |  |  |  |  |  |  |  |  |  |  |  |  |  |
|  |  |  |  |  | HLA-C*01:02 |  |  |  |  |  |  |  |  |  |  |  |  |  |  |
|  |  |  |  |  | HLA-C*04:01 |  |  |  |  |  |  |  |  |  |  |  |  |  |  |
|  |  |  |  |  | HLA-C*06:02 |  |  |  |  |  |  |  |  |  |  |  |  |  |  |
|  |  |  |  |  | HLA-C*07:01 |  |  |  |  |  |  |  |  |  |  |  |  |  |  |
| S271-279 | 271 | 271-279 | QPRTFLLKY | QPRTFLLKY | HLA-B*35:01 | 1 | 1 | 1 | 1 | 1 | 1 | 1 | 1 | 1 | 1 | - | 1 | 1 | 1 |
| S280-288 | 280 | 280-288 | NENGTITDA | NENGTITDA | HLA-B*40:06 | 1 | 1 | 1 | 1 | 1 | 1 | 1 | 1 | 1 | 1 | - | 1 | 1 | 1 |
| S285-293 | 285 | 285-293 | ITDAVGCAL | ITDAVGCAL | HLA-C*04:01 | 0 | 0 | 0 | 0 | 0 | 0 | 0 | 0 | 0 | 1 | - | 0 | 0 | 0 |
| S292-300 | 292 | 292-300 | ALDPLSETK | ALDPLSETK | HLA-A*11:01 | 1 | 1 | 1 | 1 | 1 | 1 | 1 | 1 | 1 | 1 | - | 1 | 0 | 0 |
| S302-310 | 302 | 302-310 | TLKSFTVEK | TLKSFTVEK | HLA-A*11:01 | 1 | 1 | 1 | 1 | 1 | 1 | 1 | 1 | 1 | 0 | - | 1 | 0 | 0 |
| S308-316 | 308 | 308-316 | VEKGIYQTS | VEKGIYQTS | HLA-B*40:06 | 1 | 1 | 1 | 1 | 1 | 1 | 1 | 1 | 1 | 0 | - | 1 | 0 | 0 |
| S311-319 | 311 | 311-319 | GIYQTSNFR | GIYQTSNFR | HLA-A*11:01 | 1 | 1 | 1 | 1 | 1 | 1 | 1 | 1 | 1 | 0 | - | 1 | 1 | 0 |
| S312-320 | 312 | 312-320 | IYQTSNFRV | IYQTSNFRV | HLA-A*24:02 | 1 | 1 | 1 | 1 | 1 | 1 | 1 | 1 | 1 | 0 | - | 1 | 1 | 0 |
| S318-326 | 318 | 318-326 | FRVQP**I**ESI | FRVQPIESI | HLA-C*06:02 | 0 | 0 | 0 | 1 | 0 | 0 | 0 | 0 | 0 | 0 | - | 0 | 0 | 0 |
|  |  |  |  |  | HLA-C*07:01 |  |  |  |  |  |  |  |  |  |  |  |  |  |  |
|  |  |  | FRVQP**T**ESI | FRVQPTESI | HLA-C*06:02 | 1 | 1 | 1 | 0 | 1 | 1 | 1 | 1 | 1 | 0 | - | 1 | 1 | 0 |
|  |  |  |  |  | HLA-C*07:01 |  |  |  |  |  |  |  |  |  |  |  |  |  |  |
| S319-327 | 319 | 319-327 | RVQP**I**ESIV | RVQPIESIV | HLA-B*13:02 | 0 | 0 | 0 | 1 | 0 | 0 | 0 | 0 | 0 | 0 | - | 0 | 0 | 0 |
|  |  |  | RVQP**T**ESIV | RVQPTESIV | HLA-B*13:02 | 1 | 1 | 1 | 0 | 1 | 1 | 1 | 1 | 1 | 0 | - | 1 | 1 | 0 |
| S321-329 | 321 | 321-329 | QP**I**ESIVRF | QPIESIVRF | HLA-B*07:02 | 0 | 0 | 0 | 1 | 0 | 0 | 0 | 0 | 0 | 0 | - | 0 | 0 | 0 |
|  |  |  |  |  | HLA-B*35:01 |  |  |  |  |  |  |  |  |  |  |  |  |  |  |
|  |  |  |  |  | HLA-B*51:01 |  |  |  |  |  |  |  |  |  |  |  |  |  |  |
|  |  |  |  |  | HLA-C*04:01 |  |  |  |  |  |  |  |  |  |  |  |  |  |  |
|  |  |  | QP**T**ESIVRF | QPTESIVRF | HLA-B*07:02 | 1 | 1 | 1 | 0 | 1 | 1 | 1 | 1 | 1 | 0 | - | 1 | 1 | 0 |
|  |  |  |  |  | HLA-B*35:01 |  |  |  |  |  |  |  |  |  |  |  |  |  |  |
|  |  |  |  |  | HLA-B*51:01 |  |  |  |  |  |  |  |  |  |  |  |  |  |  |
|  |  |  |  |  | HLA-C*04:01 |  |  |  |  |  |  |  |  |  |  |  |  |  |  |
| S327-335 | 327 | 327-335 | VRFPNITNL | VRFPNITNL | HLA-B*52:01 | 1 | 1 | 1 | 1 | 1 | 1 | 1 | 1 | 1 | 0 | - | 1 | 1 | 0 |
|  |  |  |  |  | HLA-C*06:02 |  |  |  |  |  |  |  |  |  |  |  |  |  |  |
|  |  |  |  |  | HLA-C*07:01 |  |  |  |  |  |  |  |  |  |  |  |  |  |  |
| S339-347 | 339 | 339-347 | **H**EVFNAT**T**F | HEVFNATTF | HLA-B*18:01 | 0 | 1 | 1 | 1 | 1 | 1 | 1 | 1 | 1 | 0 | - | 1 | 1 | 0 |
|  |  |  | **G**EVFNAT**R**F | GEVFNATRF | HLA-B*18:01 | 1 | 0 | 0 | 0 | 0 | 0 | 0 | 0 | 0 | 0 | - | 0 | 0 | 0 |
| S342-350 | 342 | 342-350 | FNATRFASV | FNATRFASV | HLA-B*08:01 | 1 | 0 | 0 | 0 | 0 | 0 | 0 | 0 | 0 | 0 | - | 0 | 0 | 0 |
| S343-351 | 343 | 343-351 | NAT**T**FASVY | NATTFASVY | HLA-B*35:01 | 0 | 1 | 1 | 1 | 1 | 1 | 1 | 1 | 1 | 0 | - | 1 | 1 | 0 |
|  |  |  |  |  | HLA-B*46:01 |  |  |  |  |  |  |  |  |  |  |  |  |  |  |
|  |  |  | NAT**R**FASVY | NATRFASVY | HLA-B*35:01 | 1 | 0 | 0 | 0 | 0 | 0 | 0 | 0 | 0 | 0 | - | 0 | 0 | 0 |
|  |  |  |  |  | HLA-B*46:01 |  |  |  |  |  |  |  |  |  |  |  |  |  |  |
| S345-353 | 345 | 345-353 | TRFASVYAW | TRFASVYAW | HLA-C*06:02 | 1 | 0 | 0 | 0 | 0 | 0 | 0 | 0 | 0 | 0 | - | 0 | 0 | 0 |
|  |  |  |  |  | HLA-C*07:01 |  |  |  |  |  |  |  |  |  |  |  |  |  |  |
| S348-356 | 348 | 348-356 | ASVYAWNRK | ASVYAWNRK | HLA-A*11:01 | 1 | 1 | 1 | 1 | 1 | 1 | 1 | 1 | 1 | 0 | - | 1 | 1 | 0 |
| S349-357 | 349 | 349-357 | SVYAWNRKR | SVYAWNRKR | HLA-A*11:01 | 1 | 1 | 1 | 1 | 1 | 1 | 1 | 1 | 1 | 0 | - | 1 | 1 | 0 |
| S361-369 | 361 | 361-369 | CVADYSV**I**Y | CVADYSVIY | HLA-B*15:01 | 0 | 1 | 1 | 1 | 1 | 1 | 1 | 1 | 1 | 0 | - | 1 | 1 | 1 |
|  |  |  |  |  | HLA-B*35:01 |  |  |  |  |  |  |  |  |  |  |  |  |  |  |
|  |  |  |  |  | HLA-B*46:01 |  |  |  |  |  |  |  |  |  |  |  |  |  |  |
|  |  |  | CVADYSV**L**Y | CVADYSVLY | HLA-B*35:01 | 1 | 0 | 0 | 0 | 0 | 0 | 0 | 0 | 0 | 0 | - | 0 | 0 | 0 |
|  |  |  |  |  | HLA-B*46:01 |  |  |  |  |  |  |  |  |  |  |  |  |  |  |
| S366-374 | 366 | 366-374 | SV**I**YNFA**P**F | SVIYNFAPF | HLA-B*46:01 | 0 | 1 | 1 | 1 | 1 | 1 | 1 | 1 | 1 | 0 | - | 1 | 1 | 1 |
|  |  |  | SV**L**YNSA**S**F | SVLYNSASF | HLA-B*46:01 | 1 | 0 | 0 | 0 | 0 | 0 | 0 | 0 | 0 | 0 | - | 0 | 0 | 0 |
| S367-375 | 367 | 367-375 | VIYNFAPFF | VIYNFAPFF | HLA-B*46:01 | 0 | 1 | 1 | 1 | 1 | 1 | 1 | 1 | 1 | 0 | - | 1 | 1 | 1 |
| S370-378 | 370 | 370-378 | NSASFSTFK | NSASFSTFK | HLA-A*11:01 | 1 | 0 | 0 | 0 | 0 | 0 | 0 | 0 | 0 | 0 | - | 0 | 0 | 0 |
| S372-380 | 372 | 372-380 | A**P**F**FA**FKCY | APFFAFKCY | HLA-B*35:01 | 0 | 1 | 1 | 1 | 1 | 1 | 1 | 1 | 1 | 0 | - | 1 | 1 | 1 |
|  |  |  | A**S**F**ST**FKCY | ASFSTFKCY | HLA-B*15:01 | 1 | 0 | 0 | 0 | 0 | 0 | 0 | 0 | 0 | 0 | - | 0 | 0 | 0 |
|  |  |  |  |  | HLA-B*46:01 |  |  |  |  |  |  |  |  |  |  |  |  |  |  |
| S379-387 | 379 | 379-387 | CYGVSP**I**KL | CYGVSPIKL | HLA-A*24:02 | 0 | 1 | 0 | 0 | 0 | 0 | 0 | 0 | 0 | 0 | - | 0 | 0 | 0 |
|  |  |  | CYGVSP**T**KL | CYGVSPTKL | HLA-A*24:02 | 1 | 0 | 1 | 1 | 1 | 1 | 1 | 1 | 1 | 0 | - | 1 | 1 | 1 |
| S382-390 | 382 | 382-390 | VSP**I**KLNDL | VSPIKLNDL | HLA-C*01:02 | 0 | 1 | 0 | 0 | 0 | 0 | 0 | 0 | 0 | 0 | - | 0 | 0 | 0 |
|  |  |  | VSP**T**KLNDL | VSPTKLNDL | HLA-C*01:02 | 1 | 0 | 1 | 1 | 1 | 1 | 1 | 1 | 1 | 0 | - | 1 | 1 | 1 |
| S388-396 | 388 | 388-396 | NDLCFTNVY | NDLCFTNVY | HLA-B*18:01 | 1 | 1 | 1 | 1 | 1 | 1 | 1 | 1 | 1 | 0 | - | 1 | 1 | 1 |
| S392-400 | 392 | 392-400 | FTNVYADSF | FTNVYADSF | HLA-B*46:01 | 1 | 1 | 1 | 1 | 1 | 1 | 1 | 1 | 1 | 0 | - | 1 | 1 | 1 |
| S402-410 | 402 | 402-410 | IRG**N**EV**S**QI | IRGNEVSQI | HLA-C*06:02 | 0 | 1 | 1 | 1 | 1 | 1 | 1 | 1 | 1 | 0 | - | 1 | 1 | 1 |
|  |  |  | IRG**D**EV**R**QI | IRGDEVRQI | HLA-C*06:02 | 1 | 0 | 0 | 0 | 0 | 0 | 0 | 0 | 0 | 0 | - | 0 | 0 | 0 |
|  |  |  |  |  | HLA-C*07:01 |  |  |  |  |  |  |  |  |  |  |  |  |  |  |
| S409-417 | 409 | 409-417 | QIAPGQTGK | QIAPGQTGK | HLA-A*11:01 | 1 | 0 | 0 | 0 | 0 | 0 | 0 | 0 | 0 | 0 | - | 0 | 0 | 0 |
| S410-418 | 410 | 410-418 | IAPGQTG**N**I | IAPGQTGNI | HLA-C*01:02 | 0 | 1 | 1 | 1 | 1 | 1 | 1 | 1 | 1 | 0 | - | 1 | 1 | 1 |
|  |  |  | IAPGQTG**K**I | IAPGQTGKI | HLA-C*01:02 | 1 | 0 | 0 | 0 | 0 | 0 | 0 | 0 | 0 | 0 | - | 0 | 0 | 0 |
| S413-421 | 413 | 413-421 | GQTG**N**IADY | GQTGNIADY | HLA-B*15:01 | 0 | 1 | 1 | 1 | 1 | 1 | 1 | 1 | 1 | 0 | - | 1 | 1 | 1 |
|  |  |  | GQTG**K**IADY | GQTGKIADY | HLA-B*15:01 | 1 | 0 | 0 | 0 | 0 | 0 | 0 | 0 | 0 | 0 | - | 0 | 0 | 0 |
| S417-425 | 417 | 417-425 | **N**IADYNYKL | NIADYNYKL | HLA-A*02:01 | 0 | 1 | 1 | 1 | 1 | 1 | 1 | 1 | 1 | 0 | - | 1 | 1 | 1 |
|  |  |  |  |  | HLA-A*02:05 |  |  |  |  |  |  |  |  |  |  |  |  |  |  |
|  |  |  |  |  | HLA-A*02:06 |  |  |  |  |  |  |  |  |  |  |  |  |  |  |
|  |  |  | **K**IADYNYKL | KIADYNYKL | HLA-A*02:01 | 1 | 0 | 0 | 0 | 0 | 0 | 0 | 0 | 0 | 0 | - | 0 | 0 | 0 |
|  |  |  |  |  | HLA-A*02:03 |  |  |  |  |  |  |  |  |  |  |  |  |  |  |
|  |  |  |  |  | HLA-A*02:05 |  |  |  |  |  |  |  |  |  |  |  |  |  |  |
|  |  |  |  |  | HLA-A*02:06 |  |  |  |  |  |  |  |  |  |  |  |  |  |  |
|  |  |  |  |  | HLA-B*13:02 |  |  |  |  |  |  |  |  |  |  |  |  |  |  |
|  |  |  |  |  | HLA-C*04:01 |  |  |  |  |  |  |  |  |  |  |  |  |  |  |
| S424-432 | 424 | 424-432 | KLPDDFTGC | KLPDDFTGC | HLA-A*02:06 | 1 | 1 | 1 | 1 | 1 | 1 | 1 | 1 | 1 | 0 | - | 1 | 1 | 1 |
| S432-440 | 432 | 432-440 | CVIAWNSNK | CVIAWNSNK | HLA-A*11:01 | 0 | 1 | 1 | 1 | 1 | 1 | 1 | 1 | 1 | 0 | - | 1 | 1 | 1 |
| S433-441 | 433 | 433-441 | VIAWNSN**K**L | VIAWNSNKL | HLA-C*01:02 | 0 | 1 | 1 | 1 | 1 | 1 | 1 | 1 | 1 | 0 | - | 1 | 1 | 1 |
|  |  |  | VIAWNSN**N**L | VIAWNSNNL | HLA-C*01:02 | 1 | 0 | 0 | 0 | 0 | 0 | 0 | 0 | 0 | 0 | - | 0 | 0 | 0 |
| S444-452 | 444 | 444-452 | KPSGNYNYL | KPSGNYNYL | HLA-B*07:02 | 0 | 1 | 1 | 1 | 1 | 1 | 1 | 1 | 1 | 0 | - | 1 | 1 | 1 |
| S448-456 | 448 | 448-456 | NYNYLYRLF | NYNYLYRLF | HLA-A*24:02 | 1 | 1 | 1 | 1 | 1 | 1 | 1 | 1 | 1 | 0 | - | 1 | 1 | 1 |
|  |  |  |  |  | HLA-C*07:01 |  |  |  |  |  |  |  |  |  |  |  |  |  |  |
| S453-461 | 453 | 453-461 | YRLFRKS**K**L | YRLFRKSKL | HLA-B*08:01 | 0 | 1 | 1 | 1 | 1 | 1 | 1 | 1 | 1 | 0 | - | 1 | 1 | 0 |
|  |  |  |  |  | HLA-C*06:02 |  |  |  |  |  |  |  |  |  |  |  |  |  |  |
|  |  |  |  |  | HLA-C*07:01 |  |  |  |  |  |  |  |  |  |  |  |  |  |  |
|  |  |  | YRLFRKS**N**L | YRLFRKSNL | HLA-B*08:01 | 1 | 0 | 0 | 0 | 0 | 0 | 0 | 0 | 0 | 0 | - | 0 | 0 | 0 |
|  |  |  |  |  | HLA-C*06:02 |  |  |  |  |  |  |  |  |  |  |  |  |  |  |
|  |  |  |  |  | HLA-C*07:01 |  |  |  |  |  |  |  |  |  |  |  |  |  |  |
| S454-462 | 454 | 454-462 | RLFRKS**K**LK | RLFRKSKLK | HLA-A*11:01 | 0 | 1 | 1 | 1 | 1 | 1 | 1 | 1 | 1 | 0 | - | 1 | 1 | 0 |
|  |  |  | RLFRKS**N**LK | RLFRKSNLK | HLA-A*11:01 | 1 | 0 | 0 | 0 | 0 | 0 | 0 | 0 | 0 | 0 | - | 0 | 0 | 0 |
| S456-464 | 456 | 456-464 | FRKS**K**LKPF | FRKSKLKPF | HLA-B*08:01 | 0 | 1 | 1 | 1 | 1 | 1 | 1 | 1 | 1 | 0 | - | 1 | 1 | 0 |
|  |  |  |  |  | HLA-C*06:02 |  |  |  |  |  |  |  |  |  |  |  |  |  |  |
|  |  |  |  |  | HLA-C*07:01 |  |  |  |  |  |  |  |  |  |  |  |  |  |  |
|  |  |  | FRKS**N**LKPF | FRKSNLKPF | HLA-C*06:02 | 1 | 0 | 0 | 0 | 0 | 0 | 0 | 0 | 0 | 0 | - | 0 | 0 | 0 |
|  |  |  |  |  | HLA-C*07:01 |  |  |  |  |  |  |  |  |  |  |  |  |  |  |
| S460-468 | 460 | 460-468 | KLKPFERDI | KLKPFERDI | HLA-B*13:02 | 0 | 1 | 1 | 1 | 1 | 1 | 1 | 1 | 1 | 0 | - | 1 | 0 | 0 |
| S464-472 | 464 | 464-472 | FERDISTEI | FERDISTEI | HLA-B*13:02 | 1 | 1 | 1 | 1 | 1 | 1 | 1 | 1 | 1 | 0 | - | 1 | 0 | 0 |
|  |  |  |  |  | HLA-B*18:01 |  |  |  |  |  |  |  |  |  |  |  |  |  |  |
|  |  |  |  |  | HLA-B*40:06 |  |  |  |  |  |  |  |  |  |  |  |  |  |  |
|  |  |  |  |  | HLA-B*52:01 |  |  |  |  |  |  |  |  |  |  |  |  |  |  |
| S478-486 | 478 | 478-486 | TPCNGVEGF | TPCNGVEGF | HLA-B*35:01 | 1 | 0 | 0 | 0 | 0 | 0 | 0 | 0 | 0 | 0 | - | 0 | 0 | 0 |
| S484-492 | 484 | 484-492 | AGPNCYSPL | AGPNCYSPL | HLA-C*01:02 | 0 | 1 | 1 | 1 | 1 | 1 | 1 | 1 | 1 | 0 | - | 1 | 0 | 0 |
| S487-495 | 487 | 487-495 | NCY**S**PLQSY | NCYSPLQSY | HLA-B*35:01 | 0 | 1 | 1 | 1 | 1 | 1 | 1 | 1 | 1 | 0 | - | 1 | 0 | 0 |
|  |  |  |  |  | HLA-B*46:01 |  |  |  |  |  |  |  |  |  |  |  |  |  |  |
|  |  |  | NCY**F**PLQSY | NCYFPLQSY | HLA-B*35:01 | 1 | 0 | 0 | 0 | 0 | 0 | 0 | 0 | 0 | 0 | - | 0 | 0 | 0 |
|  |  |  |  |  | HLA-B*46:01 |  |  |  |  |  |  |  |  |  |  |  |  |  |  |
| S489-497 | 489 | 489-497 | Y**S**PLQSYGF | YSPLQSYGF | HLA-C*01:02 | 0 | 1 | 1 | 1 | 1 | 1 | 1 | 1 | 1 | 0 | - | 1 | 0 | 0 |
|  |  |  | Y**F**PLQSYGF | YFPLQSYGF | HLA-A*24:02 | 1 | 0 | 0 | 0 | 0 | 0 | 0 | 0 | 0 | 0 | - | 0 | 0 | 0 |
|  |  |  |  |  | HLA-C*04:01 |  |  |  |  |  |  |  |  |  |  |  |  |  |  |
| S493-501 | 493 | 493-501 | QSYGFRPTY | QSYGFRPTY | HLA-B*15:01 | 0 | 1 | 1 | 1 | 1 | 1 | 1 | 1 | 1 | 0 | - | 0 | 0 | 0 |
|  |  |  |  |  | HLA-B*35:01 |  |  |  |  |  |  |  |  |  |  |  |  |  |  |
|  |  |  |  |  | HLA-B*46:01 |  |  |  |  |  |  |  |  |  |  |  |  |  |  |
|  |  |  |  |  | HLA-C*06:02 |  |  |  |  |  |  |  |  |  |  |  |  |  |  |
|  |  |  |  |  | HLA-C*07:01 |  |  |  |  |  |  |  |  |  |  |  |  |  |  |
| S495-503 | 495 | 495-503 | YGFQPTNGV | YGFQPTNGV | HLA-B*52:01 | 1 | 0 | 0 | 0 | 0 | 0 | 0 | 0 | 0 | 0 | - | 0 | 0 | 0 |
| S497-505 | 497 | 497-505 | F**R**PT**Y**GVGY | FRPTYGVGY | HLA-C*06:02 | 0 | 1 | 1 | 1 | 1 | 1 | 1 | 1 | 1 | 0 | - | 0 | 0 | 0 |
|  |  |  |  |  | HLA-C*07:01 |  |  |  |  |  |  |  |  |  |  |  |  |  |  |
|  |  |  | F**Q**PT**N**GVGY | FQPTNGVGY | HLA-B*15:01 | 1 | 0 | 0 | 0 | 0 | 0 | 0 | 0 | 0 | 0 | - | 0 | 0 | 0 |
|  |  |  |  |  | HLA-B*46:01 |  |  |  |  |  |  |  |  |  |  |  |  |  |  |
| S503-511 | 503 | 503-511 | VGYQPYRVV | VGYQPYRVV | HLA-B*52:01 | 1 | 1 | 1 | 1 | 1 | 1 | 1 | 1 | 1 | 0 | - | 0 | 0 | 0 |
|  |  |  |  |  | HLA-C*06:02 |  |  |  |  |  |  |  |  |  |  |  |  |  |  |
| S505-513 | 505 | 505-513 | YQPYRVVVL | YQPYRVVVL | HLA-A*02:05 | 1 | 1 | 1 | 1 | 1 | 1 | 1 | 1 | 1 | 0 | - | 1 | 0 | 0 |
|  |  |  |  |  | HLA-B*08:01 |  |  |  |  |  |  |  |  |  |  |  |  |  |  |
|  |  |  |  |  | HLA-B*52:01 |  |  |  |  |  |  |  |  |  |  |  |  |  |  |
|  |  |  |  |  | HLA-C*01:02 |  |  |  |  |  |  |  |  |  |  |  |  |  |  |
|  |  |  |  |  | HLA-C*06:02 |  |  |  |  |  |  |  |  |  |  |  |  |  |  |
|  |  |  |  |  | HLA-C*07:01 |  |  |  |  |  |  |  |  |  |  |  |  |  |  |
| S507-515 | 507 | 507-515 | PYRVVVLSF | PYRVVVLSF | HLA-A*24:02 | 1 | 1 | 1 | 1 | 1 | 1 | 1 | 1 | 1 | 0 | - | 1 | 1 | 0 |
| S512-520 | 512 | 512-520 | VLSFELLHA | VLSFELLHA | HLA-A*02:03 | 1 | 1 | 1 | 1 | 1 | 1 | 1 | 1 | 1 | 1 | - | 1 | 1 | 0 |
| S515-523 | 515 | 515-523 | FELLHA**P**AT | FELLHAPAT | HLA-B*40:06 | 1 | 0 | 0 | 0 | 0 | 0 | 0 | 0 | 1 | 0 | - | 0 | 0 | 0 |
|  |  |  | FELLHA**T**AT | FELLHATAT | HLA-B*40:06 | 0 | 0 | 1 | 0 | 0 | 1 | 0 | 0 | 0 | 0 | - | 0 | 0 | 0 |
| S520-528 | 520 | 520-528 | A**S**ATVCGPK | ASATVCGPK | HLA-A*11:01 | 0 | 1 | 0 | 1 | 1 | 0 | 1 | 1 | 0 | 1 | - | 1 | 1 | 0 |
|  |  |  | A**T**ATVCGPK | ATATVCGPK | HLA-A*11:01 | 0 | 0 | 1 | 0 | 0 | 1 | 0 | 0 | 0 | 0 | - | 0 | 0 | 0 |
| S525-533 | 525 | 525-533 | CGPKKSTNL | CGPKKSTNL | HLA-C*01:02 | 1 | 1 | 1 | 1 | 1 | 1 | 1 | 1 | 1 | 1 | - | 1 | 1 | 0 |
| S526-534 | 526 | 526-534 | GPKKSTNLV | GPKKSTNLV | HLA-B*07:02 | 1 | 1 | 1 | 1 | 1 | 1 | 1 | 1 | 1 | 1 | - | 1 | 1 | 0 |
| S529-537 | 529 | 529-537 | KSTNLVKNK | KSTNLVKNK | HLA-A*11:01 | 1 | 1 | 1 | 1 | 1 | 1 | 1 | 1 | 1 | 1 | - | 1 | 1 | 0 |
| S533-541 | 533 | 533-541 | LVKNKCVNF | LVKNKCVNF | HLA-B*08:01 | 1 | 1 | 1 | 1 | 1 | 1 | 1 | 1 | 1 | 1 | - | 1 | 1 | 0 |
| S550-558 | 550 | 550-558 | GVLTESNKK | GVLTESNKK | HLA-A*11:01 | 1 | 1 | 1 | 1 | 1 | 1 | 1 | 1 | 1 | 1 | - | 1 | 1 | 0 |
| S554-562 | 554 | 554-562 | ESNKKFLPF | ESNKKFLPF | HLA-B*08:01 | 1 | 1 | 1 | 1 | 1 | 1 | 1 | 1 | 1 | 1 | - | 1 | 1 | 0 |
| S557-565 | 557 | 557-565 | KKFLPFQQF | KKFLPFQQF | HLA-B*52:01 | 1 | 1 | 1 | 1 | 1 | 1 | 1 | 1 | 1 | 1 | - | 1 | 1 | 0 |
|  |  |  |  |  | HLA-C*06:02 |  |  |  |  |  |  |  |  |  |  |  |  |  |  |
|  |  |  |  |  | HLA-C*07:01 |  |  |  |  |  |  |  |  |  |  |  |  |  |  |
| S574-582 | 574 | 574-582 | DAVRDPQTL | DAVRDPQTL | HLA-B*35:01 | 1 | 1 | 1 | 1 | 1 | 1 | 1 | 1 | 1 | 1 | - | 1 | 1 | 1 |
|  |  |  |  |  | HLA-B*51:01 |  |  |  |  |  |  |  |  |  |  |  |  |  |  |
| S576-584 | 576 | 576-584 | VRDPQTLEI | VRDPQTLEI | HLA-C*04:01 | 1 | 1 | 1 | 1 | 1 | 1 | 1 | 1 | 1 | 1 | - | 1 | 1 | 1 |
|  |  |  |  |  | HLA-C*06:02 |  |  |  |  |  |  |  |  |  |  |  |  |  |  |
|  |  |  |  |  | HLA-C*07:01 |  |  |  |  |  |  |  |  |  |  |  |  |  |  |
| S584-592 | 584 | 584-592 | ILDITPCSF | ILDITPCSF | HLA-C*04:01 | 1 | 1 | 1 | 1 | 1 | 1 | 1 | 1 | 1 | 1 |  | 1 | 1 | 1 |
| S604-612 | 604 | 604-612 | TSNQVAVLY | TSNQVAVLY | HLA-B*35:01 | 1 | 1 | 1 | 1 | 1 | 1 | 1 | 1 | 1 | 1 | - | 1 | 1 | 0 |
|  |  |  |  |  | HLA-B*46:01 |  |  |  |  |  |  |  |  |  |  |  |  |  |  |
|  |  |  |  |  | HLA-C*07:01 |  |  |  |  |  |  |  |  |  |  |  |  |  |  |
| S612-620 | 612 | 612-620 | YQDVNCTEV | YQDVNCTEV | HLA-A*02:05 | 1 | 0 | 0 | 0 | 0 | 0 | 0 | 0 | 0 | 0 | - | 0 | 0 | 0 |
|  |  |  |  |  | HLA-A*02:06 |  |  |  |  |  |  |  |  |  |  |  |  |  |  |
|  |  |  |  |  | HLA-C*04:01 |  |  |  |  |  |  |  |  |  |  |  |  |  |  |
| S618-626 | 618 | 618-626 | TEVPVAIHA | TEVPVAIHA | HLA-B*18:01 | 1 | 1 | 1 | 1 | 1 | 1 | 1 | 1 | 1 | 1 | - | 1 | 1 | 0 |
|  |  |  |  |  | HLA-B*40:06 |  |  |  |  |  |  |  |  |  |  |  |  |  |  |
| S625-633 | 625 | 625-633 | HADQLTPTW | HADQLTPTW | HLA-B*35:01 | 1 | 1 | 1 | 1 | 1 | 1 | 1 | 1 | 1 | 1 | - | 1 | 1 | 0 |
|  |  |  |  |  | HLA-C*04:01 |  |  |  |  |  |  |  |  |  |  |  |  |  |  |
| S627-635 | 627 | 627-635 | DQLTPTWRV | DQLTPTWRV | HLA-B*13:02 | 1 | 1 | 1 | 1 | 1 | 1 | 1 | 1 | 1 | 1 | - | 1 | 1 | 0 |
|  |  |  |  |  | HLA-B*52:01 |  |  |  |  |  |  |  |  |  |  |  |  |  |  |
| S628-636 | 628 | 628-636 | QLTPTWRVY | QLTPTWRVY | HLA-B*15:01 | 1 | 1 | 1 | 1 | 1 | 1 | 1 | 1 | 1 | 1 | - | 1 | 1 | 0 |
|  |  |  |  |  | HLA-B*46:01 |  |  |  |  |  |  |  |  |  |  |  |  |  |  |
| S634-642 | 634 | 634-642 | RVYSTGSNV | RVYSTGSNV | HLA-A*02:03 | 1 | 1 | 1 | 1 | 1 | 1 | 1 | 1 | 1 | 1 | - | 1 | 1 | 0 |
|  |  |  |  |  | HLA-A*02:05 |  |  |  |  |  |  |  |  |  |  |  |  |  |  |
|  |  |  |  |  | HLA-A*02:06 |  |  |  |  |  |  |  |  |  |  |  |  |  |  |
|  |  |  |  |  | HLA-B*13:02 |  |  |  |  |  |  |  |  |  |  |  |  |  |  |
|  |  |  |  |  | HLA-B*52:01 |  |  |  |  |  |  |  |  |  |  |  |  |  |  |
| S635-643 | 635 | 635-643 | VYSTGSNVF | VYSTGSNVF | HLA-A*24:02 | 1 | 1 | 1 | 1 | 1 | 1 | 1 | 1 | 1 | 1 | - | 1 | 1 | 0 |
|  |  |  |  |  | HLA-C*04:01 |  |  |  |  |  |  |  |  |  |  |  |  |  |  |
|  |  |  |  |  | HLA-C*07:01 |  |  |  |  |  |  |  |  |  |  |  |  |  |  |
| S660-668 | 660 | 660-668 | YECDIPIGA | YECDIPIGA | HLA-B*40:06 | 1 | 1 | 1 | 1 | 1 | 1 | 1 | 1 | 1 | 1 | - | 1 | 1 | 1 |
| S666-674 | 666 | 666-674 | IGAGICASY | IGAGICASY | HLA-B*46:01 | 1 | 1 | 1 | 1 | 1 | 1 | 1 | 1 | 1 | 1 | - | 1 | 1 | 1 |
| S679-687 | 679 | 679-687 | NSPRRARSV | NSPRRARSV | HLA-C*01:02 | 1 | 0 | 0 | 0 | 0 | 0 | 0 | 0 | 0 | 0 | - | 0 | 0 | 0 |
| S680-688 | 680 | 680-688 | SPRRARSVA | SPRRARSVA | HLA-B*07:02 | 1 | 0 | 0 | 0 | 0 | 0 | 0 | 0 | 0 | 0 | - | 0 | 0 | 0 |
| S684-692 | 684 | 684-692 | ARSVASQSI | ARSVASQSI | HLA-C*06:02 | 1 | 1 | 1 | 1 | 1 | 1 | 1 | 1 | 1 | 1 | - | 1 | 1 | 1 |
|  |  |  |  |  | HLA-C*07:01 |  |  |  |  |  |  |  |  |  |  |  |  |  |  |
| S687-695 | 687 | 687-695 | VASQSIIAY | VASQSIIAY | HLA-B*15:01 | 1 | 1 | 1 | 1 | 1 | 1 | 1 | 1 | 1 | 0 | - | 1 | 1 | 1 |
|  |  |  |  |  | HLA-B*35:01 |  |  |  |  |  |  |  |  |  |  |  |  |  |  |
|  |  |  |  |  | HLA-B*46:01 |  |  |  |  |  |  |  |  |  |  |  |  |  |  |
|  |  |  |  |  | HLA-C*07:01 |  |  |  |  |  |  |  |  |  |  |  |  |  |  |
| S689-697 | 689 | 689-697 | SQSIIAYTM | SQSIIAYTM | HLA-B*13:02 | 1 | 1 | 1 | 1 | 1 | 1 | 1 | 1 | 1 | 0 | - | 1 | 1 | 1 |
|  |  |  |  |  | HLA-B*15:01 |  |  |  |  |  |  |  |  |  |  |  |  |  |  |
|  |  |  |  |  | HLA-B*52:01 |  |  |  |  |  |  |  |  |  |  |  |  |  |  |
| S691-699 | 691 | 691-699 | SIIAYTMSL | SIIAYTMSL | HLA-A*02:01 | 1 | 1 | 1 | 1 | 1 | 1 | 1 | 1 | 1 | 0 | - | 1 | 1 | 1 |
|  |  |  |  |  | HLA-A*02:03 |  |  |  |  |  |  |  |  |  |  |  |  |  |  |
|  |  |  |  |  | HLA-A*02:05 |  |  |  |  |  |  |  |  |  |  |  |  |  |  |
|  |  |  |  |  | HLA-A*02:06 |  |  |  |  |  |  |  |  |  |  |  |  |  |  |
|  |  |  |  |  | HLA-B*08:01 |  |  |  |  |  |  |  |  |  |  |  |  |  |  |
|  |  |  |  |  | HLA-B*46:01 |  |  |  |  |  |  |  |  |  |  |  |  |  |  |
|  |  |  |  |  | HLA-C*01:02 |  |  |  |  |  |  |  |  |  |  |  |  |  |  |
| S699-707 | 699 | 699-707 | LGAENSVAY | LGAENSVAY | HLA-B*35:01 | 1 | 1 | 1 | 1 | 1 | 1 | 1 | 1 | 1 | 0 | - | 1 | 1 | 0 |
|  |  |  |  |  | HLA-B*46:01 |  |  |  |  |  |  |  |  |  |  |  |  |  |  |
| S710-718 | 710 | 710-718 | NSIAIPTNF | NSIAIPTNF | HLA-B*46:01 | 1 | 1 | 1 | 1 | 1 | 1 | 1 | 1 | 1 | 0 | - | 1 | 1 | 0 |
| S712-720 | 712 | 712-720 | IAIPTNFTI | IAIPTNFTI | HLA-B*13:02 | 1 | 1 | 1 | 1 | 1 | 1 | 1 | 1 | 1 | 0 | - | 1 | 1 | 0 |
|  |  |  |  |  | HLA-B*46:01 |  |  |  |  |  |  |  |  |  |  |  |  |  |  |
|  |  |  |  |  | HLA-B*51:01 |  |  |  |  |  |  |  |  |  |  |  |  |  |  |
|  |  |  |  |  | HLA-B*52:01 |  |  |  |  |  |  |  |  |  |  |  |  |  |  |
| S714-722 | 714 | 714-722 | IPTNFTISV | IPTNFTISV | HLA-B*07:02 | 1 | 1 | 1 | 1 | 1 | 1 | 1 | 1 | 1 | 0 | - | 1 | 1 | 0 |
|  |  |  |  |  | HLA-B*35:01 |  |  |  |  |  |  |  |  |  |  |  |  |  |  |
|  |  |  |  |  | HLA-B*51:01 |  |  |  |  |  |  |  |  |  |  |  |  |  |  |
| S718-726 | 718 | 718-726 | FTISVTTEI | FTISVTTEI | HLA-A*02:03 | 1 | 1 | 1 | 1 | 1 | 1 | 1 | 1 | 1 | 0 | - | 1 | 1 | 0 |
|  |  |  |  |  | HLA-A*02:05 |  |  |  |  |  |  |  |  |  |  |  |  |  |  |
|  |  |  |  |  | HLA-A*02:06 |  |  |  |  |  |  |  |  |  |  |  |  |  |  |
|  |  |  |  |  | HLA-B*46:01 |  |  |  |  |  |  |  |  |  |  |  |  |  |  |
|  |  |  |  |  | HLA-B*52:01 |  |  |  |  |  |  |  |  |  |  |  |  |  |  |
|  |  |  |  |  | HLA-C*06:02 |  |  |  |  |  |  |  |  |  |  |  |  |  |  |
| S721-729 | 721 | 721-729 | SVTTEILPV | SVTTEILPV | HLA-A*02:06 | 1 | 1 | 1 | 1 | 1 | 1 | 1 | 1 | 1 | 0 | - | 1 | 1 | 0 |
| S724-732 | 724 | 724-732 | TEILPVSMT | TEILPVSMT | HLA-B*40:06 | 1 | 1 | 1 | 1 | 1 | 1 | 1 | 1 | 1 | 0 | - | 1 | 1 | 0 |
| S725-733 | 725 | 725-733 | EILPVSMTK | EILPVSMTK | HLA-A*11:01 | 1 | 1 | 1 | 1 | 1 | 1 | 1 | 1 | 1 | 0 | - | 1 | 1 | 0 |
| S755-763 | 755 | 755-763 | QYGSFCTQL | QYGSFCTQL | HLA-A*24:02 | 1 | 1 | 1 | 1 | 1 | 1 | 1 | 1 | 1 | 1 | - | 1 | 1 | 0 |
| S757-765 | 757 | 757-765 | GSFCTQL**K**R | GSFCTQLKR | HLA-A*11:01 | 0 | 1 | 1 | 1 | 1 | 1 | 1 | 1 | 1 | 0 | - | 1 | 1 | 0 |
|  |  |  | GSFCTQL**N**R | GSFCTQLNR | HLA-A*11:01 | 1 | 0 | 0 | 0 | 0 | 0 | 0 | 0 | 0 | 1 | - | 0 | 0 | 0 |
| S762-770 | 762 | 762-770 | QLKRALTGI | QLKRALTGI | HLA-A*02:03 | 0 | 1 | 1 | 1 | 1 | 1 | 1 | 1 | 1 | 0 | - | 1 | 1 | 0 |
| S764-772 | 764 | 764-772 | KRALTGIAV | KRALTGIAV | HLA-C*06:02 | 0 | 1 | 1 | 1 | 1 | 1 | 1 | 1 | 1 | 0 | - | 1 | 1 | 0 |
| S777-785 | 777 | 777-785 | NTQEVFAQV | NTQEVFAQV | HLA-A*02:05 | 1 | 1 | 1 | 1 | 1 | 1 | 1 | 1 | 1 | 1 | - | 1 | 0 | 0 |
|  |  |  |  |  | HLA-A*02:06 |  |  |  |  |  |  |  |  |  |  |  |  |  |  |
| S780-788 | 780 | 780-788 | EVFAQVKQI | EVFAQVKQI | HLA-B*52:01 | 1 | 1 | 1 | 1 | 1 | 1 | 1 | 1 | 1 | 1 | - | 1 | 0 | 0 |
| S781-789 | 781 | 781-789 | VFAQVKQIY | VFAQVKQIY | HLA-C*07:01 | 1 | 1 | 1 | 1 | 1 | 1 | 1 | 1 | 1 | 1 | - | 1 | 0 | 0 |
| S783-791 | 783 | 783-791 | AQVKQIYKT | AQVKQIYKT | HLA-A*02:06 | 1 | 1 | 1 | 1 | 1 | 1 | 1 | 1 | 1 | 1 | - | 1 | 0 | 0 |
|  |  |  |  |  | HLA-B*13:02 |  |  |  |  |  |  |  |  |  |  |  |  |  |  |
| S786-794 | 786 | 786-794 | KQIYKTPPI | KQIYKTPPI | HLA-A*02:06 | 1 | 1 | 1 | 1 | 1 | 1 | 1 | 1 | 1 | 1 | - | 1 | 0 | 0 |
|  |  |  |  |  | HLA-B*13:02 |  |  |  |  |  |  |  |  |  |  |  |  |  |  |
|  |  |  |  |  | HLA-B*52:01 |  |  |  |  |  |  |  |  |  |  |  |  |  |  |
| S787-795 | 787 | 787-795 | QIYKTPPIK | QIYKTPPIK | HLA-A*11:01 | 1 | 1 | 1 | 1 | 1 | 1 | 1 | 1 | 1 | 1 | - | 1 | 0 | 0 |
| S788-796 | 788 | 788-796 | IYKTPPIKY | IYKTPPIKY | HLA-A*24:02 | 0 | 1 | 1 | 1 | 1 | 1 | 1 | 1 | 1 | 0 |  | 1 | 0 | 0 |
|  |  |  |  |  | HLA-C*06:02 |  |  |  |  |  |  |  |  |  |  |  |  |  |  |
|  |  |  |  |  | HLA-C*07:01 |  |  |  |  |  |  |  |  |  |  |  |  |  |  |
| S789-797 | 789 | 789-797 | YKTPPIK**Y**F | YKTPPIKYF | HLA-C*06:02 | 0 | 1 | 1 | 1 | 1 | 1 | 1 | 1 | 1 | 0 | - | 1 | 0 | 0 |
|  |  |  |  |  | HLA-C*07:01 |  |  |  |  |  |  |  |  |  |  |  |  |  |  |
|  |  |  | YKTPPIK**D**F | YKTPPIKDF | HLA-C*06:02 | 1 | 0 | 0 | 0 | 0 | 0 | 0 | 0 | 0 | 1 | - | 0 | 0 | 0 |
|  |  |  |  |  | HLA-C*07:01 |  |  |  |  |  |  |  |  |  |  |  |  |  |  |
| S803-811 | 803 | 803-811 | SQILPDPSK | SQILPDPSK | HLA-A*11:01 | 1 | 1 | 1 | 1 | 1 | 1 | 1 | 1 | 1 | 1 | - | 1 | 0 | 0 |
| S814-822 | 814 | 814-822 | KRSFIEDLL | KRSFIEDLL | HLA-C*07:01 | 1 | 1 | 1 | 1 | 1 | 1 | 1 | 1 | 1 | 1 | - | 1 | 0 | 0 |
| S818-826 | 818 | 818-826 | IEDLLFNKV | IEDLLFNKV | HLA-B*40:06 | 1 | 1 | 1 | 1 | 1 | 1 | 1 | 1 | 1 | 1 | - | 1 | 1 | 0 |
| S820-828 | 820 | 820-828 | DLLFNKVTL | DLLFNKVTL | HLA-B*08:01 | 1 | 1 | 1 | 1 | 1 | 1 | 1 | 1 | 1 | 1 | - | 1 | 1 | 0 |
| S821-829 | 821 | 821-829 | LLFNKVTLA | LLFNKVTLA | HLA-A*02:01 | 1 | 1 | 1 | 1 | 1 | 1 | 1 | 1 | 1 | 1 | - | 1 | 1 | 0 |
|  |  |  |  |  | HLA-A*02:03 |  |  |  |  |  |  |  |  |  |  |  |  |  |  |
|  |  |  |  |  | HLA-A*02:05 |  |  |  |  |  |  |  |  |  |  |  |  |  |  |
|  |  |  |  |  | HLA-A*02:06 |  |  |  |  |  |  |  |  |  |  |  |  |  |  |
| S827-835 | 827 | 827-835 | TLADAGFIK | TLADAGFIK | HLA-A*11:01 | 1 | 1 | 1 | 1 | 1 | 1 | 1 | 1 | 1 | 1 | - | 1 | 1 | 0 |
| S833-841 | 833 | 833-841 | FIKQYGDCL | FIKQYGDCL | HLA-B*08:01 | 1 | 1 | 1 | 1 | 1 | 1 | 1 | 1 | 1 | 1 | - | 1 | 1 | 0 |
| S852-860 | 852 | 852-860 | AQK**F**NGLTV | AQKFNGLTV | HLA-B*13:02 | 1 | 1 | 1 | 1 | 1 | 1 | 1 | 1 | 1 | 1 | - | 1 | 0 | 0 |
|  |  |  |  |  | HLA-B*52:01 |  |  |  |  |  |  |  |  |  |  |  |  |  |  |
|  |  |  | AQK**V**NGLTV | AQKVNGLTV | HLA-B*13:02 | 0 | 0 | 0 | 0 | 0 | 0 | 0 | 0 | 0 | 0 | - | 0 | 1 | 0 |
|  |  |  |  |  | HLA-B*52:01 |  |  |  |  |  |  |  |  |  |  |  |  |  |  |
| S857-865 | 857 | 857-865 | GLTVLPPLL | GLTVLPPLL | HLA-A*02:01 | 1 | 1 | 1 | 1 | 1 | 1 | 1 | 1 | 1 | 1 | - | 1 | 1 | 0 |
| S861-869 | 861 | 861-869 | LPPLLTDEM | LPPLLTDEM | HLA-B*35:01 | 1 | 1 | 1 | 1 | 1 | 1 | 1 | 1 | 1 | 1 | - | 1 | 1 | 0 |
| S865-873 | 865 | 865-873 | LTDEMIAQY | LTDEMIAQY | HLA-B*35:01 | 1 | 1 | 1 | 1 | 1 | 1 | 1 | 1 | 1 | 1 | - | 1 | 1 | 0 |
|  |  |  |  |  | HLA-C*04:01 |  |  |  |  |  |  |  |  |  |  |  |  |  |  |
| S867-875 | 867 | 867-875 | DEMIAQYTS | DEMIAQYTS | HLA-B*18:01 | 1 | 1 | 1 | 1 | 1 | 1 | 1 | 1 | 1 | 1 | - | 1 | 1 | 1 |
| S869-877 | 869 | 869-877 | MIAQYTSAL | MIAQYTSAL | HLA-A*02:05 | 1 | 1 | 1 | 1 | 1 | 1 | 1 | 1 | 1 | 1 | - | 1 | 1 | 1 |
|  |  |  |  |  | HLA-B*08:01 |  |  |  |  |  |  |  |  |  |  |  |  |  |  |
|  |  |  |  |  | HLA-B*46:01 |  |  |  |  |  |  |  |  |  |  |  |  |  |  |
|  |  |  |  |  | HLA-C*01:02 |  |  |  |  |  |  |  |  |  |  |  |  |  |  |
| S870-878 | 870 | 870-878 | IAQYTSALL | IAQYTSALL | HLA-C*01:02 | 1 | 1 | 1 | 1 | 1 | 1 | 1 | 1 | 1 | 1 | - | 1 | 1 | 1 |
| S871-879 | 871 | 871-879 | AQYTSALLA | AQYTSALLA | HLA-B*13:02 | 1 | 1 | 1 | 1 | 1 | 1 | 1 | 1 | 1 | 1 | - | 1 | 1 | 1 |
| S892-900 | 892 | 892-900 | AALQIPFAM | AALQIPFAM | HLA-B*35:01 | 1 | 1 | 1 | 1 | 1 | 1 | 1 | 1 | 1 | 1 | - | 1 | 1 | 1 |
|  |  |  |  |  | HLA-B*46:01 |  |  |  |  |  |  |  |  |  |  |  |  |  |  |
| S894-902 | 894 | 894-902 | LQIPFAMQM | LQIPFAMQM | HLA-A*02:05 | 1 | 1 | 1 | 1 | 1 | 1 | 1 | 1 | 1 | 1 | - | 1 | 1 | 1 |
|  |  |  |  |  | HLA-A*02:06 |  |  |  |  |  |  |  |  |  |  |  |  |  |  |
|  |  |  |  |  | HLA-B*13:02 |  |  |  |  |  |  |  |  |  |  |  |  |  |  |
|  |  |  |  |  | HLA-B*15:01 |  |  |  |  |  |  |  |  |  |  |  |  |  |  |
|  |  |  |  |  | HLA-B*46:01 |  |  |  |  |  |  |  |  |  |  |  |  |  |  |
|  |  |  |  |  | HLA-B*52:01 |  |  |  |  |  |  |  |  |  |  |  |  |  |  |
|  |  |  |  |  | HLA-C*06:02 |  |  |  |  |  |  |  |  |  |  |  |  |  |  |
| S896-904 | 896 | 896-904 | IPFAMQMAY | IPFAMQMAY | HLA-B*18:01 | 1 | 1 | 1 | 1 | 1 | 1 | 1 | 1 | 1 | 1 | - | 1 | 1 | 1 |
|  |  |  |  |  | HLA-B*35:01 |  |  |  |  |  |  |  |  |  |  |  |  |  |  |
| S898-906 | 898 | 898-906 | FAMQMAYRF | FAMQMAYRF | HLA-B*35:01 | 1 | 1 | 1 | 1 | 1 | 1 | 1 | 1 | 1 | 1 | - | 1 | 1 | 1 |
|  |  |  |  |  | HLA-B*46:01 |  |  |  |  |  |  |  |  |  |  |  |  |  |  |
| S915-923 | 915 | 915-923 | VLYENQKLI | VLYENQKLI | HLA-A*02:01 | 1 | 1 | 1 | 1 | 1 | 1 | 1 | 1 | 1 | 1 | - | 1 | 1 | 1 |
|  |  |  |  |  | HLA-A*02:03 |  |  |  |  |  |  |  |  |  |  |  |  |  |  |
|  |  |  |  |  | HLA-B*13:02 |  |  |  |  |  |  |  |  |  |  |  |  |  |  |
|  |  |  |  |  | HLA-B*52:01 |  |  |  |  |  |  |  |  |  |  |  |  |  |  |
| S919-927 | 919 | 919-927 | NQKLIANQF | NQKLIANQF | HLA-B*15:01 | 1 | 1 | 1 | 1 | 1 | 1 | 1 | 1 | 1 | 1 | - | 1 | 1 | 1 |
|  |  |  |  |  | HLA-B*18:01 |  |  |  |  |  |  |  |  |  |  |  |  |  |  |
|  |  |  |  |  | HLA-B*52:01 |  |  |  |  |  |  |  |  |  |  |  |  |  |  |
| S923-931 | 923 | 923-931 | IANQFNSAI | IANQFNSAI | HLA-B*51:01 | 1 | 1 | 1 | 1 | 1 | 1 | 1 | 1 | 1 | 1 | - | 1 | 1 | 1 |
|  |  |  |  |  | HLA-B*52:01 |  |  |  |  |  |  |  |  |  |  |  |  |  |  |
| S937-945 | 937 | 937-945 | SLSSTASAL | SLSSTASAL | HLA-A*02:03 | 1 | 1 | 1 | 1 | 1 | 1 | 1 | 1 | 1 | 1 | - | 1 | 1 | 1 |
|  |  |  |  |  | HLA-C*01:02 |  |  |  |  |  |  |  |  |  |  |  |  |  |  |
| S939-947 | 939 | 939-947 | SSTASALGK | SSTASALGK | HLA-A*11:01 | 1 | 1 | 1 | 1 | 1 | 1 | 1 | 1 | 1 | 1 | - | 1 | 1 | 1 |
| S951-959 | 951 | 951-959 | VVN**H**NAQAL | VVNHNAQAL | HLA-C*01:02 | 0 | 1 | 1 | 1 | 1 | 1 | 1 | 1 | 1 | 1 | - | 1 | 1 | 0 |
|  |  |  | VVN**Q**NAQAL | VVNQNAQAL | HLA-B*46:01 | 1 | 0 | 0 | 0 | 0 | 0 | 0 | 0 | 0 | 0 | - | 0 | 0 | 0 |
|  |  |  |  |  | HLA-C*01:02 |  |  |  |  |  |  |  |  |  |  |  |  |  |  |
| S956-964 | 956 | 956-964 | AQALNTLVK | AQALNTLVK | HLA-A*11:01 | 1 | 1 | 1 | 1 | 1 | 1 | 1 | 1 | 1 | 1 | - | 1 | 1 | 0 |
| S958-966 | 958 | 958-966 | ALNTLVKQL | ALNTLVKQL | HLA-A*02:01 | 1 | 1 | 1 | 1 | 1 | 1 | 1 | 1 | 1 | 1 | - | 1 | 1 | 0 |
|  |  |  |  |  | HLA-A*02:03 |  |  |  |  |  |  |  |  |  |  |  |  |  |  |
|  |  |  |  |  | HLA-A*02:05 |  |  |  |  |  |  |  |  |  |  |  |  |  |  |
|  |  |  |  |  | HLA-B*13:02 |  |  |  |  |  |  |  |  |  |  |  |  |  |  |
| S961-969 | 961 | 961-969 | TLVKQLSSK | TLVKQLSSK | HLA-A*11:01 | 0 | 1 | 1 | 1 | 1 | 1 | 1 | 1 | 1 | 1 | - | 1 | 1 | 0 |
| S962-970 | 962 | 962-970 | LVKQLSS**K**F | LVKQLSSKF | HLA-B*15:01 | 0 | 1 | 1 | 1 | 1 | 1 | 1 | 1 | 1 | 1 | - | 1 | 1 | 0 |
|  |  |  |  |  | HLA-B*46:01 |  |  |  |  |  |  |  |  |  |  |  |  |  |  |
|  |  |  | LVKQLSS**N**F | LVKQLSSNF | HLA-B*15:01 | 1 | 0 | 0 | 0 | 0 | 0 | 0 | 0 | 0 | 0 | - | 0 | 0 | 0 |
|  |  |  |  |  | HLA-B*46:01 |  |  |  |  |  |  |  |  |  |  |  |  |  |  |
| S964-972 | 964 | 964-972 | KQLSS**K**FGA | KQLSSKFGA | HLA-B*13:02 | 0 | 1 | 1 | 1 | 1 | 1 | 1 | 1 | 1 | 1 | - | 1 | 1 | 0 |
| S964-972 | 964 | 964-972 | KQLSS**N**FGA | KQLSSNFGA | HLA-B*13:02 | 1 | 0 | 0 | 0 | 0 | 0 | 0 | 0 | 0 | 0 | - | 0 | 0 | 0 |
| S968-976 | 968 | 968-976 | S**K**FGAISSV | SKFGAISSV | HLA-B*13:02 | 0 | 1 | 1 | 1 | 1 | 1 | 1 | 1 | 1 | 1 | - | 1 | 1 | 1 |
|  |  |  |  |  | HLA-B*52:01 |  |  |  |  |  |  |  |  |  |  |  |  |  |  |
|  |  |  |  |  | HLA-C*06:02 |  |  |  |  |  |  |  |  |  |  |  |  |  |  |
|  |  |  | S**N**FGAISSV | SNFGAISSV | HLA-B*13:02 | 1 | 0 | 0 | 0 | 0 | 0 | 0 | 0 | 0 | 0 | - | 0 | 0 | 0 |
|  |  |  |  |  | HLA-B*52:01 |  |  |  |  |  |  |  |  |  |  |  |  |  |  |
| S975-983 | 975 | 975-983 | SVLNDILSR | SVLNDILSR | HLA-A*11:01 | 1 | 1 | 1 | 1 | 1 | 1 | 1 | 1 | 1 | 1 | - | 1 | 1 | 1 |
| S976-984 | 976 | 976-984 | VLNDILSRL | VLNDILSRL | HLA-A*02:01 | 1 | 1 | 1 | 1 | 1 | 1 | 1 | 1 | 1 | 1 | - | 1 | 1 | 1 |
|  |  |  |  |  | HLA-A*02:03 |  |  |  |  |  |  |  |  |  |  |  |  |  |  |
|  |  |  |  |  | HLA-A*02:05 |  |  |  |  |  |  |  |  |  |  |  |  |  |  |
|  |  |  |  |  | HLA-A*02:06 |  |  |  |  |  |  |  |  |  |  |  |  |  |  |
|  |  |  |  |  | HLA-B*13:02 |  |  |  |  |  |  |  |  |  |  |  |  |  |  |
|  |  |  |  |  | HLA-C*01:02 |  |  |  |  |  |  |  |  |  |  |  |  |  |  |
|  |  |  |  |  | HLA-C*04:01 |  |  |  |  |  |  |  |  |  |  |  |  |  |  |
| S983-991 | 983 | 983-991 | RLDKVEAEV | RLDKVEAEV | HLA-A*02:01 | 1 | 1 | 1 | 1 | 1 | 1 | 1 | 1 | 1 | 1 | - | 1 | 1 | 1 |
|  |  |  |  |  | HLA-A*02:03 |  |  |  |  |  |  |  |  |  |  |  |  |  |  |
|  |  |  |  |  | HLA-A*02:06 |  |  |  |  |  |  |  |  |  |  |  |  |  |  |
|  |  |  |  |  | HLA-B*13:02 |  |  |  |  |  |  |  |  |  |  |  |  |  |  |
|  |  |  |  |  | HLA-C*04:01 |  |  |  |  |  |  |  |  |  |  |  |  |  |  |
| S989-997 | 989 | 989-997 | AEVQIDRLI | AEVQIDRLI | HLA-B*40:06 | 1 | 1 | 1 | 1 | 1 | 1 | 1 | 1 | 1 | 1 | - | 1 | 1 | 1 |
|  |  |  |  |  | HLA-B*52:01 |  |  |  |  |  |  |  |  |  |  |  |  |  |  |
| S996-1004 | 996 | 996-1004 | LITGRLQSL | LITGRLQSL | HLA-B*08:01 | 1 | 1 | 1 | 1 | 1 | 1 | 1 | 1 | 1 | 1 | - | 1 | 1 | 1 |
| S999-1007 | 999 | 999-1007 | GRLQSLQTY | GRLQSLQTY | HLA-C*06:02 | 1 | 1 | 1 | 1 | 1 | 1 | 1 | 1 | 1 | 0 | - | 1 | 1 | 1 |
|  |  |  |  |  | HLA-C*07:01 |  |  |  |  |  |  |  |  |  |  |  |  |  |  |
| S1000-1008 | 1000 | 1000-1008 | RLQSLQTYV | RLQSLQTYV | HLA-A*02:01 | 1 | 1 | 1 | 1 | 1 | 1 | 1 | 1 | 1 | 0 | - | 1 | 1 | 1 |
|  |  |  |  |  | HLA-A*02:03 |  |  |  |  |  |  |  |  |  |  |  |  |  |  |
|  |  |  |  |  | HLA-A*02:05 |  |  |  |  |  |  |  |  |  |  |  |  |  |  |
|  |  |  |  |  | HLA-A*02:06 |  |  |  |  |  |  |  |  |  |  |  |  |  |  |
|  |  |  |  |  | HLA-B*13:02 |  |  |  |  |  |  |  |  |  |  |  |  |  |  |
| S1004-1012 | 1004 | 1004-1012 | LQTYVTQQL | LQTYVTQQL | HLA-A*02:05 | 1 | 1 | 1 | 1 | 1 | 1 | 1 | 1 | 1 | 0 | - | 1 | 1 | 1 |
|  |  |  |  |  | HLA-A*02:06 |  |  |  |  |  |  |  |  |  |  |  |  |  |  |
|  |  |  |  |  | HLA-B*13:02 |  |  |  |  |  |  |  |  |  |  |  |  |  |  |
|  |  |  |  |  | HLA-B*52:01 |  |  |  |  |  |  |  |  |  |  |  |  |  |  |
| S1005-1013 | 1005 | 1005-1013 | QTYVTQQLI | QTYVTQQLI | HLA-B*13:02 | 1 | 1 | 1 | 1 | 1 | 1 | 1 | 1 | 1 | 0 | - | 1 | 1 | 1 |
|  |  |  |  |  | HLA-B*52:01 |  |  |  |  |  |  |  |  |  |  |  |  |  |  |
| S1010-1018 | 1010 | 1010-1018 | QQLIRAAEI | QQLIRAAEI | HLA-B*13:02 | 1 | 1 | 1 | 1 | 1 | 1 | 1 | 1 | 1 | 0 | - | 1 | 1 | 1 |
|  |  |  |  |  | HLA-B*52:01 |  |  |  |  |  |  |  |  |  |  |  |  |  |  |
| S1020-1028 | 1020 | 1020-1028 | **A**SANLAATK | ASANLAATK | HLA-A*11:01 | 1 | 1 | 1 | 0 | 1 | 1 | 1 | 1 | 1 | 1 | - | 1 | 1 | 1 |
|  |  |  | **V**SANLAATK | VSANLAATK | HLA-A*11:01 | 0 | 0 | 0 | 1 | 0 | 0 | 0 | 0 | 0 | 0 | - | 0 | 0 | 0 |
| S1048-1056 | 1048 | 1048-1056 | HLMSFPQSA | HLMSFPQSA | HLA-A*02:01 | 1 | 1 | 1 | 1 | 1 | 1 | 1 | 1 | 1 | 1 | - | 1 | 1 | 1 |
|  |  |  |  |  | HLA-A*02:03 |  |  |  |  |  |  |  |  |  |  |  |  |  |  |
|  |  |  |  |  | HLA-A*02:05 |  |  |  |  |  |  |  |  |  |  |  |  |  |  |
|  |  |  |  |  | HLA-A*02:06 |  |  |  |  |  |  |  |  |  |  |  |  |  |  |
|  |  |  |  |  | HLA-B*13:02 |  |  |  |  |  |  |  |  |  |  |  |  |  |  |
| S1052-1060 | 1052 | 1052-1060 | FPQSAPHGV | FPQSAPHGV | HLA-B*07:02 | 1 | 1 | 1 | 1 | 1 | 1 | 1 | 1 | 1 | 1 | - | 1 | 1 | 1 |
|  |  |  |  |  | HLA-B*35:01 |  |  |  |  |  |  |  |  |  |  |  |  |  |  |
|  |  |  |  |  | HLA-B*51:01 |  |  |  |  |  |  |  |  |  |  |  |  |  |  |
|  |  |  |  |  | HLA-C*04:01 |  |  |  |  |  |  |  |  |  |  |  |  |  |  |
| S1054-1062 | 1054 | 1054-1062 | QSAPHGVVF | QSAPHGVVF | HLA-B*15:01 | 1 | 1 | 1 | 1 | 1 | 1 | 1 | 1 | 1 | 1 | - | 1 | 1 | 1 |
|  |  |  |  |  | HLA-B*35:01 |  |  |  |  |  |  |  |  |  |  |  |  |  |  |
|  |  |  |  |  | HLA-B*46:01 |  |  |  |  |  |  |  |  |  |  |  |  |  |  |
|  |  |  |  |  | HLA-C*07:01 |  |  |  |  |  |  |  |  |  |  |  |  |  |  |
| S1055-1063 | 1055 | 1055-1063 | SAPHGVVFL | SAPHGVVFL | HLA-C*01:02 | 1 | 1 | 1 | 1 | 1 | 1 | 1 | 1 | 1 | 1 | - | 1 | 1 | 1 |
|  |  |  |  |  | HLA-C*06:02 |  |  |  |  |  |  |  |  |  |  |  |  |  |  |
| S1059-1067 | 1059 | 1059-1067 | GVVFLHVTY | GVVFLHVTY | HLA-B*15:01 | 1 | 1 | 1 | 1 | 1 | 1 | 1 | 1 | 1 | 1 | - | 1 | 1 | 1 |
| S1060-1068 | 1060 | 1060-1068 | VVFLHVTYV | VVFLHVTYV | HLA-A*02:01 | 1 | 1 | 1 | 1 | 1 | 1 | 1 | 1 | 1 | 1 | - | 1 | 1 | 1 |
|  |  |  |  |  | HLA-A*02:03 |  |  |  |  |  |  |  |  |  |  |  |  |  |  |
|  |  |  |  |  | HLA-A*02:05 |  |  |  |  |  |  |  |  |  |  |  |  |  |  |
|  |  |  |  |  | HLA-A*02:06 |  |  |  |  |  |  |  |  |  |  |  |  |  |  |
|  |  |  |  |  | HLA-B*13:02 |  |  |  |  |  |  |  |  |  |  |  |  |  |  |
|  |  |  |  |  | HLA-B*52:01 |  |  |  |  |  |  |  |  |  |  |  |  |  |  |
|  |  |  |  |  | HLA-C*06:02 |  |  |  |  |  |  |  |  |  |  |  |  |  |  |
| S1065-1073 | 1065 | 1065-1073 | VTYVPAQEK | VTYVPAQEK | HLA-A*11:01 | 1 | 1 | 1 | 1 | 1 | 1 | 1 | 1 | 1 | 1 | - | 1 | 1 | 1 |
| S1067-1075 | 1067 | 1067-1075 | YVPAQEKNF | YVPAQEKNF | HLA-C*01:02 | 1 | 1 | 1 | 1 | 1 | 1 | 1 | 1 | 1 | 1 | - | 1 | 1 | 1 |
| S1071-1079 | 1071 | 1071-1079 | QEKNFTTAP | QEKNFTTAP | HLA-B*40:06 | 1 | 1 | 1 | 1 | 1 | 1 | 1 | 1 | 1 | 1 | - | 1 | 1 | 1 |
| S1073-1081 | 1073 | 1073-1081 | KNFTTAPAI | KNFTTAPAI | HLA-B*52:01 | 1 | 1 | 1 | 1 | 1 | 1 | 1 | 1 | 1 | 1 | - | 1 | 1 | 1 |
| S1087-1095 | 1087 | 1087-1095 | AHFPREGVF | AHFPREGVF | HLA-C*07:01 | 1 | 1 | 1 | 1 | 1 | 1 | 1 | 1 | 1 | 0 | - | 1 | 1 | 1 |
| S1094-1102 | 1094 | 1094-1102 | VFVSNGTHW | VFVSNGTHW | HLA-A*24:02 | 1 | 1 | 1 | 1 | 1 | 1 | 1 | 1 | 1 | 1 | - | 1 | 1 | 1 |
| S1095-1103 | 1095 | 1095-1103 | FVSNGTHWF | FVSNGTHWF | HLA-B*46:01 | 1 | 1 | 1 | 1 | 1 | 1 | 1 | 1 | 1 | 1 | - | 1 | 1 | 1 |
| S1099-1107 | 1099 | 1099-1107 | GTHWFVTQR | GTHWFVTQR | HLA-A*11:01 | 1 | 1 | 1 | 1 | 1 | 1 | 1 | 1 | 1 | 1 | - | 1 | 1 | 1 |
| S1101-1109 | 1101 | 1101-1109 | HWFVTQRNF | HWFVTQRNF | HLA-A*24:02 | 1 | 1 | 1 | 1 | 1 | 1 | 1 | 1 | 1 | 1 | - | 1 | 1 | 1 |
| S1106-1114 | 1106 | 1106-1114 | QRNFYEPQI | QRNFYEPQI | HLA-C*06:02 | 1 | 1 | 1 | 1 | 1 | 1 | 1 | 1 | 1 | 1 | - | 1 | 1 | 1 |
| S1107-1115 | 1107 | 1107-1115 | RNFYEPQII | RNFYEPQII | HLA-B*52:01 | 1 | 1 | 1 | 1 | 1 | 1 | 1 | 1 | 1 | 1 | - | 1 | 1 | 1 |
| S1109-1117 | 1109 | 1109-1117 | FYEPQIITT | FYEPQIITT | HLA-C*04:01 | 1 | 1 | 1 | 1 | 1 | 1 | 1 | 1 | 1 | 1 | - | 1 | 1 | 1 |
| S1113-1121 | 1113 | 1113-1121 | QIITTDNTF | QIITTDNTF | HLA-B*15:01 | 1 | 1 | 1 | 1 | 1 | 1 | 1 | 1 | 1 | 1 | - | 1 | 1 | 1 |
| S1116-1124 | 1116 | 1116-1124 | AEIR**A**SANL | AEIRASANL | HLA-B*40:06 | 1 | 1 | 1 | 0 | 1 | 1 | 1 | 1 | 1 | 1 | - | 1 | 1 | 1 |
|  |  |  | AEIR**V**SANL | AEIRVSANL | HLA-B*40:06 | 0 | 0 | 0 | 1 | 0 | 0 | 0 | 0 | 0 | 0 | - | 0 | 0 | 0 |
| S1130-1138 | 1130 | 1130-1138 | IGIVNNTVY | IGIVNNTVY | HLA-B*46:01 | 1 | 1 | 1 | 1 | 1 | 1 | 1 | 1 | 1 | 1 | - | 1 | 1 | 1 |
| S1137-1145 | 1137 | 1137-1145 | VYDPLQPEL | VYDPLQPEL | HLA-A*24:02 | 1 | 1 | 1 | 1 | 1 | 1 | 1 | 1 | 1 | 1 | - | 1 | 1 | 1 |
|  |  |  |  |  | HLA-B*08:01 |  |  |  |  |  |  |  |  |  |  |  |  |  |  |
|  |  |  |  |  | HLA-C*01:02 |  |  |  |  |  |  |  |  |  |  |  |  |  |  |
|  |  |  |  |  | HLA-C*04:01 |  |  |  |  |  |  |  |  |  |  |  |  |  |  |
|  |  |  |  |  | HLA-C*06:02 |  |  |  |  |  |  |  |  |  |  |  |  |  |  |
|  |  |  |  |  | HLA-C*07:01 |  |  |  |  |  |  |  |  |  |  |  |  |  |  |
| S1144-1152 | 1144 | 1144-1152 | ELDSFKEEL | ELDSFKEEL | HLA-C*04:01 | 1 | 1 | 1 | 1 | 1 | 1 | 1 | 1 | 1 | 1 | - | 1 | 1 | 1 |
| S1171-1179 | 1171 | 1171-1179 | GINASVVNI | GINASVVNI | HLA-B*13:02 | 1 | 1 | 1 | 1 | 1 | 1 | 1 | 1 | 1 | 0 | - | 1 | 1 | 1 |
| S1175-1183 | 1175 | 1175-1183 | SVVNIQKEI | SVVNIQKEI | HLA-B*13:02 | 1 | 1 | 1 | 1 | 1 | 1 | 1 | 1 | 1 | 0 | - | 1 | 1 | 1 |
| S1181-1189 | 1181 | 1181-1189 | KEIDRLNEV | KEIDRLNEV | HLA-A*02:05 | 1 | 1 | 1 | 1 | 1 | 1 | 1 | 1 | 1 | 0 | - | 1 | 1 | 1 |
|  |  |  |  |  | HLA-B*13:02 |  |  |  |  |  |  |  |  |  |  |  |  |  |  |
|  |  |  |  |  | HLA-B*40:06 |  |  |  |  |  |  |  |  |  |  |  |  |  |  |
|  |  |  |  |  | HLA-B*52:01 |  |  |  |  |  |  |  |  |  |  |  |  |  |  |
| S1185-1193 | 1185 | 1185-1193 | RLNEVAKNL | RLNEVAKNL | HLA-A*02:01 | 1 | 1 | 1 | 1 | 1 | 1 | 1 | 1 | 1 | 0 | - | 1 | 1 | 1 |
|  |  |  |  |  | HLA-A*02:03 |  |  |  |  |  |  |  |  |  |  |  |  |  |  |
|  |  |  |  |  | HLA-A*02:05 |  |  |  |  |  |  |  |  |  |  |  |  |  |  |
|  |  |  |  |  | HLA-B*13:02 |  |  |  |  |  |  |  |  |  |  |  |  |  |  |
| S1192-1200 | 1192 | 1192-1200 | NLNESLIDL | NLNESLIDL | HLA-A*02:01 | 1 | 1 | 1 | 1 | 1 | 1 | 1 | 1 | 1 | 0 | - | 1 | 1 | 1 |
|  |  |  |  |  | HLA-A*02:03 |  |  |  |  |  |  |  |  |  |  |  |  |  |  |
| S1201-1209 | 1201 | 1201-1209 | QELGKYEQY | QELGKYEQY | HLA-B*18:01 | 1 | 1 | 1 | 1 | 1 | 1 | 1 | 1 | 1 | 0 | - | 1 | 1 | 1 |
| S1206-1214 | 1206 | 1206-1214 | YEQYIKWPW | YEQYIKWPW | HLA-B*18:01 | 1 | 1 | 1 | 1 | 1 | 1 | 1 | 1 | 1 | 0 | - | 1 | 1 | 1 |
| S1208-1216 | 1208 | 1208-1216 | QYIKWPWYI | QYIKWPWYI | HLA-A*24:02 | 1 | 1 | 1 | 1 | 1 | 1 | 1 | 1 | 1 | 0 | - | 1 | 1 | 1 |
|  |  |  |  |  | HLA-C*06:02 |  |  |  |  |  |  |  |  |  |  |  |  |  |  |
|  |  |  |  |  | HLA-C*07:01 |  |  |  |  |  |  |  |  |  |  |  |  |  |  |
| S1220-1228 | 1220 | 1220-1228 | FIAGLIAIV | FIAGLIAIV | HLA-A*02:01 | 1 | 1 | 1 | 1 | 1 | 1 | 1 | 1 | 1 | 0 | - | 1 | 1 | 1 |
|  |  |  |  |  | HLA-A*02:03 |  |  |  |  |  |  |  |  |  |  |  |  |  |  |
|  |  |  |  |  | HLA-A*02:05 |  |  |  |  |  |  |  |  |  |  |  |  |  |  |
|  |  |  |  |  | HLA-A*02:06 |  |  |  |  |  |  |  |  |  |  |  |  |  |  |
| S1225-1233 | 1225 | 1225-1233 | IAIVMVTIM | IAIVMVTIM | HLA-B*46:01 | 1 | 1 | 1 | 1 | 1 | 1 | 1 | 1 | 1 | 0 | - | 1 | 1 | 1 |
| S1257-1265 | 1257 | 1257-1265 | DEDDSEPVL | DEDDSEPVL | HLA-B*18:01 | 1 | 1 | 1 | 1 | 1 | 1 | 1 | 1 | 1 | 1 | - | 1 | 0 | 0 |
| S1262-1270 | 1262 | 1262-1270 | EPVLKGV**K**L | EPVLKGVKL | HLA-B*07:02 | 1 | 1 | 1 | 1 | 1 | 1 | 1 | 1 | 1 | 0 | - | 1 | 0 | 0 |
|  |  |  |  |  | HLA-B*08:01 |  |  |  |  |  |  |  |  |  |  |  |  |  |  |
|  |  |  | EPVLKGV**E**L | EPVLKGVEL | HLA-B*07:02 | 0 | 0 | 0 | 0 | 0 | 0 | 0 | 0 | 0 | 1 | - | 0 | 0 | 0 |
|  |  |  |  |  | HLA-B*08:01 |  |  |  |  |  |  |  |  |  |  |  |  |  |  |
|  |  |  |  |  | HLA-B*35:01 |  |  |  |  |  |  |  |  |  |  |  |  |  |  |
| S1264-1272 | 1264 | 1264-1272 | VLKGV**K**LHY | VLKGVKLHY | HLA-B*15:01 | 1 | 1 | 1 | 1 | 1 | 1 | 1 | 1 | 1 | 0 | - | 1 | 0 | 0 |
|  |  |  |  |  | HLA-B*46:01 |  |  |  |  |  |  |  |  |  |  |  |  |  |  |
|  |  |  | VLKGV**E**LHY | VLKGVELHY | HLA-B*15:01 | 0 | 0 | 0 | 0 | 0 | 0 | 0 | 0 | 0 | 1 | - | 0 | 0 | 0 |
|  |  |  |  |  | HLA-B*46:01 |  |  |  |  |  |  |  |  |  |  |  |  |  |  |

**Supplementary Table 9:** Epitope mapping of the patients sample sequences for CD8+ T-cell for 10-mer peptide length.

| CODE | S_POS | POS | EPITOPE | CORE | MHC | SARS_CoV_2_REF | S_N411 | S_N413 | S_N414 | S_N415 | S_N417 | S_N424 | S_N425 | S_N436 | S_N442 | S_N445 | S_N448 | S_N451 | S_N454 |
| --- | --- | --- | --- | --- | --- | --- | --- | --- | --- | --- | --- | --- | --- | --- | --- | --- | --- | --- | --- |
| S16-28 | 16 | 16-28 (DELETION AT 24,25,26) | VNLITRTQSY | VLITRTQSY | HLA-B*15:01 | 0 | 1 | 1 | 1 | 1 | 1 | 1 | 1 | 1 | 0 | - | 1 | 1 | 0 |
| S23-32 | 23 | 23-32 | QLPPAYTNSF | QLPPAYTSF | HLA-A*24:02 | 1 | 0 | 0 | 0 | 0 | 0 | 0 | 0 | 0 | 0 | - | 0 | 0 | 0 |
|  |  |  |  | QLPPATNSF | HLA-C*01:02 | 1 | 0 | 0 | 0 | 0 | 0 | 0 | 0 | 0 | 0 | - | 0 | 0 | 0 |
| S28-37 | 28 | 28-37 | YTNSFTRGVY | YTNSFTRVY | HLA-B*46:01 | 1 | 1 | 1 | 1 | 1 | 1 | 1 | 1 | 1 | 0 | - | 0 | 1 | 0 |
| S35-44 | 35 | 35-44 | GVYYPDKVFR | GVYPDKVFR | HLA-A*11:01 | 1 | 1 | 1 | 1 | 1 | 1 | 1 | 1 | 1 | 0 | - | 0 | 0 | 0 |
| S38-47 | 38 | 38-47 | YPDKVFRSSV | YPDKVRSSV | HLA-B*07:02 | 1 | 1 | 1 | 1 | 1 | 1 | 1 | 1 | 1 | 0 | - | 0 | 0 | 0 |
|  |  |  | YPDKVFRSSV | YPDKVFRSV | HLA-B*51:01 | 1 | 1 | 1 | 1 | 1 | 1 | 1 | 1 | 1 | 0 | - | 0 | 0 | 0 |
| S55-64 | 55 | 55-64 | FLPFFSNVTW | FPFFSNVTW | HLA-B*35:01 | 1 | 1 | 1 | 1 | 1 | 1 | 1 | 1 | 1 | 0 | - | 0 | 0 | 0 |
| S56-65 | 56 | 56-65 | LPFFSNVTWF | LPFFSNVTF | HLA-B*35:01 | 1 | 1 | 1 | 1 | 1 | 1 | 1 | 1 | 1 | 0 | - | 0 | 0 | 0 |
| S77-86 | 77 | 77-86 | KRFDNPVLPF | KFDNPVLPF | HLA-C*04:01 | 1 | 1 | 1 | 0 | 0 | 1 | 0 | 1 | 0 | 0 | - | 0 | 0 | 0 |
|  |  |  |  | KRFDNPVLF | HLA-C*06:02 | 1 | 1 | 1 | 0 | 0 | 1 | 0 | 1 | 0 | 0 | - | 0 | 0 | 0 |
|  |  |  |  |  | HLA-C*07:01 |  |  |  |  |  |  |  |  |  |  |  |  |  |  |
| S83-92 | 83 | 83-92 | VLPFNDGVYF | VPFNDGVYF | HLA-B*35:01 | 1 | 1 | 1 | 0 | 0 | 1 | 0 | 1 | 0 | 0 | - | 0 | 0 | 0 |
| S135-144 | 135 | 135-144 | FCNDPFLGVY | FCNDPFLVY | HLA-B*46:01 | 1 | 0 | 0 | 0 | 0 | 0 | 0 | 0 | 0 | 0 | - | 0 | 0 | 0 |
| S143-152 | 143 | 143-152 | VYYHKNNKSW | VYYHKNNSW | HLA-A*24:02 | 1 | 0 | 0 | 0 | 0 | 0 | 0 | 0 | 0 | 0 | - | 0 | 0 | 0 |
| S159-168 | 159 | 159-168 | VYSSANNCTF | VYSSANNTF | HLA-A*24:02 | 1 | 1 | 1 | 1 | 1 | 1 | 1 | 1 | 1 | 0 | - | 0 | 0 | 0 |
| S175-184 | 175 | 175-184 | FLMDLEGKEV | FLMDEGKEV | HLA-A*02:01 | 0 | 0 | 0 | 0 | 0 | 0 | 0 | 1 | 0 | 0 | - | 0 | 0 | 0 |
|  |  |  |  |  | HLA-A*02:03 |  |  |  |  |  |  |  |  |  |  |  |  |  |  |
|  |  |  |  |  | HLA-A*02:05 |  |  |  |  |  |  |  |  |  |  |  |  |  |  |
|  |  |  |  |  | HLA-A*02:06 |  |  |  |  |  |  |  |  |  |  |  |  |  |  |
| S203-212 | 203 | 203-212 | IYSKHTPINL | IYSKTPINL | HLA-A*24:02 | 1 | 1 | 1 | 1 | 1 | 1 | 1 | 1 | 1 | 1 | - | 1 | 1 | 0 |
| S211-220 | 211 | 211-220 | NLERDLPQGF | NERDLPQGF | HLA-B*18:01 | 0 | 1 | 1 | 1 | 1 | 1 | 1 | 1 | 1 | 1 | - | 1 | 1 | 0 |
| S214-223 | 214 | 214-223 | RDLPQGFSAL | RLPQGFSAL | HLA-C*01:02 | 1 | 1 | 1 | 1 | 1 | 1 | 1 | 1 | 1 | 1 | - | 1 | 1 | 0 |
| S229-238 | 229 | 229-238 | LPIGINITRF | LPIGINITF | HLA-B*35:01 | 1 | 1 | 1 | 1 | 1 | 1 | 1 | 1 | 1 | 1 | - | 1 | 1 | 1 |
| S237-248 | 237 | 237-248 (DELETION AT 243,244) | RFQTLLHRSY | RQTLLHRSY | HLA-B*15:01 | 0 | 1 | 0 | 0 | 0 | 0 | 0 | 0 | 0 | 0 | - | 0 | 0 | 0 |
| S243-252 | 243 | 243-252 | ALHRSYLTPV | ALHSYLTPV | HLA-A*02:03 | 0 | 0 | 1 | 0 | 0 | 1 | 0 | 0 | 1 | 0 | - | 0 | 0 | 1 |
| S266-275 | 266 | 266-275 | YVGYLQPRTF | YVYLQPRTF | HLA-B*46:01 | 1 | 1 | 1 | 1 | 1 | 1 | 1 | 1 | 1 | 1 | - | 1 | 1 | 1 |
| S267-276 | 267 | 267-276 | VGYLQPRTFL | VYLQPRTFL | HLA-A*24:02 | 1 | 1 | 1 | 1 | 1 | 1 | 1 | 1 | 1 | 1 | - | 1 | 1 | 1 |
| S268-277 | 268 | 268-277 | GYLQPRTFLL | GYLPRTFLL | HLA-A*24:02 | 1 | 1 | 1 | 1 | 1 | 1 | 1 | 1 | 1 | 1 | - | 1 | 1 | 1 |
| S270-279 | 270 | 270-279 | LQPRTFLLKY | LQRTFLLKY | HLA-B*15:01 | 1 | 1 | 1 | 1 | 1 | 1 | 1 | 1 | 1 | 1 | - | 1 | 1 | 1 |
| S319-328 | 319 | 319-328 | RVQPIESIVR | RVQPISIVR | HLA-A*11:01 | 0 | 0 | 0 | 1 | 0 | 0 | 0 | 0 | 0 | 0 | - | 0 | 0 | 0 |
| S320-329 | 320 | 320-329 | VQP**I**ESIVRF | VQIESIVRF | HLA-B*15:01 | 0 | 0 | 0 | 1 | 0 | 0 | 0 | 0 | 0 | 0 | - | 0 | 0 | 0 |
|  |  |  |  | VPIESIVRF | HLA-B*35:01 |  |  |  |  |  |  |  |  |  |  |  |  |  |  |
| S320-329 | 320 | 320-329 | VQP**T**ESIVRF | VQTESIVRF | HLA-B*15:01 | 1 | 1 | 1 | 0 | 1 | 1 | 1 | 1 | 1 | 0 | - | 1 | 1 | 0 |
| S326-335 | 326 | 326-335 | IVRFPNITNL | IRFPNITNL | HLA-C*06:02 | 1 | 1 | 1 | 1 | 1 | 1 | 1 | 1 | 1 | 0 | - | 1 | 1 | 0 |
|  |  |  |  |  | HLA-C*07:01 |  |  |  |  |  |  |  |  |  |  |  |  |  |  |
| S329-338 | 329 | 329-338 | FPNITNLCPF | FPNITNLPF | HLA-B*35:01 | 1 | 1 | 1 | 1 | 1 | 1 | 1 | 1 | 1 | 0 | - | 1 | 1 | 0 |
| S339-348 | 339 | 339-348 | **H**EVFNAT**T**FA | HEFNATTFA | HLA-B*40:06 | 0 | 1 | 1 | 1 | 1 | 1 | 1 | 1 | 1 | 0 | - | 1 | 1 | 0 |
|  |  |  | **G**EVFNAT**R**FA | GEFNATRFA | HLA-B*40:06 | 1 | 0 | 0 | 0 | 0 | 0 | 0 | 0 | 0 | 0 | - | 0 | 0 | 0 |
| S342-351 | 342 | 342-351 | FNATTFASVY | FATTFASVY | HLA-B*46:01 | 0 | 1 | 1 | 1 | 1 | 1 | 1 | 1 | 1 | 0 | - | 1 | 1 | 0 |
| S368-377 | 368 | 368-377 | **I**YN**F**A**P**F**FA**F | IYNFAPFFF | HLA-A*24:02 | 0 | 1 | 1 | 1 | 1 | 1 | 1 | 1 | 1 | 0 | - | 1 | 1 | 1 |
|  |  |  | **L**YN**S**A**S**F**ST**F | LYNSASFTF | HLA-A*24:02 | 1 | 0 | 0 | 0 | 0 | 0 | 0 | 0 | 0 | 0 | - | 0 | 0 | 0 |
| S369-378 | 369 | 369-378 | YNSASFSTFK | YSASFSTFK | HLA-A*11:01 | 1 | 0 | 0 | 0 | 0 | 0 | 0 | 0 | 0 | 0 | - | 0 | 0 | 0 |
| S386-395 | 386 | 386-395 | KLNDLCFTNV | KLNDLFTNV | HLA-A*02:01 | 1 | 1 | 1 | 1 | 1 | 1 | 1 | 1 | 1 | 0 | - | 1 | 1 | 1 |
|  |  |  |  |  | HLA-A*02:03 |  |  |  |  |  |  |  |  |  |  |  |  |  |  |
| S408-417 | 408 | 408-417 | RQIAPGQTGK | RIAPGQTGK | HLA-A*11:01 | 1 | 0 | 0 | 0 | 0 | 0 | 0 | 0 | 0 | 0 | - | 0 | 0 | 0 |
| S424-433 | 424 | 424-433 | KLPDDFTGCV | KLPDDFTGV | HLA-A*02:01 | 1 | 1 | 1 | 1 | 1 | 1 | 1 | 1 | 1 | 0 | - | 1 | 1 | 1 |
|  |  |  |  |  | HLA-A*02:03 |  |  |  |  |  |  |  |  |  |  |  |  |  |  |
|  |  |  |  |  | HLA-A*02:05 |  |  |  |  |  |  |  |  |  |  |  |  |  |  |
|  |  |  |  |  | HLA-A*02:06 |  |  |  |  |  |  |  |  |  |  |  |  |  |  |
| S464-473 | 464 | 464-473 | FERDISTEIY | FERDISTEY | HLA-B*18:01 | 1 | 1 | 1 | 1 | 1 | 1 | 1 | 1 | 1 | 0 | - | 1 | 0 | 0 |
| S470-479 | 470 | 470-479 | TEIYQAG**NQ**P | TEYQAGNQP | HLA-B*40:06 | 0 | 1 | 0 | 0 | 0 | 0 | 0 | 0 | 0 | 0 | - | 0 | 0 | 0 |
|  |  |  | TEIYQAG**ST**P | TEYQAGSTP | HLA-B*40:06 | 1 | 0 | 0 | 0 | 0 | 0 | 0 | 0 | 0 | 0 | - | 0 | 0 | 0 |
| S488-497 | 488 | 488-497 | CY**S**PLQSYGF | CYSPLQSYF | HLA-A*24:02 | 0 | 1 | 1 | 1 | 1 | 1 | 1 | 1 | 1 | 0 | - | 1 | 0 | 0 |
|  |  |  | CY**F**PLQSYGF | CYFPLQSYF | HLA-A*24:02 | 1 | 0 | 0 | 0 | 0 | 0 | 0 | 0 | 0 | 0 | - | 0 | 0 | 0 |
| S492-501 | 492 | 492-501 | LQSYGFRPTY | LQYGFRPTY | HLA-B*15:01 | 0 | 1 | 1 | 1 | 1 | 1 | 1 | 1 | 1 | 0 | - | 0 | 0 | 0 |
| S506-515 | 506 | 506-515 | QPYRVVVLSF | QPRVVVLSF | HLA-B*07:02 | 1 | 1 | 1 | 1 | 1 | 1 | 1 | 1 | 1 | 0 | - | 1 | 0 | 0 |
| S515-524 | 515 | 515-524 | FELLHAPATV | FLLHAPATV | HLA-A*02:01 | 1 | 0 | 0 | 0 | 0 | 0 | 0 | 0 | 1 | 0 | - | 0 | 0 | 0 |
|  |  |  |  |  | HLA-A*02:03 |  |  |  |  |  |  |  |  |  |  |  |  |  |  |
| S520-529 | 520 | 520-529 | A**S**ATVCGPKK | ASATVGPKK | HLA-A*11:01 | 0 | 1 | 0 | 1 | 1 | 0 | 1 | 1 | 0 | 1 | - | 1 | 1 | 0 |
|  |  |  | A**T**ATVCGPKK | ATATVGPKK | HLA-A*11:01 | 0 | 0 | 1 | 0 | 0 | 1 | 0 | 0 | 0 | 0 | - | 0 | 0 | 0 |
| S524-533 | 524 | 524-533 | VCGPKKSTNL | VGPKKSTNL | HLA-C*01:02 | 1 | 1 | 1 | 1 | 1 | 1 | 1 | 1 | 1 | 1 | - | 1 | 1 | 0 |
| S576-585 | 576 | 576-585 | VRDPQTLEIL | VRDPQTLEL | HLA-C*07:01 | 1 | 1 | 1 | 1 | 1 | 1 | 1 | 1 | 1 | 1 | - | 1 | 1 | 1 |
| S608-617 | 608 | 608-617 | VATTHMSVTY | VATTHSVTY | HLA-B*46:01 | 0 | 0 | 0 | 0 | 0 | 0 | 0 | 0 | 0 | 1 | - | 0 | 0 | 1 |
| S626-635 | 626 | 626-635 | ADQLTPTWRV | AQLTPTWRV | HLA-B*13:02 | 1 | 1 | 1 | 1 | 1 | 1 | 1 | 1 | 1 | 1 | - | 1 | 1 | 0 |
| S634-643 | 634 | 634-643 | RVYSTGSNVF | RYSTGSNVF | HLA-A*24:02 | 1 | 1 | 1 | 1 | 1 | 1 | 1 | 1 | 1 | 1 | - | 1 | 1 | 0 |
|  |  |  |  | RVYSGSNVF | HLA-B*15:01 | 1 | 1 | 1 | 1 | 1 | 1 | 1 | 1 | 1 | 1 | - | 1 | 1 | 0 |
|  |  |  |  |  | HLA-B*46:01 |  |  |  |  |  |  |  |  |  |  |  |  |  |  |
| S659-668 | 659 | 659-668 | SYECDIPIGA | SECDIPIGA | HLA-B*40:06 | 1 | 1 | 1 | 1 | 1 | 1 | 1 | 1 | 1 | 1 | - | 1 | 1 | 1 |
| S686-695 | 686 | 686-695 | SVASQSIIAY | SVAQSIIAY | HLA-B*15:01 | 1 | 1 | 1 | 1 | 1 | 1 | 1 | 1 | 1 | 0 | - | 1 | 1 | 1 |
|  |  |  |  | SASQSIIAY | HLA-B*35:01 | 1 | 1 | 1 | 1 | 1 | 1 | 1 | 1 | 1 | 0 | - | 1 | 1 | 1 |
|  |  |  |  |  | HLA-B*46:01 |  |  |  |  |  |  |  |  |  |  |  |  |  |  |
| S698-707 | 698 | 698-707 | SLGAENSVAY | SLAENSVAY | HLA-B*15:01 | 1 | 1 | 1 | 1 | 1 | 1 | 1 | 1 | 1 | 0 | - | 1 | 1 | 0 |
|  |  |  |  |  | HLA-B*46:01 |  |  |  |  |  |  |  |  |  |  |  |  |  |  |
| S724-733 | 724 | 724-733 | TEILPVSMTK | TILPVSMTK | HLA-A*11:01 | 1 | 1 | 1 | 1 | 1 | 1 | 1 | 1 | 1 | 0 | - | 1 | 1 | 0 |
| S727-736 | 727 | 727-736 | LPVSMTKTSV | LPVSMTKTV | HLA-B*51:01 | 1 | 1 | 1 | 1 | 1 | 1 | 1 | 1 | 1 | 0 | - | 1 | 1 | 0 |
| S747-756 | 747 | 747-756 | TECSNLLLQY | TESNLLLQY | HLA-B*18:01 | 1 | 1 | 1 | 1 | 1 | 1 | 1 | 1 | 1 | 1 | - | 1 | 1 | 0 |
| S787-796 | 787 | 787-796 | QIYKTPPIKY | QIYKPPIKY | HLA-B*15:01 | 0 | 1 | 1 | 1 | 1 | 1 | 1 | 1 | 1 | 0 | - | 1 | 0 | 0 |
| S788-797 | 788 | 788-797 | IYKTPPIK**Y**F | IYTPPIKYF | HLA-A*24:02 | 0 | 1 | 1 | 1 | 1 | 1 | 1 | 1 | 1 | 0 | - | 1 | 0 | 0 |
|  |  |  |  | IYKPPIKYF | HLA-C*07:01 | 0 | 1 | 1 | 1 | 1 | 1 | 1 | 1 | 1 | 0 | - | 1 | 0 | 0 |
|  |  |  | IYKTPPIK**D**F | IYKTPPIKF | HLA-A*24:02 | 1 | 0 | 0 | 0 | 0 | 0 | 0 | 0 | 0 | 1 | - | 0 | 0 | 0 |
| S826-835 | 826 | 826-835 | VTLADAGFIK | VTLDAGFIK | HLA-A*11:01 | 1 | 1 | 1 | 1 | 1 | 1 | 1 | 1 | 1 | 1 | - | 1 | 1 | 0 |
| S852-861 | 852 | 852-861 | AQK**F**NGLTVL | AQFNGLTVL | HLA-B*13:02 | 1 | 1 | 1 | 1 | 1 | 1 | 1 | 1 | 1 | 1 | - | 1 | 0 | 0 |
|  |  |  |  |  | HLA-B*15:01 |  |  |  |  |  |  |  |  |  |  |  |  |  |  |
|  |  |  | AQK**V**NGLTVL | AQKNGLTVL | HLA-B*15:01 | 0 | 0 | 0 | 0 | 0 | 0 | 0 | 0 | 0 | 0 | - | 0 | 1 | 0 |
| S860-869 | 860 | 860-869 | VLPPLLTDEM | VLPPLLTEM | HLA-C*01:02 | 1 | 1 | 1 | 1 | 1 | 1 | 1 | 1 | 1 | 1 | - | 1 | 1 | 0 |
| S864-873 | 864 | 864-873 | LQEDEMIAQY | LQEDMIAQY | HLA-B*15:01 | 0 | 0 | 0 | 0 | 0 | 0 | 0 | 0 | 0 | 0 | - | 0 | 0 | 1 |
| S895-904 | 895 | 895-904 | QIPFAMQMAY | QPFAMQMAY | HLA-B*35:01 | 1 | 1 | 1 | 1 | 1 | 1 | 1 | 1 | 1 | 1 | - | 1 | 1 | 1 |
| S924-933 | 924 | 924-933 | ANQFNSAIGK | AQFNSAIGK | HLA-A*11:01 | 1 | 1 | 1 | 1 | 1 | 1 | 1 | 1 | 1 | 1 | - | 1 | 1 | 1 |
| S925-934 | 925 | 925-934 | NQFNSAIGKI | NQFNSAIKI | HLA-B*13:02 | 1 | 1 | 1 | 1 | 1 | 1 | 1 | 1 | 1 | 1 | - | 1 | 1 | 1 |
| S947-956 | 947 | 947-956 | KLQDVVN**H**N**A** | KLQDVVHNA | HLA-A*02:01 | 0 | 1 | 1 | 1 | 1 | 1 | 1 | 1 | 1 | 1 | - | 1 | 1 | 0 |
|  |  |  |  |  | HLA-A*02:03 |  |  |  |  |  |  |  |  |  |  |  |  |  |  |
|  |  |  | KLQDVVN**E**N**F** | KLQDVVNEF | HLA-B*15:01 | 0 | 0 | 0 | 0 | 0 | 0 | 0 | 0 | 0 | 0 | - | 0 | 0 | 1 |
|  |  |  | KLQDVVN**Q**N**A** | KLQDVVQNA | HLA-A*02:03 | 1 | 0 | 0 | 0 | 0 | 0 | 0 | 0 | 0 | 0 | - | 0 | 0 | 0 |
| S964-973 | 964 | 964-973 | KQLSS**K**FGAI | KQLSSKFAI | HLA-B*13:02 | 0 | 1 | 1 | 1 | 1 | 1 | 1 | 1 | 1 | 1 | - | 1 | 1 | 0 |
|  |  |  | KQLSS**N**FGAI | KQLSSNFAI | HLA-B*13:02 | 1 | 0 | 0 | 0 | 0 | 0 | 0 | 0 | 0 | 0 | - | 0 | 0 | 0 |
| S975-984 | 975 | 975-984 | SVLNDILSRL | SLNDILSRL | HLA-A*02:03 | 1 | 1 | 1 | 1 | 1 | 1 | 1 | 1 | 1 | 1 | - | 1 | 1 | 1 |
|  |  |  |  |  | HLA-A*02:05 |  |  |  |  |  |  |  |  |  |  |  |  |  |  |
| S995-1004 | 995 | 995-1004 | RLITGRLQSL | RLIGRLQSL | HLA-A*02:03 | 1 | 1 | 1 | 1 | 1 | 1 | 1 | 1 | 1 | 1 | - | 1 | 1 | 1 |
| S1016-1025 | 1016 | 1016-1025 | AEIR**A**SANLA | AEIRASNLA | HLA-B*40:06 | 1 | 1 | 1 | 0 | 1 | 1 | 1 | 1 | 1 | 1 | - | 1 | 1 | 1 |
|  |  |  | AEIR**V**SANLA | AEIRVSNLA | HLA-B*40:06 | 0 | 0 | 0 | 1 | 0 | 0 | 0 | 0 | 0 | 0 | - | 0 | 0 | 0 |
| S1019-1028 | 1019 | 1019-1028 | R**A**SANLAATK | RSANLAATK | HLA-A*11:01 | 1 | 1 | 1 | 0 | 1 | 1 | 1 | 1 | 1 | 1 | - | 1 | 1 | 1 |
|  |  |  | R**V**SANLAATK | RVANLAATK | HLA-A*11:01 | 0 | 0 | 0 | 1 | 0 | 0 | 0 | 0 | 0 | 0 | - | 0 | 0 | 0 |
| S1047-1056 | 1047 | 1047-1056 | YHLMSFPQSA | YLMSFPQSA | HLA-A*02:01 | 1 | 1 | 1 | 1 | 1 | 1 | 1 | 1 | 1 | 1 | - | 1 | 1 | 1 |
|  |  |  |  |  | HLA-A*02:03 |  |  |  |  |  |  |  |  |  |  |  |  |  |  |
| S1052-1061 | 1052 | 1052-1061 | FPQSAPHGVV | FPQSAPHVV | HLA-B*51:01 | 1 | 1 | 1 | 1 | 1 | 1 | 1 | 1 | 1 | 1 | - | 1 | 1 | 1 |
| S1054-1063 | 1054 | 1054-1063 | QSAPHGVVFL | QAPHGVVFL | HLA-C*01:02 | 1 | 1 | 1 | 1 | 1 | 1 | 1 | 1 | 1 | 1 | - | 1 | 1 | 1 |
| S1064-1073 | 1064 | 1064-1073 | HVTYVPAQEK | HTYVPAQEK | HLA-A*11:01 | 1 | 1 | 1 | 1 | 1 | 1 | 1 | 1 | 1 | 1 | - | 1 | 1 | 1 |
| S1066-1075 | 1066 | 1066-1075 | TYVPAQEKNF | TYVPAQEKF | HLA-A*24:02 | 1 | 1 | 1 | 1 | 1 | 1 | 1 | 1 | 1 | 1 | - | 1 | 1 | 1 |
| S1070-1079 | 1070 | 1070-1079 | AQEKNFTTAP | AEKNFTTAP | HLA-B*40:06 | 1 | 1 | 1 | 1 | 1 | 1 | 1 | 1 | 1 | 1 | - | 1 | 1 | 1 |
| S1071-1080 | 1071 | 1071-1080 | QEKNFTTAPA | QEKNFTTAA | HLA-B*40:06 | 1 | 1 | 1 | 1 | 1 | 1 | 1 | 1 | 1 | 1 | - | 1 | 1 | 1 |
| S1094-1103 | 1094 | 1094-1103 | VFVSNGTHWF | VFVSNGTHF | HLA-A*24:02 | 1 | 1 | 1 | 1 | 1 | 1 | 1 | 1 | 1 | 1 | - | 1 | 1 | 1 |
| S1136-1145 | 1136 | 1136-1145 | TVYDPLQPEL | TVYDPLQEL | HLA-A*02:03 | 1 | 1 | 1 | 1 | 1 | 1 | 1 | 1 | 1 | 1 | - | 1 | 1 | 1 |
|  |  |  |  |  | HLA-A*02:05 |  |  |  |  |  |  |  |  |  |  |  |  |  |  |
|  |  |  |  | TVYDPLPEL | HLA-A*02:06 | 1 | 1 | 1 | 1 | 1 | 1 | 1 | 1 | 1 | 1 | - | 1 | 1 | 1 |
|  |  |  |  | TYDPLQPEL | HLA-C*04:01 | 1 | 1 | 1 | 1 | 1 | 1 | 1 | 1 | 1 | 1 | - | 1 | 1 | 1 |
| S1137-1146 | 1137 | 1137-1146 | VYDPLQPELD | VYDPLQPEL | HLA-C*04:01 | 1 | 1 | 1 | 1 | 1 | 1 | 1 | 1 | 1 | 1 | - | 1 | 1 | 1 |
| S1139-1148 | 1139 | 1139-1148 | DPLQPELDSF | DPLQPELSF | HLA-B*35:01 | 1 | 1 | 1 | 1 | 1 | 1 | 1 | 1 | 1 | 1 | - | 1 | 1 | 1 |
| S1181-1190 | 1181 | 1181-1190 | KEIDRLNEVA | KEIDLNEVA | HLA-B*40:06 | 1 | 1 | 1 | 1 | 1 | 1 | 1 | 1 | 1 | 0 | - | 1 | 1 | 1 |
| S1196-1205 | 1196 | 1196-1205 | SLIDLQELGK | SLIDLQLGK | HLA-A*11:01 | 1 | 1 | 1 | 1 | 1 | 1 | 1 | 1 | 1 | 0 | - | 1 | 1 | 1 |
| S1200-1209 | 1200 | 1200-1209 | LQELGKYEQY | LQLGKYEQY | HLA-B*15:01 | 1 | 1 | 1 | 1 | 1 | 1 | 1 | 1 | 1 | 0 | - | 1 | 1 | 1 |
| S1208-1217 | 1208 | 1208-1217 | QYIKWPWYIW | QYIKWPYIW | HLA-A*24:02 | 1 | 1 | 1 | 1 | 1 | 1 | 1 | 1 | 1 | 0 | - | 1 | 1 | 1 |
| S1211-1220 | 1211 | 1211-1220 | KWPWYIWLGF | KWPWYIWLF | HLA-A*24:02 | 1 | 1 | 1 | 1 | 1 | 1 | 1 | 1 | 1 | 0 | - | 1 | 1 | 1 |
| S1261-1270 | 1261 | 1261-1270 | SEPVLKGV**K**L | SPVLKGVKL | HLA-B*07:02 | 1 | 1 | 1 | 1 | 1 | 1 | 1 | 1 | 1 | 0 | - | 1 | 0 | 0 |
|  |  |  | SEPVLKGV**E**L | SPVLKGVEL | HLA-B*07:02 | 0 | 0 | 0 | 0 | 0 | 0 | 0 | 0 | 0 | 1 | - | 0 | 0 | 0 |

**Supplementary Table 10:** Epitope mapping of the patient’s sample sequences for CD8+ T-cell for 11-mer peptide length.

| CODE | S_POS | POS | EPITOPE | CORE | MHC | SARS_CoV_2_REF | S_N411 | S_N413 | S_N414 | S_N415 | S_N417 | S_N424 | S_N425 | S_N436 | S_N442 | S_N445 | S_N448 | S_N451 | S_N454 |
| --- | --- | --- | --- | --- | --- | --- | --- | --- | --- | --- | --- | --- | --- | --- | --- | --- | --- | --- | --- |
| S21-34 | 21 | 21-34 (DELETION AT 24,25,26) | RTQSYTNSFTR | RTYTNSFTR | HLA-A*11:01 | 0 | 1 | 1 | 1 | 1 | 1 | 1 | 1 | 1 | 0 | - | 1 | 1 | 0 |
| S38-48 | 38 | 38-48 | YPDKVFRSSVL | YPDKRSSVL | HLA-B*07:02 | 1 | 1 | 1 | 1 | 1 | 1 | 1 | 1 | 1 | 0 | - | 0 | 0 | 0 |
|  |  |  |  |  | HLA-B*08:01 |  |  |  |  |  |  |  |  |  |  |  |  |  |  |
|  |  |  |  | YPDKVSSVL | HLA-C*04:01 | 1 | 1 | 1 | 1 | 1 | 1 | 1 | 1 | 1 | 0 | - | 0 | 0 | 0 |
| S54-64 | 54 | 54-64 | LFLPFFSNVTW | LFLPSNVTW | HLA-A*24:02 | 1 | 1 | 1 | 1 | 1 | 1 | 1 | 1 | 1 | 0 | - | 0 | 0 | 0 |
| S81-91 | 81 | 81-91 | NPVLPFNDGVY | NPVLPFNVY | HLA-B*35:01 | 1 | 1 | 1 | 0 | 0 | 1 | 0 | 1 | 0 | 0 | - | 0 | 0 | 0 |
| S153-163 | 153 | 153-163 | MESEFRVYSSA | MESEFRVSA | HLA-B*40:06 | 1 | 1 | 1 | 1 | 1 | 1 | 1 | 1 | 1 | 1 | - | 1 | 0 | 0 |
| S158-168 | 158 | 158-168 | RVYSSANNCTF | RVYSSANTF | HLA-B*15:01 | 1 | 1 | 1 | 1 | 1 | 1 | 1 | 1 | 1 | 0 | - | 0 | 0 | 0 |
| S182-192 | 182 | 182-192 | EEVNFKNLREF | EEVNFKNEF | HLA-B*18:01 | 0 | 0 | 0 | 0 | 0 | 0 | 0 | 0 | 0 | 0 | - | 1 | 0 | 0 |
| S212-222 | 212 | 212-222 | LERDLPQGFSA | LERDLPQSA | HLA-B*40:06 | 0 | 1 | 1 | 1 | 1 | 1 | 1 | 1 | 1 | 1 | - | 1 | 1 | 0 |
| S236-248 | 236 | 236-248 (DELETION AT 243,244) | TRFQTLLHRSY | TRFQTHRSY | HLA-C*07:01 | 0 | 1 | 0 | 0 | 0 | 0 | 0 | 0 | 0 | 0 | - | 0 | 0 | 0 |
| S265-275 | 265 | 265-275 | YYVGYLQPRTF | YYYLQPRTF | HLA-A*24:02 | 1 | 1 | 1 | 1 | 1 | 1 | 1 | 1 | 1 | 1 | - | 1 | 1 | 1 |
| S325-335 | 325 | 325-335 | SIVRFPNITNL | SRFPNITNL | HLA-C*06:02 | 1 | 1 | 1 | 1 | 1 | 1 | 1 | 1 | 1 | 0 | - | 1 | 1 | 0 |
|  |  |  |  |  | HLA-C*07:01 |  |  |  |  |  |  |  |  |  |  |  |  |  |  |
| S328-338 | 328 | 328-338 | RFPNITNLCPF | RFPNITNLF | HLA-A*24:02 | 1 | 1 | 1 | 1 | 1 | 1 | 1 | 1 | 1 | 0 | - | 1 | 1 | 0 |
| S337-347 | 337 | 337-347 | PFHEVFNATTF | PFHEVFNTF | HLA-A*24:02 | 0 | 1 | 1 | 1 | 1 | 1 | 1 | 1 | 1 | 0 | - | 1 | 1 | 0 |
| S386-396 | 386 | 386-396 | KLNDLCFTNVY | KLNDLTNVY | HLA-B*15:01 | 1 | 1 | 1 | 1 | 1 | 1 | 1 | 1 | 1 | 0 | - | 1 | 1 | 1 |
| S408-418 | 408 | 408-418 | SQIAPGQTGNI | SQIAPGQTI | HLA-B*13:02 | 0 | 1 | 1 | 1 | 1 | 1 | 1 | 1 | 1 | 0 | - | 1 | 1 | 1 |
|  |  |  |  |  | HLA-B*52:01 |  |  |  |  |  |  |  |  |  |  |  |  |  |  |
| S462-472 | 462 | 462-472 | KPFERDISTEI | KPRDISTEI | HLA-B*07:02 | 1 | 1 | 1 | 1 | 1 | 1 | 1 | 1 | 1 | 0 | - | 1 | 0 | 0 |
| S485-495 | 485 | 485-495 | GPNCYSPLQSY | GPYSPLQSY | HLA-B*35:01 | 0 | 1 | 1 | 1 | 1 | 1 | 1 | 1 | 1 | 0 | - | 1 | 0 | 0 |
| S523-533 | 523 | 523-533 | TVCGPKKSTNL | TVPKKSTNL | HLA-C*01:02 | 1 | 1 | 1 | 1 | 1 | 1 | 1 | 1 | 1 | 1 | - | 1 | 1 | 0 |
| S588-598 | 588 | 588-598 | TPCSFGGVSVI | TPFGGVSVI | HLA-B*51:01 | 1 | 1 | 1 | 1 | 1 | 1 | 1 | 1 | 1 | 1 | - | 1 | 1 | 1 |
| S610-620 | 610 | 610-620 | VLYQGVNCTEV | VLYQGVTEV | HLA-A*02:01 | 0 | 1 | 1 | 1 | 1 | 1 | 1 | 1 | 1 | 1 | - | 1 | 1 | 0 |
|  |  |  |  |  | HLA-A*02:03 |  |  |  |  |  |  |  |  |  |  |  |  |  |  |
| S664-674 | 664 | 664-674 | IPIGAGICASY | IPIGAGISY | HLA-B*35:01 | 1 | 1 | 1 | 1 | 1 | 1 | 1 | 1 | 1 | 1 | - | 1 | 1 | 1 |
| S697-707 | 697 | 697-707 | MSLGAENSVAY | MSAENSVAY | HLA-B*35:01 | 1 | 1 | 1 | 1 | 1 | 1 | 1 | 1 | 1 | 0 | - | 1 | 1 | 0 |
| S723-733 | 723 | 723-733 | TTEILPVSMTK | TTLPVSMTK | HLA-A*11:01 | 1 | 1 | 1 | 1 | 1 | 1 | 1 | 1 | 1 | 0 | - | 1 | 1 | 0 |
| S779-789 | 779 | 779-789 | QEVFAQVKQIY | QEVQVKQIY | HLA-B*18:01 | 1 | 1 | 1 | 1 | 1 | 1 | 1 | 1 | 1 | 1 | - | 1 | 0 | 0 |
| S786-796 | 786 | 786-796 | KQIYKTPPIKY | KQITPPIKY | HLA-B*15:01 | 0 | 1 | 1 | 1 | 1 | 1 | 1 | 1 | 1 | 0 | - | 1 | 0 | 0 |
| S795-805 | 795 | 795-805 | KYFGGFNFSQI | KYFGGFNQI | HLA-A*24:02 | 0 | 1 | 1 | 1 | 1 | 1 | 1 | 1 | 1 | 0 | - | 1 | 0 | 0 |
| S815-825 | 815 | 815-825 | RSFIEDLLFNK | RSFDLLFNK | HLA-A*11:01 | 1 | 1 | 1 | 1 | 1 | 1 | 1 | 1 | 1 | 1 | - | 1 | 0 | 0 |
| S825-835 | 825 | 825-835 | KVTLADAGFIK | KVADAGFIK | HLA-A*11:01 | 1 | 1 | 1 | 1 | 1 | 1 | 1 | 1 | 1 | 1 | - | 1 | 1 | 0 |
| S827-837 | 827 | 827-837 | TLADAGFIKQY | TLADAGKQY | HLA-B*15:01 | 1 | 1 | 1 | 1 | 1 | 1 | 1 | 1 | 1 | 1 | - | 1 | 1 | 0 |
| S894-904 | 894 | 894-904 | LQIPFAMQMAY | LQIPFQMAY | HLA--B*15:01 | 1 | 1 | 1 | 1 | 1 | 1 | 1 | 1 | 1 | 1 | - | 1 | 1 | 1 |
|  |  |  |  | LPFAMQMAY | HLA--B*35:01 | 1 | 1 | 1 | 1 | 1 | 1 | 1 | 1 | 1 | 1 | - | 1 | 1 | 1 |
| S911-921 | 911 | 911-921 | VTQNVLYENQK | VTQNVLNQK | HLA-A*11:01 | 1 | 1 | 1 | 1 | 1 | 1 | 1 | 1 | 1 | 1 | - | 1 | 1 | 1 |
| S917-927 | 917 | 917-927 | YENQKLIANQF | YENQIANQF | HLA-B*18:01 | 1 | 1 | 1 | 1 | 1 | 1 | 1 | 1 | 1 | 1 | - | 1 | 1 | 1 |
| S974-984 | 974 | 974-984 | SSVLNDILSRL | SLNDILSRL | HLA-A*02:03 | 1 | 1 | 1 | 1 | 1 | 1 | 1 | 1 | 1 | 1 | - | 1 | 1 | 1 |
| S1016-1026 | 1016 | 1016-1026 | AEIR**A**SANLAA | AEISANLAA | HLA-B*40:06 | 1 | 1 | 1 | 0 | 1 | 1 | 1 | 1 | 1 | 1 | - | 1 | 1 | 1 |
|  |  |  | AEIR**V**SANLAA | AEISANLAA | HLA-B*40:06 | 0 | 0 | 0 | 1 | 0 | 0 | 0 | 0 | 0 | 0 | - | 0 | 0 | 0 |
| S1052-1062 | 1052 | 1052-1062 | FPQSAPHGVVF | FPAPHGVVF | HLA-B*07:02 | 1 | 1 | 1 | 1 | 1 | 1 | 1 | 1 | 1 | 1 | - | 1 | 1 | 1 |
|  |  |  |  |  | HLA-B*35:01 |  |  |  |  |  |  |  |  |  |  |  |  |  |  |
|  |  |  |  |  | HLA-B*51:01 |  |  |  |  |  |  |  |  |  |  |  |  |  |  |
|  |  |  |  | FSAPHGVVF | HLA-B*46:01 | 1 | 1 | 1 | 1 | 1 | 1 | 1 | 1 | 1 | 1 |  | 1 | 1 | 1 |
| S1076-1086 | 1076 | 1076-1086 | TTAPAICHDGK | TTAPAICHK | HLA-A*11:01 | 1 | 1 | 1 | 1 | 1 | 1 | 1 | 1 | 1 | 0 | - | 1 | 1 | 1 |
| S1111-1121 | 1111 | 1111-1121 | EPQIITTDNTF | EPITTDNTF | HLA-B*35:01 | 1 | 1 | 1 | 1 | 1 | 1 | 1 | 1 | 1 | 1 | - | 1 | 1 | 1 |
| S1128-1138 | 1128 | 1128-1138 | VVIGIVNNTVY | VVIGNNTVY | HLA-B*15:01 | 1 | 1 | 1 | 1 | 1 | 1 | 1 | 1 | 1 | 1 | - | 1 | 1 | 1 |
| S1135-1145 | 1135 | 1135-1145 | NTVYDPLQPEL | NYDPLQPEL | HLA-C*04:01 | 1 | 1 | 1 | 1 | 1 | 1 | 1 | 1 | 1 | 1 | - | 1 | 1 | 1 |
| S1171-1181 | 1171 | 1171-1181 | GINASVVNIQK | GINASVIQK | HLA-A*11:01 | 1 | 1 | 1 | 1 | 1 | 1 | 1 | 1 | 1 | 0 | - | 1 | 1 | 1 |
| S1179-1189 | 1179 | 1179-1189 | IQKEIDRLNEV | IQIDRLNEV | HLA-A*02:05 | 1 | 1 | 1 | 1 | 1 | 1 | 1 | 1 | 1 | 0 | - | 1 | 1 | 1 |
| S1196-1206 | 1196 | 1196-1206 | SLIDLQELGKY | SLIDLQEKY | HLA-B*15:01 | 1 | 1 | 1 | 1 | 1 | 1 | 1 | 1 | 1 | 0 | - | 1 | 1 | 1 |
| S1199-1209 | 1199 | 1199-1209 | DLQELGKYEQY | DELGKYEQY | HLA-B*18:01 | 1 | 1 | 1 | 1 | 1 | 1 | 1 | 1 | 1 | 0 | - | 1 | 1 | 1 |
| S1208-1218 | 1208 | 1208-1218 | QYIKWPWYIWL | QYIKWPWYL | HLA-A*24:02 | 1 | 1 | 1 | 1 | 1 | 1 | 1 | 1 | 1 | 0 | - | 1 | 1 | 1 |
| S1255-1265 | 1255 | 1255-1265 | KFDEDDSEPVL | KFDDSEPVL | HLA-C*04:01 | 1 | 1 | 1 | 1 | 1 | 1 | 1 | 1 | 1 | 1 | - | 1 | 0 | 0 |

**Supplementary Table 11: B-cell prediction of the patient’s sample sequences.**

| **CODE** | **S_POS** | **POSTION** | **EPITOPE/PEPTIDE** | **NICPR411** | **NICPR413** | **NICPR414** | **NICPR415** | **NICPR417** | **NICPR424** | **NICPR425** | **NICPR436** | **NICPR442** | **NICPR445** | **NICPR448** | **NICPR451** | **NICPR454** | **TOTAL** |
| --- | --- | --- | --- | --- | --- | --- | --- | --- | --- | --- | --- | --- | --- | --- | --- | --- | --- |
| S5-22 | 5 | 5-22 | LVLLPLVSSQCVNLITRT | 1 | 1 | 1 | 1 | 1 | 1 | 1 | 1 | 0 | - | 1 | 1 | 0 | 10 |
| S14-34 | 14 | 14-34 | QCVNLITRTQSYTNSFTR | 1 | 1 | 1 | 1 | 1 | 1 | 1 | 1 | 0 | - | 1 | 1 | 0 | 10 |
| S21-41 | 21 | 21-41 | RTQSYTNSFTRGVYYPDK | 1 | 1 | 1 | 1 | 1 | 1 | 1 | 1 | 0 | - | 0 | 0 | 0 | 8 |
| S34-51 | 34 | 34-51 | RGVYYPDKVFRSSVLHST | 1 | 1 | 1 | 1 | 1 | 1 | 1 | 1 | 0 | - | 0 | 0 | 0 | 8 |
| S41-58 | 41 | 41-58 | KVFRSSVLHSTQDLFLPF | 1 | 1 | 1 | 1 | 1 | 1 | 1 | 1 | 0 | - | 0 | 0 | 0 | 8 |
| S50-67 | 50 | 50-67 | STQDLFLPFFSNVTWFHA | 1 | 1 | 1 | 1 | 1 | 1 | 1 | 1 | 0 | - | 0 | 0 | 0 | 8 |
| S74-91 | 74 | 74-91 | NGTKRFDNPVLPFNDGVY | 1 | 1 | 0 | 0 | 1 | 0 | 1 | 0 | 0 | - | 0 | 0 | 0 | 4 |
| S83-100 | 83 | 83-100 | VLPFNDGVYFASTEKSNI | 1 | 1 | 0 | 0 | 1 | 0 | 1 | 0 | 0 | - | 0 | 0 | 0 | 4 |
| S90-107 | 90 | 90-107 | VYFASTEKSNIIRGWIFG | 1 | 1 | 0 | 1 | 1 | 1 | 1 | 1 | 0 | - | 0 | 0 | 0 | 7 |
| S95-112 | 95 | 95-112 | TEKSNIIRGWIFGTTLDS | 0 | 0 | 0 | 0 | 0 | 0 | 0 | 0 | 1 | - | 0 | 0 | 0 | 1 |
| S100-117 | 100 | 100-117 | IIRGWIFGTTLDSKTQSL | 1 | 1 | 1 | 1 | 1 | 1 | 1 | 1 | 0 | - | 1 | 1 | 0 | 10 |
| S106-123 | 106 | 106-123 | FGTTLDSKTQSLLIVNNA | 1 | 1 | 1 | 1 | 1 | 1 | 1 | 1 | 1 | - | 1 | 1 | 0 | 11 |
| S117-138 | 117 | 117-138 | LLIVNNATNVVIKVCEFQ | 1 | 1 | 1 | 1 | 1 | 1 | 1 | 1 | 0 | - | 1 | 1 | 0 | 10 |
| S119-136 | 119 | 119-136 | IVNNATNVVIKVCEFQFC | 0 | 0 | 0 | 0 | 0 | 0 | 0 | 0 | 1 | - | 0 | 0 | 0 | 1 |
| S124-141 | 124 | 124-141 | TNVVIKVCEFQFCNDPFL | 0 | 1 | 1 | 1 | 1 | 1 | 1 | 1 | 0 | - | 1 | 1 | 0 | 9 |
| S124-148 | 124 | 124-148 | TNVVIKVCEFQFCNDQKN | 1 | 0 | 0 | 0 | 0 | 0 | 0 | 0 | 0 | - | 0 | 0 | 0 | 1 |
| S127-144 | 127 | 127-144 | VIKVCEFQFCNDPFLDVY | 0 | 0 | 0 | 0 | 0 | 0 | 0 | 0 | 1 | - | 0 | 0 | 0 | 1 |
| S135-153 | 135 | 135-153 | FCNDPFLDVYQKNNKSWM | 0 | 0 | 0 | 0 | 0 | 0 | 0 | 0 | 1 | - | 0 | 0 | 0 | 1 |
| S137-155 | 137 | 137-155 | NDPFLDVYQKNNKSWMES | 0 | 1 | 0 | 1 | 1 | 1 | 1 | 1 | 0 | - | 1 | 0 | 0 | 7 |
| S137-155 | 137 | 137-155 | NDPFLDVYKKNNKSWMES | 0 | 0 | 1 | 0 | 0 | 0 | 0 | 0 | 0 | - | 0 | 0 | 0 | 1 |
| S147-164 | 147 | 147-164 | KNNKSWMESEFRVYSSAN | 0 | 0 | 0 | 0 | 0 | 0 | 0 | 0 | 1 | - | 0 | 0 | 0 | 1 |
| S149-166 | 149 | 149-166 | NKSWMESEFRVYSSANNC | 1 | 1 | 1 | 1 | 1 | 1 | 1 | 1 | 0 | - | 1 | 0 | 0 | 9 |
| S156-173 | 156 | 156-173 | EFRVYSSANNCTFEYVSQ | 1 | 0 | 0 | 0 | 0 | 0 | 0 | 0 | 0 | - | 0 | 0 | 0 | 1 |
| S164-181 | 164 | 164-181 | NNCTFEYVSQPFLMDLEG | 1 | 0 | 0 | 0 | 0 | 0 | 0 | 0 | 0 | - | 0 | 0 | 0 | 1 |
| S169-186 | 169 | 169-186 | EYVSQPFLMDLVGKEGNF | 0 | 1 | 0 | 0 | 1 | 0 | 0 | 1 | 0 | - | 0 | 0 | 0 | 3 |
| S169-186 | 169 | 169-186 | EYVSQPFLMDLEGEEGNF | 0 | 0 | 1 | 0 | 0 | 0 | 0 | 0 | 0 | - | 0 | 0 | 0 | 1 |
| S169-186 | 169 | 169-186 | EYVSQPFLMDLEGKEGNF | 0 | 0 | 0 | 1 | 0 | 0 | 0 | 0 | 0 | - | 0 | 0 | 0 | 1 |
| S169-186 | 169 | 169-186 | EYVSQPFLMDLEGKEVNF | 0 | 0 | 0 | 0 | 0 | 0 | 1 | 0 | 0 | - | 0 | 0 | 0 | 1 |
| S169-186 | 169 | 169-186 | EYVSQPFLMDLEGNEGNF | 0 | 0 | 0 | 0 | 0 | 1 | 0 | 0 | 0 | - | 0 | 0 | 0 | 1 |
| S171-188 | 171 | 171-188 | VSQPFLMDLEGKEGNFKN | 1 | 0 | 0 | 0 | 0 | 0 | 0 | 0 | 0 | - | 0 | 0 | 0 | 1 |
| S175-192 | 175 | 175-192 | FLMDLVGKEGNFKNLREF | 0 | 1 | 0 | 0 | 1 | 0 | 0 | 1 | 0 | - | 0 | 0 | 0 | 3 |
| S175-192 | 175 | 175-192 | FLMDLEGEEGNFKNLREF | 0 | 0 | 1 | 0 | 0 | 0 | 0 | 0 | 0 | - | 0 | 0 | 0 | 1 |
| S175-192 | 175 | 175-192 | FLMDLEGKEGNFKNLREF | 0 | 0 | 0 | 1 | 0 | 0 | 0 | 0 | 0 | - | 0 | 0 | 0 | 1 |
| S175-192 | 175 | 175-192 | FLMDLEGKEVNFKNLREF | 0 | 0 | 0 | 0 | 0 | 0 | 1 | 0 | 0 | - | 0 | 0 | 0 | 1 |
| S175-192 | 175 | 175-192 | FLMDLEGNEGNFKNLREF | 0 | 0 | 0 | 0 | 0 | 1 | 0 | 0 | 0 | - | 0 | 0 | 0 | 1 |
| S178-195 | 178 | 178-195 | DLEGKEGNFKNLREFVFK | 1 | 0 | 0 | 0 | 0 | 0 | 0 | 0 | 0 | - | 0 | 0 | 0 | 1 |
| S182-199 | 182 | 182-199 | KEGNFKNLREFVFKNIDG | 0 | 0 | 0 | 0 | 0 | 0 | 0 | 0 | 0 | - | 0 | 1 | 0 | 1 |
| S183-200 | 183 | 183-200 | EGNFKNLREFVFKNIDGY | 0 | 1 | 1 | 1 | 1 | 1 | 0 | 1 | 0 | - | 0 | 0 | 0 | 6 |
| S183-200 | 183 | 183-200 | EVNFKNLREFVFKNIDGY | 0 | 0 | 0 | 0 | 0 | 0 | 1 | 0 | 0 | - | 0 | 0 | 0 | 1 |
| S186-203 | 186 | 186-203 | FKNLREFVFKNIDGYFKI | 0 | 0 | 0 | 0 | 0 | 0 | 0 | 0 | 0 | - | 1 | 0 | 0 | 1 |
| S188-205 | 188 | 188-205 | NLREFVFKNIDGYFKIYS | 1 | 0 | 0 | 0 | 0 | 0 | 0 | 0 | 1 | - | 0 | 0 | 0 | 2 |
| S193-210 | 193 | 193-210 | VFKNIDGYFKIYSKHTPI | 0 | 0 | 0 | 0 | 0 | 0 | 0 | 0 | 0 | - | 0 | 1 | 0 | 1 |
| S194-211 | 194 | 194-211 | FKNIDGYFKIYSKHTPIN | 0 | 0 | 0 | 0 | 0 | 0 | 0 | 0 | 1 | - | 0 | 0 | 0 | 1 |
| S195-212 | 195 | 195-212 | KNIDGYFKIYSKHTPINL | 1 | 0 | 0 | 0 | 0 | 0 | 0 | 0 | 0 | - | 0 | 0 | 0 | 1 |
| S196-213 | 196 | 196-213 | NIDGYFKIYSKHTPINLE | 0 | 0 | 1 | 1 | 0 | 1 | 1 | 0 | 0 | - | 1 | 0 | 0 | 5 |
| S199-216 | 199 | 199-216 | GYFKIYSKHTPINLERDL | 0 | 1 | 0 | 0 | 1 | 0 | 0 | 1 | 0 | - | 0 | 1 | 0 | 4 |
| S203-220 | 203 | 203-220 | IYSKHTPINLERDLPQGF | 1 | 0 | 0 | 0 | 0 | 0 | 0 | 0 | 1 | - | 0 | 0 | 0 | 2 |
| S208-225 | 208 | 208-225 | TPINLERDLPQGFSALEP | 0 | 1 | 1 | 1 | 1 | 1 | 1 | 1 | 0 | - | 1 | 1 | 0 | 9 |
| S209-226 | 209 | 209-226 | PINLERDLPQGFSALEPL | 1 | 0 | 0 | 0 | 0 | 0 | 0 | 0 | 0 | - | 0 | 0 | 0 | 1 |
| S213-230 | 213 | 213-230 | ERDLPQGFSALEPLVDLP | 0 | 0 | 0 | 0 | 0 | 0 | 0 | 0 | 1 | - | 0 | 0 | 0 | 1 |
| S218-235 | 218 | 218-235 | QGFSALEPLVDLPIGINI | 0 | 1 | 1 | 1 | 1 | 1 | 1 | 1 | 0 | - | 1 | 1 | 0 | 9 |
| S220-237 | 220 | 220-237 | FSALEPLVDLPIGINITR | 1 | 0 | 0 | 0 | 0 | 0 | 0 | 0 | 1 | - | 0 | 0 | 0 | 2 |
| S225-242 | 225 | 225-242 | PLVDLPIGINITRFQTLL | 0 | 1 | 1 | 1 | 1 | 1 | 1 | 1 | 0 | - | 1 | 1 | 0 | 9 |
| S229-246 | 229 | 229-246 | LPIGINITRFQTLLALHR | 0 | 0 | 0 | 0 | 0 | 0 | 0 | 0 | 1 | - | 0 | 0 | 0 | 1 |
| S230-249 | 230 | 230-249 | PIGINITRFQTLLHRSYL | 1 | 0 | 0 | 0 | 0 | 0 | 0 | 0 | 0 | - | 0 | 0 | 0 | 1 |
| S236-253 | 236 | 236-253 | TRFQTLLALHRSYLTPGG | 0 | 0 | 1 | 1 | 0 | 1 | 1 | 0 | 0 | - | 1 | 1 | 0 | 6 |
| S236-253 | 236 | 236-253 | TRFQTLLALHRSYLTPVD | 0 | 1 | 0 | 0 | 1 | 0 | 0 | 1 | 0 | - | 0 | 0 | 1 | 4 |
| S237-256 | 237 | 237-256 | RFQTLLHRSYLTPGGSSS | 1 | 0 | 0 | 0 | 0 | 0 | 0 | 0 | 0 | - | 0 | 0 | 0 | 1 |
| S242-259 | 242 | 242-259 | LALHRSYLTPGGSSSGWT | 0 | 0 | 1 | 1 | 0 | 1 | 1 | 0 | 0 | - | 1 | 1 | 0 | 6 |
| S242-259 | 242 | 242-259 | LALHRSYLTPVDSSSGWT | 0 | 1 | 0 | 0 | 1 | 0 | 0 | 1 | 0 | - | 0 | 0 | 0 | 3 |
| S243-260 | 243 | 243-260 | ALHRSYLTPGGSSSGWTA | 0 | 0 | 0 | 0 | 0 | 0 | 0 | 0 | 1 | - | 0 | 0 | 0 | 1 |
| S248-265 | 248 | 248-265 | YLTPGGSSSGWTAGAAAY | 1 | 0 | 0 | 0 | 0 | 0 | 0 | 0 | 0 | - | 0 | 0 | 0 | 1 |
| S250-267 | 250 | 250-267 | TPGGSSSGWTAGAAAYYV | 0 | 0 | 0 | 0 | 0 | 0 | 0 | 0 | 1 | - | 0 | 0 | 0 | 1 |
| S251-268 | 251 | 251-268 | PGGSSSGWTAGAAAYYVG | 0 | 0 | 1 | 1 | 0 | 1 | 1 | 0 | 0 | - | 1 | 1 | 0 | 6 |
| S251-268 | 251 | 251-268 | PVDSSSGWTAGAAAYYVG | 0 | 1 | 0 | 0 | 1 | 0 | 0 | 1 | 0 | - | 0 | 0 | 0 | 3 |
| S255-272 | 255 | 255-272 | SSGWTAGAAAYYVGYLQP | 1 | 0 | 0 | 0 | 0 | 0 | 0 | 0 | 0 | - | 0 | 0 | 0 | 1 |
| S257-274 | 257 | 257-274 | GWTAGAAAYYVGYLQPRT | 0 | 1 | 1 | 1 | 1 | 1 | 1 | 1 | 0 | - | 1 | 1 | 0 | 9 |
| S262-279 | 262 | 262-279 | AAAYYVGYLQPRTFLLKY | 0 | 0 | 0 | 0 | 0 | 0 | 0 | 0 | 1 | - | 0 | 0 | 0 | 1 |
| S266-283 | 266 | 266-283 | YVGYLQPRTFLLKYNENG | 1 | 0 | 0 | 0 | 0 | 0 | 0 | 0 | 0 | - | 0 | 0 | 0 | 1 |
| S270-287 | 270 | 270-287 | LQPRTFLLKYNENGTITD | 0 | 0 | 1 | 1 | 0 | 1 | 1 | 0 | 0 | - | 1 | 1 | 0 | 6 |
| S272-289 | 272 | 272-289 | PRTFLLKYNENGTITDAV | 0 | 1 | 0 | 0 | 1 | 0 | 0 | 1 | 0 | - | 0 | 0 | 0 | 3 |
| S275-292 | 275 | 275-292 | FLLKYNENGTITDAVGCA | 0 | 0 | 0 | 0 | 0 | 0 | 0 | 0 | 1 | - | 0 | 0 | 0 | 1 |
| S276-293 | 276 | 276-293 | LLKYNENGTITDAVDCAL | 1 | 0 | 0 | 0 | 0 | 0 | 0 | 0 | 0 | - | 0 | 0 | 1 | 2 |
| S277-294 | 277 | 277-294 | LKYNENGTITDAVDCALD | 0 | 0 | 1 | 1 | 0 | 1 | 1 | 0 | 0 | - | 1 | 0 | 0 | 5 |
| S281-298 | 281 | 281-298 | ENGTITDAVDCALDPLSE | 0 | 1 | 0 | 0 | 1 | 0 | 0 | 1 | 0 | - | 0 | 0 | 0 | 3 |
| S282-299 | 282 | 282-299 | NGTITDAVGCALDPLSET | 0 | 0 | 0 | 0 | 0 | 0 | 0 | 0 | 1 | - | 0 | 0 | 0 | 1 |
| S292-309 | 292 | 292-309 | ALDPLSETKCTLKSFTVE | 1 | 0 | 0 | 0 | 0 | 0 | 0 | 0 | 0 | - | 0 | 0 | 0 | 1 |
| S293-310 | 293 | 293-310 | LDPLSETKCTLKSFTVEK | 0 | 1 | 1 | 1 | 1 | 1 | 1 | 1 | 0 | - | 1 | 0 | 0 | 8 |
| S307-324 | 307 | 307-324 | TVEKGIYQTSNFRVQPTE | 1 | 0 | 0 | 0 | 0 | 0 | 0 | 0 | 0 | - | 0 | 0 | 0 | 1 |
| S308-325 | 308 | 308-325 | VEKGIYQTSNFRVQPTES | 0 | 1 | 0 | 1 | 1 | 1 | 1 | 1 | 0 | - | 1 | 0 | 0 | 7 |
| S308-325 | 308 | 308-325 | VEKGIYQTSNFRVQPIES | 0 | 0 | 1 | 0 | 0 | 0 | 0 | 0 | 0 | - | 0 | 0 | 0 | 1 |
| S331-348 | 311 | 331-348 | NITNLCPFHEVFNATTFA | 1 | 0 | 0 | 0 | 0 | 0 | 0 | 0 | 0 | - | 0 | 0 | 0 | 1 |
| S312-329 | 312 | 312-329 | IYQTSNFRVQPTESIVRF | 0 | 0 | 0 | 0 | 0 | 0 | 0 | 0 | 0 | - | 0 | 1 | 0 | 1 |
| S321-338 | 321 | 321-338 | QPTESIVRFPNITNLCPF | 0 | 0 | 0 | 0 | 0 | 0 | 0 | 0 | 0 | - | 0 | 1 | 0 | 1 |
| S324-341 | 324 | 324-341 | ESIVRFPNITNLCPFHEV | 1 | 0 | 0 | 0 | 0 | 0 | 0 | 0 | 0 | - | 0 | 0 | 0 | 1 |
| S325-342 | 325 | 325-342 | SIVRFPNITNLCPFHEVF | 0 | 1 | 1 | 1 | 1 | 1 | 1 | 1 | 0 | - | 1 | 0 | 0 | 8 |
| S332-349 | 332 | 332-349 | ITNLCPFHEVFNATTFAS | 0 | 1 | 1 | 1 | 1 | 1 | 1 | 1 | 0 | - | 1 | 0 | 0 | 8 |
| S337-354 | 337 | 337-354 | PFHEVFNATTFASVYAWN | 0 | 0 | 0 | 0 | 0 | 0 | 0 | 0 | 0 | - | 0 | 1 | 0 | 1 |
| S338-355 | 338 | 338-355 | FHEVFNATTFASVYAWNR | 1 | 0 | 0 | 0 | 0 | 0 | 0 | 0 | 0 | - | 0 | 0 | 0 | 1 |
| S339-356 | 339 | 339-356 | HEVFNATTFASVYAWNRK | 0 | 1 | 1 | 1 | 1 | 1 | 1 | 1 | 0 | - | 1 | 0 | 0 | 8 |
| S345-362 | 345 | 345-362 | TTFASVYAWNRKRISNCV | 1 | 0 | 0 | 0 | 0 | 0 | 0 | 0 | 0 | - | 0 | 0 | 0 | 1 |
| S346-363 | 346 | 346-363 | TFASVYAWNRKRISNCVA | 0 | 1 | 1 | 1 | 1 | 1 | 1 | 1 | 0 | - | 1 | 1 | 0 | 9 |
| S351-368 | 351 | 351-368 | YAWNRKRISNCVADYSVI | 1 | 0 | 0 | 0 | 0 | 0 | 0 | 0 | 0 | - | 0 | 0 | 0 | 1 |
| S352-369 | 352 | 352-369 | AWNRKRISNCVADYSVIY | 0 | 1 | 1 | 1 | 1 | 1 | 1 | 1 | 0 | - | 1 | 1 | 0 | 9 |
| S357-374 | 357 | 357-374 | RISNCVADYSVIYNFAPF | 1 | 0 | 0 | 0 | 0 | 0 | 0 | 0 | 0 | - | 0 | 0 | 1 | 2 |
| S358-375 | 358 | 358-375 | ISNCVADYSVIYNFAPFF | 0 | 1 | 1 | 1 | 1 | 1 | 1 | 1 | 0 | - | 1 | 1 | 0 | 9 |
| S368-385 | 368 | 368-385 | IYNFAPFFAFKCYGVSPI | 1 | 0 | 0 | 0 | 0 | 0 | 0 | 0 | 0 | - | 0 | 0 | 0 | 1 |
| S369-386 | 369 | 369-386 | YNFAPFFAFKCYGVSPTK | 0 | 1 | 1 | 1 | 1 | 1 | 1 | 1 | 0 | - | 1 | 1 | 0 | 9 |
| S380-397 | 380 | 380-397 | YGVSPIKLNDLCFTNVYA | 1 | 0 | 0 | 0 | 0 | 0 | 0 | 0 | 0 | - | 0 | 0 | 0 | 1 |
| S381-398 | 381 | 381-398 | GVSPTKLNDLCFTNVYAD | 0 | 1 | 1 | 1 | 1 | 1 | 1 | 1 | 0 | - | 1 | 1 | 1 | 10 |
| S386-403 | 386 | 386-403 | KLNDLCFTNVYADSFVIR | 1 | 0 | 0 | 0 | 0 | 0 | 0 | 0 | 0 | - | 0 | 0 | 0 | 1 |
| S387-404 | 387 | 387-404 | LNDLCFTNVYADSFVIRG | 0 | 1 | 1 | 1 | 1 | 1 | 1 | 1 | 0 | - | 1 | 1 | 0 | 9 |
| S392-409 | 392 | 392-409 | FTNVYADSFVIRGNEVSQ | 1 | 0 | 0 | 0 | 0 | 0 | 0 | 0 | 0 | - | 0 | 0 | 0 | 1 |
| S393-410 | 393 | 393-410 | TNVYADSFVIRGNEVSQI | 0 | 1 | 1 | 1 | 1 | 1 | 1 | 1 | 0 | - | 1 | 1 | 0 | 9 |
| S400-417 | 400 | 400-417 | FVIRGNEVSQIAPGQTGN | 1 | 0 | 0 | 0 | 0 | 0 | 0 | 0 | 0 | - | 0 | 0 | 0 | 1 |
| S401-418 | 401 | 401-418 | VIRGNEVSQIAPGQTGNI | 0 | 1 | 1 | 1 | 1 | 1 | 1 | 1 | 0 | - | 1 | 1 | 0 | 9 |
| S410-427 | 410 | 410-427 | IAPGQTGNIADYNYKLPD | 1 | 0 | 0 | 0 | 0 | 0 | 0 | 0 | 0 | - | 0 | 0 | 0 | 1 |
| S411-428 | 411 | 411-428 | APGQTGNIADYNYKLPDD | 0 | 1 | 1 | 1 | 1 | 1 | 1 | 1 | 0 | - | 1 | 1 | 0 | 9 |
| S416-433 | 416 | 416-433 | GNIADYNYKLPDDFTGCV | 1 | 0 | 0 | 0 | 0 | 0 | 0 | 0 | 0 | - | 0 | 0 | 0 | 1 |
| S417-434 | 417 | 417-434 | NIADYNYKLPDDFTGCVI | 0 | 1 | 1 | 1 | 1 | 1 | 1 | 1 | 0 | - | 1 | 1 | 0 | 9 |
| S422-439 | 422 | 422-439 | NYKLPDDFTGCVIAWNSN | 1 | 0 | 0 | 0 | 0 | 0 | 0 | 0 | 0 | - | 0 | 0 | 0 | 1 |
| S423-440 | 423 | 423-440 | YKLPDDFTGCVIAWNSNK | 0 | 1 | 1 | 1 | 1 | 1 | 1 | 1 | 0 | - | 1 | 1 | 0 | 9 |
| S430-447 | 430 | 430-447 | TGCVIAWNSNKLDSKPSG | 1 | 0 | 0 | 0 | 0 | 0 | 0 | 0 | 0 | - | 0 | 0 | 1 | 2 |
| S431-448 | 431 | 431-448 | GCVIAWNSNKLDSKPSGN | 0 | 1 | 1 | 1 | 1 | 1 | 1 | 1 | 0 | - | 1 | 1 | 0 | 9 |
| S437-454 | 437 | 437-454 | NSNKLDSKPSGNYNYLYR | 1 | 0 | 0 | 0 | 0 | 0 | 0 | 0 | 0 | - | 0 | 0 | 0 | 1 |
| S438-455 | 438 | 438-455 | SNKLDSKPSGNYNYLYRL | 0 | 1 | 1 | 1 | 1 | 1 | 1 | 1 | 0 | - | 1 | 1 | 0 | 9 |
| S453-470 | 453 | 453--470 | YRLFRKSKLKPFERDIST | 1 | 0 | 0 | 0 | 0 | 0 | 0 | 0 | 0 | - | 0 | 0 | 0 | 1 |
| S454-471 | 454 | 454-471 | RLFRKSKLKPFERDISTE | 0 | 1 | 1 | 1 | 1 | 1 | 1 | 1 | 0 | - | 1 | 0 | 0 | 8 |
| S463-480 | 463 | 463-480 | PFERDISTEIYQAGNQPC | 1 | 0 | 0 | 0 | 0 | 0 | 0 | 0 | 0 | - | 0 | 0 | 0 | 1 |
| S464-481 | 464 | 464-481 | FERDISTEIYQAGNRPCN | 0 | 1 | 0 | 0 | 1 | 1 | 0 | 1 | 0 | - | 0 | 0 | 0 | 4 |
| S464-481 | 464 | 464-481 | FERDISTEIYQAGNKPCN | 0 | 0 | 1 | 0 | 0 | 0 | 1 | 0 | 0 | - | 1 | 0 | 0 | 3 |
| S464-481 | 464 | 464-481 | FERDISTEIYQAGNNPCN | 0 | 0 | 0 | 1 | 0 | 0 | 0 | 0 | 0 | - | 0 | 0 | 0 | 1 |
| S476-493 | 476 | 476-493 | GNQPCNGVAGPNCYSPLQ | 1 | 0 | 0 | 0 | 0 | 0 | 0 | 0 | 0 | - | 0 | 0 | 0 | 1 |
| S477-494 | 477 | 477-494 | NRPCNGVAGPNCYSPLQS | 0 | 1 | 0 | 0 | 1 | 1 | 0 | 1 | 0 | - | 0 | 0 | 0 | 4 |
| S477-494 | 477 | 477-494 | NKPCNGVAGPNCYSPLQS | 0 | 0 | 1 | 0 | 0 | 0 | 1 | 0 | 0 | - | 1 | 0 | 0 | 3 |
| S477-494 | 477 | 477-494 | NNPCNGVAGPNCYSPLQS | 0 | 0 | 0 | 1 | 0 | 0 | 0 | 0 | 0 | - | 0 | 0 | 0 | 1 |
| S482-499 | 482 | 482-499 | GVAGPNCYSPLQSYGFRP | 1 | 0 | 0 | 0 | 0 | 0 | 0 | 0 | 0 | - | 0 | 0 | 0 | 1 |
| S483-500 | 483 | 483-500 | VAGPNCYSPLQSYGFRPT | 0 | 1 | 1 | 1 | 1 | 1 | 1 | 1 | 0 | - | 0 | 0 | 0 | 7 |
| S490-507 | 490 | 490-507 | SPLQSYGFRPTYGVGYQP | 1 | 0 | 0 | 0 | 0 | 0 | 0 | 0 | 0 | - | 0 | 0 | 0 | 1 |
| S491-508 | 491 | 491-508 | PLQSYGFRPTYGVGYQPY | 0 | 1 | 1 | 1 | 1 | 1 | 1 | 1 | 0 | - | 0 | 0 | 0 | 7 |
| S505-522 | 505 | 505-522 | YQPYRVVVLSFELLHASA | 1 | 0 | 0 | 0 | 0 | 0 | 0 | 0 | 0 | - | 0 | 0 | 0 | 1 |
| S506-523 | 506 | 506-523 | QPYRVVVLSFELLHASAT | 0 | 0 | 1 | 1 | 0 | 1 | 1 | 0 | 0 | - | 1 | 0 | 0 | 5 |
| S506-523 | 506 | 506-523 | QPYRVVVLSFELLHATAT | 0 | 1 | 0 | 0 | 1 | 0 | 0 | 0 | 0 | - | 0 | 0 | 0 | 2 |
| S506-523 | 506 | 506-523 | QPYRVVVLSFELLHAPAT | 0 | 0 | 0 | 0 | 0 | 0 | 0 | 1 | 0 | - | 0 | 0 | 0 | 1 |
| S514-531 | 514 | 514-531 | SFELLHASATVCGPKKST | 0 | 0 | 0 | 0 | 0 | 0 | 0 | 0 | 0 | - | 0 | 1 | 0 | 1 |
| S515-532 | 515 | 515-532 | FELLHASATVCGPKKSTN | 1 | 0 | 0 | 0 | 0 | 0 | 0 | 0 | 0 | - | 0 | 0 | 0 | 1 |
| S516-533 | 516 | 516-533 | ELLHASATVCGPKKSTNL | 0 | 0 | 1 | 1 | 0 | 1 | 1 | 0 | 0 | - | 1 | 0 | 0 | 5 |
| S516-533 | 516 | 516-533 | ELLHATATVCGPKKSTNL | 0 | 1 | 0 | 0 | 1 | 0 | 0 | 0 | 0 | - | 0 | 0 | 0 | 2 |
| S516-533 | 516 | 516-533 | ELLHAPATVCGPKKSTNL | 0 | 0 | 0 | 0 | 0 | 0 | 0 | 1 | 0 | - | 0 | 0 | 0 | 1 |
| S525-542 | 525 | 525-542 | CGPKKSTNLVKNKCVNFN | 1 | 0 | 0 | 0 | 0 | 0 | 0 | 0 | 0 | - | 0 | 0 | 0 | 1 |
| S526-543 | 526 | 526-543 | GPKKSTNLVKNKCVNFNF | 0 | 1 | 1 | 1 | 1 | 1 | 1 | 1 | 0 | - | 1 | 0 | 0 | 8 |
| S533-550 | 533 | 533-550 | LVKNKCVNFNFNGLTGTG | 1 | 0 | 0 | 0 | 0 | 0 | 0 | 0 | 0 | - | 0 | 0 | 0 | 1 |
| S534-551 | 534 | 534-551 | VKNKCVNFNFNGLTGTGV | 0 | 1 | 1 | 1 | 1 | 1 | 1 | 1 | 0 | - | 1 | 0 | 0 | 8 |
| S543-560 | 543 | 543-560 | FNGLTGTGVLTESNKKFL | 1 | 0 | 0 | 0 | 0 | 0 | 0 | 0 | 0 | - | 0 | 0 | 0 | 1 |
| S544-561 | 544 | 544-561 | NGLTGTGVLTESNKKFLP | 0 | 1 | 1 | 1 | 1 | 1 | 1 | 1 | 0 | - | 1 | 0 | 0 | 8 |
| S550-567 | 550 | 550-567 | GVLTESNKKFLPFQQFGR | 1 | 0 | 0 | 0 | 0 | 0 | 0 | 0 | 0 | - | 0 | 0 | 0 | 1 |
| S551-568 | 551 | 551-568 | VLTESNKKFLPFQQFGRD | 0 | 1 | 1 | 1 | 1 | 1 | 1 | 1 | 0 | - | 1 | 0 | 0 | 8 |
| S552-569 | 552 | 552-569 | LTESNKKFLPFQQFGRDI | 0 | 0 | 0 | 0 | 0 | 0 | 0 | 0 | 1 | - | 0 | 0 | 0 | 1 |
| S556-573 | 556 | 556-573 | NKKFLPFQQFGRDIADTT | 1 | 0 | 0 | 0 | 0 | 0 | 0 | 0 | 0 | - | 0 | 0 | 0 | 1 |
| S557-574 | 557 | 557-574 | KKFLPFQQFGRDIADTTD | 0 | 1 | 1 | 1 | 1 | 1 | 1 | 1 | 0 | - | 0 | 0 | 0 | 7 |
| S559-576 | 559 | 559-576 | FLPFQQFGRDIADTTDAV | 0 | 0 | 0 | 0 | 0 | 0 | 0 | 0 | 0 | - | 1 | 1 | 0 | 2 |
| S565-582 | 565 | 565-582 | FGRDIADTTDAVRDPQTL | 1 | 0 | 0 | 0 | 0 | 0 | 0 | 0 | 0 | - | 0 | 0 | 0 | 1 |
| S566-583 | 566 | 566-583 | GRDIADTTDAVRDPQTLE | 0 | 0 | 1 | 1 | 0 | 1 | 1 | 0 | 0 | - | 0 | 0 | 0 | 4 |
| S567-584 | 567 | 567-584 | RDIADTTDAVRDPQTLEI | 0 | 1 | 0 | 0 | 1 | 0 | 0 | 1 | 0 | - | 1 | 1 | 0 | 5 |
| S571-588 | 571 | 571-588 | DTTDAVRDPQTLEILDIT | 0 | 0 | 0 | 0 | 0 | 0 | 0 | 0 | 1 | - | 0 | 0 | 0 | 1 |
| S575-592 | 575 | 575-592 | AVRDPQTLEILDITPCSF | 1 | 0 | 0 | 0 | 0 | 0 | 0 | 0 | 0 | - | 0 | 0 | 0 | 1 |
| S576-593 | 576 | 576-593 | VRDPQTLEILDITPCSFG | 0 | 1 | 1 | 1 | 1 | 1 | 1 | 1 | 0 | - | 1 | 1 | 0 | 9 |
| S585-602 | 585 | 585-602 | LDITPCSFGGVSVITPGT | 1 | 0 | 0 | 0 | 0 | 0 | 0 | 0 | 1 | - | 0 | 0 | 0 | 2 |
| S586-603 | 586 | 586-603 | DITPCSFGGVSVITPGTN | 0 | 1 | 1 | 1 | 1 | 1 | 1 | 1 | 0 | - | 1 | 1 | 0 | 9 |
| S593-610 | 593 | 593-610 | GGVSVITPGTNTSNQVAV | 0 | 0 | 0 | 0 | 0 | 0 | 0 | 0 | 1 | - | 0 | 0 | 0 | 1 |
| S596-613 | 596 | 596-613 | SVITPGTNTSNQVAVLYQ | 1 | 0 | 0 | 0 | 0 | 0 | 0 | 0 | 0 | - | 0 | 0 | 0 | 1 |
| S597-614 | 597 | 597-614 | VITPGTNTSNQVAVLYQG | 0 | 1 | 1 | 1 | 1 | 1 | 1 | 1 | 0 | - | 1 | 1 | 0 | 9 |
| S599-616 | 599 | 599-616 | TPGTNTSNQVAVLYQGVN | 0 | 0 | 0 | 0 | 0 | 0 | 0 | 0 | 1 | - | 0 | 0 | 0 | 1 |
| S608-625 | 608 | 608-625 | VAVLYQGVNCTEVPVAIH | 1 | 0 | 0 | 0 | 0 | 0 | 0 | 0 | 0 | - | 0 | 0 | 0 | 1 |
| S609-626 | 609 | 609-626 | AVLYQGVNCTEVPVAIHA | 0 | 1 | 1 | 1 | 1 | 1 | 1 | 1 | 0 | - | 1 | 1 | 0 | 9 |
| S610-627 | 610 | 610-627 | VLYQGVNCTEVPVAIHAD | 0 | 0 | 0 | 0 | 0 | 0 | 0 | 0 | 1 | - | 0 | 0 | 0 | 1 |
| S614-631 | 614 | 614-631 | GVNCTEVPVAIHADQLTP | 1 | 0 | 0 | 0 | 0 | 0 | 0 | 0 | 0 | - | 0 | 0 | 0 | 1 |
| S615-632 | 615 | 615-632 | VNCTEVPVAIHADQLTPT | 0 | 1 | 1 | 1 | 1 | 1 | 1 | 1 | 0 | - | 1 | 1 | 0 | 9 |
| S620-637 | 620 | 620-637 | VPVAIHADQLTPTWRVYS | 0 | 0 | 0 | 0 | 0 | 0 | 0 | 0 | 1 | - | 0 | 0 | 0 | 1 |
| S624-641 | 624 | 624-641 | IHADQLTPTWRVYSTGSN | 1 | 0 | 0 | 0 | 0 | 0 | 0 | 0 | 0 | - | 0 | 0 | 0 | 1 |
| S625-642 | 625 | 625-642 | HADQLTPTWRVYSTGSNV | 0 | 1 | 1 | 1 | 1 | 1 | 1 | 1 | 0 | - | 1 | 1 | 0 | 9 |
| S626-643 | 626 | 626-643 | ADQLTPTWRVYSTGSNVF | 0 | 0 | 0 | 0 | 0 | 0 | 0 | 0 | 1 | - | 0 | 0 | 0 | 1 |
| S638-655 | 638 | 638-655 | TGSNVFQTRAGCLIGAEY | 0 | 0 | 0 | 0 | 0 | 0 | 0 | 0 | 1 | - | 0 | 0 | 0 | 1 |
| S642-659 | 642 | 642-659 | VFQTRAGCLIGAEYVNNS | 1 | 0 | 0 | 0 | 0 | 0 | 0 | 0 | 0 | - | 0 | 0 | 0 | 1 |
| S643-660 | 643 | 643-660 | FQTRAGCLIGAEYVNNSY | 0 | 1 | 1 | 1 | 1 | 1 | 1 | 1 | 0 | - | 1 | 1 | 0 | 9 |
| S649-666 | 649 | 649-666 | CLIGAEYVNNSYECDIPI | 1 | 0 | 0 | 0 | 0 | 0 | 0 | 0 | 1 | - | 0 | 0 | 0 | 2 |
| S650-667 | 650 | 650-667 | LIGAEYVNNSYECDIPIG | 0 | 1 | 1 | 1 | 1 | 1 | 1 | 1 | 0 | - | 1 | 1 | 0 | 9 |
| S655-672 | 655 | 655-672 | YVNNSYECDIPIGAGICA | 0 | 0 | 0 | 0 | 0 | 0 | 0 | 0 | 1 | - | 0 | 0 | 0 | 1 |
| S659-676 | 659 | 659-676 | SYECDIPIGAGICASYQT | 1 | 0 | 0 | 0 | 0 | 0 | 0 | 0 | 0 | - | 0 | 0 | 0 | 1 |
| S660-677 | 660 | 660-677 | YECDIPIGAGICASYQTQ | 0 | 1 | 1 | 1 | 1 | 1 | 1 | 1 | 0 | - | 1 | 1 | 0 | 9 |
| S662-679 | 662 | 662-679 | CDIPIGAGICASYQTQTK | 0 | 0 | 0 | 0 | 0 | 0 | 0 | 0 | 1 | - | 0 | 0 | 0 | 1 |
| S666-683 | 666 | 666-683 | IGAGICASYQTQTKSHRR | 1 | 0 | 0 | 0 | 0 | 0 | 0 | 0 | 0 | - | 0 | 0 | 0 | 1 |
| S667-684 | 667 | 667-684 | GAGICASYQTQTKSHRRA | 0 | 1 | 1 | 1 | 1 | 1 | 1 | 1 | 0 | - | 1 | 1 | 1 | 10 |
| S668-685 | 668 | 668-685 | AGICASYQTQTKSHRRAR | 0 | 0 | 0 | 0 | 0 | 0 | 0 | 0 | 1 | - | 0 | 0 | 0 | 1 |
| S672-689 | 672 | 672-689 | ASYQTQTKSHRRARSVAS | 1 | 0 | 0 | 0 | 0 | 0 | 0 | 0 | 0 | - | 0 | 0 | 0 | 1 |
| S673-690 | 673 | 673-690 | SYQTQTKSHRRARSVASQ | 0 | 1 | 1 | 1 | 1 | 1 | 1 | 1 | 0 | - | 1 | 1 | 0 | 9 |
| S681-698 | 681 | 681-698 | HRRARSVASQSIIAYTMS | 1 | 0 | 0 | 0 | 0 | 0 | 0 | 0 | 0 | - | 0 | 0 | 0 | 1 |
| S682-699 | 682 | 682-699 | RRARSVASQSIIAYTMSL | 0 | 1 | 1 | 1 | 1 | 1 | 1 | 1 | 0 | - | 1 | 1 | 0 | 9 |
| S688-705 | 688 | 688-705 | ASQSIIAYTMSLGAENSV | 1 | 0 | 0 | 0 | 0 | 0 | 0 | 0 | 0 | - | 0 | 0 | 0 | 1 |
| S689-706 | 689 | 689-706 | SQSIIAYTMSLGAENSVA | 0 | 1 | 1 | 1 | 1 | 1 | 1 | 1 | 0 | - | 1 | 1 | 0 | 9 |
| S702-719 | 702 | 702-719 | ENSVAYSNNSIAIPTNFT | 1 | 0 | 0 | 0 | 0 | 0 | 0 | 0 | 0 | - | 0 | 0 | 0 | 1 |
| S703-720 | 703 | 703-720 | NSVAYSNNSIAIPTNFTI | 0 | 1 | 1 | 1 | 1 | 1 | 1 | 1 | 0 | - | 1 | 1 | 0 | 9 |
| S710-727 | 710 | 710-727 | NSIAIPTNFTISVTTEIL | 1 | 0 | 0 | 0 | 0 | 0 | 0 | 0 | 0 | - | 0 | 0 | 0 | 1 |
| S711-728 | 711 | 711-728 | SIAIPTNFTISVTTEILP | 0 | 1 | 1 | 1 | 1 | 1 | 1 | 1 | 0 | - | 1 | 1 | 0 | 9 |
| S722-739 | 722 | 722-739 | VTTEILPVSMTKTSVDCT | 1 | 0 | 0 | 0 | 0 | 0 | 0 | 0 | 0 | - | 0 | 0 | 0 | 1 |
| S723-740 | 723 | 723-740 | TTEILPVSMTKTSVDCTM | 0 | 1 | 1 | 1 | 1 | 1 | 1 | 1 | 0 | - | 1 | 1 | 0 | 9 |
| S734-751 | 734 | 734-751 | TSVDCTMYICGDSTECSN | 1 | 0 | 0 | 0 | 0 | 0 | 0 | 0 | 0 | - | 0 | 0 | 0 | 1 |
| S735-752 | 735 | 735-752 | SVDCTMYICGDSTECSNL | 0 | 1 | 1 | 1 | 1 | 1 | 1 | 1 | 0 | - | 1 | 1 | 0 | 9 |
| S740-757 | 740 | 740-757 | MYICGDSTECSNLLLQYG | 0 | 0 | 0 | 0 | 0 | 0 | 0 | 0 | 1 | - | 0 | 0 | 0 | 1 |
| S741-758 | 741 | 741-758 | YICGDSTECSNLLLQYGS | 1 | 0 | 0 | 0 | 0 | 0 | 0 | 0 | 0 | - | 0 | 0 | 0 | 1 |
| S742-759 | 742 | 742-759 | ICGDSTECSNLLLQYGSF | 0 | 1 | 1 | 1 | 1 | 1 | 1 | 1 | 0 | - | 1 | 1 | 0 | 9 |
| S746-763 | 746 | 746-763 | STECSNLLLQYGSFCTQL | 0 | 0 | 0 | 0 | 0 | 0 | 0 | 0 | 1 | - | 0 | 0 | 0 | 1 |
| S749-766 | 749 | 749-766 | CSNLLLQYGSFCTQLKRA | 1 | 0 | 0 | 0 | 0 | 0 | 0 | 0 | 0 | - | 0 | 0 | 0 | 1 |
| S750-767 | 750 | 750-767 | SNLLLQYGSFCTQLKRAL | 0 | 1 | 1 | 1 | 1 | 1 | 1 | 1 | 0 | - | 1 | 1 | 0 | 9 |
| S752-769 | 752 | 752-769 | LLLQYGSFCTQLNRALTG | 0 | 0 | 0 | 0 | 0 | 0 | 0 | 0 | 1 | - | 0 | 0 | 0 | 1 |
| S755-772 | 755 | 755-772 | QYGSFCTQLKRALTGIAV | 1 | 0 | 0 | 0 | 0 | 0 | 0 | 0 | 0 | - | 0 | 0 | 0 | 1 |
| S756-773 | 756 | 756-773 | YGSFCTQLKRALTGIAVE | 0 | 1 | 1 | 1 | 1 | 1 | 1 | 1 | 0 | - | 1 | 1 | 0 | 9 |
| S758-775 | 758 | 758-775 | SFCTQLNRALTGIAVEQD | 0 | 0 | 0 | 0 | 0 | 0 | 0 | 0 | 1 | - | 0 | 0 | 0 | 1 |
| S761-778 | 761 | 761-778 | TQLKRALTGIAVEQDKNT | 1 | 0 | 0 | 0 | 0 | 0 | 0 | 0 | 0 | - | 0 | 0 | 0 | 1 |
| S762-779 | 762 | 762-779 | QLKRALTGIAVEQDKNTQ | 0 | 1 | 1 | 1 | 1 | 1 | 1 | 1 | 0 | - | 1 | 0 | 0 | 8 |
| S764-781 | 764 | 764-781 | NRALTGIAVEQDKNTQEV | 0 | 0 | 0 | 0 | 0 | 0 | 0 | 0 | 1 | - | 0 | 0 | 0 | 1 |
| S770-787 | 770 | 770-787 | IAVEQDKNTQEVFAQVKQ | 1 | 0 | 0 | 0 | 0 | 0 | 0 | 0 | 0 | - | 0 | 0 | 0 | 1 |
| S771-788 | 771 | 771-788 | AVEQDKNTQEVFAQVKQI | 0 | 1 | 1 | 1 | 1 | 1 | 1 | 1 | 0 | - | 1 | 0 | 0 | 8 |
| S778-795 | 778 | 778-795 | TQEVFAQVKQIYKTPPIK | 1 | 0 | 0 | 0 | 0 | 0 | 0 | 0 | 0 | - | 0 | 0 | 0 | 1 |
| S779-796 | 779 | 779-796 | QEVFAQVKQIYKTPPIKY | 0 | 1 | 1 | 1 | 1 | 1 | 1 | 1 | 0 | - | 1 | 0 | 0 | 8 |
| S788-805 | 788 | 788-805 | IYKTPPIKYFGGFNFSQI | 1 | 0 | 0 | 0 | 0 | 0 | 0 | 0 | 0 | - | 0 | 0 | 0 | 1 |
| S789-806 | 789 | 789-806 | YKTPPIKYFGGFNFSQIL | 0 | 1 | 1 | 1 | 1 | 1 | 1 | 1 | 0 | - | 1 | 0 | 0 | 8 |
| S795-812 | 795 | 795-812 | KYFGGFNFSQILPDPSKP | 1 | 0 | 0 | 0 | 0 | 0 | 0 | 0 | 0 | - | 0 | 0 | 0 | 1 |
| S796-813 | 796 | 796-813 | YFGGFNFSQILPDPSKPS | 0 | 1 | 1 | 1 | 1 | 1 | 1 | 1 | 0 | - | 1 | 0 | 0 | 8 |
| S806-823 | 804 | 806-823 | LPDPSKPSKRSFIEDLLF | 1 | 0 | 0 | 0 | 0 | 0 | 0 | 0 | 0 | - | 0 | 0 | 0 | 1 |
| S807-824 | 807 | 807-824 | PDPSKPSKRSFIEDLLFN | 0 | 1 | 1 | 1 | 1 | 1 | 1 | 1 | 0 | - | 1 | 0 | 0 | 8 |
| S810-827 | 810 | 810-827 | SKPSKRSFIEDLLFNKVT | 0 | 0 | 0 | 0 | 0 | 0 | 0 | 0 | 1 | - | 0 | 0 | 0 | 1 |
| S812-829 | 812 | 812-829 | PSKRSFIEDLLFNKVTLA | 1 | 0 | 0 | 0 | 0 | 0 | 0 | 0 | 0 | - | 0 | 0 | 0 | 1 |
| S813-830 | 813 | 813-830 | SKRSFIEDLLFNKVTLAD | 0 | 1 | 1 | 1 | 1 | 1 | 1 | 1 | 0 | - | 1 | 0 | 0 | 8 |
| S816-833 | 816 | 816-833 | SFIEDLLFNKVTLADAGF | 0 | 0 | 0 | 0 | 0 | 0 | 0 | 0 | 1 | - | 0 | 0 | 0 | 1 |
| S818-835 | 818 | 818-835 | IEDLLFNKVTLADAGFIK | 1 | 0 | 0 | 0 | 0 | 0 | 0 | 0 | 0 | - | 0 | 0 | 0 | 1 |
| S819-836 | 819 | 819-836 | EDLLFNKVTLADAGFIKQ | 0 | 1 | 1 | 1 | 1 | 1 | 1 | 1 | 0 | - | 1 | 1 | 0 | 9 |
| S824-841 | 824 | 824-841 | NKVTLADAGFIKQYGDCL | 1 | 0 | 0 | 0 | 0 | 0 | 0 | 0 | 0 | - | 0 | 0 | 0 | 1 |
| S825-842 | 825 | 825-842 | KVTLADAGFIKQYGDCLG | 0 | 1 | 1 | 1 | 1 | 1 | 1 | 1 | 0 | - | 1 | 1 | 0 | 9 |
| S828-845 | 828 | 828-845 | LADAGFIKQYGDCLGDIA | 0 | 0 | 0 | 0 | 0 | 0 | 0 | 0 | 1 | - | 0 | 0 | 0 | 1 |
| S832-849 | 832 | 832-849 | GFIKQYGDCLGDIAARDL | 1 | 0 | 0 | 0 | 0 | 0 | 0 | 0 | 0 | - | 0 | 0 | 0 | 1 |
| S833-850 | 833 | 833-850 | FIKQYGDCLGDIAARDLI | 0 | 1 | 1 | 1 | 1 | 1 | 1 | 1 | 0 | - | 1 | 1 | 0 | 9 |
| S839-856 | 839 | 839-856 | DCLGDIAARDLICAQKFN | 0 | 0 | 0 | 0 | 0 | 0 | 0 | 0 | 1 | - | 0 | 0 | 0 | 1 |
| S845-862 | 845 | 845-862 | AARDLICAQKFNGLTVLP | 1 | 0 | 0 | 0 | 0 | 0 | 0 | 0 | 0 | - | 0 | 0 | 0 | 1 |
| S846-863 | 846 | 846-863 | ARDLICAQKFNGLTVLPP | 0 | 1 | 1 | 1 | 1 | 1 | 1 | 1 | 0 | - | 1 | 0 | 0 | 8 |
| S846-863 | 846 | 846-863 | ARDLICAQKVNGLTVLPP | 0 | 0 | 0 | 0 | 0 | 0 | 0 | 0 | 0 | - | 0 | 1 | 0 | 1 |
| S854-871 | 854 | 854-871 | KFNGLTVLPPLLTDEMIA | 0 | 0 | 0 | 0 | 0 | 0 | 0 | 0 | 1 | - | 0 | 0 | 0 | 1 |
| S858-875 | 858 | 858-875 | LTVLPPLLTDEMIAQYTS | 1 | 0 | 0 | 0 | 0 | 0 | 0 | 0 | 0 | - | 0 | 0 | 0 | 1 |
| S859-876 | 859 | 859-876 | TVLPPLLTDEMIAQYTSA | 0 | 1 | 1 | 1 | 1 | 1 | 1 | 1 | 0 | - | 1 | 1 | 0 | 9 |
| S860-877 | 860 | 860-877 | VLPPLLTDEMIAQYTSAL | 0 | 0 | 0 | 0 | 0 | 0 | 0 | 0 | 1 | - | 0 | 0 | 0 | 1 |
| S867-884 | 867 | 867-884 | DEMIAQYTSALLAGTITS | 1 | 0 | 0 | 0 | 0 | 0 | 0 | 0 | 0 | - | 0 | 0 | 0 | 1 |
| S868-885 | 868 | 868-885 | EMIAQYTSALLAGTITSG | 0 | 1 | 1 | 1 | 1 | 1 | 1 | 1 | 0 | - | 1 | 0 | 0 | 8 |
| S869-886 | 869 | 869-886 | MIAQYTSALLAGTITSGW | 0 | 0 | 0 | 0 | 0 | 0 | 0 | 0 | 0 | - | 0 | 1 | 0 | 1 |
| S871-888 | 871 | 871-888 | AQYTSALLAGTITSGWTF | 0 | 0 | 0 | 0 | 0 | 0 | 0 | 0 | 1 | - | 0 | 0 | 0 | 1 |
| S875-892 | 875 | 875-892 | SALLAGTITSGWTFGAGA | 1 | 0 | 0 | 0 | 0 | 0 | 0 | 0 | 0 | - | 0 | 0 | 0 | 1 |
| S876-893 | 876 | 876-893 | ALLAGTITSGWTFGAGAA | 0 | 1 | 1 | 1 | 1 | 1 | 1 | 1 | 0 | - | 1 | 0 | 0 | 8 |
| S877-894 | 877 | 877-894 | LLAGTITSGWTFGAGAAL | 0 | 0 | 0 | 0 | 0 | 0 | 0 | 0 | 1 | - | 0 | 0 | 0 | 1 |
| S892-909 | 892 | 892-909 | AALQIPFAMQMAYRFNGI | 1 | 0 | 0 | 0 | 0 | 0 | 0 | 0 | 0 | - | 0 | 0 | 0 | 1 |
| S893-910 | 893 | 893-910 | ALQIPFAMQMAYRFNGIG | 0 | 1 | 1 | 1 | 1 | 1 | 1 | 1 | 0 | - | 1 | 0 | 0 | 8 |
| S894-911 | 894 | 894-911 | LQIPFAMQMAYRFNGIGV | 0 | 0 | 0 | 0 | 0 | 0 | 0 | 0 | 1 | - | 0 | 0 | 0 | 1 |
| S898-915 | 898 | 898-915 | FAMQMAYRFNGIGVTQNV | 1 | 0 | 0 | 0 | 0 | 0 | 0 | 0 | 0 | - | 0 | 0 | 0 | 1 |
| S899-916 | 899 | 899-916 | AMQMAYRFNGIGVTQNVL | 0 | 1 | 1 | 1 | 1 | 1 | 1 | 1 | 0 | - | 1 | 0 | 0 | 8 |
| S906-923 | 906 | 906-923 | FNGIGVTQNVLYENQKLI | 1 | 0 | 0 | 0 | 0 | 0 | 0 | 0 | 0 | - | 0 | 0 | 1 | 2 |
| S907-924 | 907 | 907-924 | NGIGVTQNVLYENQKLIA | 0 | 1 | 1 | 1 | 1 | 1 | 1 | 1 | 0 | - | 1 | 0 | 0 | 8 |
| S909-926 | 909 | 909-926 | IGVTQNVLYENQKLIANQ | 0 | 0 | 0 | 0 | 0 | 0 | 0 | 0 | 1 | - | 0 | 0 | 0 | 1 |
| S914-931 | 914 | 914-931 | NVLYENQKLIANQFNSAI | 0 | 0 | 0 | 0 | 0 | 0 | 0 | 0 | 0 | - | 0 | 1 | 0 | 1 |
| S915-932 | 915 | 915-932 | VLYENQKLIANQFNSAIG | 0 | 0 | 0 | 0 | 0 | 0 | 0 | 0 | 1 | - | 0 | 0 | 0 | 1 |
| S918-935 | 918 | 918-935 | ENQKLIANQFNSAIGKIQ | 1 | 0 | 0 | 0 | 0 | 0 | 0 | 0 | 0 | - | 0 | 0 | 0 | 1 |
| S919-936 | 919 | 919-936 | NQKLIANQFNSAIGKIQD | 0 | 1 | 1 | 1 | 1 | 1 | 1 | 1 | 0 | - | 1 | 0 | 0 | 8 |
| S921-938 | 921 | 921-938 | KLIANQFNSAIGKIQDSL | 0 | 0 | 0 | 0 | 0 | 0 | 0 | 0 | 0 | - | 0 | 1 | 0 | 1 |
| S924-941 | 924 | 924-941 | ANQFNSAIGKIQDSLSST | 0 | 0 | 0 | 0 | 0 | 0 | 0 | 0 | 1 | - | 0 | 0 | 0 | 1 |
| S928-945 | 928 | 928-945 | NSAIGKIQDSLSSTASAL | 1 | 0 | 0 | 0 | 0 | 0 | 0 | 0 | 0 | - | 0 | 0 | 0 | 1 |
| S929-946 | 929 | 929-946 | SAIGKIQDSLSSTASALG | 0 | 1 | 1 | 1 | 1 | 1 | 1 | 1 | 0 | - | 1 | 1 | 0 | 9 |
| S935-952 | 935 | 935-952 | QDSLSSTASALGKLQDVV | 0 | 0 | 0 | 0 | 0 | 0 | 0 | 0 | 1 | - | 0 | 0 | 0 | 1 |
| S942-959 | 942 | 942-959 | ASALGKLQDVVNHNAQAL | 0 | 0 | 0 | 0 | 0 | 0 | 0 | 0 | 1 | - | 0 | 0 | 0 | 1 |
| S944-961 | 944 | 944-961 | ALGKLQDVVNHNAQALNT | 1 | 0 | 0 | 0 | 0 | 0 | 0 | 0 | 0 | - | 0 | 0 | 0 | 1 |
| S945-962 | 945 | 945-962 | LGKLQDVVNHNAQALNTL | 0 | 1 | 1 | 1 | 1 | 1 | 1 | 1 | 0 | - | 1 | 1 | 0 | 9 |
| S951-968 | 951 | 951-968 | VVNHNAQALNTLVKQLSS | 1 | 0 | 0 | 0 | 0 | 0 | 0 | 0 | 0 | - | 0 | 0 | 0 | 1 |
| S952-969 | 952 | 952-969 | VNHNAQALNTLVKQLSSK | 0 | 1 | 1 | 1 | 1 | 1 | 1 | 1 | 0 | - | 1 | 1 | 0 | 9 |
| S954-971 | 954 | 954-971 | HNAQALNTLVKQLSSKFG | 0 | 0 | 0 | 0 | 0 | 0 | 0 | 0 | 1 | - | 0 | 0 | 0 | 1 |
| S959-976 | 959 | 959-976 | LNTLVKQLSSKFGAISSV | 0 | 0 | 0 | 0 | 0 | 0 | 0 | 0 | 0 | - | 0 | 1 | 0 | 1 |
| S961-978 | 961 | 961-978 | TLVKQLSSKFGAISSVLN | 1 | 0 | 0 | 0 | 0 | 0 | 0 | 0 | 0 | - | 0 | 0 | 0 | 1 |
| S962-979 | 962 | 962-979 | LVKQLSSKFGAISSVLND | 0 | 1 | 1 | 1 | 1 | 1 | 1 | 1 | 1 | - | 1 | 0 | 0 | 9 |
| S966-983 | 966 | 966-983 | LSSKFGAISSVLNDILSR | 0 | 0 | 0 | 0 | 0 | 0 | 0 | 0 | 0 | - | 0 | 1 | 0 | 1 |
| S972-989 | 972 | 972-989 | AISSVLNDILSRLDKVEA | 1 | 0 | 0 | 0 | 0 | 0 | 0 | 0 | 0 | - | 0 | 0 | 0 | 1 |
| S973-990 | 973 | 973-990 | ISSVLNDILSRLDKVEAE | 0 | 1 | 1 | 1 | 1 | 1 | 1 | 1 | 0 | - | 1 | 1 | 0 | 9 |
| S979-996 | 979 | 979-996 | DILSRLDKVEAEVQIDRL | 0 | 0 | 0 | 0 | 0 | 0 | 0 | 0 | 1 | - | 0 | 0 | 0 | 1 |
| S983-1000 | 983 | 983-1000 | RLDKVEAEVQIDRLITGR | 1 | 0 | 0 | 0 | 0 | 0 | 0 | 0 | 0 | - | 0 | 0 | 1 | 2 |
| S984-1001 | 984 | 984-1001 | LDKVEAEVQIDRLITGRL | 0 | 1 | 1 | 1 | 1 | 1 | 1 | 1 | 0 | - | 1 | 1 | 0 | 9 |
| S998-1015 | 998 | 998-1015 | TGRLQSLQTYVTQQLIRA | 1 | 0 | 0 | 0 | 0 | 0 | 0 | 0 | 0 | - | 0 | 0 | 0 | 1 |
| S999-1016 | 999 | 999-1016 | GRLQSLQTYVTQQLIRAA | 0 | 1 | 1 | 1 | 1 | 1 | 1 | 1 | 0 | - | 1 | 1 | 1 | 10 |
| S1004-1021 | 1004 | 1004-1021 | LQTYVTQQLIRAAEIRAS | 1 | 0 | 0 | 0 | 0 | 0 | 0 | 0 | 0 | - | 0 | 0 | 0 | 1 |
| S1005-1022 | 1005 | 1005-1022 | QTYVTQQLIRAAEIRASA | 0 | 1 | 0 | 1 | 1 | 1 | 1 | 1 | 0 | - | 1 | 1 | 0 | 8 |
| S1005-1022 | 1005 | 1005-1022 | QTYVTQQLIRAAEIRVSA | 0 | 0 | 1 | 0 | 0 | 0 | 0 | 0 | 0 | - | 0 | 0 | 0 | 1 |
| S1015-1032 | 1015 | 1015-1032 | AAEIRASANLAATKMSEC | 1 | 0 | 0 | 0 | 0 | 0 | 0 | 0 | 0 | - | 0 | 0 | 0 | 1 |
| S1016-1033 | 1016 | 1016-1033 | AEIRASANLAATKMSECV | 0 | 1 | 0 | 1 | 1 | 1 | 1 | 1 | 0 | - | 1 | 1 | 0 | 8 |
| S1016-1033 | 1016 | 1016-1033 | AEIRVSANLAATKMSECV | 0 | 0 | 1 | 0 | 0 | 0 | 0 | 0 | 0 | - | 0 | 0 | 0 | 1 |
| S1018-1035 | 1018 | 1018-1035 | IRASANLAATKMSECVLG | 0 | 0 | 0 | 0 | 0 | 0 | 0 | 0 | 1 | - | 0 | 0 | 0 | 1 |
| S1022-1039 | 1022 | 1022-1039 | ANLAATKMSECVLGQSKR | 1 | 0 | 0 | 0 | 0 | 0 | 0 | 0 | 0 | - | 0 | 0 | 0 | 1 |
| S1023-1040 | 1023 | 1023-1040 | NLAATKMSECVLGQSKRV | 0 | 1 | 1 | 1 | 1 | 1 | 1 | 1 | 0 | - | 1 | 1 | 0 | 9 |
| S1030-1047 | 1030 | 1030-1047 | SECVLGQSKRVDFCGKGY | 0 | 0 | 0 | 0 | 0 | 0 | 0 | 0 | 1 | - | 0 | 0 | 0 | 1 |
| S1034-1051 | 1034 | 1034-1051 | LGQSKRVDFCGKGYHLMS | 1 | 0 | 0 | 0 | 0 | 0 | 0 | 0 | 0 | - | 0 | 0 | 1 | 2 |
| S1035-1052 | 1035 | 1035-1052 | GQSKRVDFCGKGYHLMSF | 0 | 1 | 1 | 1 | 1 | 1 | 1 | 1 | 0 | - | 1 | 1 | 0 | 9 |
| S1042-1059 | 1042 | 1042-1059 | FCGKGYHLMSFPQSAPHG | 1 | 0 | 0 | 0 | 0 | 0 | 0 | 0 | 0 | - | 0 | 0 | 0 | 1 |
| S1043-1060 | 1043 | 1043-1060 | CGKGYHLMSFPQSAPHGV | 0 | 1 | 1 | 1 | 1 | 1 | 1 | 1 | 0 | - | 1 | 1 | 0 | 9 |
| S1050-1067 | 1050 | 1050-1067 | MSFPQSAPHGVVFLHVTY | 0 | 0 | 0 | 0 | 0 | 0 | 0 | 0 | 1 | - | 0 | 0 | 0 | 1 |
| S1058-1075 | 1058 | 1058-1075 | HGVVFLHVTYVPAQEKNF | 0 | 0 | 0 | 0 | 0 | 0 | 0 | 0 | 1 | - | 0 | 0 | 0 | 1 |
| S1059-1076 | 1059 | 1059-1076 | GVVFLHVTYVPAQEKNFT | 1 | 0 | 0 | 0 | 0 | 0 | 0 | 0 | 0 | - | 0 | 0 | 0 | 1 |
| S1060-1077 | 1060 | 1060-1077 | VVFLHVTYVPAQEKNFTT | 0 | 1 | 1 | 1 | 1 | 1 | 1 | 1 | 0 | - | 1 | 1 | 0 | 9 |
| S1065-1082 | 1065 | 1065-1082 | VTYVPAQEKNFTTAPAIC | 0 | 0 | 0 | 0 | 0 | 0 | 0 | 0 | 1 | - | 0 | 0 | 0 | 1 |
| S1069-1086 | 1069 | 1069-1086 | PAQEKNFTTAPAICHDGK | 1 | 0 | 0 | 0 | 0 | 0 | 0 | 0 | 0 | - | 0 | 0 | 0 | 1 |
| S1070-1087 | 1070 | 1070-1087 | AQEKNFTTAPAICHDGKA | 0 | 1 | 1 | 1 | 1 | 1 | 1 | 1 | 0 | - | 1 | 1 | 0 | 9 |
| S1096-1113 | 1096 | 1096-1113 | VSNGTHWFVTQRNFYEPQ | 0 | 0 | 0 | 0 | 0 | 0 | 0 | 0 | 1 | - | 0 | 0 | 0 | 1 |
| S1100-1117 | 1100 | 1100-1117 | THWFVTQRNFYEPQIITT | 1 | 0 | 0 | 0 | 0 | 0 | 0 | 0 | 0 | - | 0 | 0 | 0 | 1 |
| S1101-1118 | 1101 | 1101-1118 | HWFVTQRNFYEPQIITTD | 0 | 1 | 1 | 1 | 1 | 1 | 1 | 1 | 0 | - | 1 | 1 | 0 | 9 |
| S1105-1122 | 1105 | 1105-1122 | TQRNFYEPQIITTDNTFV | 0 | 0 | 0 | 0 | 0 | 0 | 0 | 0 | 1 | - | 0 | 0 | 0 | 1 |
| S1109-1126 | 1109 | 1109-1126 | FYEPQIITTDNTFVSGNC | 1 | 0 | 0 | 0 | 0 | 0 | 0 | 0 | 0 | - | 0 | 0 | 0 | 1 |
| S1110-1127 | 1110 | 1110-1127 | YEPQIITTDNTFVSGNCD | 0 | 1 | 1 | 1 | 1 | 1 | 1 | 1 | 0 | - | 1 | 1 | 0 | 9 |
| S1114-1131 | 1114 | 1114-1131 | IITTDNTFVSGNCDVVIG | 0 | 0 | 0 | 0 | 0 | 0 | 0 | 0 | 1 | - | 0 | 0 | 0 | 1 |
| S1126-1143 | 1126 | 1126-1143 | CDVVIGIVNNTVYDPLQP | 1 | 0 | 0 | 0 | 0 | 0 | 0 | 0 | 0 | - | 0 | 0 | 0 | 1 |
| S1127-1144 | 1127 | 1127-1144 | DVVIGIVNNTVYDPLQPE | 0 | 1 | 1 | 1 | 1 | 1 | 1 | 1 | 1 | - | 1 | 1 | 0 | 10 |
| S1135-1152 | 1135 | 1135-1152 | NTVYDPLQPELDSFKEEL | 1 | 0 | 0 | 0 | 0 | 0 | 0 | 0 | 1 | - | 0 | 0 | 0 | 2 |
| S1136-1153 | 1136 | 1136-1153 | TVYDPLQPELDSFKEELD | 0 | 1 | 1 | 1 | 1 | 1 | 1 | 1 | 0 | - | 1 | 1 | 0 | 9 |
| S1143-1160 | 1143 | 1143-1160 | PELDSFKEELDKYFKNHT | 1 | 0 | 0 | 0 | 0 | 0 | 0 | 0 | 1 | - | 0 | 0 | 0 | 2 |
| S1144-1161 | 1144 | 1144-1161 | ELDSFKEELDKYFKNHTS | 0 | 1 | 1 | 1 | 1 | 1 | 1 | 1 | 0 | - | 1 | 1 | 0 | 9 |
| S1153-1170 | 1153 | 1153-1170 | DKYFKNHTSPDVDLGDIS | 0 | 0 | 0 | 0 | 0 | 0 | 0 | 0 | 1 | - | 0 | 0 | 0 | 1 |
| S1157-1174 | 1157 | 1157-1174 | KNHTSPDVDLGDISGINA | 1 | 0 | 0 | 0 | 0 | 0 | 0 | 0 | 0 | - | 0 | 0 | 0 | 1 |
| S1158-1175 | 1158 | 1158-1175 | NHTSPDVDLGDISGINAS | 0 | 1 | 1 | 1 | 1 | 1 | 1 | 1 | 0 | - | 1 | 1 | 0 | 9 |
| S1166-1183 | 1166 | 1166-1183 | LGDISGINASVVNIQKEI | 1 | 0 | 0 | 0 | 0 | 0 | 0 | 0 | 0 | - | 0 | 0 | 0 | 1 |
| S1167-1184 | 1167 | 1167-1184 | GDISGINASVVNIQKEID | 0 | 1 | 1 | 1 | 1 | 1 | 1 | 1 | 0 | - | 1 | 1 | 0 | 9 |
| S1175-1192 | 1175 | 1175-1192 | SVVNIQKEIDRLNEVAKN | 1 | 0 | 0 | 0 | 0 | 0 | 0 | 0 | 0 | - | 0 | 0 | 0 | 1 |
| S1176-1193 | 1176 | 1176-1193 | VVNIQKEIDRLNEVAKNL | 0 | 1 | 1 | 1 | 1 | 1 | 1 | 1 | 0 | - | 1 | 1 | 0 | 9 |
| S1188-1205 | 1188 | 1188-1205 | EVAKNLNESLIDLQELGK | 1 | 0 | 0 | 0 | 0 | 0 | 0 | 0 | 0 | - | 0 | 0 | 0 | 1 |
| S1189-1206 | 1189 | 1189-1206 | VAKNLNESLIDLQELGKY | 0 | 1 | 1 | 1 | 1 | 1 | 1 | 1 | 0 | - | 1 | 1 | 0 | 9 |
| S1196-1213 | 1196 | 1196-1213 | SLIDLQELGKYEQYIKWP | 1 | 0 | 0 | 0 | 0 | 0 | 0 | 0 | 0 | - | 0 | 0 | 1 | 2 |
| S1197-1214 | 1197 | 1197-1214 | LIDLQELGKYEQYIKWPW | 0 | 1 | 1 | 1 | 1 | 1 | 1 | 1 | 0 | - | 1 | 1 | 0 | 9 |
| S1203-1220 | 1203 | 1203-1220 | LGKYEQYIKWPWYIWLGF | 1 | 0 | 0 | 0 | 0 | 0 | 0 | 0 | 0 | - | 0 | 0 | 0 | 1 |
| S1204-1221 | 1204 | 1204-1221 | GKYEQYIKWPWYIWLGFI | 0 | 1 | 1 | 1 | 1 | 1 | 1 | 1 | 0 | - | 1 | 1 | 0 | 9 |
| S1213-1230 | 1213 | 1213-1230 | PWYIWLGFIAGLIAIVMV | 1 | 0 | 0 | 0 | 0 | 0 | 0 | 0 | 0 | - | 0 | 0 | 0 | 1 |
| S1214-1231 | 1214 | 1214-1231 | WYIWLGFIAGLIAIVMVT | 0 | 1 | 1 | 1 | 1 | 1 | 1 | 1 | 0 | - | 1 | 1 | 0 | 9 |
| S1223-1240 | 1223 | 1223-1240 | GLIAIVMVTIMLCCMTSC | 1 | 0 | 0 | 0 | 0 | 0 | 0 | 0 | 0 | - | 0 | 0 | 0 | 1 |
| S1224-1241 | 1224 | 1224-1241 | LIAIVMVTIMLCCMTSCC | 0 | 1 | 1 | 1 | 1 | 1 | 1 | 1 | 0 | - | 1 | 1 | 0 | 9 |
| S1235-1252 | 1235 | 1235-1252 | CCMTSCCSCLKGCCSCGS | 1 | 0 | 0 | 0 | 0 | 0 | 0 | 0 | 0 | - | 0 | 0 | 0 | 1 |
| S1236-1253 | 1236 | 1236-1253 | CMTSCCSCLKGCCSCGSC | 0 | 1 | 1 | 1 | 1 | 1 | 1 | 1 | 0 | - | 1 | 1 | 0 | 9 |
| S1237-1254 | 1237 | 1237-1254 | MTSCCSCLKGCCSCGSCC | 0 | 0 | 0 | 0 | 0 | 0 | 0 | 0 | 1 | - | 0 | 0 | 0 | 1 |
| S1241-1258 | 1241 | 1241-1258 | CSCLKGCCSCGSCCKFDE | 1 | 0 | 0 | 0 | 0 | 0 | 0 | 0 | 0 | - | 0 | 0 | 0 | 1 |
| S1242-1259 | 1242 | 1242-1259 | SCLKGCCSCGSCCKFDED | 0 | 1 | 1 | 1 | 1 | 1 | 1 | 1 | 0 | - | 1 | 0 | 0 | 8 |
| S1247-1264 | 1247 | 1247-1264 | CCSCGSCCKFDEDDSEPV | 1 | 0 | 0 | 0 | 0 | 0 | 0 | 0 | 0 | - | 0 | 0 | 0 | 1 |
| S1248-1265 | 1248 | 1248-1265 | CSCGSCCKFDEDDSEPVL | 0 | 1 | 1 | 1 | 1 | 1 | 1 | 1 | 0 | - | 1 | 0 | 0 | 8 |
| S1253-1270 | 1253 | 1253-1270 | CCKFDEDDSEPVLKGVKL | 1 | 0 | 0 | 0 | 0 | 0 | 0 | 0 | 0 | - | 0 | 0 | 0 | 1 |
| S1254-1271 | 1254 | 1254-1271 | CKFDEDDSEPVLKGVKLH | 0 | 1 | 1 | 1 | 1 | 1 | 1 | 1 | 0 | - | 1 | 0 | 0 | 8 |

Supplementary Table S12: Binding affinity prediction for the epistatic epitopes

| Peptide | Mutation | ID | Allele | Core | Score_EL | Rank_EL | Score_BA | nM | Rank_BA | Ave |
| --- | --- | --- | --- | --- | --- | --- | --- | --- | --- | --- |
| QPFLMDLEGKQGNFK | E180V/G184V | WT_3 | DRB1_0101 | LMDLEGKQG | 0.316996 | 4.215598 | 0.531968 | 158.222122 | 15.78719 | 0.034118 |
| QPFLMDLEGKQVNFK | E180V/G184V | MUT_3 | DRB1_0101 | LMDLEGKQV | 0.726311 | 1.240118 | 0.668553 | 36.095943 | 4.614622 | 0.080747 |
| VSQPFLMDLEGKQGN | E180V/G184V | WT_3 | DRB1_0101 | LMDLEGKQG | 0.077203 | 10.413094 | 0.452611 | 373.392883 | 26.58507 | 0.041186 |
| VSQPFLMDLEGKQVN | E180V/G184V | MUT_3 | DRB1_0101 | LMDLEGKQV | 0.315574 | 4.232394 | 0.58145 | 92.630432 | 10.787018 | 0.05847 |
| SQPFLMDLEGKQGNF | E180V/G184V | WT_3 | DRB1_0101 | LMDLEGKQG | 0.153319 | 7.120648 | 0.505753 | 210.112656 | 19.015657 | 0.042727 |
| SQPFLMDLEGKQVNF | E180V/G184V | MUT_3 | DRB1_0101 | LMDLEGKQV | 0.478937 | 2.732846 | 0.635658 | 51.52663 | 6.511273 | 0.06879 |
| PFLMDLEGKQGNFKN | E180V/G184V | WT_3 | DRB1_0101 | LMDLEGKQG | 0.06868 | 10.983231 | 0.482131 | 271.301025 | 22.15064 | 0.008583 |
| PFLMDLEGKQVNFKN | E180V/G184V | MUT_3 | DRB1_0101 | LMDLEGKQV | 0.274097 | 4.756238 | 0.629948 | 54.810493 | 6.913795 | 0.034438 |
| PYRVVVLSPGDVYFA | G252V | WT_5 | DRB1_0101 | VVVLSPGDV | 0.503559 | 2.539873 | 0.666485 | 36.912651 | 4.722076 | 0.08294 |
| PYRVVVLSPVDVYFA | G252V | MUT_5 | DRB1_0101 | VVVLSPVDV | 0.725769 | 1.242818 | 0.718242 | 21.084848 | 2.512171 | 0.100016 |
| PYRVVVLSPGDVYFA | D253G | WT_6 | DRB1_0101 | VVVLSPGDV | 0.503559 | 2.539873 | 0.666485 | 36.912651 | 4.722076 | 0.08294 |
| PYRVVVLSPGGVYFA | D253G | MUT_6 | DRB1_0101 | VVVLSPGGV | 0.610975 | 1.83672 | 0.726681 | 19.244934 | 2.211614 | 0.100389 |
| SFELLHAPATVCGPK | P521S/T | WT_9 | DRB1_0101 | LLHAPATVC | 0.644205 | 1.652729 | 0.845476 | 5.322364 | 0.216155 | 0.066042 |
| SFELLHASATVCGPK | P521S/T | MUT_9 | DRB1_0101 | LLHASATVC | 0.517357 | 2.441949 | 0.821799 | 6.876398 | 0.415841 | 0.055892 |
| FELLHAPATVCGPKK | P521S/T | WT_9 | DRB1_0101 | LLHAPATVC | 0.260009 | 4.947942 | 0.778282 | 11.011517 | 0.984138 | 0.024847 |
| FELLHASATVCGPKK | P521S/T | MUT_9 | DRB1_0101 | LLHASATVC | 0.172661 | 6.615024 | 0.753206 | 14.443716 | 1.485058 | 0.029563 |
| SFELLHAPATVCGPK | P521S/T | WT_10 | DRB1_0101 | LLHAPATVC | 0.644205 | 1.652729 | 0.845476 | 5.322364 | 0.216155 | 0.066042 |
| SFELLHATATVCGPK | P521S/T | MUT_10 | DRB1_0101 | LLHATATVC | 0.5734 | 2.068445 | 0.823291 | 6.766302 | 0.400693 | 0.066564 |
| VSQPFLMDLEGKEGN | E180V | WT_2 | DRB1_0405 | FLMDLEGKE | 0.339077 | 3.819383 | 0.430311 | 475.282043 | 13.482565 | 0.041363 |
| VSQPFLMDLVGKEGN | E180V | MUT_2 | DRB1_0405 | FLMDLVGKE | 0.758204 | 0.913919 | 0.6361 | 51.281048 | 0.712752 | 0.08029 |
| QPFLMDLEGKQGNFK | E180V/G184V | WT_3 | DRB1_1201 | LMDLEGKQG | 0.038626 | 23.470648 | 0.230899 | 4111.319824 | 42.967014 | 0.034118 |
| QPFLMDLEGKQVNFK | E180V/G184V | MUT_3 | DRB1_1201 | LMDLEGKQV | 0.315025 | 3.315944 | 0.397561 | 677.395691 | 10.066605 | 0.080747 |
| KEGNFKNLREFVFKN | E180V/G184V | WT_4 | DRB1_1201 | FKNLREFVF | 0.636564 | 0.756991 | 0.440088 | 427.571716 | 6.050246 | 0.099474 |
| KVGNFKNLREFVFKN | E180V/G184V | MUT_4 | DRB1_1201 | FKNLREFVF | 0.593414 | 0.94452 | 0.463348 | 332.43866 | 4.54643 | 0.079893 |
| PYRVVVLSPGDVYFA | G252V | WT_5 | DRB1_1201 | VVVLSPGDV | 0.485497 | 1.513173 | 0.511585 | 197.264587 | 2.323115 | 0.08294 |
| PYRVVVLSPVDVYFA | G252V | MUT_5 | DRB1_1201 | VVVLSPVDV | 0.548921 | 1.164703 | 0.557085 | 120.571274 | 1.15736 | 0.100016 |
| YRVVVLSPGDVYFAS | G252V | WT_5 | DRB1_1201 | VVLSPGDVY | 0.245098 | 4.661379 | 0.50059 | 222.182983 | 2.712547 | 0.033939 |
| YRVVVLSPVDVYFAS | G252V | MUT_5 | DRB1_1201 | VVVLSPVDV | 0.283275 | 3.850763 | 0.540123 | 144.859558 | 1.496399 | 0.044653 |
| PYRVVVLSPGDVYFA | D253G | WT_6 | DRB1_1201 | VVVLSPGDV | 0.485497 | 1.513173 | 0.511585 | 197.264587 | 2.323115 | 0.08294 |
| PYRVVVLSPGGVYFA | D253G | MUT_6 | DRB1_1201 | VVVLSPGGV | 0.604613 | 0.891713 | 0.567892 | 107.266159 | 0.96687 | 0.100389 |
| YRVVVLSPGDVYFAS | D253G | WT_6 | DRB1_1201 | VVLSPGDVY | 0.245098 | 4.661379 | 0.50059 | 222.182983 | 2.712547 | 0.033939 |
| YRVVVLSPGGVYFAS | D253G | MUT_6 | DRB1_1201 | VVLSPGGVY | 0.37556 | 2.49065 | 0.561446 | 115.014572 | 1.077251 | 0.048817 |
| TFEYVSQPFLMDLEG | E180V | WT_1 | HLA-DPA10103-DPB110101 | YVSQPFLMD | 0.843858 | 0.239555 | 0.702924 | 24.88571 | 0.577248 | 0.130631 |
| TFEYVSQPFLMDLVG | E180V | MUT_1 | HLA-DPA10103-DPB110101 | YVSQPFLMD | 0.792483 | 0.344399 | 0.719162 | 20.87602 | 0.427835 | 0.110978 |
| YRVVVLSFELLHAPA | P521S/T | WT_8 | HLA-DPA10103-DPB110101 | VLSFELLHA | 0.047591 | 12.170294 | 0.60444 | 72.231087 | 2.791387 | 0.032241 |
| YRVVVLSFELLHATA | P521S/T | MUT_8 | HLA-DPA10103-DPB110101 | LSFELLHAT | 0.195165 | 3.866152 | 0.615658 | 63.975357 | 2.367773 | 0.033799 |
| TFEYVSQPFLMDLEG | E180V | WT_1 | HLA-DPA10201-DPB110201 | YVSQPFLMD | 0.410022 | 0.980773 | 0.64606 | 46.041718 | 0.63035 | 0.130631 |
| TFEYVSQPFLMDLVG | E180V | MUT_1 | HLA-DPA10201-DPB110201 | YVSQPFLMD | 0.337411 | 1.405879 | 0.660338 | 39.451122 | 0.470207 | 0.110978 |
| TFEYVSQPFLMDLEG | E180V | WT_1 | HLA-DPA10301-DPB110301 | YVSQPFLMD | 0.353064 | 0.30218 | 0.713449 | 22.207253 | 1.208501 | 0.130631 |
| TFEYVSQPFLMDLVG | E180V | MUT_1 | HLA-DPA10301-DPB110301 | YVSQPFLMD | 0.291589 | 0.540247 | 0.733587 | 17.859308 | 0.742816 | 0.110978 |
| QPFLMDLEGKQGNFK | E180V/G184V | WT_3 | HLA-DQA10501-DQB10501 | LMDLEGKQG | 0.000659 | 29.101702 | 0.112658 | 14777.20215 | 70.926308 | 0.034118 |
| QPFLMDLEGKQVNFK | E180V/G184V | MUT_3 | HLA-DQA10501-DQB10501 | LMDLEGKQV | 0.006616 | 4.393734 | 0.150149 | 9849.681641 | 52.574883 | 0.080747 |
